# Model-based quantification of protein–protein interaction aberrations for exploring dysregulated signalling pathways through pathway maps and gene expression levels

**DOI:** 10.1186/s12859-026-06543-8

**Published:** 2026-07-08

**Authors:** Kenta Kevee Kisaï, Takashi Omori

**Affiliations:** 1 Centre for Liberal Arts / Innovation and Research Support Centre, International University of Health and Welfare, 4-3 Kozunomori, Narita, Chiba 286-8686 Japan; 2 Translational Research Centre for Medical Innovation, Foundation for Biomedical Research and Innovation at Kobe, 1-5-4 Minatojima- minamimachi, Chuo-ku, Kobe, 650-0047 Japan; 3 Graduate School of Heal Data Science, Juntendo University, 6-8-1 Hinode, Urayasu, Chiba 279-0013 Japan; 4 Department of Clinical Biostatistics, School of Public Health, Graduate School of Medicine, Kyoto University, Yoshida Konoe-cho, Sakyo-ku, Kyoto, 606-8501 Japan

**Keywords:** Mathematical model, Aberration quantification, Pathway dysregulation, Protein–protein interactions, Signalling pathways, Pathway maps, Gene expression levels

## Abstract

**Background:**

Protein–protein interactions (PPIs) are fundamental components of signal transduction, and identifying dysregulated pathways is essential for understanding disease mechanisms. Conventional methods use pathway maps and cross‑sectional gene expression data to define sub‑pathways in advance, but this requirement becomes impractical as pathway complexity increases. Herein, rather than attempting to predefine all sub-pathways, we propose an alternative method whereby (1) each PPI constituting pathways is quantitatively evaluated in terms of the extent of aberration, and (2) dysregulated sub-pathways are subsequently explored based on these evaluations.

**Methods:**

To quantitatively evaluate the degree of aberration for each PPI, we constructed a mathematical model, assuming a balance between association and dissociation reactions. The extent of aberration was assessed through a model parameter defined as the difference in signal intensity between diseased and healthy groups, with consideration of protein levels. The proposed method was applied to publicly available data, including the mTOR signalling pathway map and two gene expression datasets—one from clear cell renal cell carcinoma and the other from lung squamous cell carcinoma. A simulation study was also conducted to evaluate its performance.

**Results:**

The proposed method identified PPIs that were also deemed aberrant by HiPathia, the best-performing conventional method, supporting its validity. In addition, our method explored sub-pathways that may be overlooked by predefined approaches, such as HiPathia. Furthermore, a simulation study indicated that the method exhibited sufficient performance for real-world application.

**Conclusion:**

Although our method relies on several strong assumptions, these findings demonstrate that it provides a novel framework for pathway analysis, applicable even to complex pathways when these assumptions are satisfied.

**Graphical Abstract:**

**Figure:**
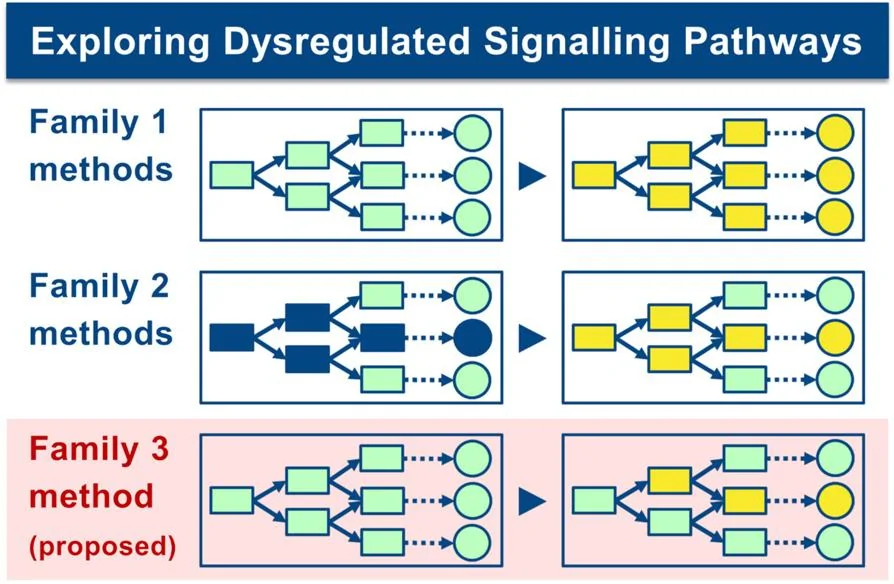

**Supplementary Information:**

The online version contains supplementary material available at https://doi.org/10.1186/s12859-026-06543-8.

## Background

In human cells, signal transduction is a mechanism that converts extracellular signals into a cascade of protein–protein interactions (PPIs), thereby guiding specific cellular functions necessary for homeostasis. Aberrations in these processes are expected to induce cellular dysfunctions, leading to the onset of diseases. The identification of dysregulated signalling pathways is essential for elucidating disease mechanisms and the acceleration of drug discovery.

**Fig. 1:**
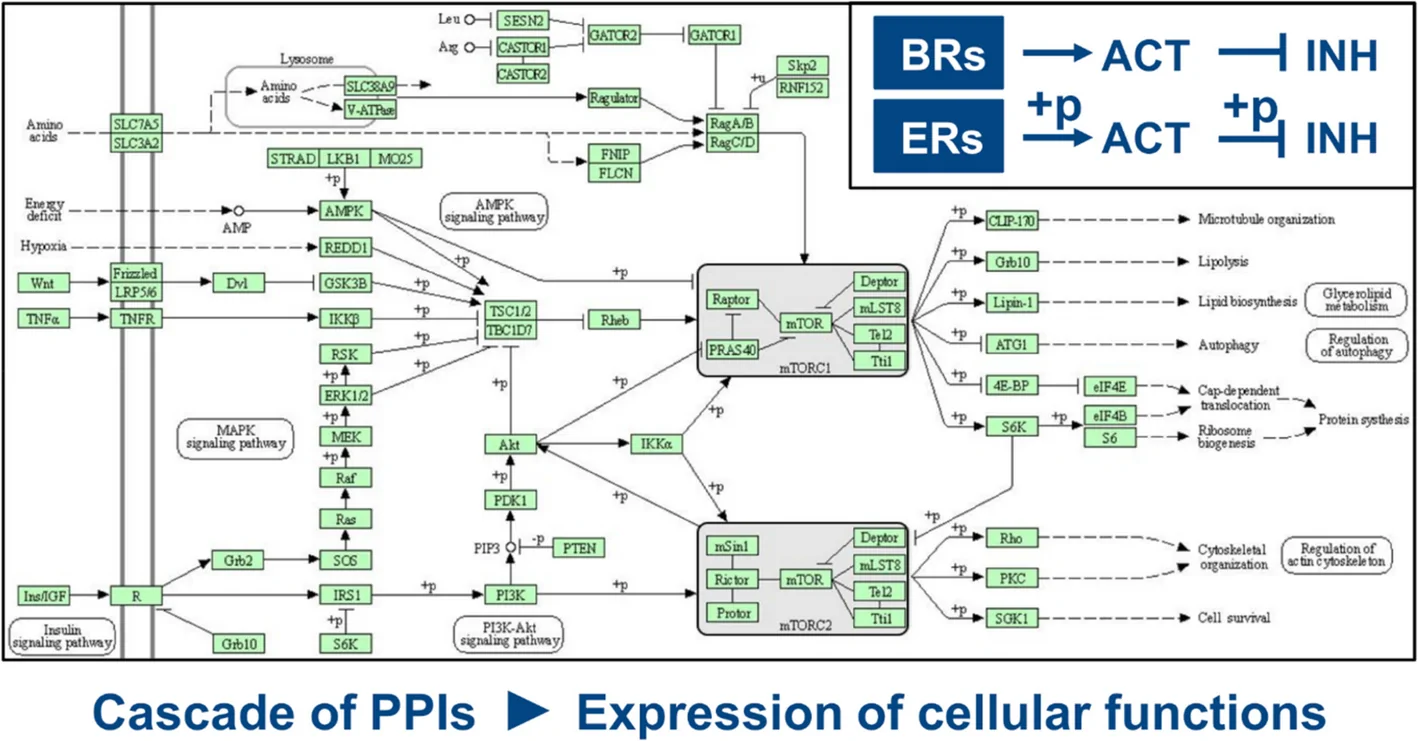
Example of pathway maps from KEGG. The map represents the mTOR signalling pathway. Squares represent proteins. Arrows, which mean activation (ACT), and T-bars, which mean inhibition (INH), represent protein–protein interactions (PPIs). PPIs consist of binding reactions (BRs) and enzymatic reactions (ERs). ERs are denoted by symbols such as ‘+p’. The map illustrates how cellular functions are orchestrated through a cascade of PPIs

Signalling pathways elucidated in basic research have been made publicly available as pathway maps. For instance, the Kyoto Encyclopaedia of Genes and Genomes database (KEGG) [1] provides access to such maps (Fig. 1). Additionally, gene expression levels in diseased (case) and healthy (control) groups have been quantified in cross-sectional studies, with these data being publicly accessible. For example, the National Cancer Institute’s Genomic Data Commons (GDC) Data Portal offers gene expression data from tumour tissue and adjacent normal tissue.

Numerous methods have been proposed for analysing signalling pathways using gene expression levels in case and control groups, with the aim of identifying dysregulated pathways. Early enrichment-based methods detect whether the entire pathway is dysregulated by evaluating the extent to which differentially expressed genes are enriched among the genes constituting the pathway. Building on these approaches, a first family of methods—pathway topology-based or structure-based methods—that take the pathway topology into account have also been proposed [2, 3] (see the upper panel of Graphical Abstract). While these methods detect pathways as a whole, they do not provide information on which sub-pathways to the specific cellular functions are dysregulated. To address this limitation, researchers have introduced a second family of methods—mechanistic pathway activity methods—to detect sub-pathways, which are expected to be better descriptors of specific cellular functions [4] (see the middle panel of the Graphical Abstract). Among existing methods, HiPathia [5] is a representative method due to its high performance in detecting cancer-related sub-pathways using publicly available data [4]. The second family of methods relies on predefinition of the sub-pathways [4]. Therefore, as pathways become more complex, it becomes increasingly difficult to predefine all possible sub-pathways from upstream PPIs to downstream cellular functions. To gain in-depth insights into disease mechanisms and accelerate drug discovery, it is imperative to develop a novel method for exploring dysregulated sub-pathways without requiring predefinition.

We propose a method to evaluate the extent of aberration in each PPI for exploring dysregulated sub-pathways based on a newly constructed mathematical model using pathway maps and gene expression levels for the case and control. By (1) quantitatively evaluating each PPI constituting pathways in terms of the extent of aberration, and (2) subsequently exploring dysregulated sub-pathways based on these evaluations, it becomes possible to find dysregulated sub-pathways that could not be captured by predefined approaches (see the lower panel of the Graphical Abstract). To evaluate the extent of aberration in each PPI, it is essential to construct a mathematical model based on the reaction mechanisms of each PPI, as reproducing these mechanisms in mathematical form enables a more accurate representation of the biological phenomena.

This paper is structured as follows: In the Methods section, the proposed method is presented. In the Results section, an application is provided. The method is applied to publicly available data, including the mTOR signalling pathway map and two datasets of gene expression levels. Similarly, HiPathia is applied to the same data to compare the resulting pathway maps with those obtained by our proposed method. In addition, the performance of our method is evaluated through a simulation study. In the Discussion section, a detailed discussion of the method is provided.

## Methods

### Proposed method

**Fig. 2:**
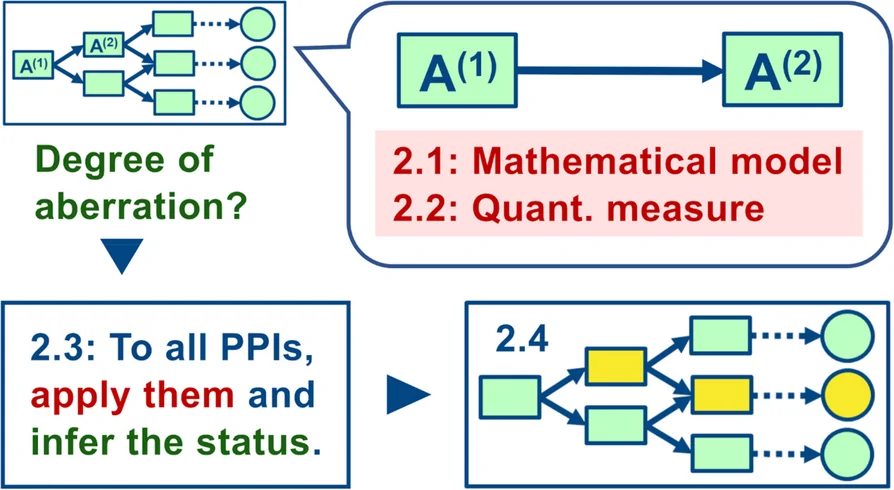
Schematic overview of the proposed method. The method comprises (2.1) a mathematical model and (2.2) a quantitative measure of aberration for each protein–protein interaction (PPI) within a pathway map. (2.3) The extent of aberration for each PPI was estimated using the maximum likelihood method. (2.4) Based on these estimates, dysregulated signalling sub-pathways were explored

As shown in Fig. 2, to explore dysregulated sub-pathways without predefinition, we develop a method as follows. In Sect. 2.1, a mathematical model for each PPI is constructed based on the reaction mechanisms of each PPI, which assumes that association and dissociation are balanced. In Sect. 2.2, a quantitative measure for the aberration of each PPI is developed using the model parameter defined as the difference in signal intensity between the case and control. In Sect. 2.3, the estimation procedure for the measure is explained. In Sect. 2.4, an example of how dysregulated sub-pathways are explored is provided.

The following assumptions are made for pathway maps and gene expression levels. Although the KEGG pathways distinguish between binding and enzyme reactions (Fig. 1), we introduce a PPI theory that does not differentiate between them. Gene expression levels are used as surrogates for the steady-state protein levels as in the first and second families of methods, and are normalised using transcripts per million (TPM) [6]. The case and control are individually matched, with control expression levels derived from adjacent normal solid tissue.

#### Mathematical model for quantifying each PPI aberration

**Fig. 3:**
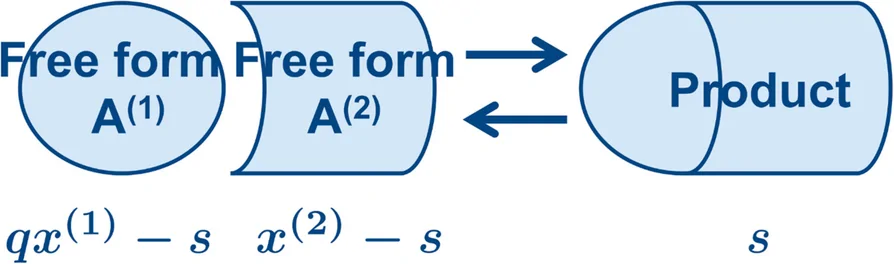
Schematic diagram representing protein–protein interaction (PPI) between proteins A^(1)^ and A^(2)^. The protein levels of A^(1)^ and A^(2)^ are denoted as $\:{x}^{\left(1\right)}$ and $\:{x}^{\left(2\right)}$, respectively. The protein levels of A^(1)^ and A^(2)^, involved in this PPI, are denoted as $\:q{x}^{\left(1\right)}$ and $\:{x}^{\left(2\right)}$, where * q* satisfies * 0<q<1*. The protein level of the product formed by 1:1 binding of the free forms in A^(1)^ and A^(2)^ is denoted as * s*, where * s* satisfies $\:0<s<q{x}^{\left(1\right)}$ and $\:0<s<{x}^{\left(2\right)}$

We focus on an arbitrary PPI as a part of signalling pathways (Fig. 1). The schematic diagram representing the PPI between proteins A^(1)^ and A^(2)^ is shown in Fig. 3. The number in parentheses represents proteins in ascending order along the pathway, proceeding downstream. The total level of the proteins A^(1)^ and A^(2)^ is denoted by $\:{x}^{\left(1\right)}$ and $\:{x}^{\left(2\right)}$, respectively. These data are measured and obtained as publicly available data. The level of A^(1)^, which is determined by upstream PPIs and involved in this focused PPI, is expected to be a portion of $\:{x}^{\left(1\right)}$. Here, with a proportion parameter * q*, representing the involved proportion of $\:{x}^{\left(1\right)}$ to this PPI, where * q* takes values such that * 0<q<1*, the involved level of A^(1)^ to this PPI is denoted as $\:q{x}^{\left(1\right)}$. We assume that the product is the result of 1:1 binding of the free forms in A^(1)^ and A^(2)^, and denote it as * s*, where * s* takes values such that $\:0<s<q{x}^{\left(1\right)}$ and $\:0<s<{x}^{\left(2\right)}$ (Fig. 3). Consequently, the protein levels of free forms of A^(1)^ and A^(2)^ are denoted as $\:q{x}^{\left(1\right)}-s$ and $\:{x}^{\left(2\right)}-s$, respectively.

We introduce the association constant * k* [7], which represents the specific affinity of each PPI. It is assumed that the product is formed in proportion to the protein levels of free forms of A^(1)^ and A^(2)^, and dissociates in proportion to its own level. Furthermore, an equilibrium state is assumed, in which the rates of association and dissociation are balanced. Accordingly, the constant * k*, where * k>0*, is defined as in Eq. (1):

1 $$k\equiv\:\frac{s}{(q{x}^{\left(1\right)}-s)({x}^{\left(2\right)}-s)}$$

* s* in Eq. (1) is unknown. Here, we assume that * s* exists at the upper limit multiplied by a proportion parameter * p*, where * 0<p<1*. If $\:q{x}^{\left(1\right)}$ is the upper limit, * s* is defined as Eq. (2.1):

2.1 $$s\equiv\:pq{x}^{\left(1\right)}$$

If $\:{x}^{\left(2\right)}$ is the upper limit, * s* is defined as Eq. (2.2):

2.2 $$s\equiv\:p{x}^{\left(2\right)}$$

As it is unclear which equation is satisfied, both equations are required to be considered. Rewriting Eq. (1) using Eq. (2.1) and solving for $\:{x}^{\left(2\right)}$ yields Eq. (3.1):

3.1 $${x}^{\left(2\right)}=\frac{p}{k\left(1-p\right)}+pq{x}^{\left(1\right)}$$

Similarly, rewriting Eq. (1) using Eq. (2.2) and solving for $\:{x}^{\left(2\right)}$ yields Eq. (3.2):

3.2 $${x}^{\left(2\right)}=\frac{1}{k\left(p-1\right)}+\frac{q}{p}{x}^{\left(1\right)}$$

  

We assume that the case and control have an identical value of * k*, and satisfy a common Eq. (3.1), but can have different values of * p* and * q* for each PPI. $\:{p}_{1}$ and $\:{q}_{1}$ stand for * p* and * q* in the case, and $\:{p}_{0}$ and $\:{q}_{0}$ stand for in the control. $\:{p}_{1}-{p}_{0}$ is denoted as $\:\delta\:$. The case and control are then expressed in a single regression formulation by incorporating random‑effect and residual‑variability terms, yielding Eq. (4.1):

4.1 $${x}_{ij}^{\left(2\right)}=j\left(\frac{\delta\:+{p}_{0}}{k\left(1-\left(\delta\:+{p}_{0}\right)\right)}+\left(\delta\:+{p}_{0}\right){q}_{1}{x}_{ij}^{\left(1\right)}\right)+\left(1-j\right)\left(\frac{{p}_{0}}{k\left(1-{p}_{0}\right)}+{p}_{0}{q}_{0}{x}_{ij}^{\left(1\right)}\right)+{\gamma\:}_{i}+{\varepsilon}_{ij}$$

which is referred to as Model X1. Similarly, Eq. (3.2) yields the corresponding representation shown in Eq. (4.2):

4.2 $${x}_{ij}^{\left(2\right)}=j\left(\frac{1}{k\left(\delta\:+{p}_{0}-1\right)}+\frac{{q}_{1}}{\delta\:+{p}_{0}}{x}_{ij}^{\left(1\right)}\right)+(1-j)\left(\frac{1}{k\left({p}_{0}-1\right)}+\frac{{q}_{0}}{{p}_{0}}{x}_{ij}^{\left(1\right)}\right)+{\gamma\:}_{i}+{\varepsilon}_{ij}$$

which is referred to as Model X2. In Eqs. (4.1) and (4.2), $\:{x}_{ij}^{\left(1\right)}$ and $\:{x}_{ij}^{\left(2\right)}$ are protein levels and $\:{\varepsilon\:}_{ij}$ is a residual‑variability term representing within-individual variation for the patient * i* in group * j*. * j=1* represents the case, whereas * j=0* represents the control. $\:{\gamma\:}_{i}$ is a random effect term representing between-individual variation to consider that protein levels of the case and control are from identical patient * i*. We assume that $\:{\gamma\:}_{i}$ and $\:{\varepsilon\:}_{ij}$ are independent and follow an independent and identical normal distribution $\:{\gamma\:}_{i}\sim{N}\left(0,\:{\sigma\:}_{\gamma\:}^{2}\right)$, $\:{\sigma\:}_{\gamma\:}>0$, and ${\varepsilon\:}_{ij}\sim{N}\left(0,\:{\sigma\:}_{\varepsilon\:}^{2}\right)$, $\:{\sigma\:}_{\varepsilon\:}>0$, respectively.

#### Quantitative measure for each PPI aberration

We construct a quantitative measure for each PPI aberration based on the model parameter of the case and control. Assuming the control as the standard group and $\:{p}_{j}$ as the parameter with variability, we define $\:{s}_{0j}$ as the magnitude of signal transduction in the group * j*. Referring to Eqs. (2.1) and (2.2), we derive Eqs. (5.1) and (5.2), which correspond to Models X1 and X2, respectively:

5.1 $${s}_{0j}\equiv\:{p}_{j}{q}_{0}\mu\:\left({x}_{i0}^{\left(1\right)}\right)$$

5.2 $${s}_{0j}\equiv\:{p}_{j}\mu\:\left({x}_{i0}^{\left(2\right)}\right)$$

where $\:{q}_{0}\mu\:\left({x}_{i0}^{\left(1\right)}\right)$ and $\:\mu\:\left({x}_{i0}^{\left(2\right)}\right)$ denote the representative fixed values of $\:{q}_{0}{x}_{i0}^{\left(1\right)}$ and $\:{x}_{i0}^{\left(2\right)}$. A quantitative measure * d*, defined as the difference between $\:{s}_{01}$ and $\:{s}_{00}$ of the identical Eq. (5.1) or (5.2), is shown in Eq. (6):

6 $$d\equiv\:\frac{{s}_{01}-{s}_{00}}{v^{\prime}}=\frac{{p}_{1}-{p}_{0}}{v}=\frac{\delta\:}{v}$$

where $\:v{\prime\:}$ represents the standard error of $\:{s}_{01}-{s}_{00}$. Eq. (6) reformulates $\:{s}_{01}-{s}_{00}$ as $\:{p}_{1}-{p}_{0}$ or $\:\delta\:$, and $\:v{\prime\:}$ as * v* of the standard error of $\:\delta\:$. A larger absolute value of * d* suggests a higher degree of aberration in the PPI.

#### Estimation procedure for quantitative aberration measure

To estimate * d* in Eq. (6), we need to estimate $\:\delta\:$ and * v*, based on Eq. (4.1) or (4.2). However, because of the excessive number of parameters in each model, each containing five parameters for three variables, we are unable to estimate them. Here, as $\:{q}_{j}$ is determined by upstream PPIs, we use the upstream information and obtain the estimate $\:{\widehat{q}}_{j}$, as shown in Text S1. Then, we only impose the constraint $\:-1<\delta\:<1$, which is the theoretically acceptable range, and estimate the parameters of * k*, $\:{p}_{0}$, and $\:\delta\:$ with a maximum likelihood method. Maximum likelihood estimation in nonlinear models requires iterative computation. Suitable initial values for * k*, $\:{p}_{0}$, $\:\delta\:$, $\:{\sigma\:}_{\gamma\:}^{2}$, and $\:{\sigma\:}_{\varepsilon\:}^{2}$ are presented in Text S2. Among these candidates, the combination with the largest likelihood is selected as the initial values.

As it is unclear which model, Model X1 or X2, is the true model, model selection is performed. If the absolute value of the log-likelihood ratio between Models X1 and X2 exceeds 1, the model with the higher likelihood is selected, reflecting moderate or more evidence in its support [8]. Otherwise, Model X3, incorporating aspects of both models, is assumed to be the true model. Using the likelihoods of Models X1 and X2 as weights, $\:\left|\left.\widehat{d}\right|\right.$ in Model X3 is calculated as a weighted average.

#### Exploration for dysregulated sub-pathways

We provide an example of how dysregulated sub-pathways are explored. The exploration can be guided by the PPIs with relatively large absolute values of the estimates of * d*. The procedure consists of (1) selecting one or more PPIs located most downstream in pathways (immediately upstream of cellular functions) as endpoints, and (2) tracing upstream from these endpoints toward the initiation PPIs. This approach enables the exploration of dysregulated sub-pathways without requiring predefined sub-pathways.

### Application to publicly available data

We applied the proposed method to publicly available data, including the mTOR signalling pathway map and two gene expression datasets—clear cell renal cell carcinoma (ccRCC) and lung squamous cell carcinoma (LUSC). The objective was to compare the evaluation results of the pathway maps obtained by the proposed method and HiPathia [5]. Furthermore, whether the parameters of the proposed method were appropriately estimated was assessed.

SAS version 9.4 (SAS Institute, Inc., Cary, NC, US) was used. The NLMIXED procedure, with its default settings, was employed to perform maximum likelihood estimation in the nonlinear models described by Eqs. (4.1) and (4.2).

The mTOR signalling pathway from the KEGG database [1] (Fig. 1) was used as a pathway map. Gene expression levels from cross-sectional studies were obtained from the GDC Data Portal. The ccRCC included 72 patients with primary tumour and adjacent normal solid tissue [9], while the LUSC included 51 such patients [10]. These were unstranded-based TPM-normalised data provided as a major output by the GDC Data Portal. The assumptions and creation of the protein levels on each PPI, such as branched (Fig. S1a) or converged (Fig. S1b) structures, are provided in Text S3.

**Fig. 4:**
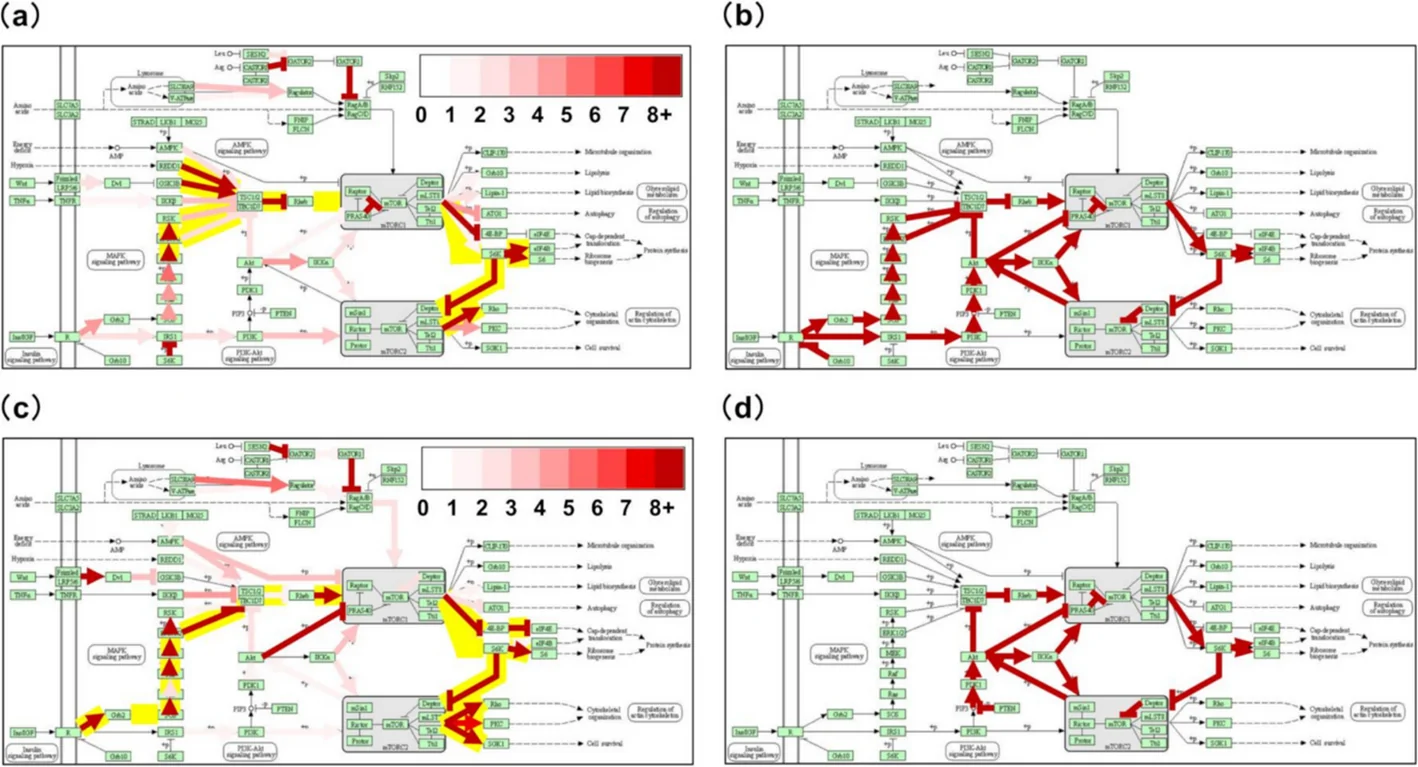
Evaluation of mTOR signalling pathway maps on datasets from (**a**, **b**) clear cell renal cell carcinoma and (**c**, **d**) lung squamous cell carcinoma, using (**a**, **c**) the proposed method and (**b**, **d**) HiPathia. In the proposed method, each protein–protein interaction (PPI) was assigned the absolute value of the estimated quantitative aberration measure, which was then used to determine the colour intensity—larger values were represented by deeper red. An example of explored dysregulated signalling sub-pathways is highlighted with a yellow frame. In HiPathia [5], decomposed sub-pathways with the smallest FDR-adjusted p-values (on the order of 10⁻⁵) obtained from the Wilcoxon signed-rank test were considered aberrant, and the corresponding PPIs are highlighted in red

The proposed method was applied to PPIs, whereas HiPathia was applied to decomposed sub-pathways, each defined as a sequence from a single PPI to a cellular function, using the R package *HiPathia* [11]. In the proposed method, each PPI constituting the pathway map was assigned the absolute value of the estimate of the quantitative aberration measure, which was then used to determine the colour intensity, with larger values represented by deeper red. An example of explored dysregulated signalling sub-pathways was highlighted with a yellow frame (Fig. 4a and c). In HiPathia, the signal intensity of each decomposed sub-pathway was estimated for each patient, and group differences were assessed using the FDR-adjusted p-values obtained from the Wilcoxon signed-rank test [5, 11]. The decomposed sub-pathways with the smallest adjusted p-values (on the order of 10⁻⁵) were considered aberrant, and the corresponding PPIs were highlighted in red (Fig. 4b and d).

To evaluate the agreement between our method and HiPathia, we computed the Jaccard index separately for PPIs classified as aberrant and for those classified as normal under varying detection thresholds. For each detection threshold, the observed Jaccard index was calculated as the proportion of PPIs on which both methods agreed among those labelled by either method. The expected value was computed by assuming independent labelling.

The estimation status of the proposed method was summarised as follows. All PPIs constituting the pathway map were classified as either signal-transducing or non-signal-transducing. Non-signal transduction, in which $\:{x}_{ij}^{\left(1\right)}$ or $\:{x}_{ij}^{\left(2\right)}$ for all patients in both groups was 0, or $\:{\widehat{q}}_{1}$ and $\:{\widehat{q}}_{0}$ were 0, represents that signal transduction failed in both groups, and then $\:\widehat{\delta\:}$ and $\:\widehat{d}$ were assigned a value of 0. The proportion of non-signal transduction was calculated with 59 PPIs as the denominator. Among signal-transducing PPIs, those for which the SAS NLMIXED procedure in Eqs. (4.1) and (4.2) failed to produce estimates were assigned the highest values to avoid overlooking aberrant PPIs. The proportion of this failed estimation was calculated with the number of signal transduction as the denominator. For the estimable PPIs, the proportion of Model X1, X2, or X3 was calculated. When $\:\widehat{v}$ in Model X1 or X2 was 0, we regarded $\:\widehat{v}$ in Model X3 as 0. When $\:\widehat{v}$ was 0, we assigned the highest value to the PPI. For the estimable PPIs, the proportion of $\:\widehat{v}=0$ was calculated.

### Simulation study

We evaluated the performance of the proposed method through a simulation. The objective of the simulation study was to assess its detectability across 48 scenarios imitating publicly available data. Furthermore, the bias in parameter estimates and the estimation status were examined.

The list of 48 scenarios on the PPI of A^(1)^ and A^(2)^ is presented in Table 1. The sample sizes were 40 and 200. $\:{x}_{ij}^{\left(1\right)}$ was created by imitating the protein level in normal solid tissue from ccRCC. We imitated PDK1 in PDK1 → Akt (Fig. 1) as low protein levels (median: 13, interquartile range: 11 to 15) (Fig. S2a and b), while Grb2 in Grb2 → SOS (Fig. 1) as high protein levels (median: 95, interquartile range: 85 to 115) (Fig. S2c and d). $\:{x}_{ij}^{\left(2\right)}$ was generated based on the following conditions. The case and control were matched within the individual patient. Larger $\:{\sigma\:}_{\varepsilon\:}$ was set to a value roughly corresponding to the absolute difference between neighbouring $\:{x}_{ij}^{\left(2\right)}$ and smaller $\:{\sigma\:}_{\varepsilon\:}$ was set to one-fifth of this value. $\:{\sigma\:}_{\gamma\:}$ was set to the same value as the $\:{\sigma\:}_{\varepsilon\:}$. Parameters * k*, $\:{q}_{1}$, $\:{q}_{0}$, and $\:{p}_{1}$ were set to the same values, 1.0, 0.5, 0.5, and 0.5, respectively, across the scenarios. $\:\delta\:$ was set to 0.00, 0.01, and 0.05, reflecting increasing degrees of aberration in PPIs.

**Table 1:** Simulation scenarios for performance of the proposed method

Scenario number		$\:{\boldsymbol{x}}_{\boldsymbol{i}\boldsymbol{j}}^{\left(1\right)}$	Model	$\:{\boldsymbol{\sigma\:}}_{\boldsymbol{\gamma\:}}$	$\:{\boldsymbol{\sigma\:}}_{\boldsymbol{\varepsilon\:}}$	$\:{\boldsymbol{p}}_{1}$	$\:{\boldsymbol{p}}_{0}$	$\:\boldsymbol{\delta\:}$	$\:\left|\boldsymbol{d}\right|$
$\:\boldsymbol{n}=40$	$\:\boldsymbol{n}=200$								
1	25	Low	X1	0.1	0.1	0.5	0.50	0.00	0.0
2	26					0.5	0.49	0.01	> 0.0
3	27					0.5	0.45	0.05	> 0.0
4	28			0.5	0.5	0.5	0.50	0.00	0.0
5	29					0.5	0.49	0.01	> 0.0
6	30					0.5	0.45	0.05	> 0.0
7	31		X2	0.4	0.4	0.5	0.50	0.00	0.0
8	32					0.5	0.49	0.01	> 0.0
9	33					0.5	0.45	0.05	> 0.0
10	34			2	2	0.5	0.50	0.00	0.0
11	35					0.5	0.49	0.01	> 0.0
12	36					0.5	0.45	0.05	> 0.0
13	37	High	X1	0.5	0.5	0.5	0.50	0.00	0.0
14	38					0.5	0.49	0.01	> 0.0
15	39					0.5	0.45	0.05	> 0.0
16	40			2.5	2.5	0.5	0.50	0.00	0.0
17	41					0.5	0.49	0.01	> 0.0
18	42					0.5	0.45	0.05	> 0.0
19	43		X2	2	2	0.5	0.50	0.00	0.0
20	44					0.5	0.49	0.01	> 0.0
21	45					0.5	0.45	0.05	> 0.0
22	46			10	10	0.5	0.50	0.00	0.0
23	47					0.5	0.49	0.01	> 0.0
24	48					0.5	0.45	0.05	> 0.0

Performance was evaluated using detection proportion, bias, and parameter estimation status. Detection proportion was calculated using the proportion for $\:\left|\left.\widehat{d}\right|\right.$ to exceed the detection threshold. Detection threshold was set to 2.0 in this simulation study. Failed estimation was assigned the highest value, as in the application. Bias was calculated as the median of $\:\widehat{\delta\:}$ minus the true value of $\:\delta\:$. When the estimate could not be obtained because of failed estimation, it was excluded from the calculation of the median. When Model X3 was selected, $\:\delta\:$ was estimated as a weighted average of the estimates from Models X1 and X2, using their likelihoods as weights. Parameter estimation status was organised as either estimable or not. The proportion of failed estimation was calculated with the number of simulation iterations as the denominator. The proportion of Model X1, X2, or X3 was calculated using the number of estimable iterations as the denominator. The proportion of $\:\widehat{v}=0$ was also calculated, as in the application.

SAS version 9.4 (SAS Institute, Inc., Cary, NC, US) was used, similar to the application. The simulation iterations for each scenario were set to 1,000. To compare scenarios, we used a common seed value for all scenarios. Even when the generated protein levels of $\:{x}_{ij}^{\left(2\right)}$ became 0 or negative, it was not expected to affect the parameter estimation. Therefore, the estimation was carried out without modification.

## Results

### Application to publicly available data

The highlighted pathway maps are presented in Fig. 4. In ccRCC, the proposed method estimated the extent of aberration for 59 PPIs (Fig. S3a), while HiPathia identified 2 of 51 predefined decomposed sub-pathways (Grb10 to eIF4B and Grb10 to S6). Similarly, in LUSC, our method evaluated 59 PPIs (Fig. S3b) and HiPathia detected 2 of 51 predefined decomposed sub-pathways (PTEN to eIF4B and PTEN to S6). HiPathia detected aberrant PPIs in a continuous manner from upstream PPIs to cellular functions, whereas the proposed method quantified the extent of aberration across all PPIs and subsequently used this information to explore dysregulated sub-pathways (ccRCC: REDD1/GSK3B/MEK to eIF4B/S6/Rho; LUSC: R to eIF4B/S6/Rho/PKC/SGK1). Furthermore, the observed Jaccard indices for aberrant PPIs were 20.6% to 45.7% in ccRCC and 12.9% to 23.4% in LUSC, whereas those for normal PPIs were 34.2% to 49.0% in ccRCC and 25.0% to 56.4% in LUSC. The corresponding expected values are provided in Table S1.

Estimation status in the proposed method is presented in Table 2. Estimable PPIs ranged from 70% to 80% in ccRCC and LUSC. Model X1 had a higher proportion in ccRCC and LUSC compared to Model X2. Model X3 accounted for below 10% in ccRCC and LUSC.

**Table 2:** Parameter estimation status for protein–protein interactions (PPIs) in mTOR signalling pathway, based on clear cell renal cell carcinoma (ccRCC) and lung squamous cell carcinoma (LUSC) datasets

Parameter estimation status	ccRCC	LUSC
Signal-transducing	59 PPIs	59 PPIs
No	0 (0.0%)	1 (1.7%)
Yes	59 (100.0%)	58 (98.3%)
Estimable	59 PPIs	58 PPIs
No	13 (22.0%)	16 (27.6%)
Yes	46 (78.0%)	42 (72.4%)
Selected model	46 PPIs	42 PPIs
Model X1	38 (82.6%)	37 (88.1%)
Model X2	6 (13.0%)	5 (11.9%)
Model X3	2 (4.3%)	0 (0.0%)
$\widehat{v}=0$	1 (2.2%)	1 (2.4%)

Fifty-nine PPIs were classified as either non-signal-transducing or signal-transducing. Non-signal transduction means that $x_{ij}^{\left(1\right)}$ or $x_{ij}^{\left(2\right)}$ for all patients in the case and control was 0, or $\hat{q}_{1}$ and $\hat{q}_{0}$ were 0. Signal-transducing PPIs were further classified as either non-estimable or estimable. Failed estimation means that the SAS NLMIXED procedure in Eqs. (4.1) and (4.2) failed to produce estimates. Estimable PPIs were subsequently categorised as Models X1, X2, and X3

### Simulation study

**Fig. 5:**
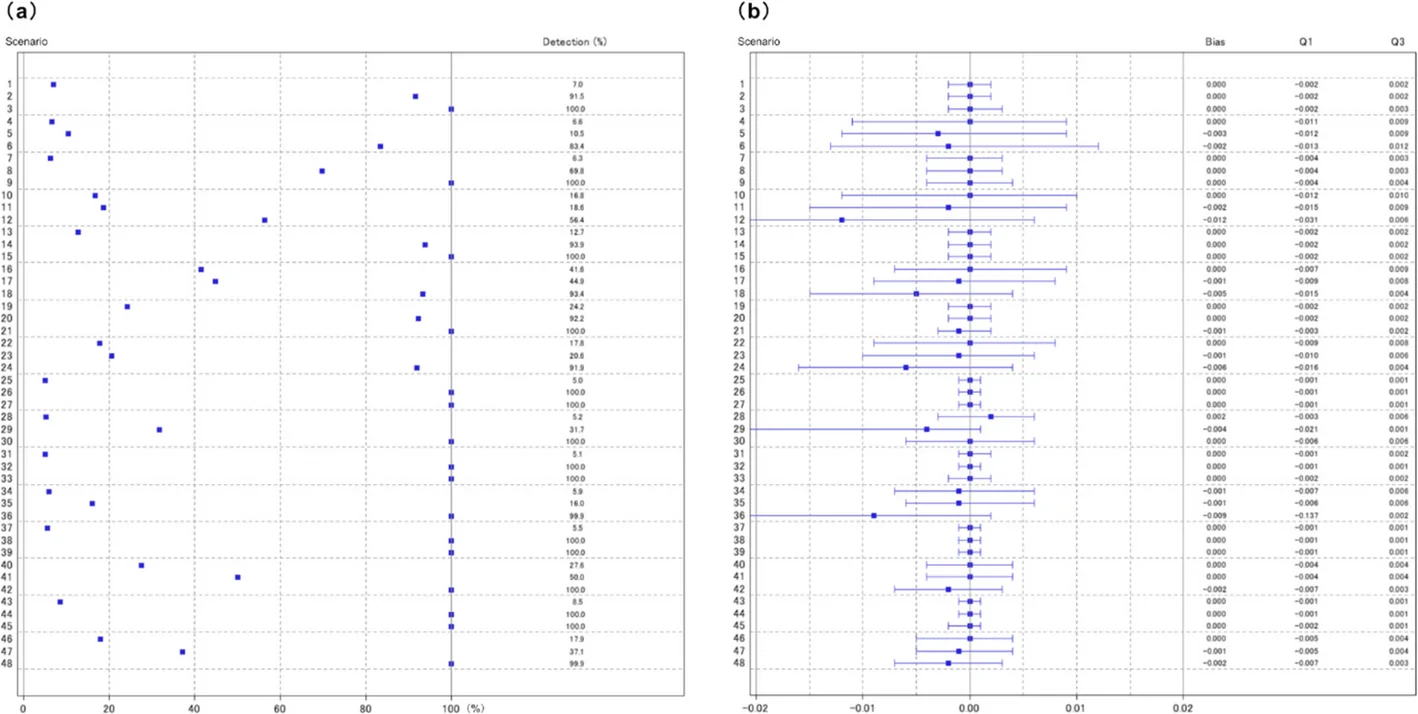
Simulation results for (**a**) detection proportion and (**b**) bias of the proposed method. Detection proportion represents the proportion for $\:\left|\left.\widehat{d}\right|\right.$ to exceed the detection threshold of 2.0. Failed estimation was assigned the highest value to avoid overlooking aberrant protein–protein interactions (PPIs). The bias (filled square) represents the median value of $\:\widehat{\delta\:}$ minus true value of $\:\delta\:$. When the estimate could not be obtained because of failed estimation, it was excluded from the calculation of the median. When Model X3 was selected, $\:\delta\:$ was estimated as a weighted average of the estimates from Models X1 and X2, using their likelihoods as weights. Q1 and Q3 (error bar) represent the first and third quartiles of the bias, respectively

The detection proportion across 48 scenarios is demonstrated in Fig. 5a. The proportion increased as the true value of $\:\delta\:$ rose from 0.00 to 0.01 and 0.05, as observed in Scenario 1, where the corresponding proportions were 7.0%, 91.5%, and 100.0%, respectively. However, when the true value was 0.00, under the smaller sample size, or larger values of $\:{\sigma\:}_{\gamma\:}$ and $\:{\sigma\:}_{\varepsilon\:}$, scenarios in which the detection proportion exceeded 10% were identified. Bias and its interquartile range across 48 scenarios are shown in Fig. 5b. The magnitude of the bias was at most approximately 0.01. The interquartile range was on the order of 10^− 3^ under smaller values of $\:{\sigma\:}_{\gamma\:}$ and $\:{\sigma\:}_{\varepsilon\:}$, and on the order of 10^− 3^ to 10^− 1^ under larger values of them. Dot plots of $\:\left|\widehat{d}\right|$ and $\:\widehat{\delta\:}$ for each scenario are provided in Fig. S4a and b, respectively. Estimable iterations exceeded 70% except for certain scenarios (Fig. S5a), and the model was correctly specified in 80% to 100% in many scenarios (Fig. S5b and c). Simulation results across 48 scenarios for the proportions of Model X3 and $\:\widehat{v}=0$ are shown in Fig. S5d and e, respectively.

## Discussion

In this study, we introduced a novel method for analysing pathway maps using gene expression levels without predefinition. Conventional methods need to predefine sub-pathways analysed [4], but such an approach becomes increasingly infeasible as pathway complexity increases. By constructing a mathematical model for each PPI, we proposed a method to quantitatively evaluate the degree of aberration of each PPI, which enables researchers to explore dysregulated sub-pathways. In application, our method evaluated non-predefined PPIs. Such results enabled the exploration of which PPIs initiate aberrations and lead to specific cellular dysfunctions (Fig. 4). The PPIs consistently identified as aberrant or not by both our method and HiPathia support the validity of our method, whereas the PPIs uniquely explored by our method highlight its potential to uncover dysregulated sub-pathways that may be overlooked by predefined approaches. Additionally, our method demonstrated 70% to 80% estimable PPIs (Table 2). Moreover, simulation results showed that the detection proportion exhibited desirable behaviour in response to increasing levels of aberration (Fig. 5a) and the magnitude of the bias was not excessively large (Fig. 5b). Similar to the application results, more than 70% of iterations were estimable except for certain scenarios (Fig. S5a). Model was correctly specified in 80% to 100% in many scenarios (Fig. S5b and c). These results suggest that the method possesses sufficient performance for real-world applications. Thus, our method contributes to understanding disease mechanisms and enhancing drug discovery, enabling researchers to focus on testing the candidate sub-pathways identified through this method and reducing animal experiments. 

Application and simulation results provide further insights into the proposed method. First, application results reflect known molecular differences between ccRCC and LUSC. In ccRCC, both the proposed method (Fig. 4a) and HiPathia (Fig. 4b) captured mTORC1‑related sub‑pathway activation, consistent with previous reports [12]. In contrast, in LUSC, our method detected Ras–Raf–MEK–ERK activation (Fig. 4c), in line with established mechanisms [13], whereas HiPathia failed to do so (Fig. 4d). These results imply that the proposed method has the potential to recover literature‑supported differences in the dominant signalling axes across cancer types. At the same time, PI3K–Akt signalling, which is important in both cancers, was not detected by our method (Fig. 4a and c) but was identified by HiPathia (Fig. 4b and d), suggesting that there is still room for improvement in our method. Second, the observed Jaccard indices exceeded the random expectation in ccRCC, whereas in LUSC they were comparable to or lower than the random expectation (Table S1). This pattern implies that, in ccRCC, the two methods capture a shared subset of both aberrant and normal PPIs, whereas in LUSC they tend to capture distinct sets of PPIs for both aberrant and normal PPIs, suggesting that, depending on the disease context, our method could identify aberrant PPIs that are not captured by conventional methods. Third, Model X1 was selected more frequently in ccRCC and LUSC compared to Model X2 (Table 2), implying that PPIs favour Model X1. Model X3 accounted for below 10% in ccRCC and LUSC (Table 2), suggesting that model selection based on likelihoods performs well. Other discussions on application and simulation results are presented in Text S4 and S5, respectively.

We note two considerations relevant to the applicability of the framework. First, although we used individually matched case–control data because such self‑controlled designs minimise confounding, the proposed method can also be applied to unmatched datasets by removing the individual‑level random effect term $\:{\gamma\:}_{i}$ from Eq. (4.1) or (4.2). Second, another direction for future work is to consider the applicability of the framework to metabolic pathways, which depend more on substrate and product abundance and often function closer to equilibrium.

Some limitations exist in the proposed method. First, the mathematical model depends on three strong assumptions that restrict the method to practical use during the exploratory phase: (1) protein levels in each PPI are constant and substituted with gene expression levels measured at a single time point; (2) each PPI is in an equilibrium state; and (3) each PPI is modelled only based on protein levels and groups, as described in detail in Text S6. Therefore, wet‑lab experiments conducted under conditions that closely mimic the human biological environment are indispensable for determining whether the sub‑pathways identified during the exploratory phase are truly dysregulated. Second, our current model treats each PPI independently. However, branched (Fig. S1a) and converged (Fig. S1b) PPIs are not fully independent. Developing a more realistic modelling strategy that incorporates such dependencies represents an important direction for future work. Third, in the current procedure, * q* is estimated using the upstream expression levels, which provides a practical way to avoid over‑parameterisation (Text S1). However, this procedure relies on weakly justified, model‑external assumptions, indicating that there remains substantial room for improvement.

Other limitations were revealed in the application and simulation. First, in the application, between 70% and 80% of PPIs were successfully estimated, whereas the remaining PPIs could not be fitted by the SAS NLMIXED procedure. To avoid missing potentially aberrant PPIs, we assigned the highest aberration values to these PPIs. Although this conservative treatment prevents false negatives, it is statistically unsatisfying, and achieving stable estimation for all PPIs remains an important future work. Second, whereas some PPIs continuously exhibited high levels of aberration in ccRCC (Fig. 4a) and LUSC (Fig. 4c), suggesting that these regions are likely to be truly dysregulated, other PPIs that exhibited high aberration levels discontinuously made the exploration more difficult. Such discontinuous patterns may arise from pathway redundancy, where the signal can be compensated by alternative pathways [14], and therefore are not necessarily noise but can reflect biologically plausible mechanisms. Researchers should take these possibilities, as well as previous findings, into account when interpreting the results. Third, although the proposed method evaluates aberrations at the PPI level, HiPathia operates at predefined sub‑pathway levels. However, no gold standard exists for aberrations at either level, making it infeasible to calculate sensitivity or specificity for either method. Therefore, the detectability was assessed through comparative application with HiPathia and a simulation study. The future availability of a reliable definition would enable sensitivity and specificity to be evaluated based on real-world data. Another limitation is that although the present study focuses on representative settings, broader applications remain an important avenue. Future work will evaluate the method on more complex pathway maps and on gene expression datasets with smaller sample sizes. Finally, simulation results showed that the detection proportion was relatively high under the smaller sample size, or the larger values of $\:{\sigma\:}_{\gamma\:}$ and $\:{\sigma\:}_{\varepsilon\:}$ when the true value of $\:\delta\:$ was 0.00 (Fig. 5a). The interquartile range of the bias was somewhat wide under larger values of $\:{\sigma\:}_{\gamma\:}$ and $\:{\sigma\:}_{\varepsilon\:}$ (Fig. 5b). These findings indicate a potential limitation in sensitivity and specificity of our method.    

## Conclusions

Without requiring predefinition of sub‑pathways analysed, which defines the third family of pathway‑analysis methods based on case and control gene expression data, the proposed method enables detailed exploration of dysregulated signalling pathways, thereby providing a practical option even for complex pathways. The proposed method relies on several strong assumptions. However, when these assumptions are satisfied, researchers can estimate the aberrant extent of signal transduction for each PPI by preparing pathway maps and fitting the model to case–control gene expression levels. Application and simulation results indicated that our method exhibits sufficient potential performance for real-world applications. Researchers can select the appropriate method depending on how detailed exploration needs to be, comparing first [2, 3], second [4], and third families of methods (see Graphical Abstract).

## Supplementary Information

Supplementary Material 1.

Supplementary Material 2.

Supplementary Material 3.

## Data Availability

The data of clear cell renal cell carcinoma (ccRCC) and lung squamous cell carcinoma (LUSC) were obtained through the Cancer Genome Atlas Program (TCGA) and provided via GDC Data Portal (ccRCC: https://portal.gdc.cancer.gov/projects/TCGA-KIRC, last accessed on 26 March 2024, LUSC: https://portal.gdc.cancer.gov/projects/TCGA-LUSC, last accessed on 6 July 2024). The processed datasets for application are available at https://1drv.ms/f/c/23d1960af8732973/IgAS2kPHiXHhT5Jjrd_XpzpMAYjuY_PktKGwmrDBAOPGIBE?e=F3aU5l. The SAS programs used for application and simulation are available at https://github.com/zakuzakugohan-kisai/Exploring-Dysregulated-Signalling-Pathways-2025.

## Abbreviations

ccRCC: clear cell renal cell carcinoma
FDR: False Discovery Rate
GDC: Genomic Data Commons
KEGG: Kyoto Encyclopaedia of Genes and Genomes
LUSC: lung squamous cell carcinoma
PPI: Protein–protein interaction
TPM: Transcripts per million
