# Supplementary material for: Model-based quantification of protein–protein interaction aberrations for exploring dysregulated signalling pathways through pathway maps and gene expression levels
Source: BMC Bioinformatics. 2026 Jul 8;27:263. doi: 10.1186/s12859-026-06543-8 (PMC13732913; doi:10.1186/s12859-026-06543-8)
Supplement: Supplementary file 1 [file 12859_2026_6543_MOESM1_ESM.pdf]

Model-based quantification of protein–protein interaction aberrations for exploring dysregulated signalling pathways through pathway maps and gene expression levels

Kenta Kevee Kisaï [1] and Takashi Omori [2]

[1] Centre for Liberal Arts / Innovation and Research Support Centre, International University of Health and Welfare, 4-3 Kozunomori, Narita, Chiba 286-8686, Japan

[2] Translational Research Centre for Medical Innovation, Foundation for Biomedical Research and Innovation at Kobe, 1-5-4 Minatojima-minamimachi, Chuo-ku, Kobe 650-0047, Japan

E-mail: kisai.zakuzakugohan@gmail.com; ORCID: https://orcid.org/0000-0002-8046-9276

[3] Graduate School of Heal Data Science, Juntendo University, 6-8-1 Hinode, Urayasu, Chiba 279-0013, Japan

[4] Department of Clinical Biostatistics, School of Public Health, Graduate School of Medicine, Kyoto University, Yoshida Konoe-cho, Sakyo-ku, Kyoto 606-8501, Japan

E-mail: t.omori.kf@juntendo.ac.jp; ORCID: https://orcid.org/0000-0001-5615-6811

Corresponding author: Kenta Kevee Kisaï

Supplementary Texts

Text S1 Estimation procedure for q_j.

Based on an activation protein–protein interaction (PPI) between $A^{(0)}$ and $A^{(1)}$, which lies upstream of the PPI between $A^{(1)}$ and $A^{(2)}$, q_j is estimated using a non-parametric approach. Assume that $x_{ij}^{(0)}$ is involved in $A^{(0)}$ and $A^{(1)}$. Moreover, if $x_{ij}^{(0)}$ is larger than $x_{ij}^{(1)}$, assume that $x_{ij}^{(1)}$ is involved in $A^{(1)}$ and $A^{(2)}$. Otherwise, assume that $x_{ij}^{(0)}$ is involved. $\hat{q}_j$ is obtained using Equation (S1.1):

$$\hat{q}_j = \frac{\sum_{i:\, x_{ij}^{(0)} \geq x_{ij}^{(1)}} 1 + \sum_{i:\, x_{ij}^{(0)} < x_{ij}^{(1)}} \frac{x_{ij}^{(0)}}{x_{ij}^{(1)}}}{n_j} \tag{S1.1}$$

where n_j is the sample size in group j, and $\hat{q}_j$ is allowed to take the boundary values 0 and 1, even though the theoretical parameter q_j is restricted to the open interval (0,1). If $A^{(0)}$ and $A^{(1)}$ form an inhibition PPI, let $\hat{q}_j$ be the value obtained by subtracting the right-hand side of Equation (S1.1) from 1. If $A^{(0)}$ does not exist, q_j is estimated based on $A^{(1)}$ and $A^{(2)}$ using Equation (S1.2):

$$\hat{q}_j = \frac{\sum_{i:\, x_{ij}^{(1)} > x_{ij}^{(2)}} \frac{x_{ij}^{(2)}}{x_{ij}^{(1)}} + \sum_{i:\, x_{ij}^{(1)} \leq x_{ij}^{(2)}} 1}{n_j} \tag{S1.2}$$

Text S2 Initial value candidates for maximum likelihood estimation in a nonlinear model.

To find suitable initial values for maximum likelihood estimation, let k vary by group j and denote it as k_j. Then, Equations (4.1) and (4.2) for group j can be expressed using α_j and β_j, as defined in Equations (S2.1) and (S2.2):

$$\alpha_j = \frac{p_j}{k_j(1 - p_j)}, \ \beta_j = \hat{q}_j p_j \tag{S2.1}$$

$$\alpha_j = \frac{1}{k_j(p_j - 1)}, \ \beta_j = \frac{\hat{q}_j}{p_j} \tag{S2.2}$$

Both linear regression models become Equation (S2.3):

$$x_{ij}^{(2)} = \alpha_j + \beta_j x_{ij}^{(1)} + \varepsilon_{ij} \tag{S2.3}$$

Using ordinary least squares, obtain the estimates of $\tilde{\alpha}_j$ and $\tilde{\beta}_j$ for α_j and β_j. Based on Equations (S2.1) and (S2.2), obtain $\tilde{k}_j$ and $\tilde{p}_j$ as in Equations (S2.4) and (S2.5):

$$\tilde{k}_j = \frac{\tilde{p}_j}{\tilde{\alpha}_j(1 - \tilde{p}_j)}, \ \tilde{p}_j = \frac{\tilde{\beta}_j}{\hat{q}_j} \tag{S2.4}$$

$$\tilde{k}_j = \frac{1}{\tilde{\alpha}_j(\tilde{p}_j - 1)}, \ \tilde{p}_j = \frac{\hat{q}_j}{\tilde{\beta}_j} \tag{S2.5}$$

Include $\tilde{k}_j$ and $\tilde{p}_j$ in Equation (S2.4) as candidates for initial values of k and p_j in Equation (4.1), and include $\tilde{k}_j$ and $\tilde{p}_j$ in Equation (S2.5) as candidates for initial values of k and p_j in Equation (4.2). The initial values for k, p_0, δ, σ_γ^2, and σ_ε^2 are set to $k = \tilde{k}_1, \tilde{k}_0$, 1, $p_0 = \tilde{p}_0$, 0.5, $\delta = \tilde{p}_1 - \tilde{p}_0$, 0.0, σ_γ^2 =10^{-4}, 10^{-3}, 10^{-2}, 10^{-1}, 10^{0}, 10^{1}, 10^{2}, 10^{3}, 10^{4}, and σ_ε^2 =10^{-4}, 10^{-3}, 10^{-2}, 10^{-1}, 10^{0}, 10^{1}, 10^{2}, 10^{3}, 10^{4}, respectively.

Text S3 Handling of gene expression data in application.

Unstranded gene expression data, obtained as the principal output from the GDC Data Portal, were employed in the application. Batch effects were not considered when integrating data across samples. For more accurate analysis, it may be desirable to use strand-specific expression data and apply an appropriate batch correction method.

Protein levels were substituted with gene expression levels linked to the KEGG database [1]. In our analysis, we applied the following assumptions regarding gene expression levels. (1) When a protein was encoded by multiple genes, their expression levels were summed. (2) Genes that were absent from the reference list were assigned an expression level of zero. (3) Expression levels corresponding to readthrough genes were excluded.

Data processing was performed as follows. (1) For protein complexes, the minimum level among the constituent proteins was used. (2) When the same proteins appeared at multiple locations within the pathway maps, connections between these instances were established wherever possible. In cases where such connections were not feasible—for example, when the same proteins were part of distinct protein complexes—its gene expression levels were equally distributed among the occurrences. Additionally, (3) the data were processed under the assumption of the 1:1 reaction as needed.

In branched (Fig. S1a) or converged (Fig. S1b) protein–protein interactions (PPIs), where a single protein interacted with multiple downstream or upstream proteins, respectively, we assumed equal involvement in each PPI and distributed the expression level equally. Therefore, in Text S1, when $A^{(1)}$ interacted with multiple upstream proteins corresponding to $A^{(0)}$, we estimated q_j as the simple average of the estimates derived from each converged PPI.

Text S4 Discussion on parameter estimation in application results.

To account for the assumption that ε_{ij} follows a normal distribution, we attempted to apply a natural logarithmic transformation to $x_{ij}^{(1)}$ and $x_{ij}^{(2)}$. However, failed estimation increased from 22.0% to 30.5% in ccRCC and from 27.6% to 37.9% in LUSC. This is likely because of the difficulty in fitting the model to the data, caused by compressing the scale of the protein levels. Therefore, we estimated the parameters without applying a logarithmic transformation.

The constraint on model parameters was imposed solely on δ in Equation (4.1) or (4.2). This estimation method was adopted to account for the inherent variability of q_j, which was treated as a fixed parameter. When $\hat{k}$ or $\hat{p}_0$ violated their constraints ($k > 0$ or $0 < p_0 < 1$), we attributed this to the statistical variability of q_j. For example, $\hat{k} \leq -1$, $\hat{p}_0 \leq -0.1$, or $\hat{p}_0 \geq 1.1$ was considered a substantial deviation. In ccRCC and LUSC, the proportion of substantially deviated cases among estimable protein–protein interactions (PPIs) was less than 5%.

Parameter estimation status provides insight into the performance of the proposed method (Table 2). Non-signal transduction was nearly 0% in ccRCC and LUSC, suggesting that protein abundance involved in protein–protein interactions (PPIs) is, to some extent, essential for signal transduction. This implies that regulating these protein levels may contribute to maintaining proper signalling. $\hat{v} = 0$ was less than 10% in ccRCC and LUSC, indicating that the estimation of δ near its boundary values is infrequent among estimable PPIs.

Text S5 Discussion on performance evaluation in simulation results.

Bias tended to shift from 0.000 to negative values as the true values of δ increased from 0.00 to 0.01 or 0.05 (Fig. 5b). Similarly, $\hat{\delta}$ tended to be distributed in a direction smaller than the true value as the true values increased from 0.00 to 0.01 or 0.05 (Fig. S4b). These results suggest that the larger the absolute true value of δ, the more likely it is to be underestimated.

More than 70% of iterations were estimable except for certain scenarios (Fig. S5a). Under a condition with the smaller sample size, the higher protein levels of $x_{ij}^{(1)}$, Model X1, and the larger σ_γ and σ_ε, failed estimation was more than 30%. An increased prevalence of protein–protein interactions (PPIs) under this condition may hinder the exploration of dysregulated pathways using the proposed method.

Model was correctly specified in 80% to 100% in many scenarios (Fig. S5b and c). However, under the lower protein levels of $x_{ij}^{(1)}$ in the true value of 0.00 or 0.01, model misspecification in some scenarios exceeded 20%. Moreover, under the larger sample size, the lower protein levels of $x_{ij}^{(1)}$, Model X2, and the larger values of σ_γ and σ_ε in the true value of 0.05, model misspecification exceeded 20%. In these scenarios, Model X3 was frequently selected (Fig. S5d). Therefore, model selection based on likelihood may lead to model misspecification. Although the application results suggest that likelihood-based model selection works well, these simulation results indicate that it does not perform well depending on the conditions. Additionally, in these scenarios, the proportion of model misspecification was higher when the sample size was large compared to when it was small. Although these phenomena remain unclear, the performance in terms of detection proportion and bias is generally acceptable. Therefore, the influence on the evaluation of the aberration measure is expected to be minimum.

$\hat{v} = 0$ was less than 2% in all scenarios (Fig. S5e). Similar to the application results,

this result suggests that the estimation of δ near its boundary values occurs with very low frequency.

Text S6 Three key assumptions underlying the proposed mathematical model.

We assumed that protein levels in each protein–protein interaction (PPI) are constant and substituted with gene expression levels measured at a single time point. Although mRNA levels do not always correlate with protein levels—especially for signalling proteins with rapid turnover or extensive post-translational regulation—this assumption is also adopted in conventional methods [2]. Moreover, performance comparison between conventional methods [2] employed the trimmed mean of M-values (TMM) [3] for normalisation. However, as TMM does not consider the sequence length of mRNA, it may fail to capture the relative expression levels between genes accurately. Thus, in our proposed model, gene expression levels were normalised using transcripts per million (TPM) [4].

We assumed an equilibrium state for each PPI in Equation (1). Given that cells tend to maintain stable states in signal transduction [5], this assumption may be applicable in the context of chronic disease. Therefore, the proposed method is recommended for use in chronic diseases. Furthermore, if this assumption is valid, it might also be applicable to feedback loops such as the one shown in Fig. 1 (Akt → IKKα → mTORC2 → Akt).

We assumed that each PPI can be modelled solely based on protein levels and groups, as shown in Equations (4.1) and (4.2). Because signal transduction is regulated through multiple layers, including both protein abundance and post-translational modifications, this assumption captures only one aspect of pathway regulation. Our mathematical model also does not distinguish between binding and enzymatic reactions, nor between activation and inhibition (Fig. 1). A possible direction for future work would be to increase the model’s expressiveness by incorporating these pieces of information. Moreover, while the association constant is typically introduced using concentrations [6], we defined it based on protein levels, because of an identical intracellular volume among proteins. Furthermore, we assumed

that the association constant is identical between the case and control, implying that the affinity [6] between the two interacting proteins is the same in both groups. However, due to splice variants, genetic mutations, or post-translational modifications, the association constant may vary between the groups.

Supplementary Figures

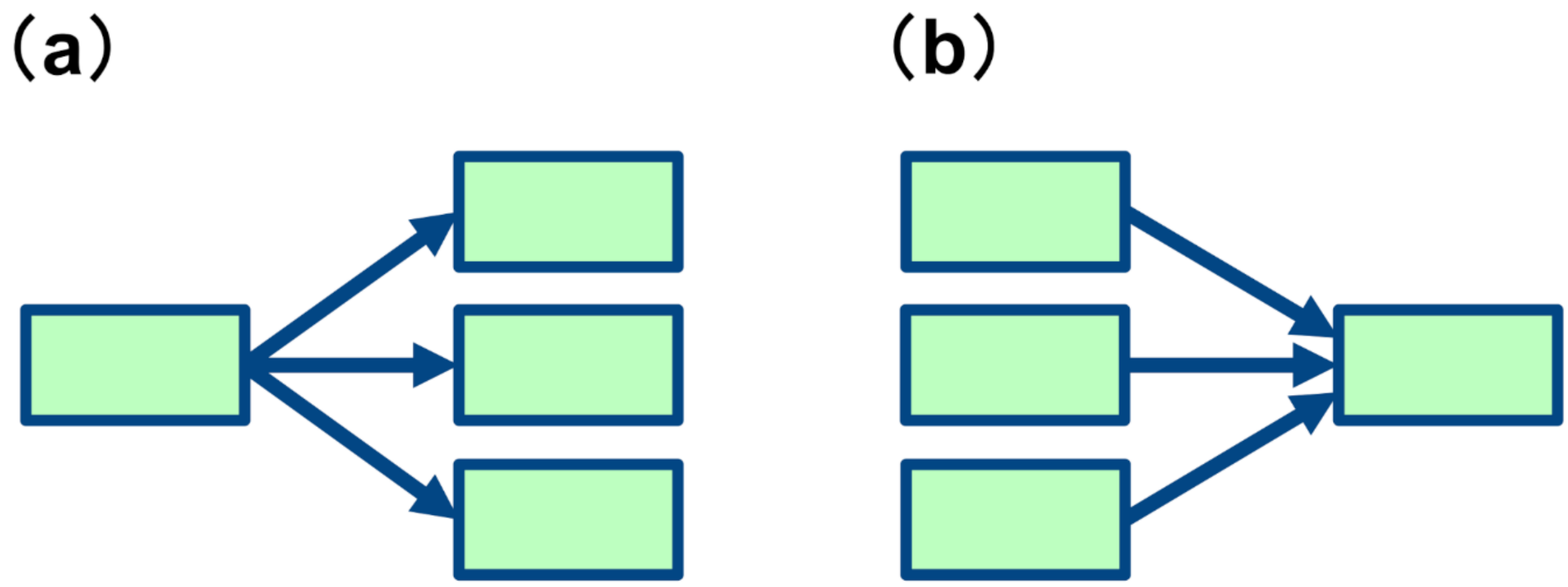

Fig. S1 Example of (**a**) branched and (**b**) converged protein–protein interactions (PPIs). Squares represent proteins and arrows represent PPIs.

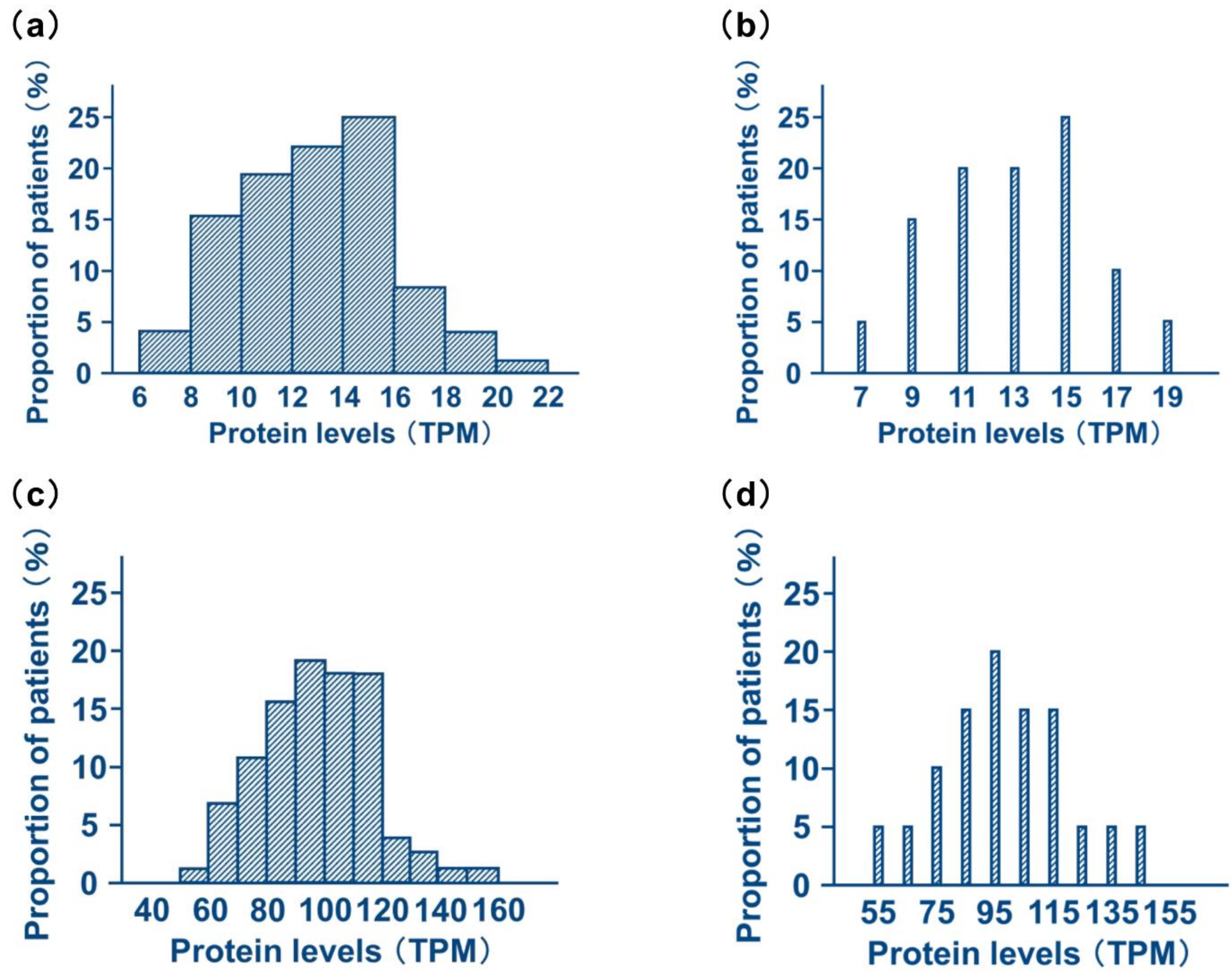

Fig. S2 Distributions of $x_{ij}^{(1)}$ for (**a**, **b**) low and (**c**, **d**) high protein levels, and (**a**, **c**) Publicly available data from normal solid tissues in clear cell renal cell carcinoma and (**b**, **d**) the imitated simulation data. Low protein levels of $x_{ij}^{(1)}$ imitated PDK1 in protein–protein interaction (PPI) of PDK1 → Akt (median: 13, interquartile range: 11 to 15), whereas high protein levels of $x_{ij}^{(1)}$ imitated Grb2 in PPI of Grb2 → SOS (median: 95, interquartile range: 85 to 115) on the mTOR signalling pathway map (Fig. 1). TPM represents transcripts per million.

(a)

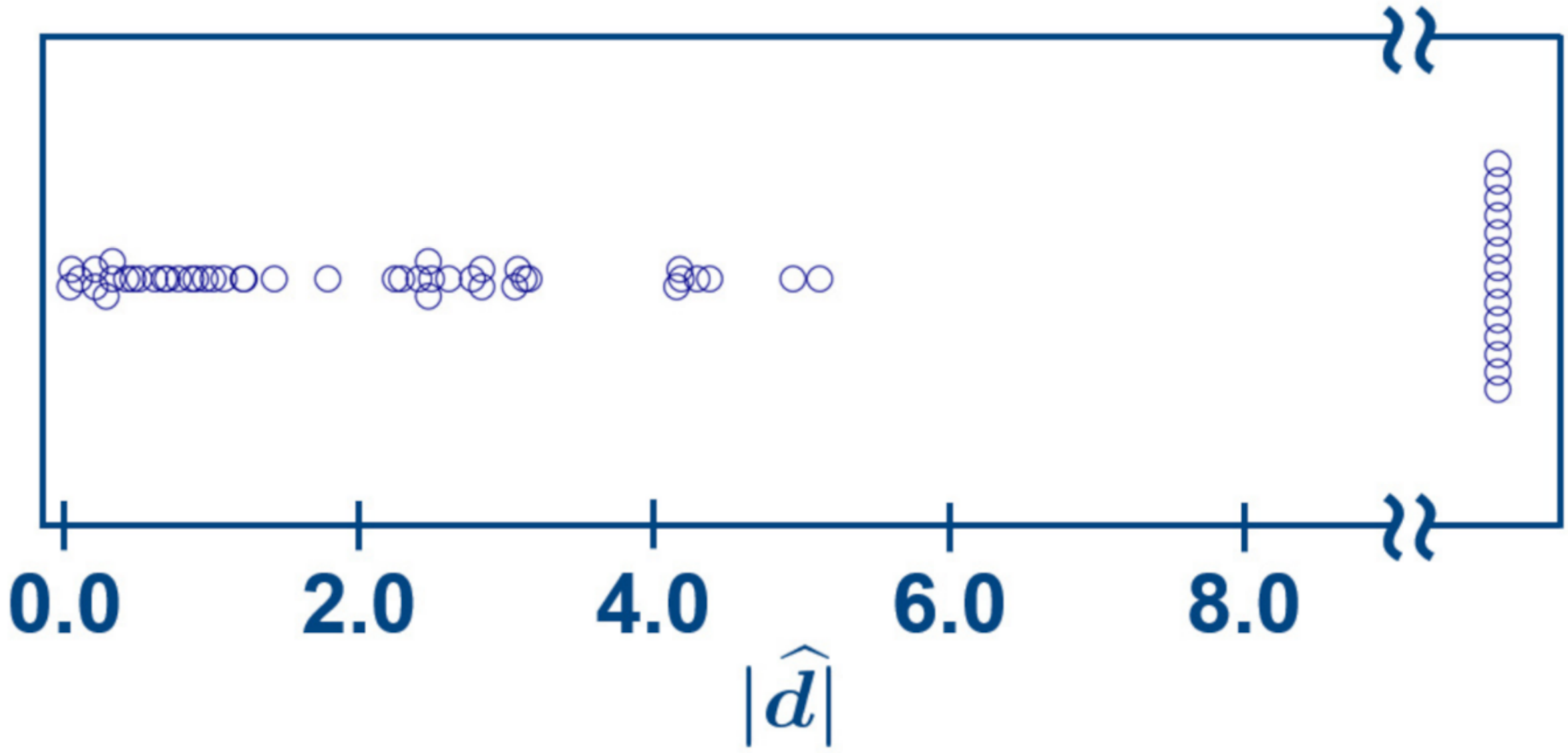

(b)

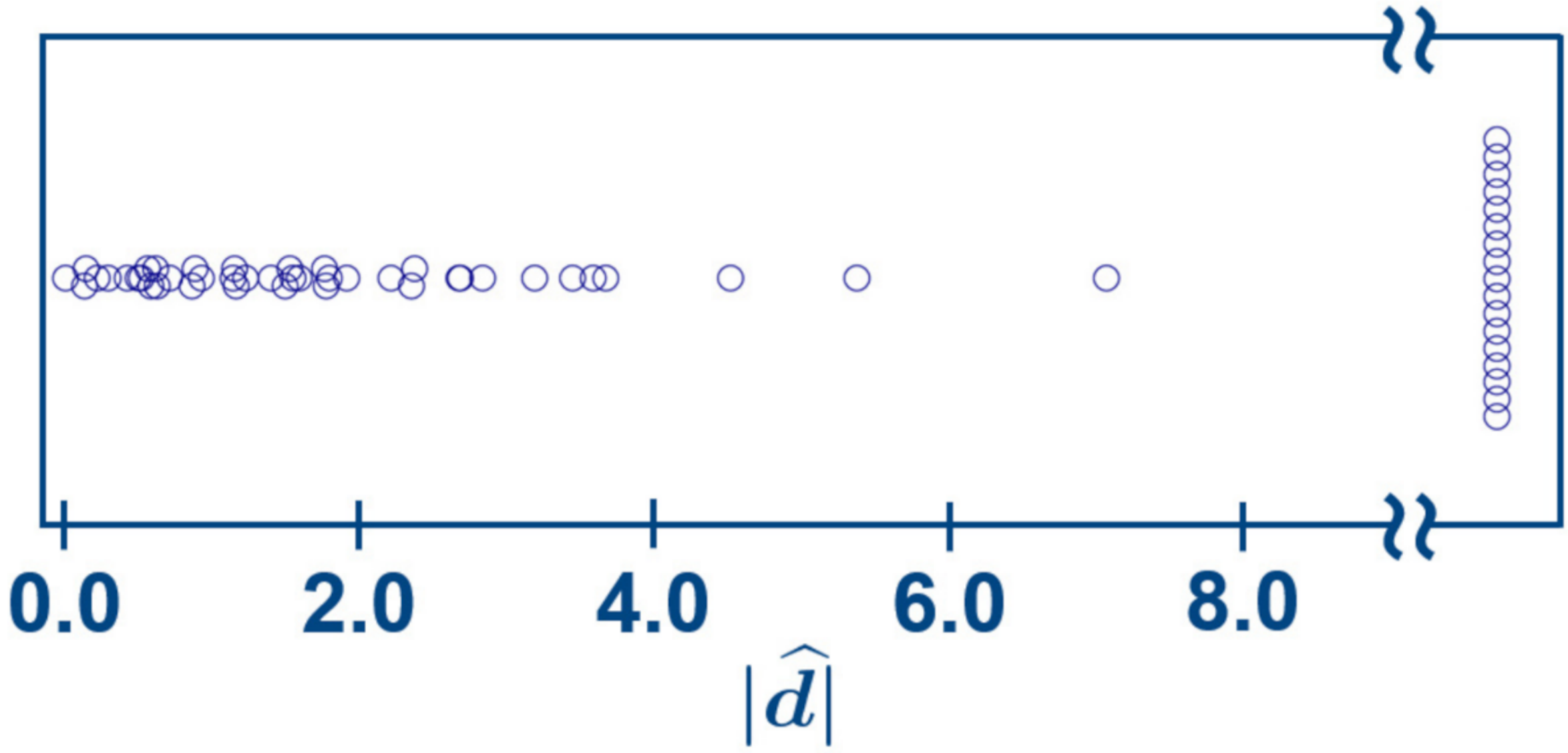

Fig. S3 Dot plots of $|\hat{d}|$ in each protein–protein interaction (PPI). The proposed method was applied to publicly available data, including the mTOR signalling pathway map and two gene expression datasets—(**a**) clear cell renal cell carcinoma and (**b**) lung squamous cell carcinoma. When $|\hat{d}|$ could not be obtained due to failed estimation or $\hat{v} = 0$, it was plotted on the right side.

Fig. S4 Dot plots of (**a**) $|\hat{d}|$ and (**b**) $\hat{\delta}$ for each scenario. In dot plots of $|\hat{d}|$, a reference line was drawn at the detection threshold of 2.0. Plots were coloured by failed estimation, Models X1, X2, and X3. When $|\hat{d}|$ was larger than 8.0, it was plotted as 8.0 for convenience. When $|\hat{d}|$ could not be obtained due to $\hat{v} = 0$, it was plotted as 9.0 for convenience. When $|\hat{d}|$ could not be obtained due to failed estimation, it was plotted as 10.0 for convenience. In dot plots of $\hat{\delta}$, a reference line was drawn at the true value of δ. Plots were coloured by Models X1, X2, and X3. When $\hat{\delta}$ could not be obtained due to failed estimation, it was not plotted. The scenario number corresponds to that in Table 1.

(a)

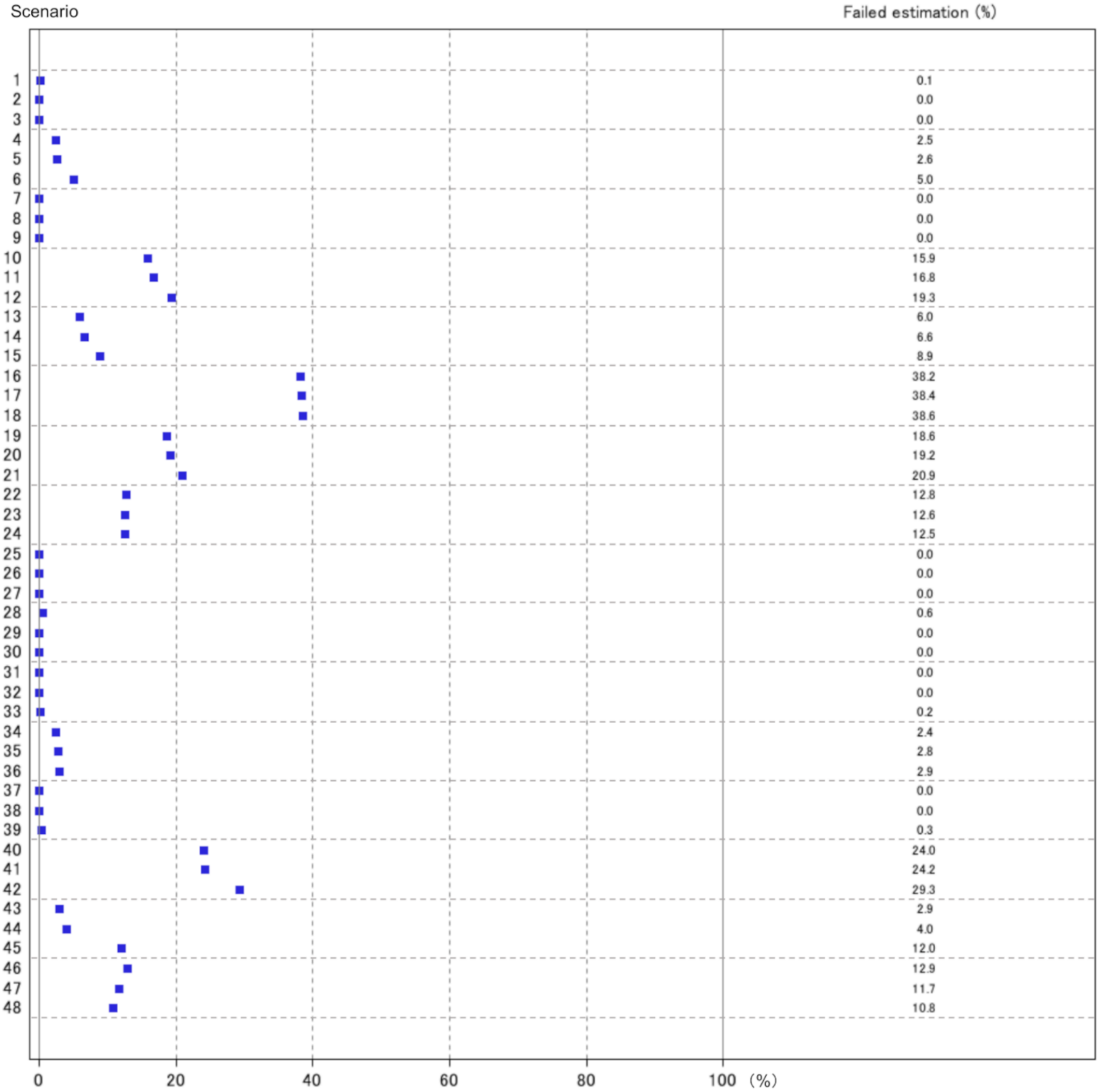
Scenario
Failed estimation (%)
1 0.1
2 0.0
3 0.0
4 2.5
5 2.6
6 5.0
7 0.0
8 0.0
9 0.0
10 15.9
11 16.8
12 19.3
13 6.0
14 6.6
15 8.9
16 38.2
17 38.4
18 38.6
19 18.6
20 19.2
21 20.9
22 12.8
23 12.6
24 12.5
25 0.0
26 0.0
27 0.0
28 0.6
29 0.0
30 0.0
31 0.0
32 0.0
33 0.2
34 2.4
35 2.8
36 2.9
37 0.0
38 0.0
39 0.3
40 24.0
41 24.2
42 29.3
43 2.9
44 4.0
45 12.0
46 12.9
47 11.7
48 10.8
0
20
40
60
80
100 (%)

(b)

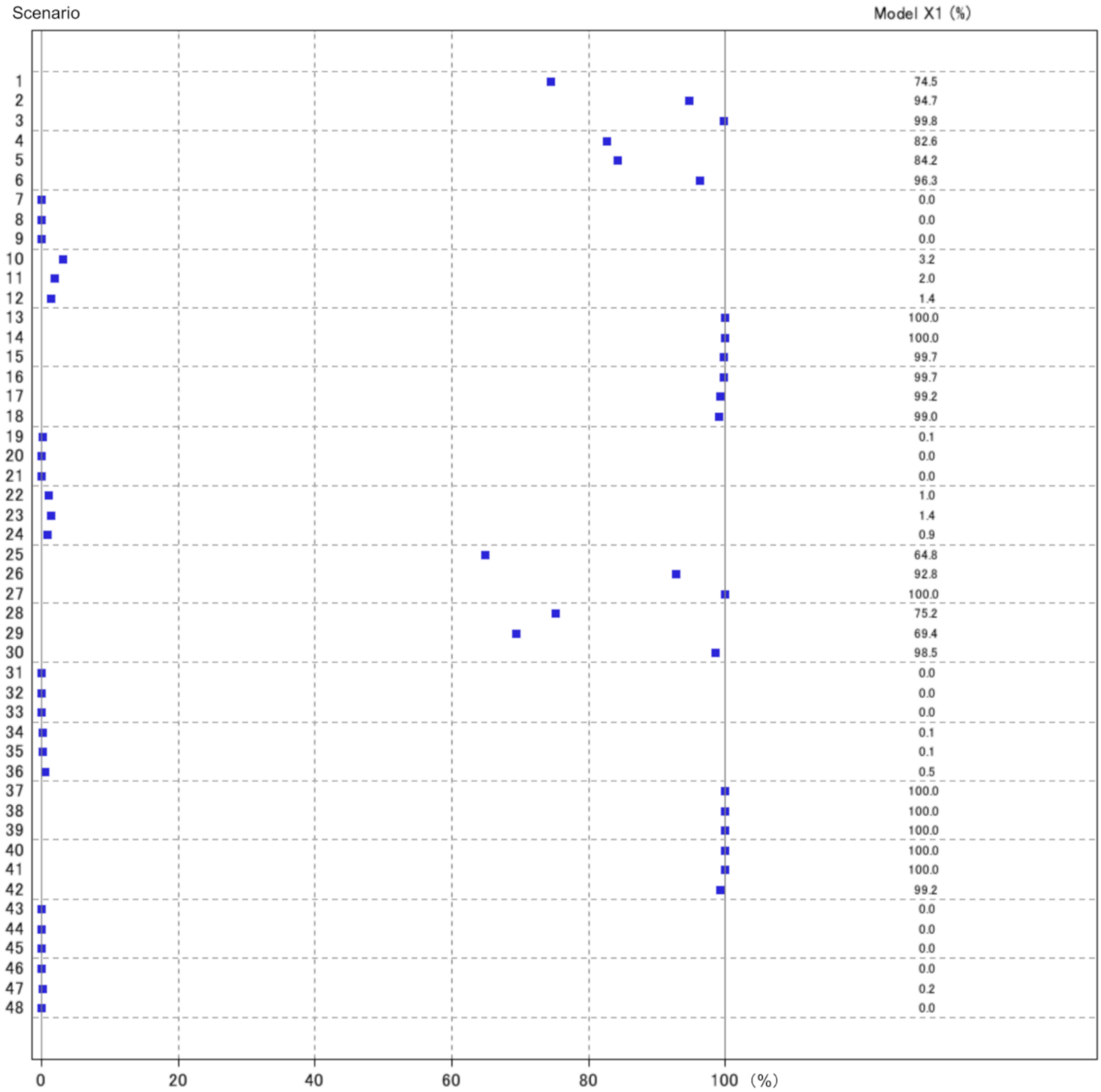
Scenario
Model X1 (%)
1
2
3
4
5
6
7
8
9
10
11
12
13
14
15
16
17
18
19
20
21
22
23
24
25
26
27
28
29
30
31
32
33
34
35
36
37
38
39
40
41
42
43
44
45
46
47
48
74.5
94.7
99.8
82.6
84.2
96.3
0.0
0.0
0.0
3.2
2.0
1.4
100.0
100.0
99.7
99.7
99.2
99.0
0.1
0.0
0.0
1.0
1.4
0.9
64.8
92.8
100.0
75.2
69.4
98.5
0.0
0.0
0.0
0.1
0.1
0.5
100.0
100.0
100.0
100.0
100.0
99.2
0.0
0.0
0.0
0.0
0.2
0.0
0
20
40
60
80
100 (%)

(c)

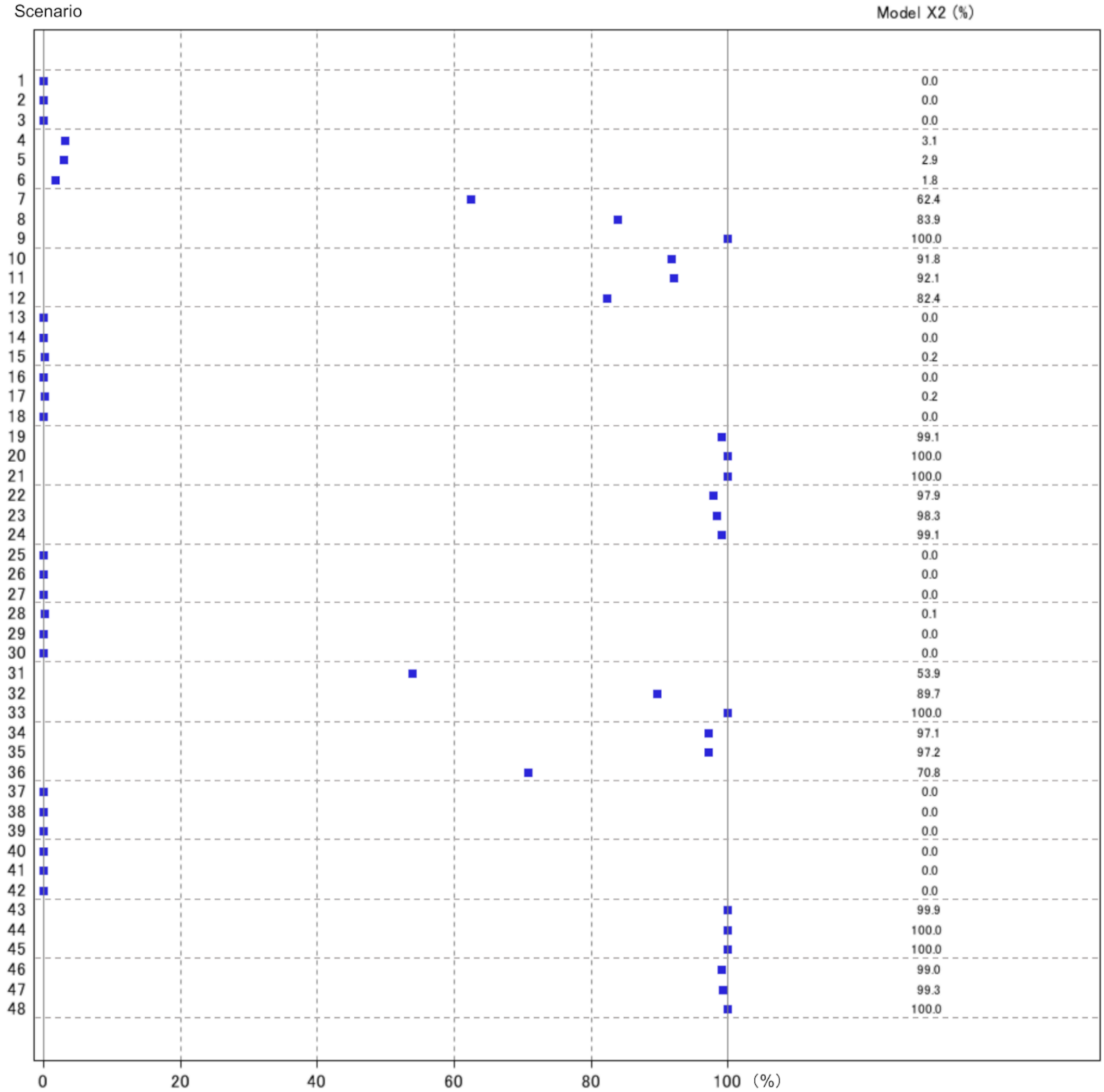

Scenario
Model X2 (%)
1 0.0
2 0.0
3 0.0
4 3.1
5 2.9
6 1.8
7 62.4
8 83.9
9 100.0
10 91.8
11 92.1
12 82.4
13 0.0
14 0.0
15 0.2
16 0.0
17 0.2
18 0.0
19 99.1
20 100.0
21 100.0
22 97.9
23 98.3
24 99.1
25 0.0
26 0.0
27 0.0
28 0.1
29 0.0
30 0.0
31 53.9
32 89.7
33 100.0
34 97.1
35 97.2
36 70.8
37 0.0
38 0.0
39 0.0
40 0.0
41 0.0
42 0.0
43 99.9
44 100.0
45 100.0
46 99.0
47 99.3
48 100.0
0
20
40
60
80
100 (%)

(d)

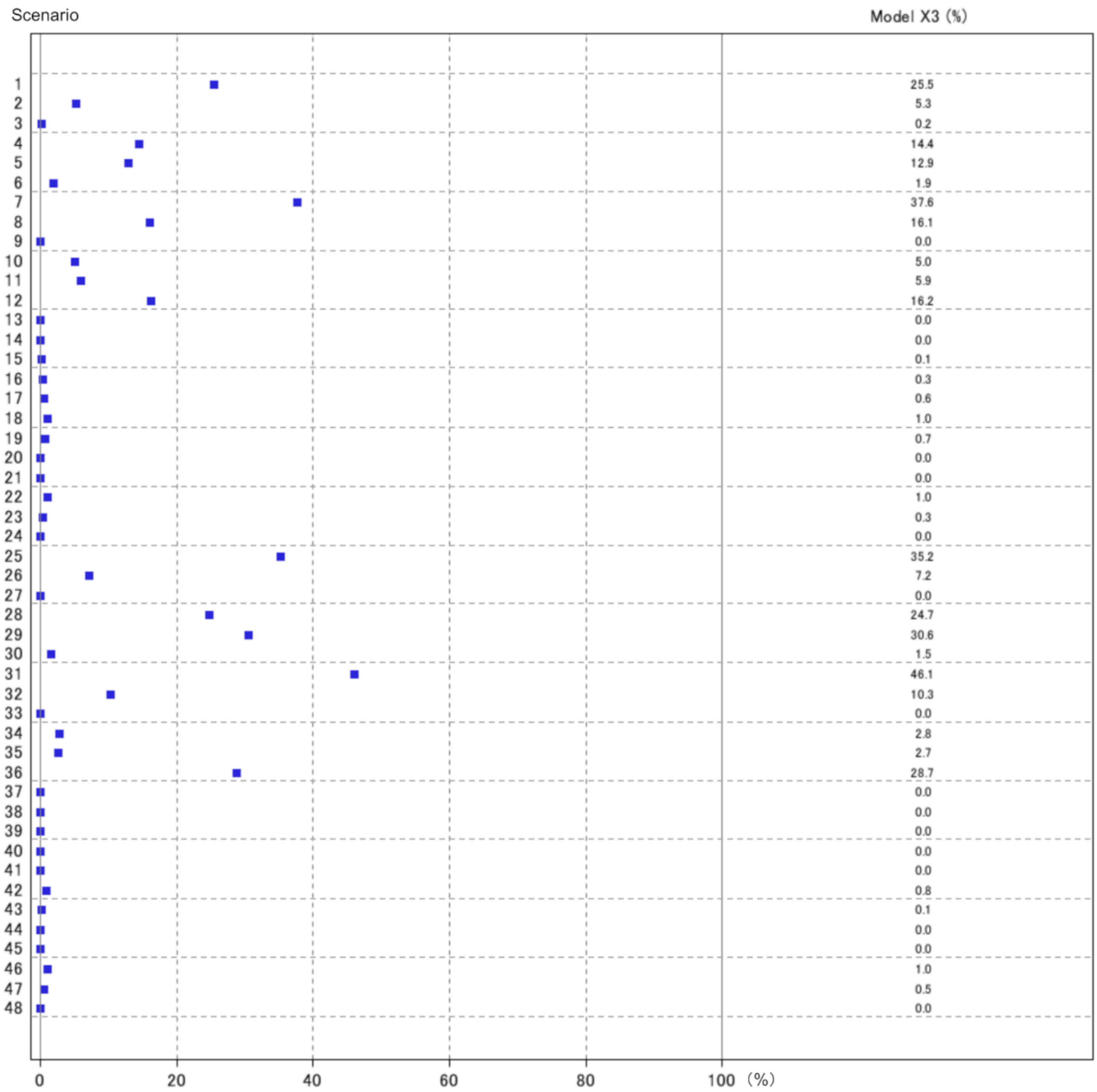
Scenario
Model X3 (%)
1 25.5
2 5.3
3 0.2
4 14.4
5 12.9
6 1.9
7 37.6
8 16.1
9 0.0
10 5.0
11 5.9
12 16.2
13 0.0
14 0.0
15 0.1
16 0.3
17 0.6
18 1.0
19 0.7
20 0.0
21 0.0
22 1.0
23 0.3
24 0.0
25 35.2
26 7.2
27 0.0
28 24.7
29 30.6
30 1.5
31 46.1
32 10.3
33 0.0
34 2.8
35 2.7
36 28.7
37 0.0
38 0.0
39 0.0
40 0.0
41 0.0
42 0.8
43 0.1
44 0.0
45 0.0
46 1.0
47 0.5
48 0.0
0 20 40 60 80 100 (%)

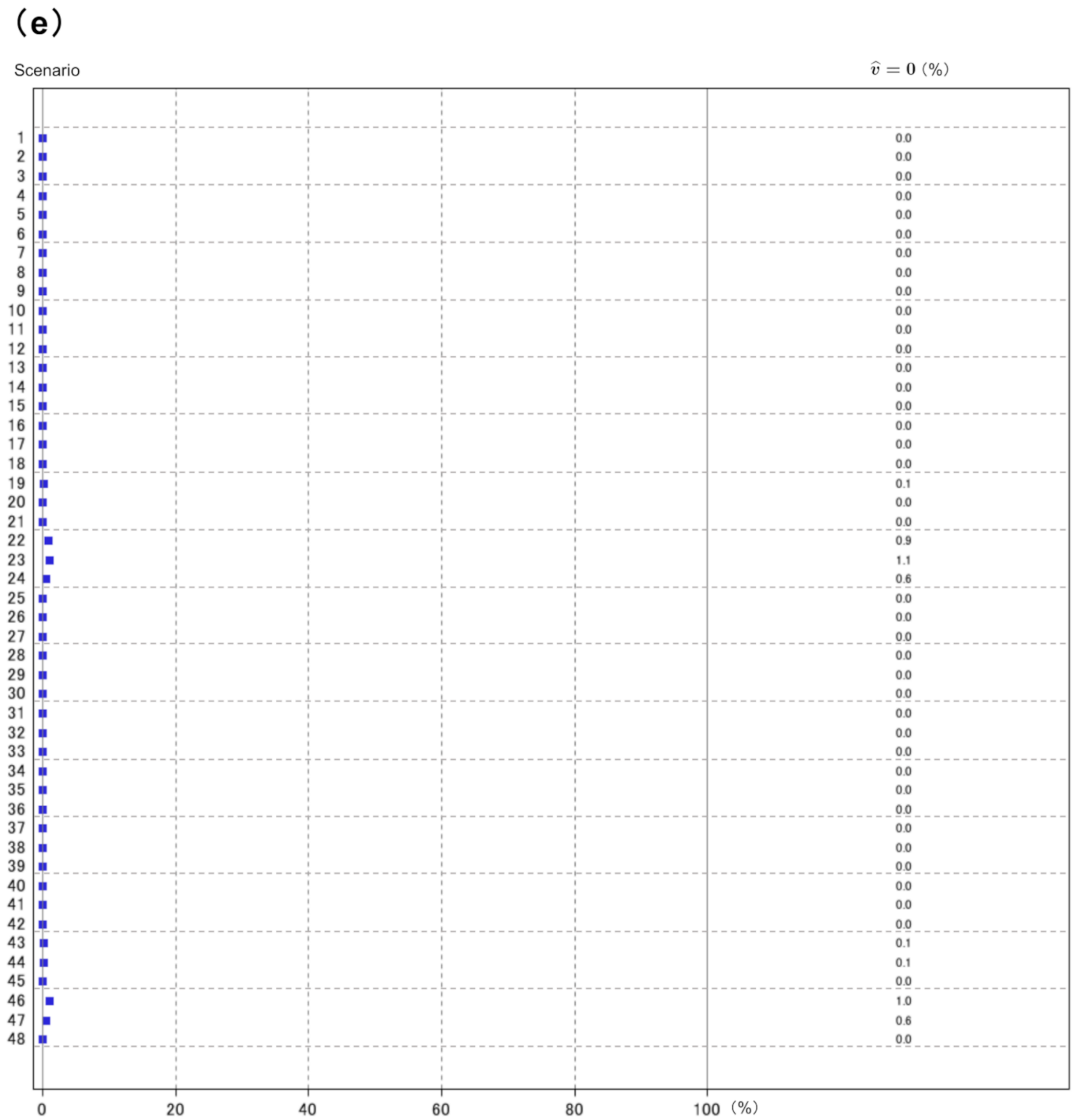

Fig. S5 Simulation results for proportion of (**a**) failed estimation, (**b**) Model X1, (**c**) Model X2, (**d**) Model X3, and (**e**) $\hat{v} = 0$. The iterations of 1,000 for each scenario were classified as either successful or failed. Failed estimation means that the SAS NLMIXED procedure in Equations (4.1) and (4.2) failed to produce estimates. The estimable iterations were subsequently categorised as Models X1, X2, and X3.

Supplementary Table

Table S1 Observed and expected Jaccard indices for aberrant and normal protein–protein interactions (PPIs) across detection thresholds.

Detection threshold	ccRCC aberrant		ccRCC normal		LUSC aberrant		LUSC normal	
	Observed	Expected	Observed	Expected	Observed	Expected	Observed	Expected
1	45.7%	37.6%	34.2%	25.3%	23.4%	23.2%	25.0%	24.8%
2	44.2%	34.8%	40.0%	30.3%	18.4%	20.4%	40.4%	42.1%
3	33.3%	28.2%	43.5%	38.8%	14.7%	18.5%	46.3%	49.3%
4	33.3%	25.0%	48.9%	41.7%	12.9%	17.0%	50.9%	53.9%
5	23.5%	19.5%	49.0%	45.8%	13.3%	16.6%	52.7%	55.1%
6	20.6%	18.5%	48.1%	46.4%	13.8%	16.1%	54.5%	56.2%
7	20.6%	18.5%	48.1%	46.4%	13.8%	16.1%	54.5%	56.2%
8	20.6%	18.5%	48.1%	46.4%	14.3%	15.6%	56.4%	57.3%

Note: The table summarises the results for clear cell renal cell carcinoma (ccRCC) and lung squamous cell carcinoma (LUSC). The observed Jaccard index was calculated as the proportion of PPIs on which both methods agreed among those labelled by either method. The expected value was computed by assuming independent labelling.

Supplementary References

[1] Kanehisa M, Goto S. KEGG: Kyoto encyclopedia of genes and genomes. Nucleic Acids Res. 2000;28:27–30. https://doi.org/10.1093/nar/28.1.27.

[2] Amadoz A, Hidalgo MR, Çubuk C, Carbonell-Caballero J, Dopazo J. A comparison of mechanistic signaling pathway activity analysis methods. Brief Bioinform. 2019;20:1655–68. https://doi.org/10.1093/bib/bby040.

[3] Robinson MD, Oshlack A. A scaling normalization method for differential expression analysis of RNA-seq data. Genome Biol. 2010;11:R25. https://doi.org/10.1186/gb-2010-11-3-r25.

[4] Wagner GP, Kin K, Lynch VJ. Measurement of mRNA abundance using RNA-seq data: RPKM measure is inconsistent among samples. Theory Biosci. 2012;131:281–5. https://doi.org/10.1007/s12064-012-0162-3.

[5] Halder S, Ghosh S, Chattopadhyay J, Chatterjee S. Bistability in cell signalling and its significance in identifying potential drug-targets. Bioinformatics. 2021;37:4156–63. https://doi.org/10.1093/bioinformatics/btab395.

[6] Hunter SA, Cochran JR. Cell-binding assays for determining the affinity of protein–protein interactions: technologies and considerations. Methods Enzymol. 2016;580:21–44. https://doi.org/10.1016/bs.mie.2016.05.002.
