# Supplementary material for: Model-based quantification of protein–protein interaction aberrations for exploring dysregulated signalling pathways through pathway maps and gene expression levels
Source: BMC Bioinformatics. 2026 Jul 8;27:263. doi: 10.1186/s12859-026-06543-8 (PMC13732913; doi:10.1186/s12859-026-06543-8)
Supplement: Supplementary file 2 [file 12859_2026_6543_MOESM2_ESM.pdf]

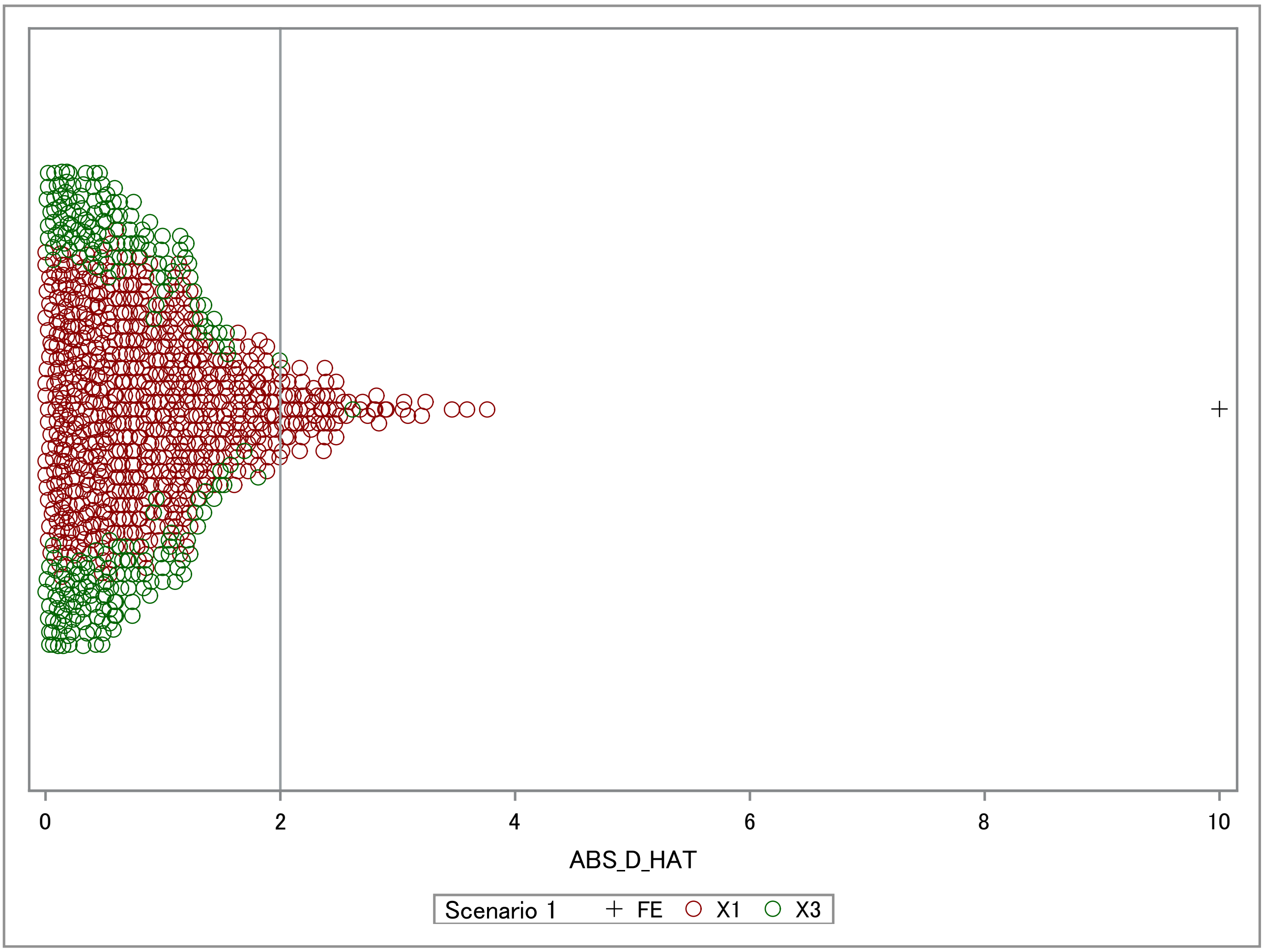
0
2
4
6
8
10
ABS_D_HAT
Scenario 1
FE
X1
X3

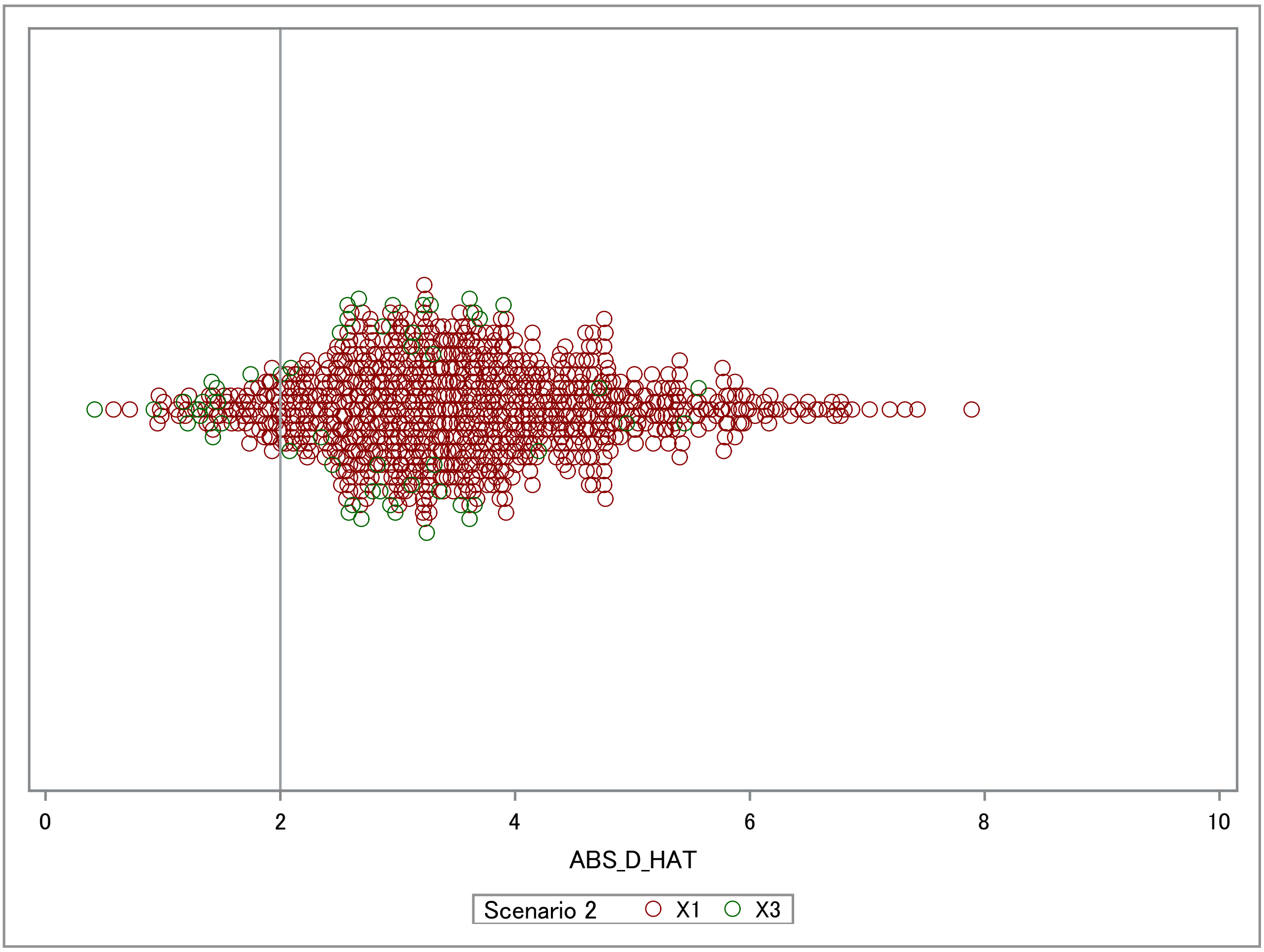
0
2
4
6
8
10
ABS_D_HAT
Scenario 2
X1
X3

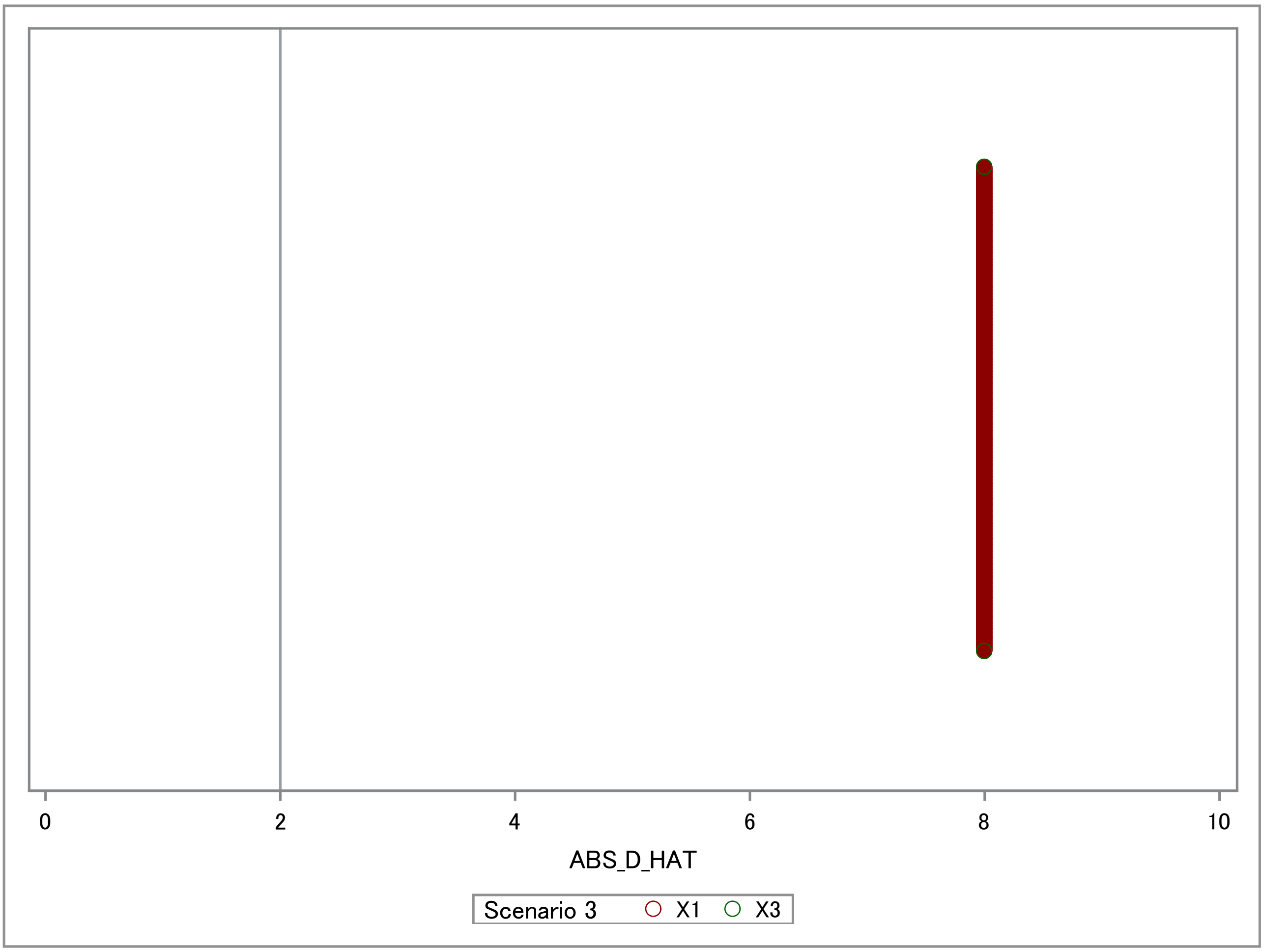
0
2
4
6
8
10
ABS_D_HAT
Scenario 3
X1
X3

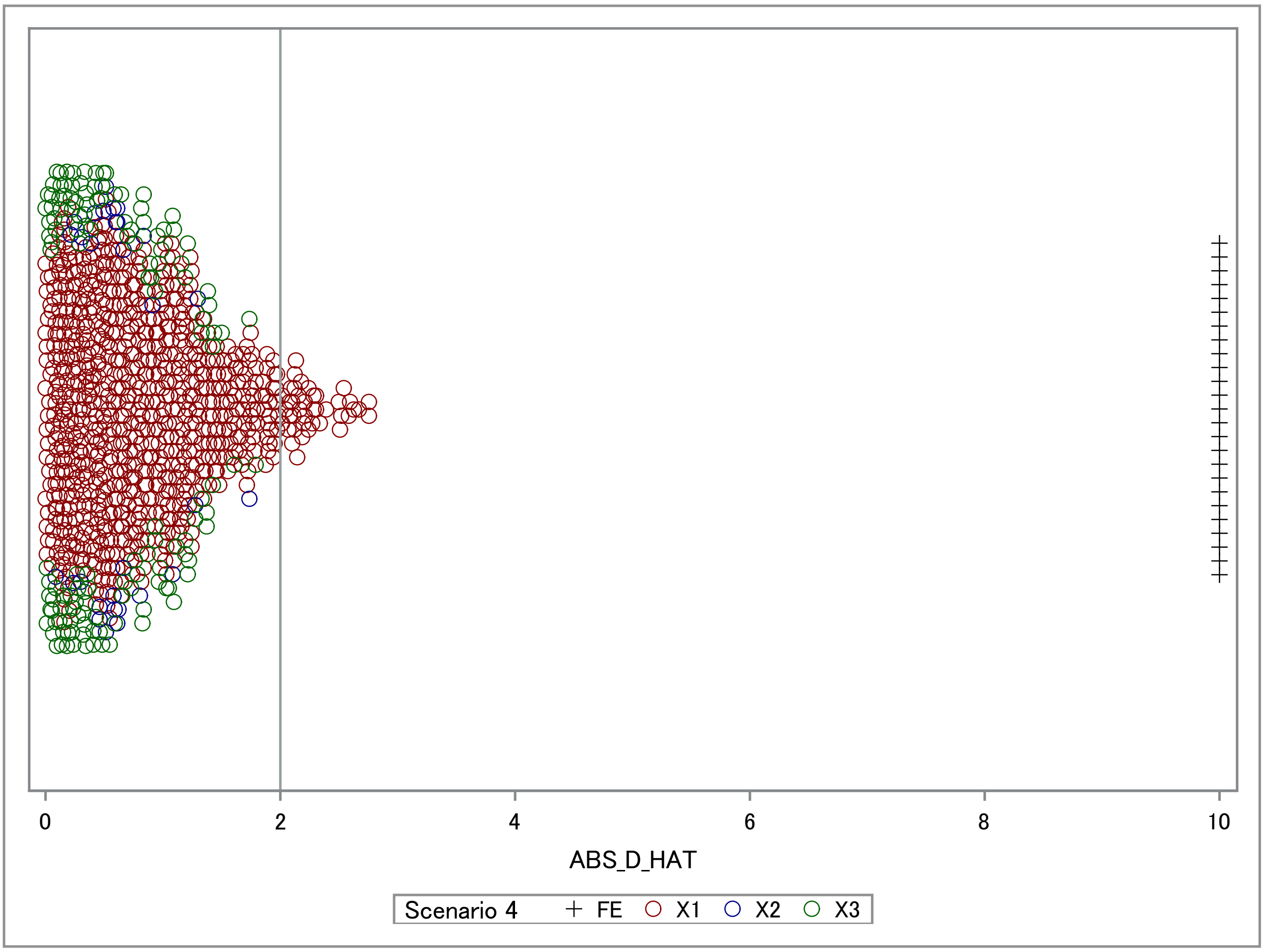
0
2
4
6
8
10
ABS_D_HAT
Scenario 4
FE
X1
X2
X3

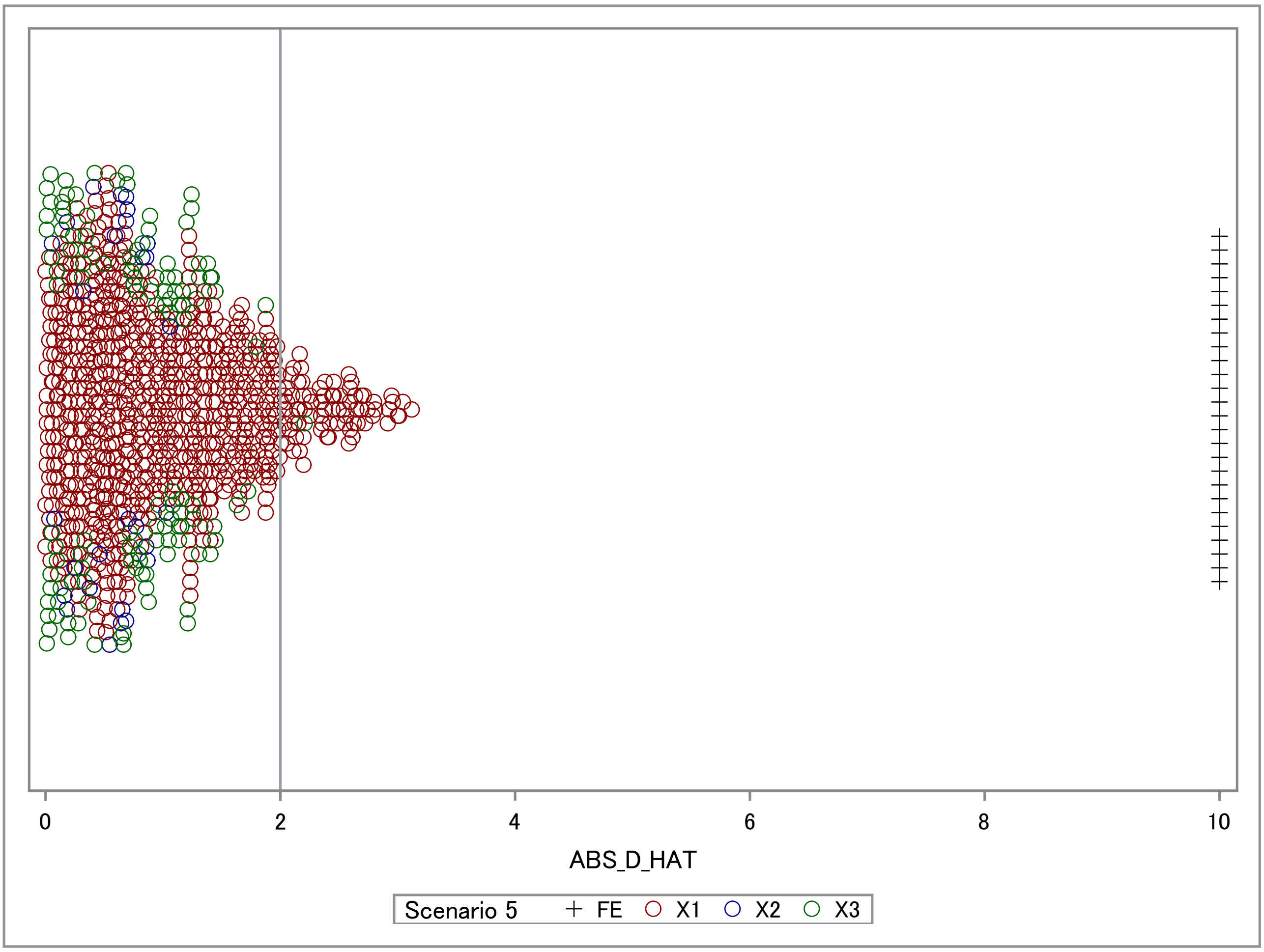
0
2
4
6
8
10
ABS_D_HAT
Scenario 5
FE
X1
X2
X3

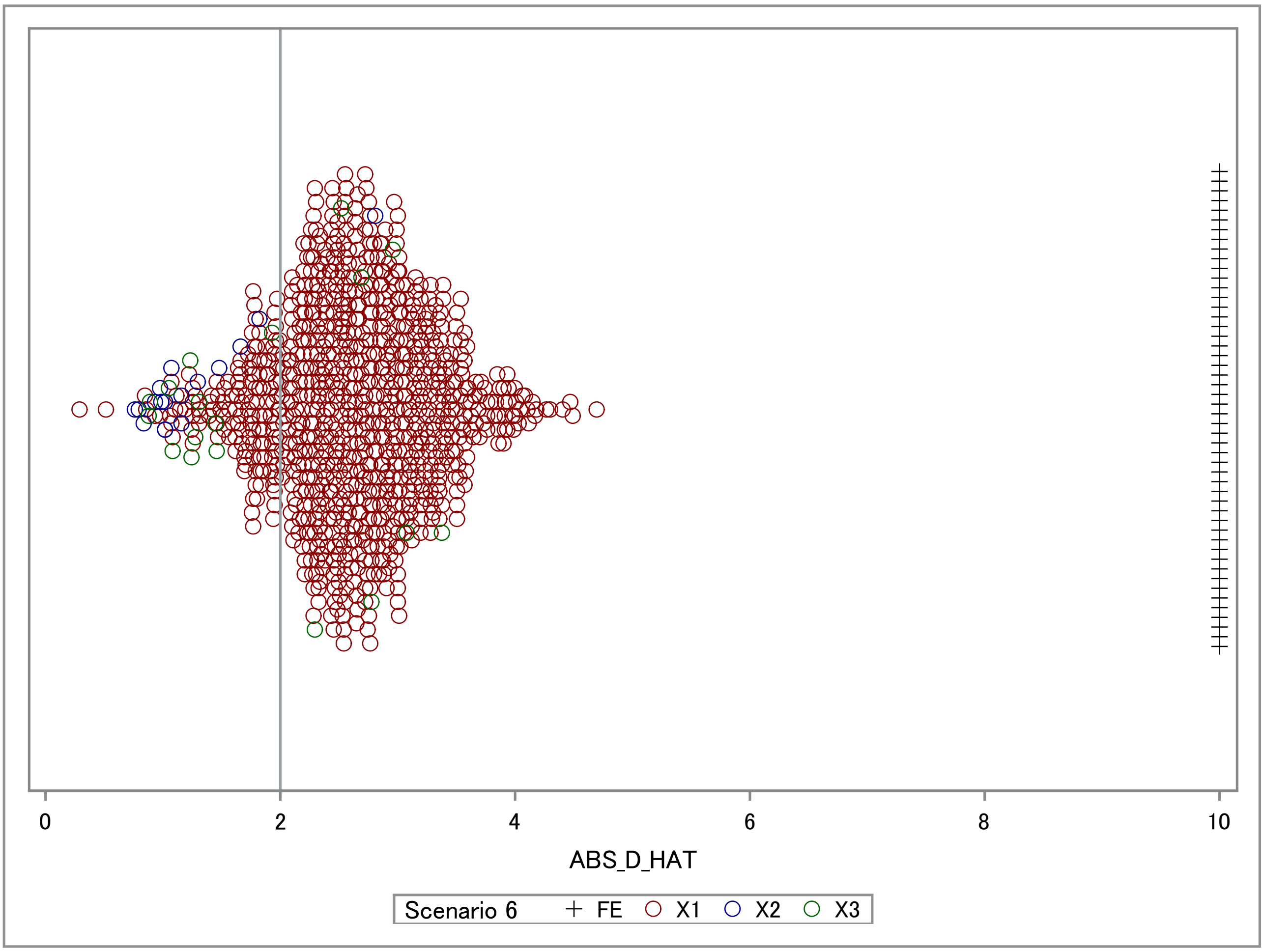
0
2
4
6
8
10
ABS_D_HAT
Scenario 6
FE
X1
X2
X3

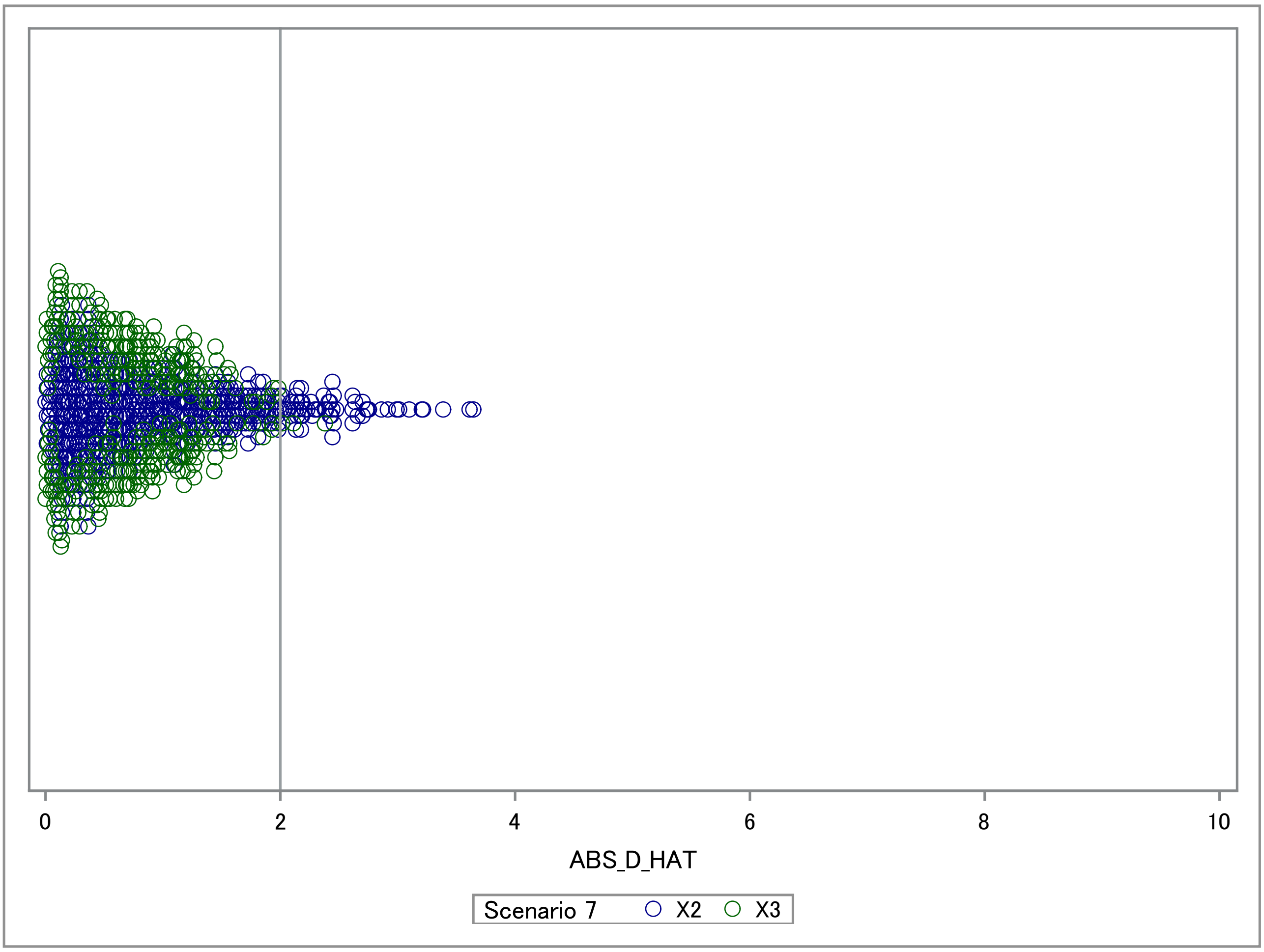
0
2
4
6
8
10
ABS_D_HAT
Scenario 7
X2
X3

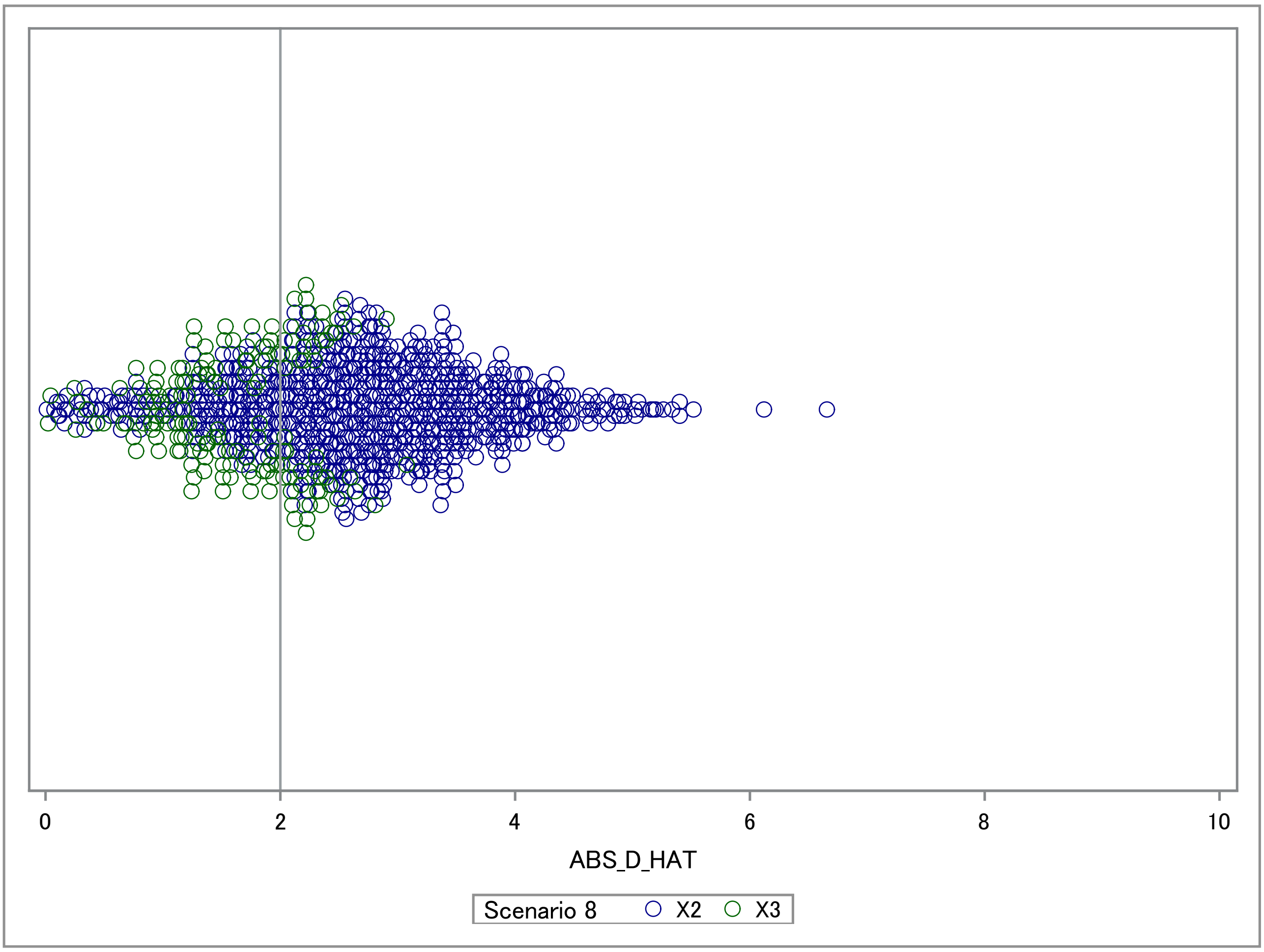
0
2
4
6
8
10
ABS_D_HAT
Scenario 8
X2
X3

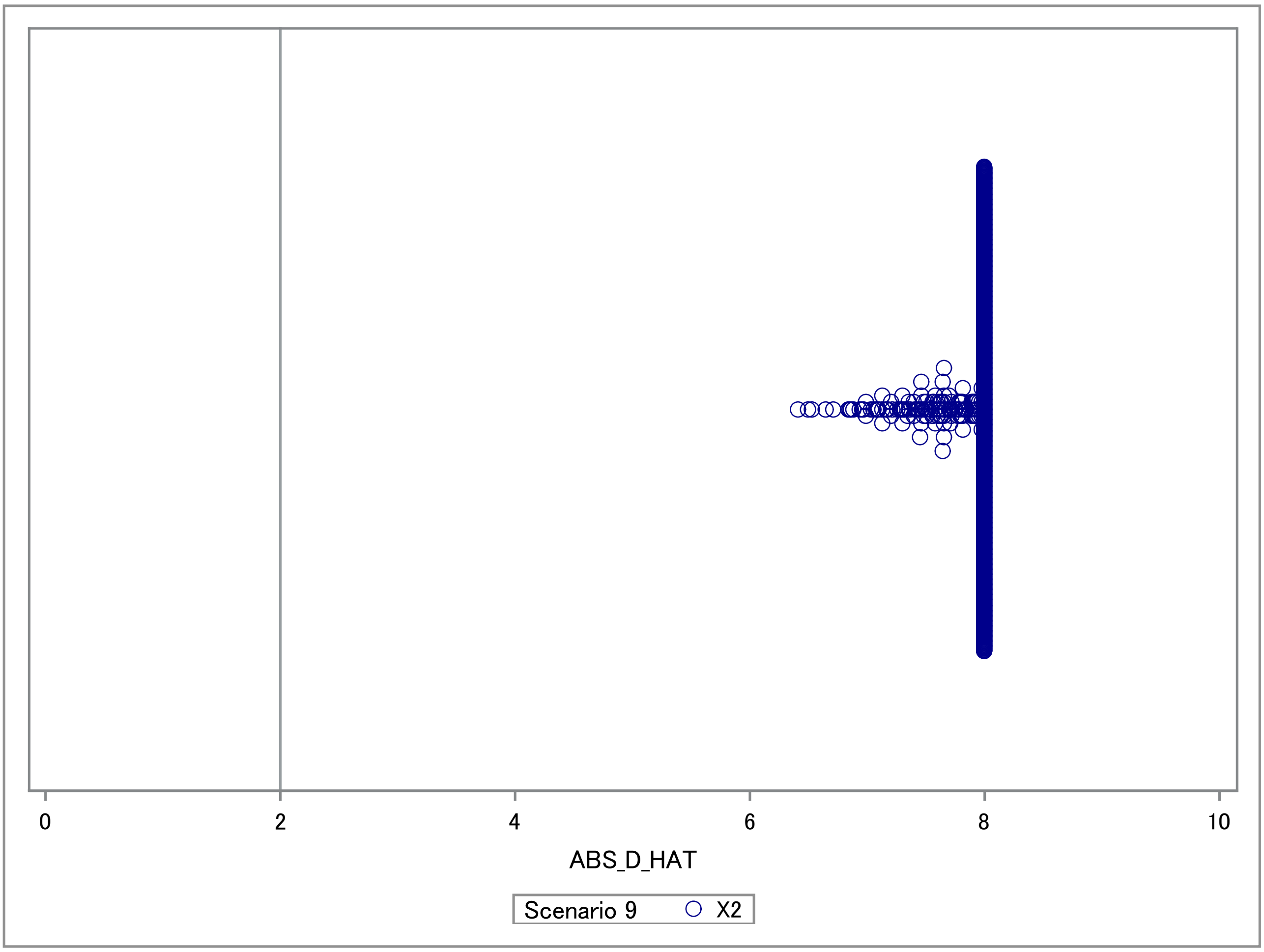
0
2
4
6
8
10
ABS_D_HAT
Scenario 9
X2

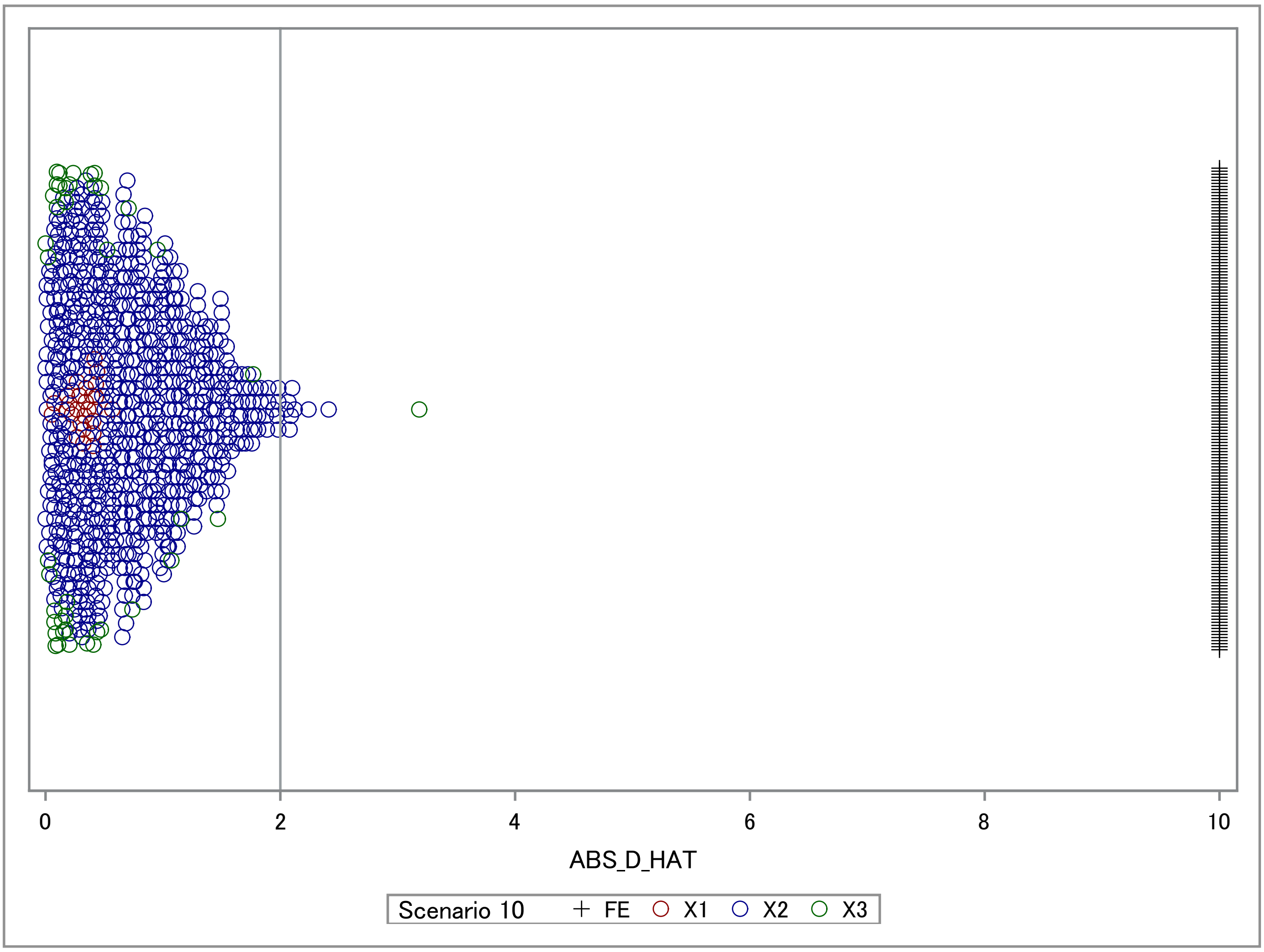
0
2
4
6
8
10
ABS_D_HAT
Scenario 10
FE
X1
X2
X3

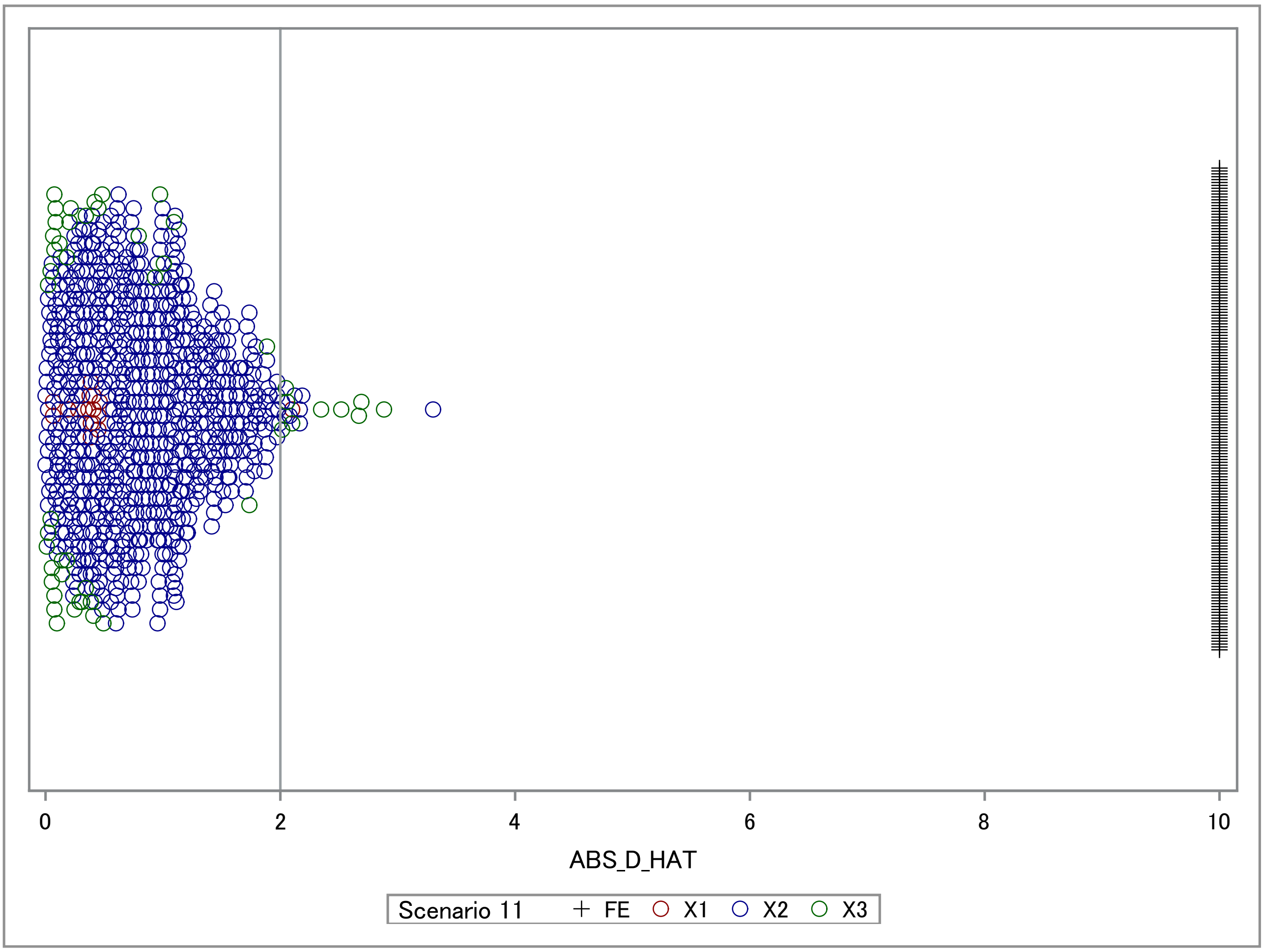
0
2
4
6
8
10
ABS_D_HAT
Scenario 11
FE
X1
X2
X3

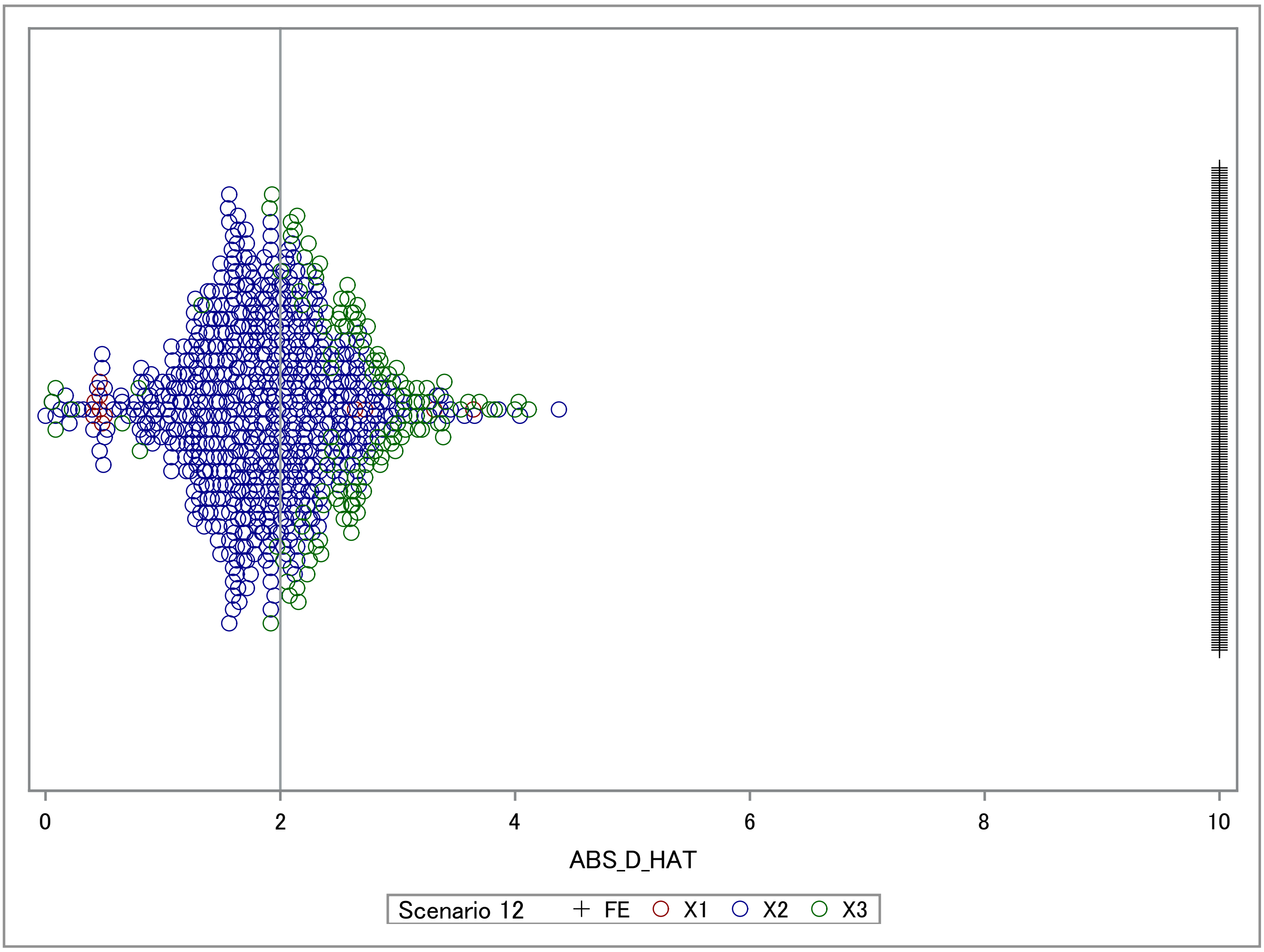
0
2
4
6
8
10
ABS_D_HAT
Scenario 12
FE
X1
X2
X3

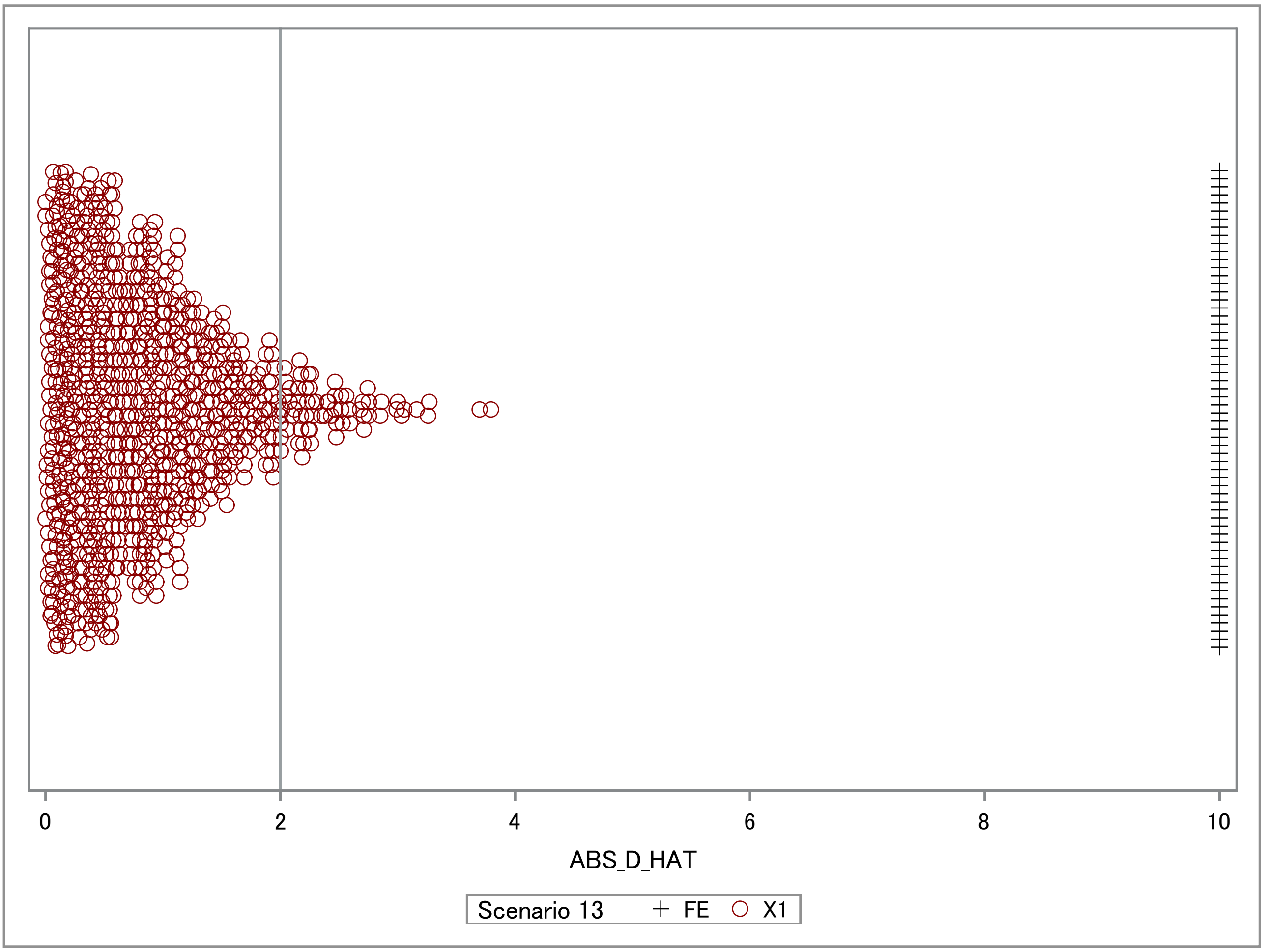
0
2
4
6
8
10
ABS_D_HAT
Scenario 13
FE
X1

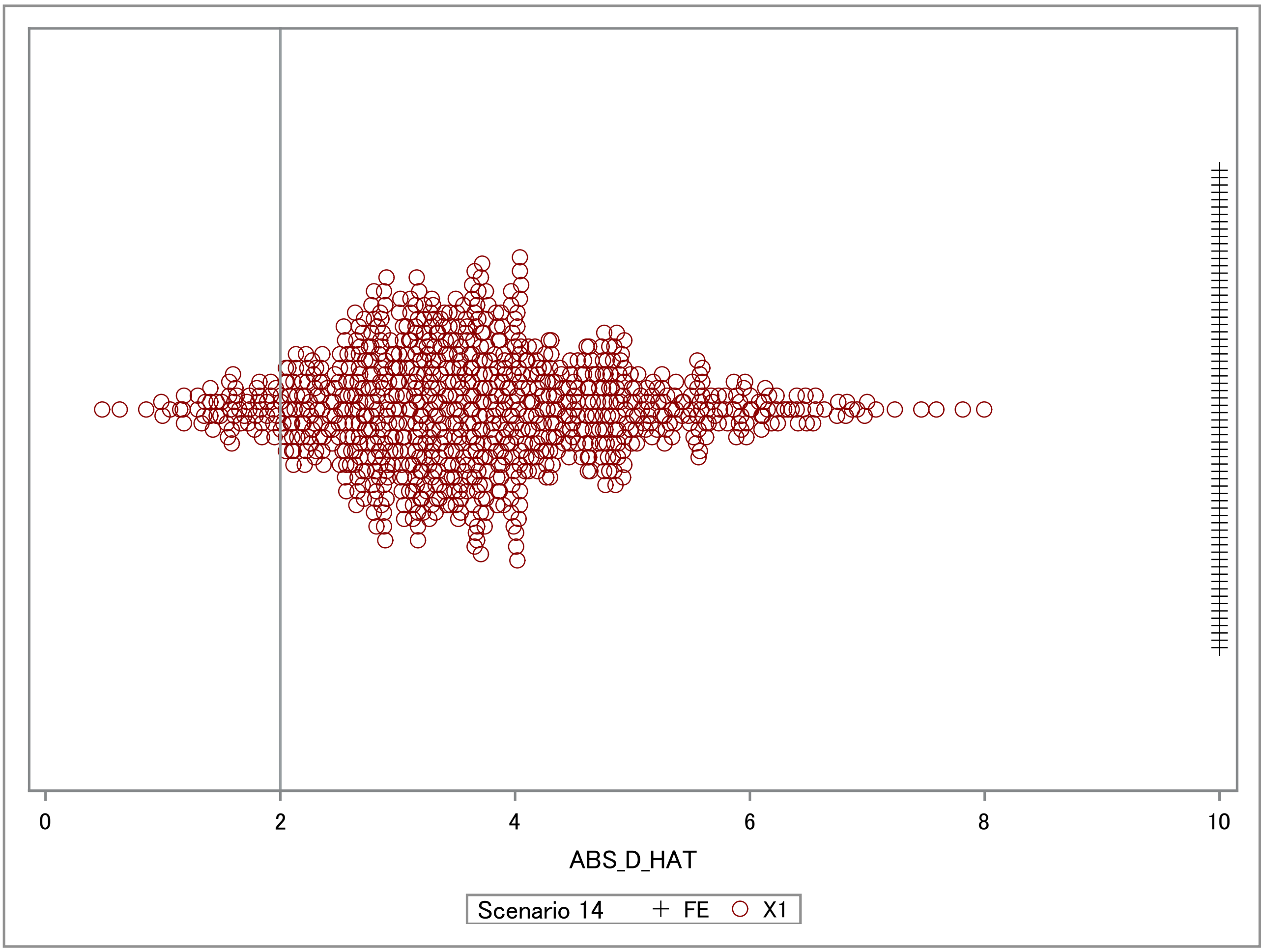
0
2
4
6
8
10
ABS_D_HAT
Scenario 14
FE
X1

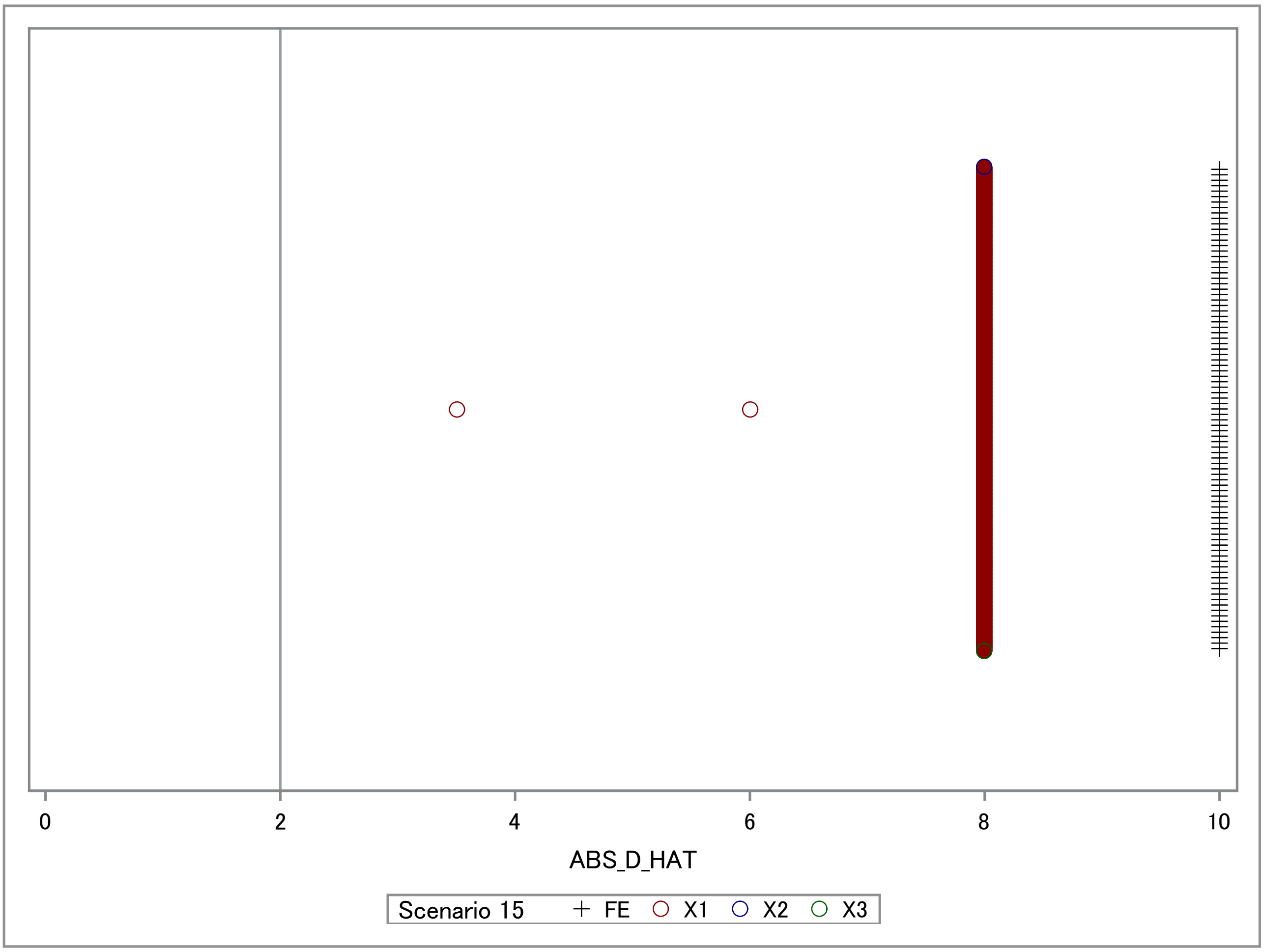
0
2
4
6
8
10
ABS_D_HAT
Scenario 15
FE
X1
X2
X3

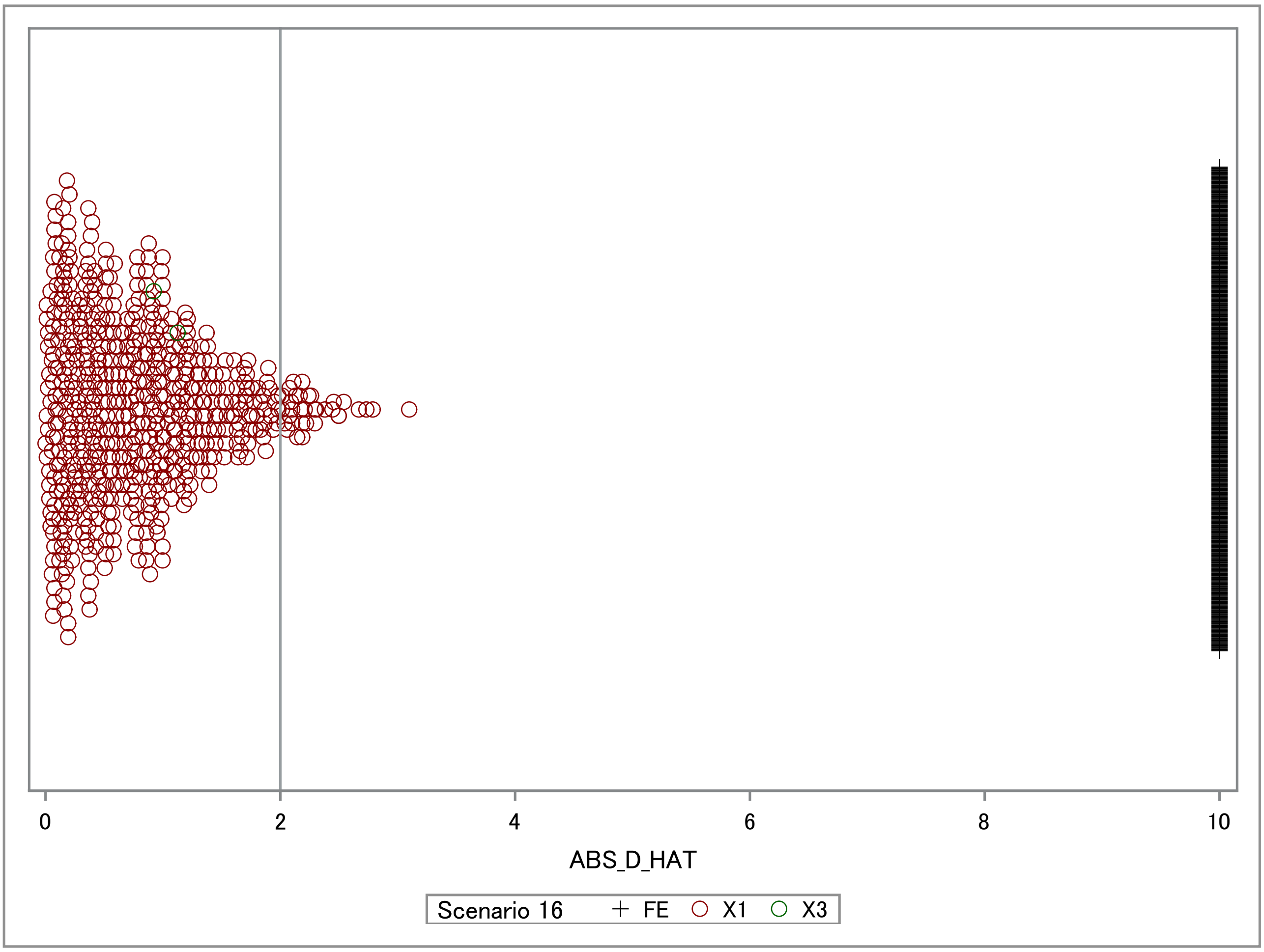
0
2
4
6
8
10
ABS_D_HAT
Scenario 16
FE
X1
X3

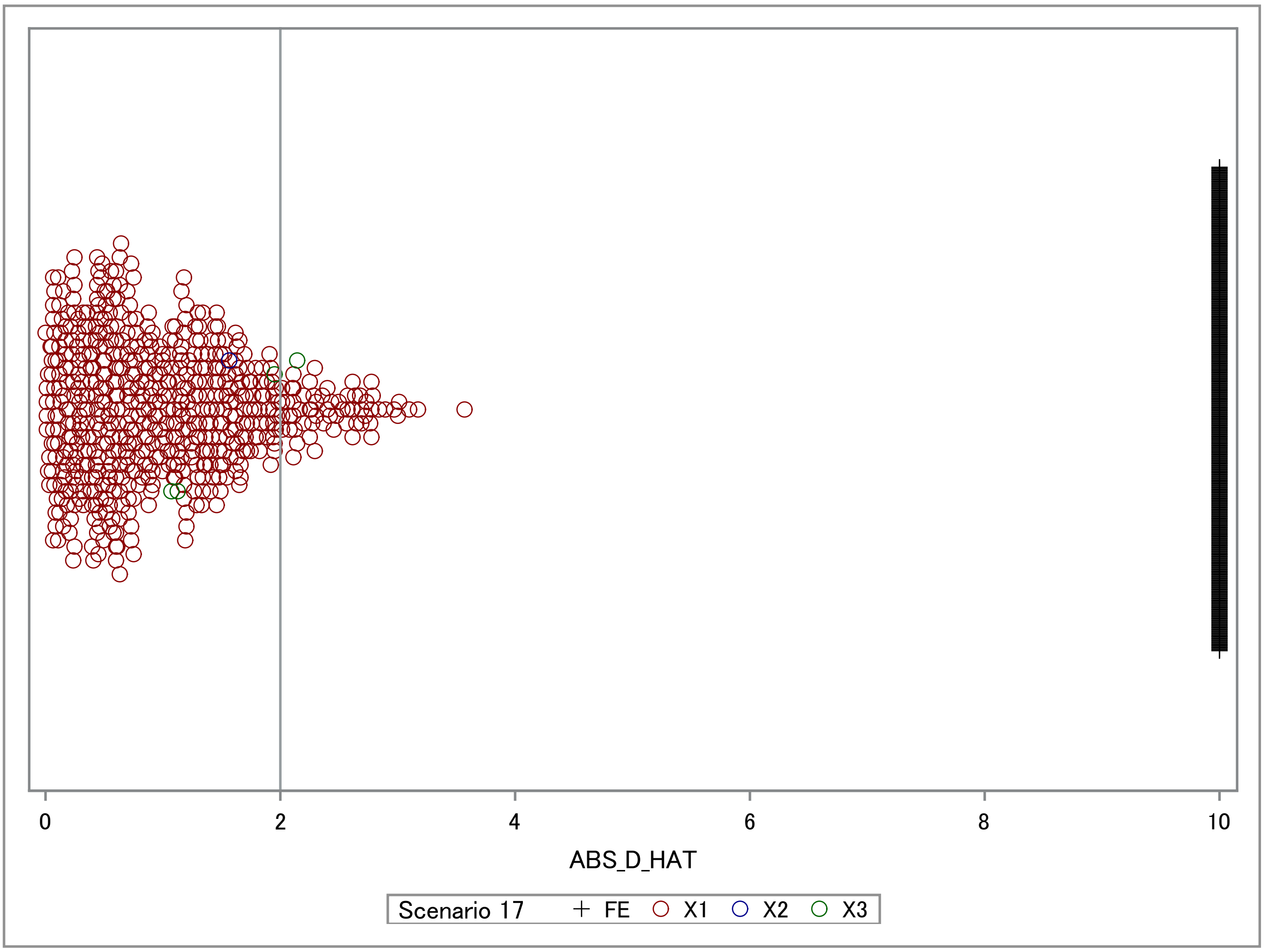
0
2
4
6
8
10
ABS_D_HAT
Scenario 17
FE
X1
X2
X3

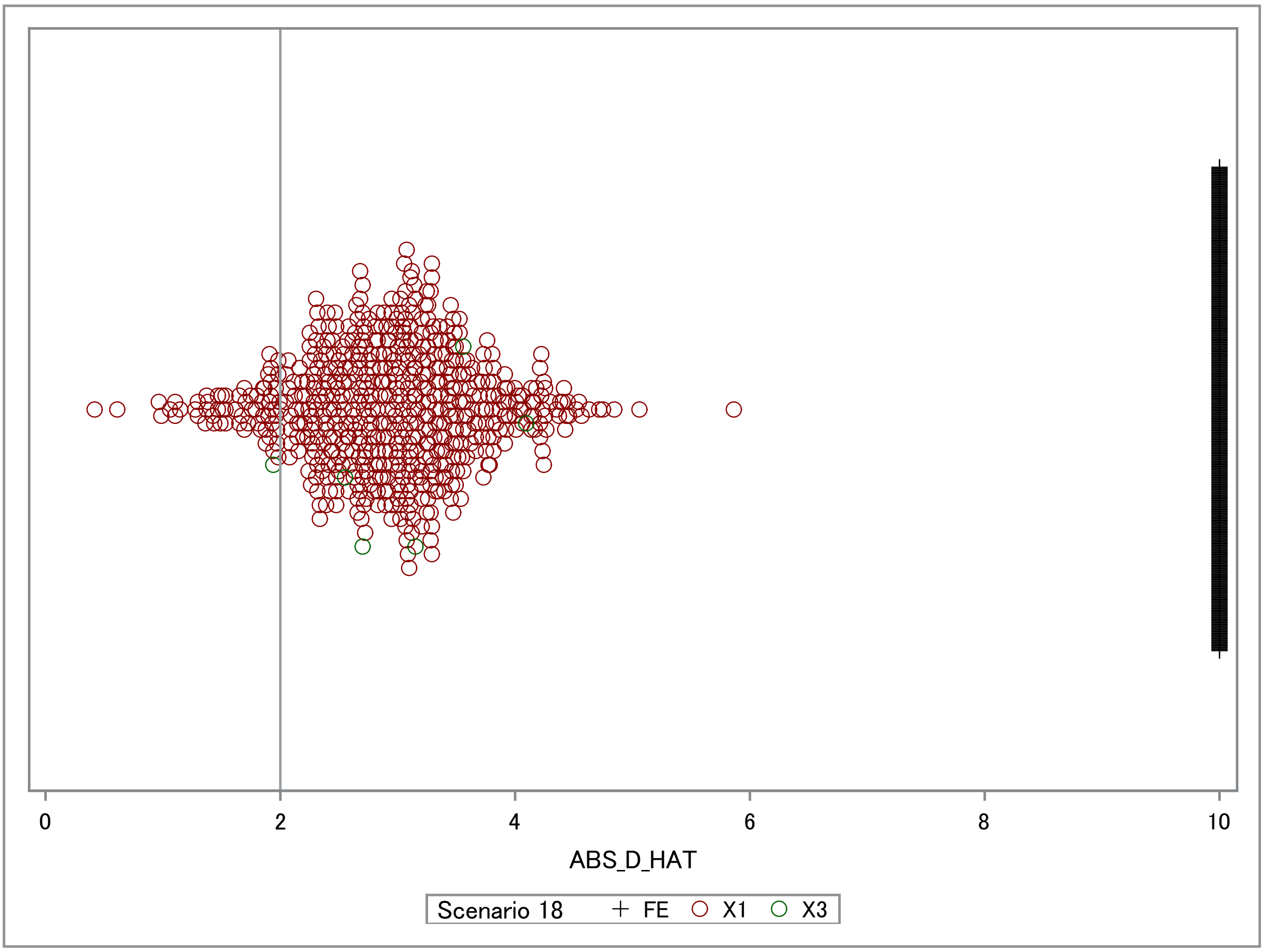

0
2
4
6
8
10
ABS_D_HAT
Scenario 18
FE
X1
X3

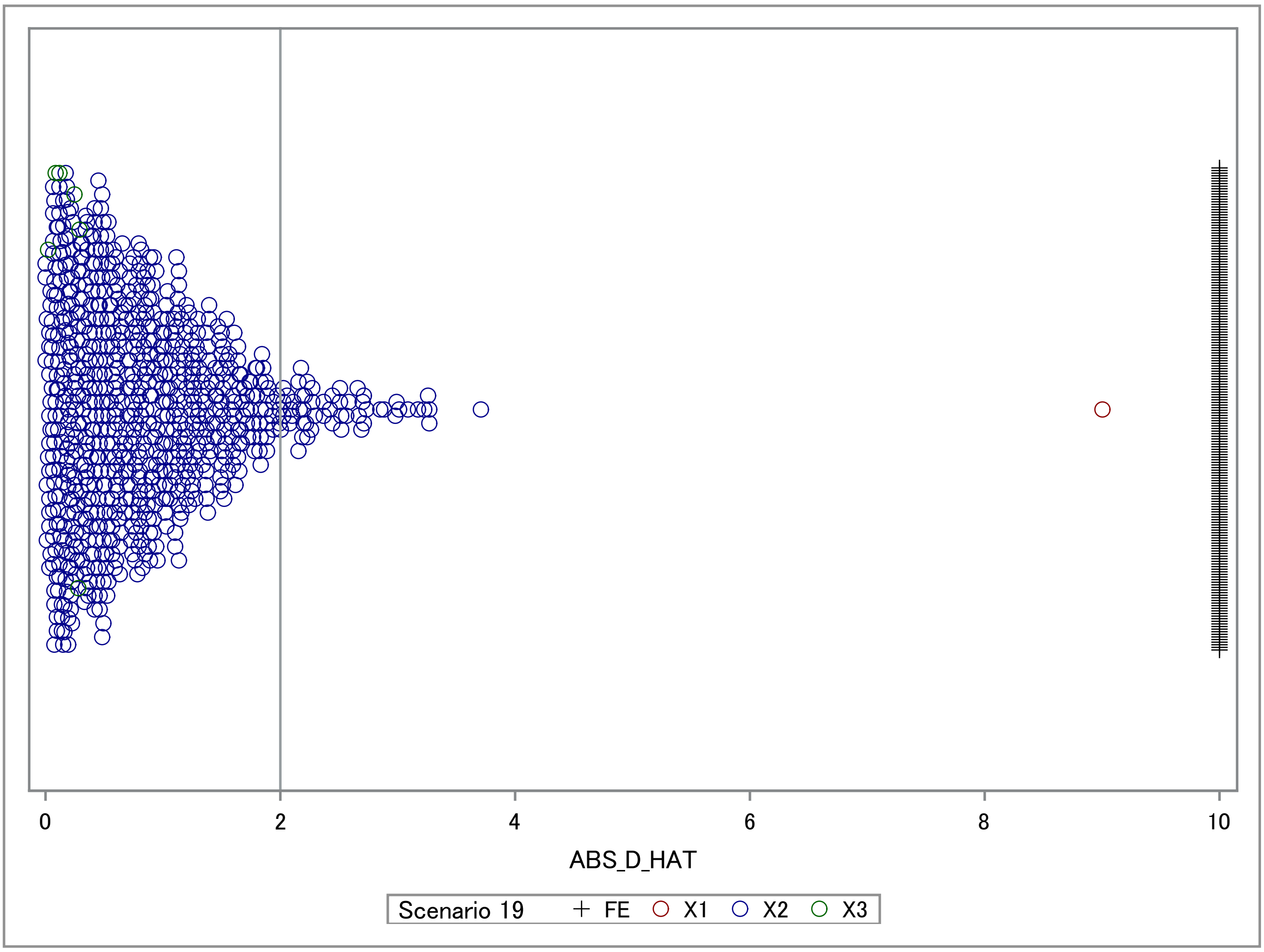
0
2
4
6
8
10
ABS_D_HAT
Scenario 19
FE
X1
X2
X3

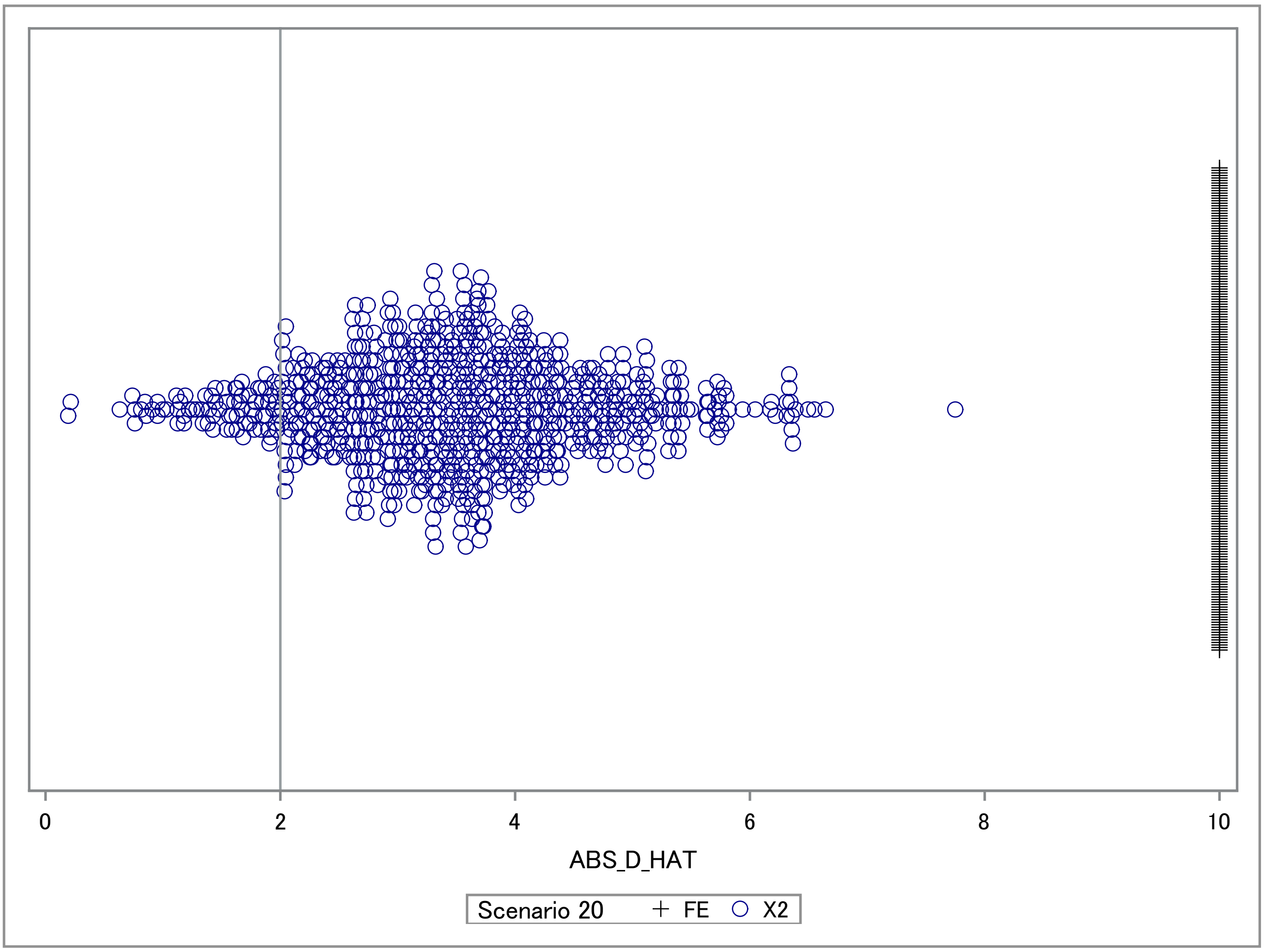
0
2
4
6
8
10
ABS_D_HAT
Scenario 20
FE
X2

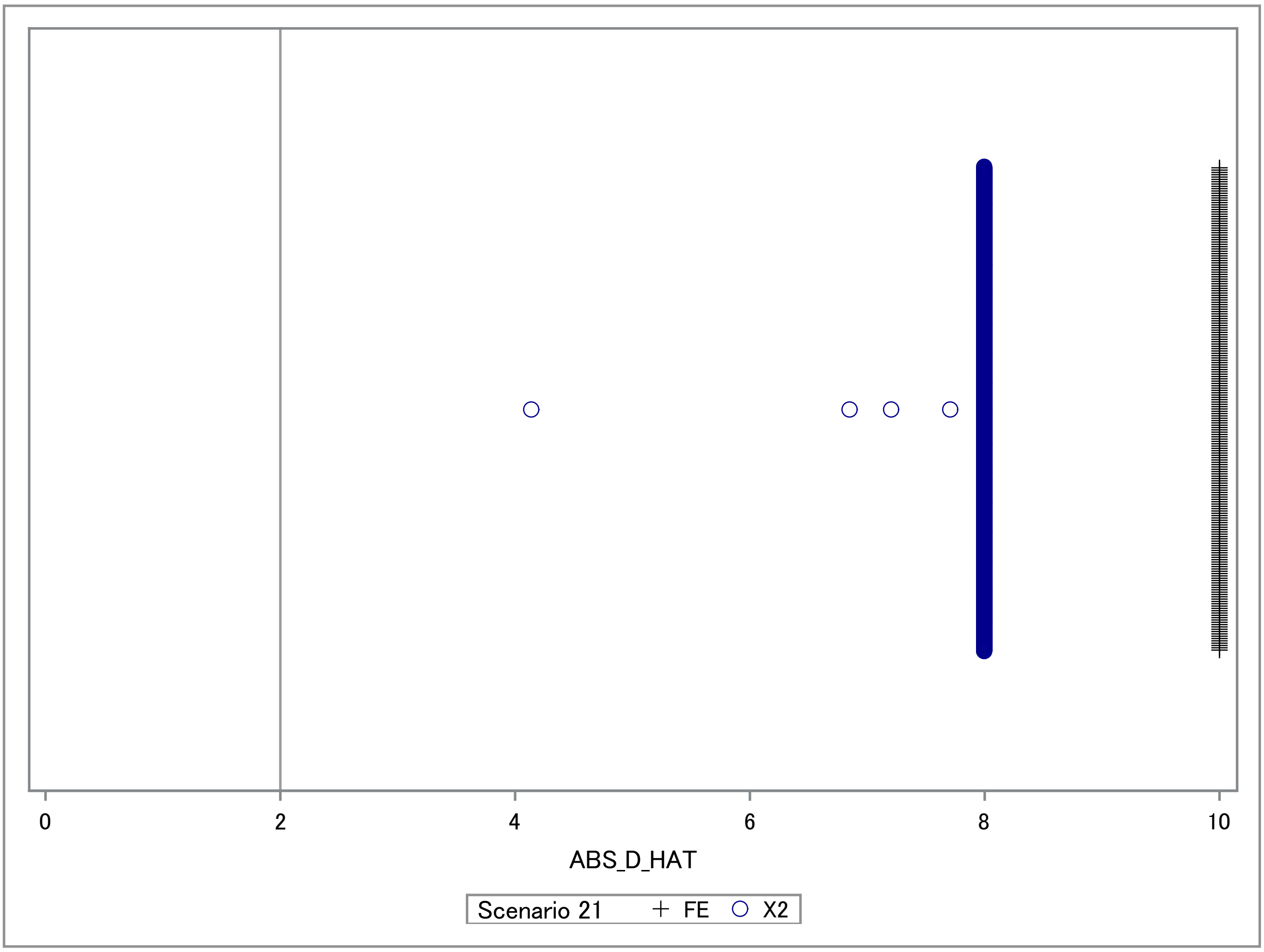
0
2
4
6
8
10
ABS_D_HAT
Scenario 21
FE
X2

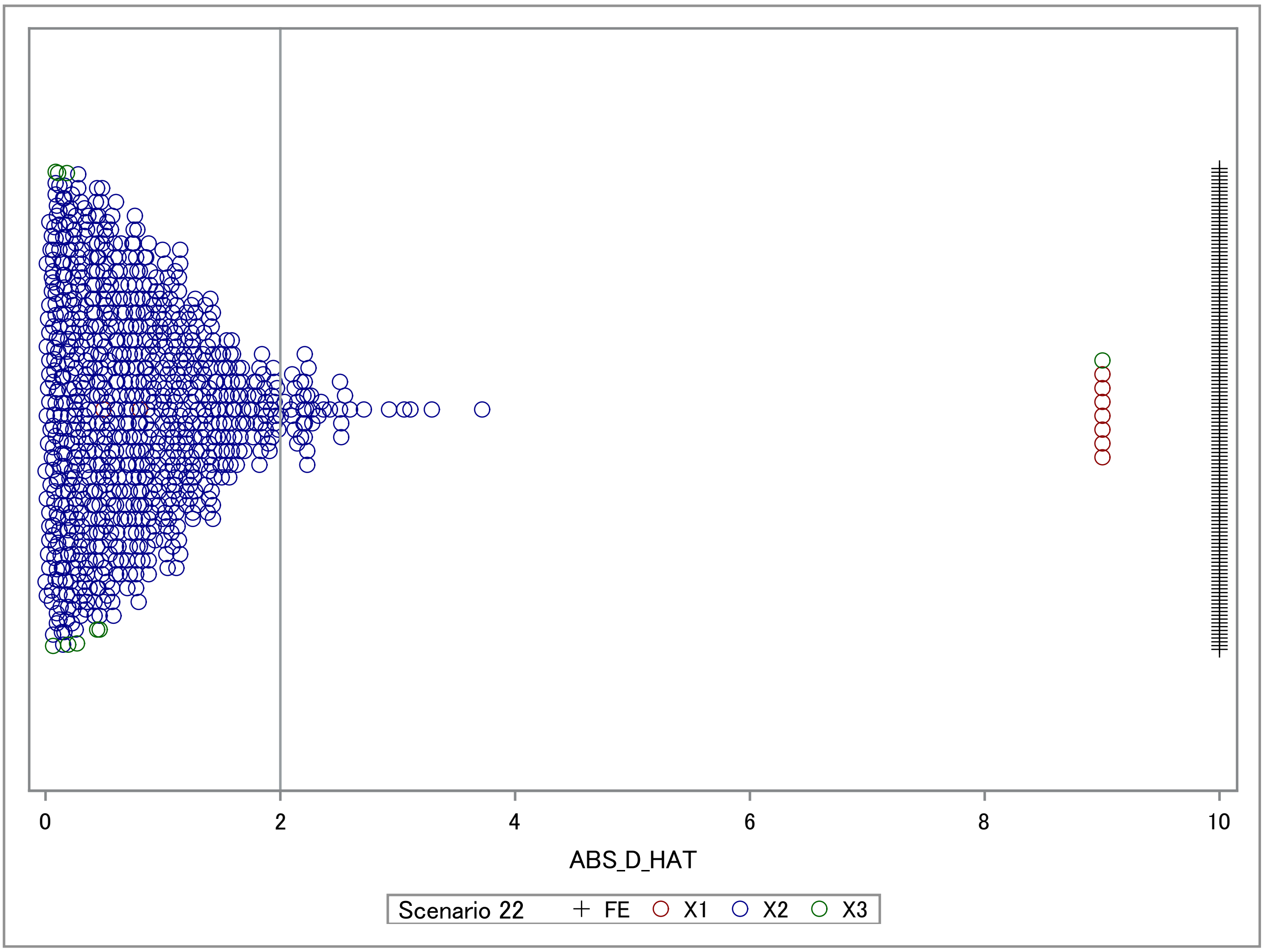
0
2
4
6
8
10
ABS_D_HAT
Scenario 22
FE
X1
X2
X3

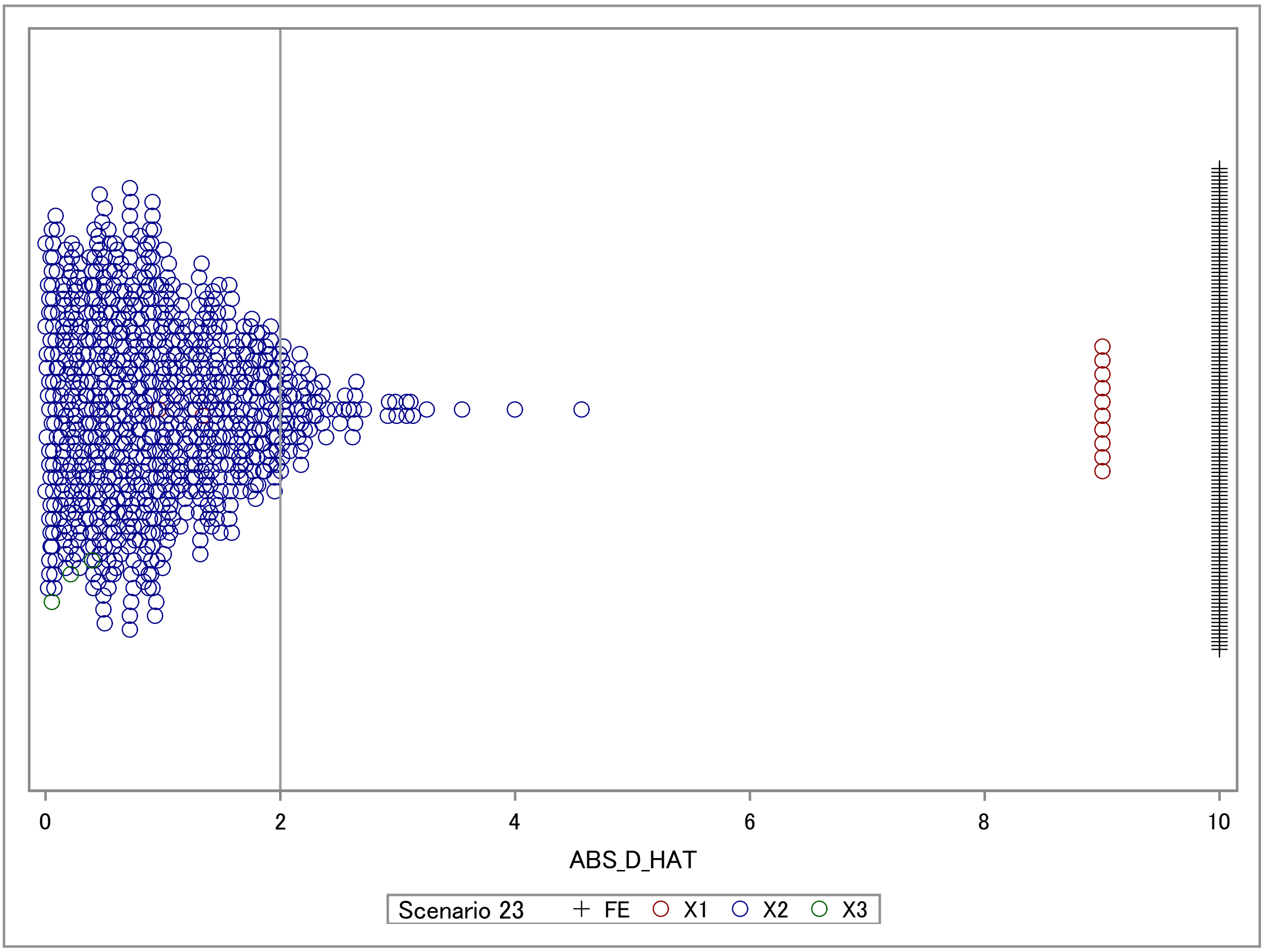
0
2
4
6
8
10
ABS_D_HAT
Scenario 23
FE
X1
X2
X3

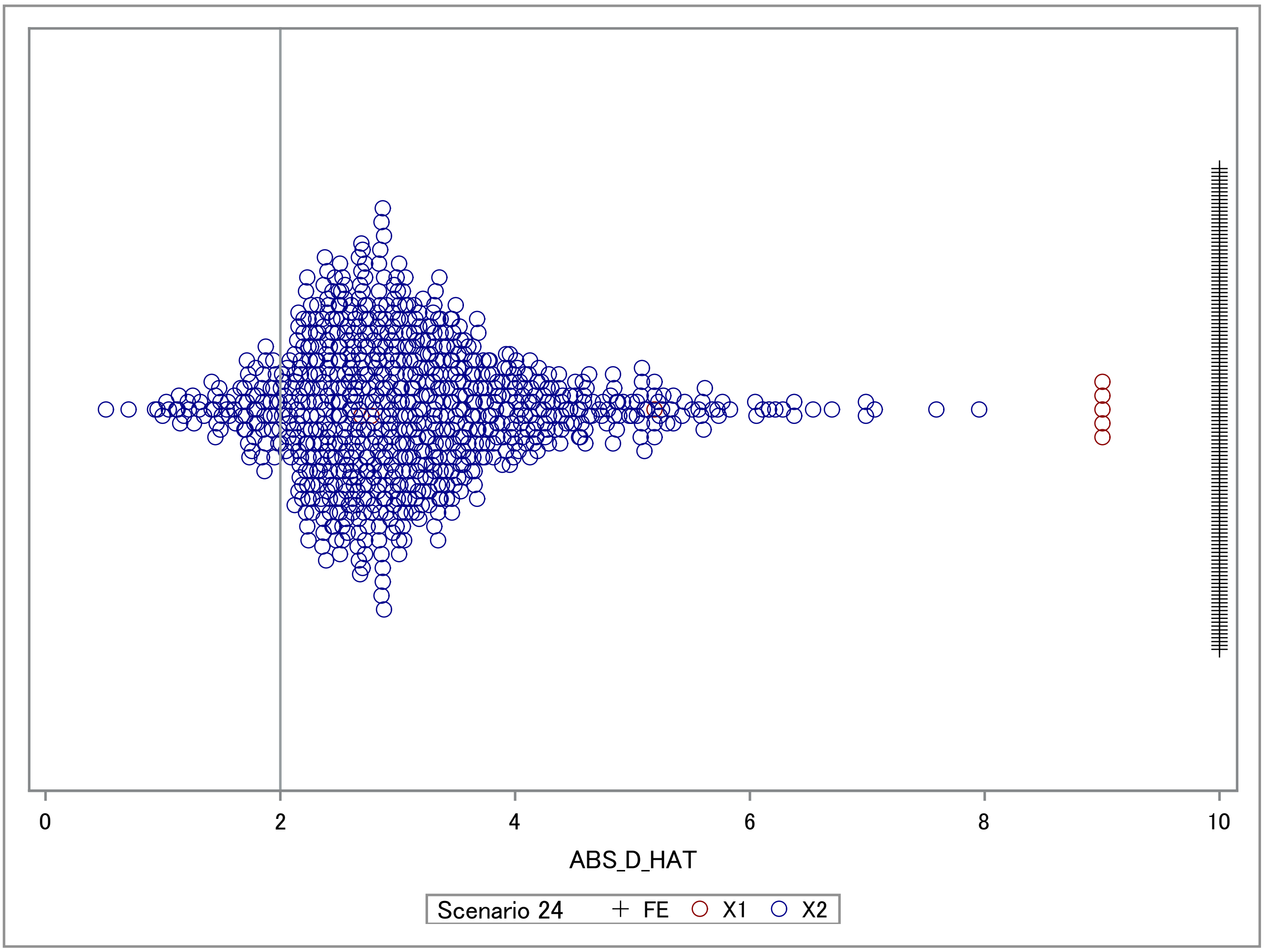
0
2
4
6
8
10
ABS_D_HAT
Scenario 24
FE
X1
X2

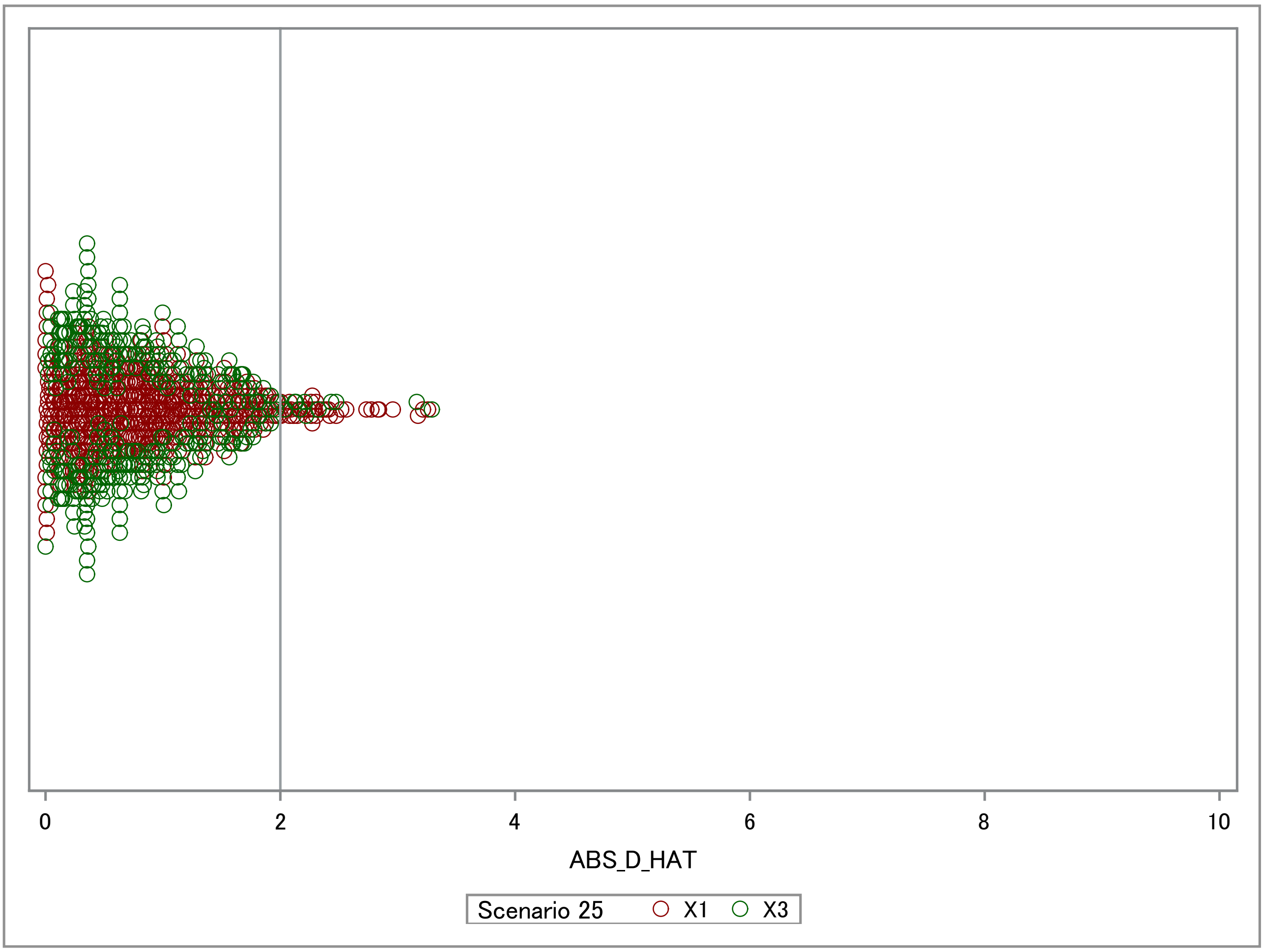
0
2
4
6
8
10
ABS_D_HAT
Scenario 25
X1
X3

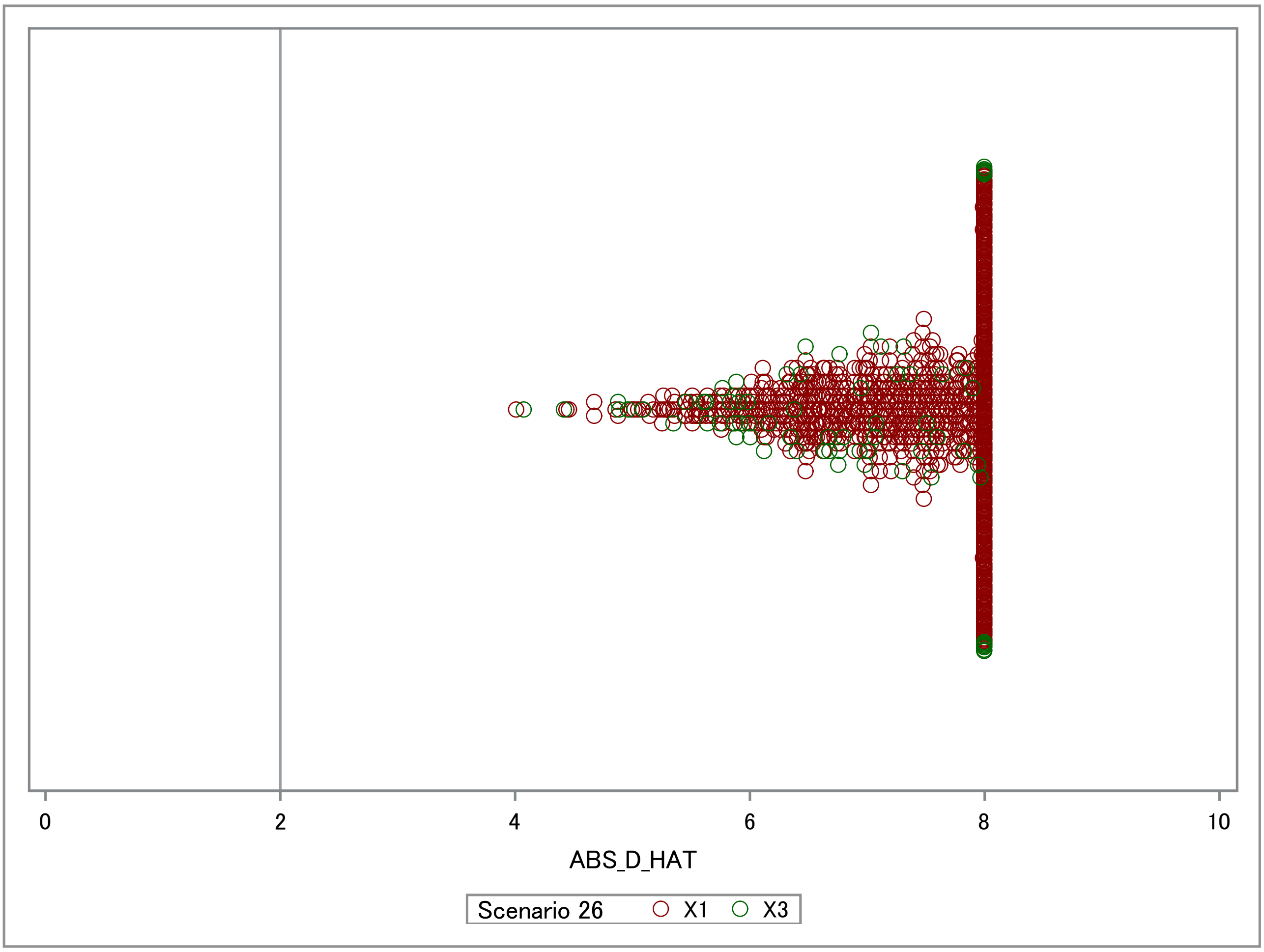
0
2
4
6
8
10
ABS_D_HAT
Scenario 26
X1
X3

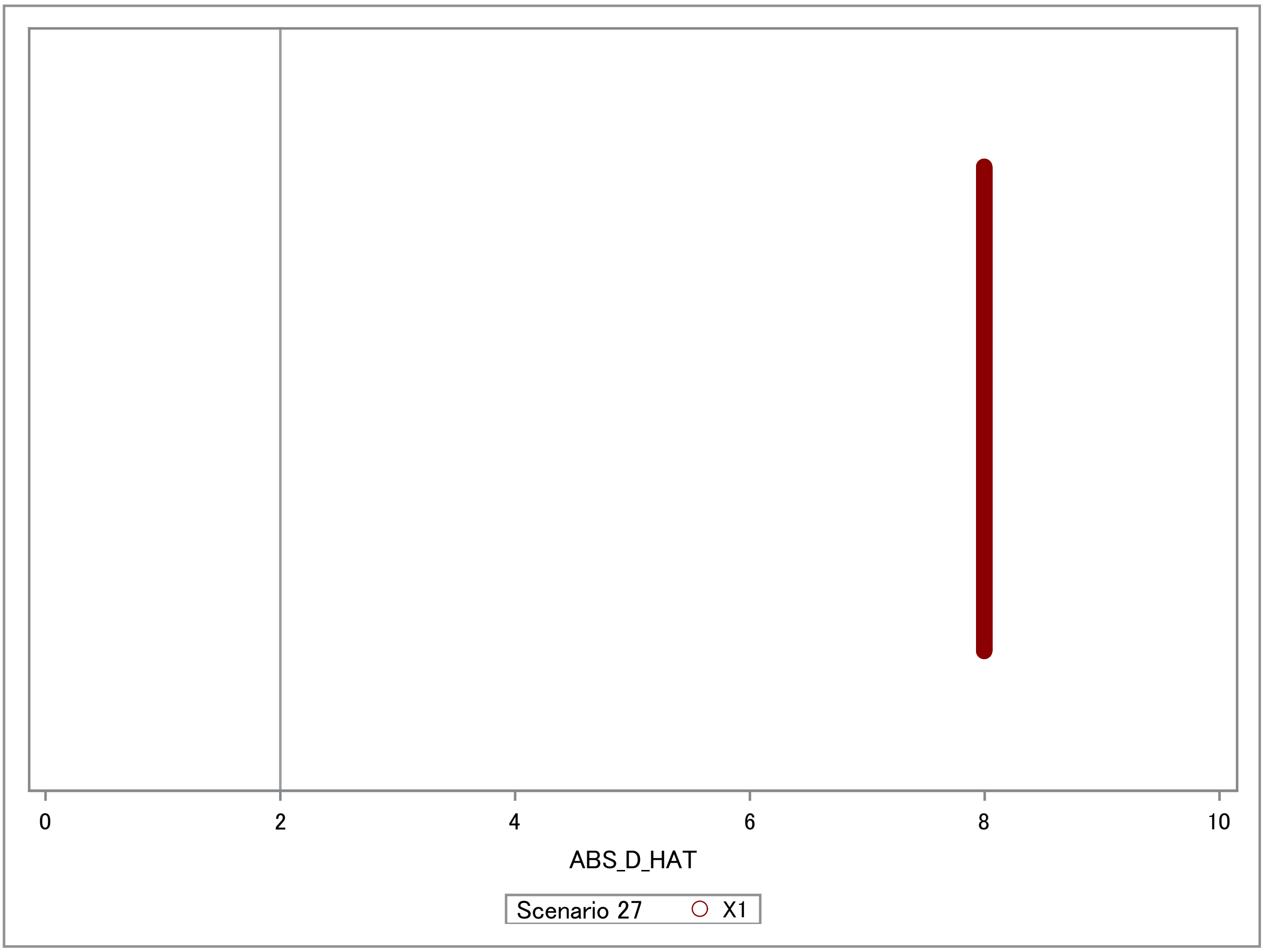

0
2
4
6
8
10
ABS_D_HAT
Scenario 27
X1

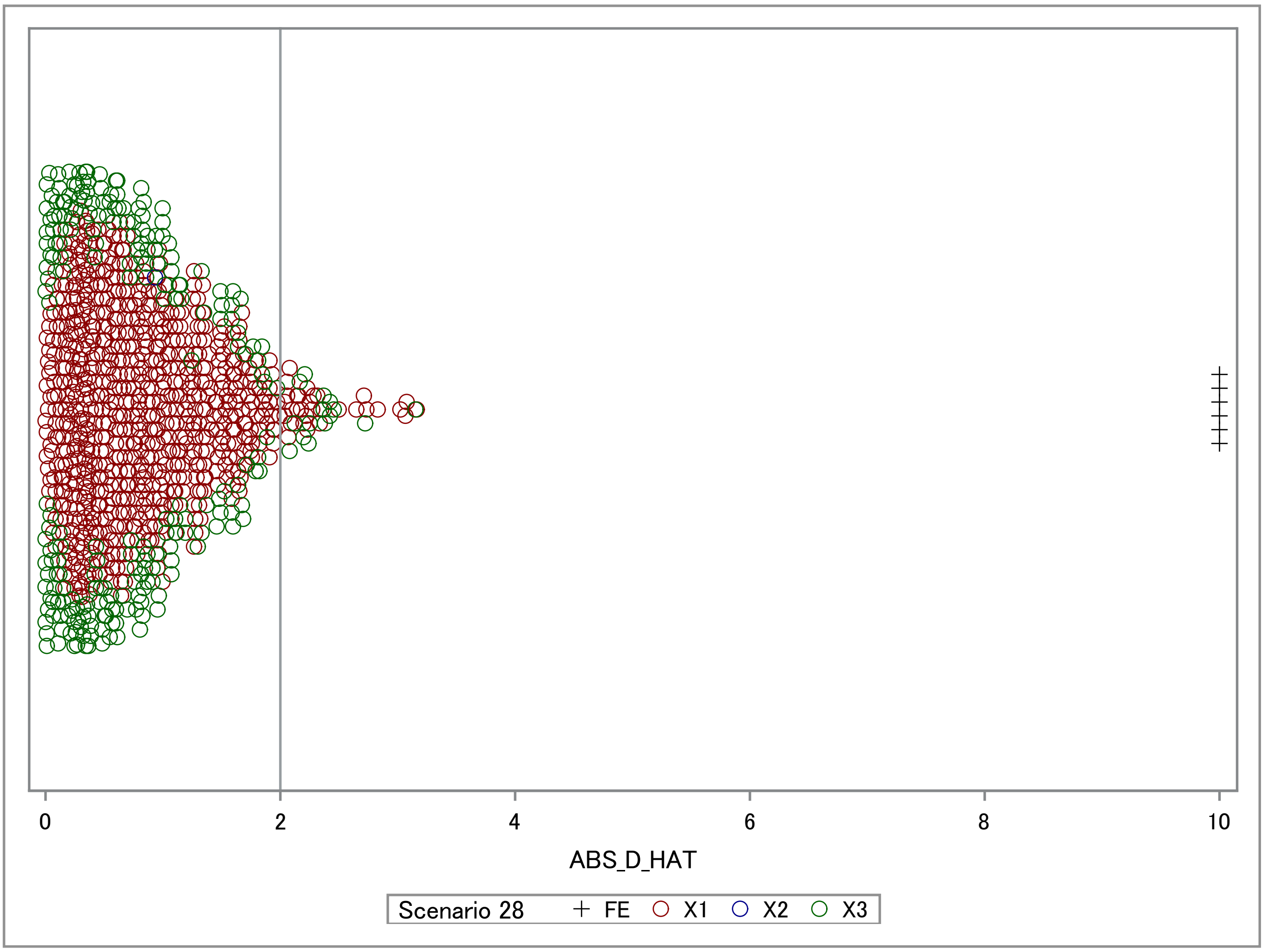
0
2
4
6
8
10
ABS_D_HAT
Scenario 28
FE
X1
X2
X3

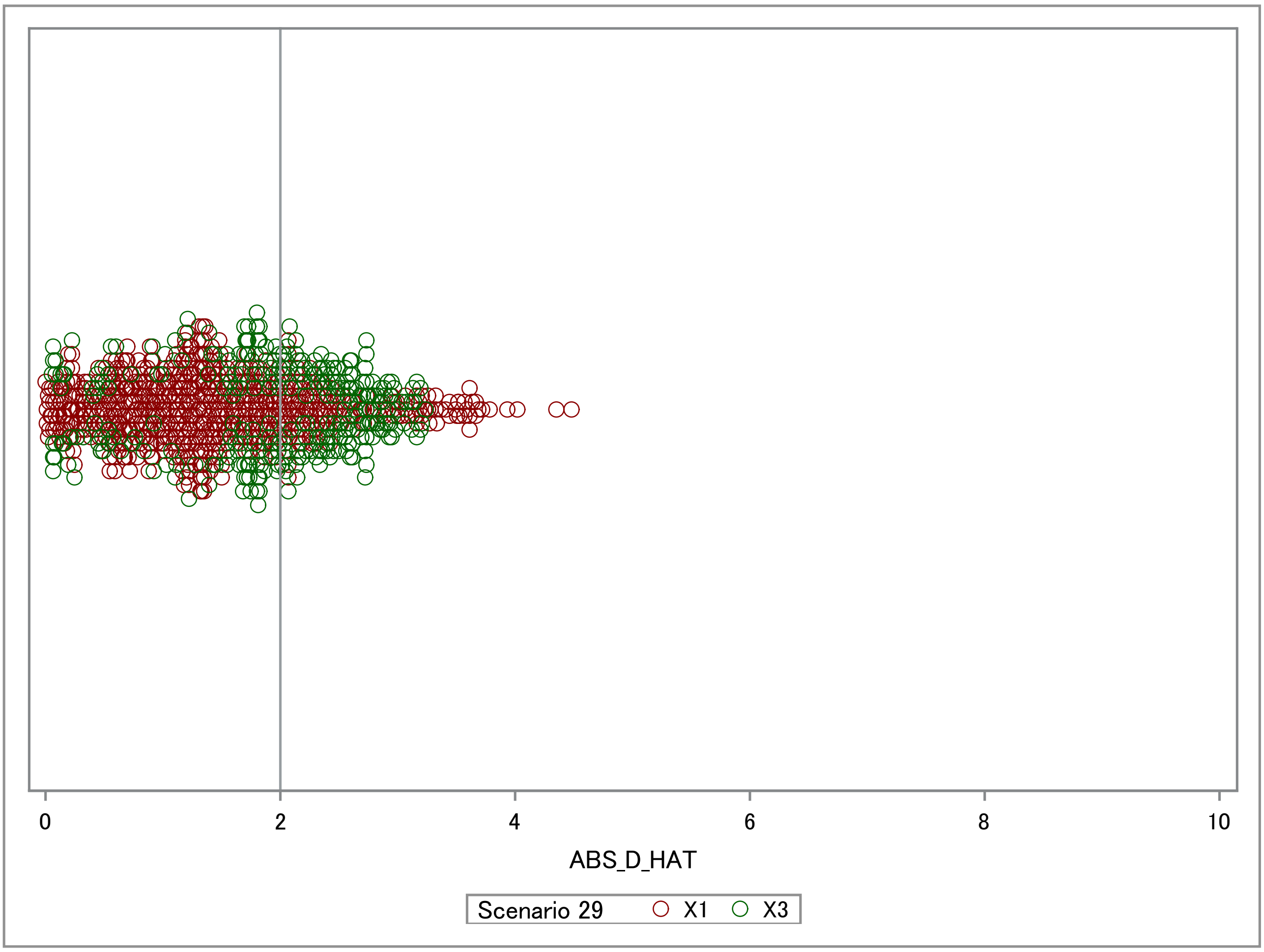
0
2
4
6
8
10
ABS_D_HAT
Scenario 29
X1
X3

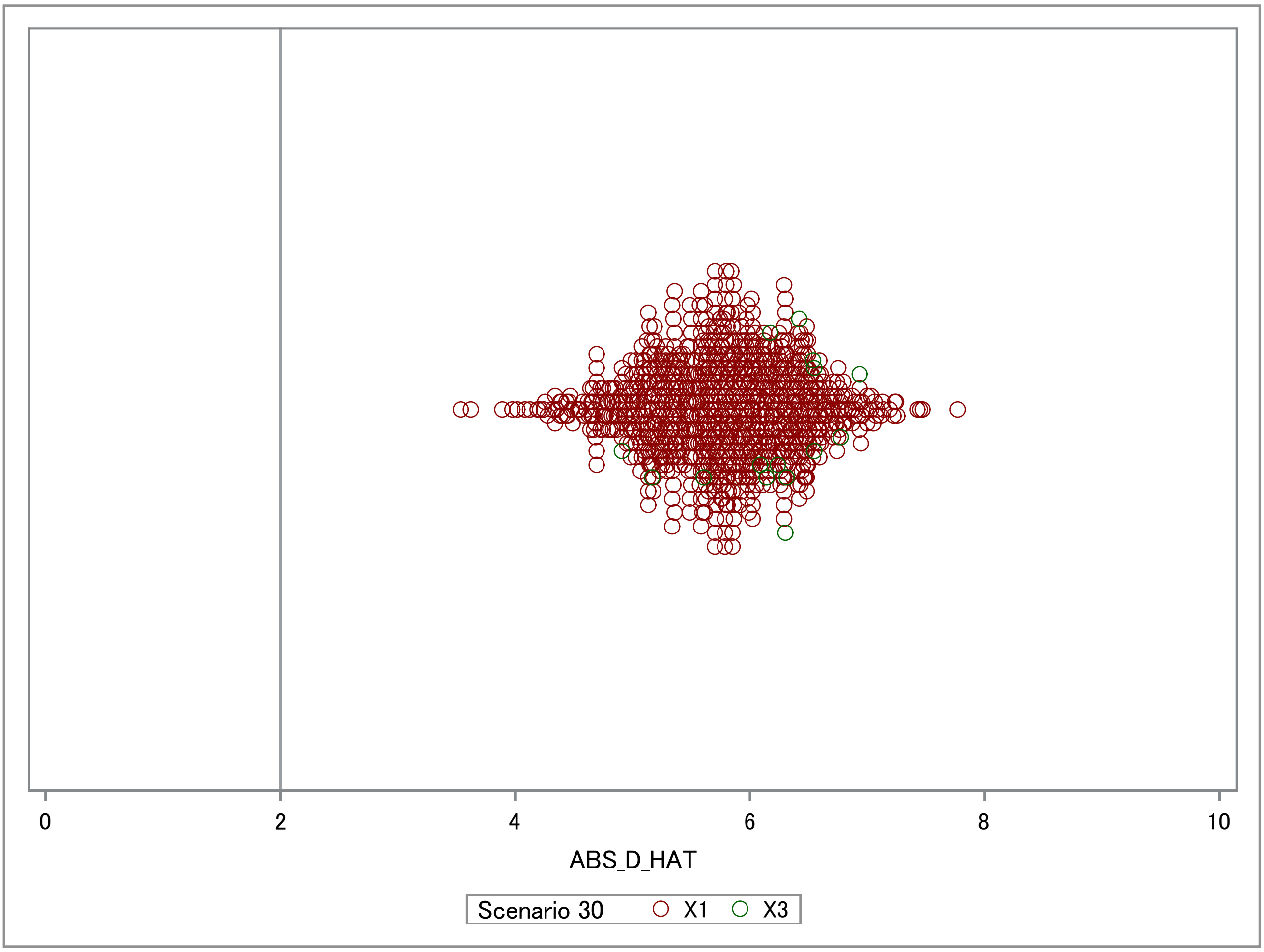

0
2
4
6
8
10
ABS_D_HAT
Scenario 30
X1
X3

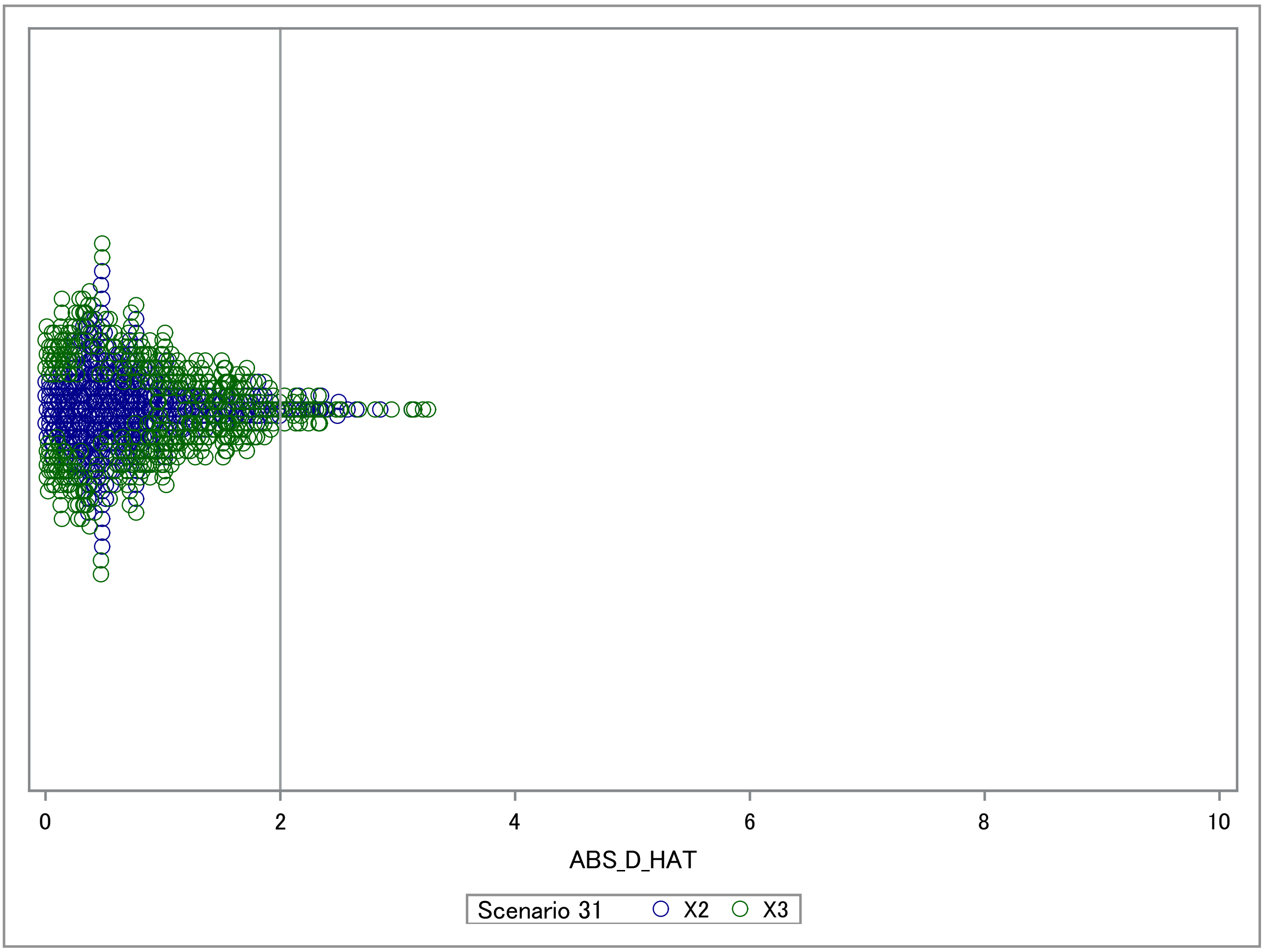
0
2
4
6
8
10
ABS_D_HAT
Scenario 31
X2
X3

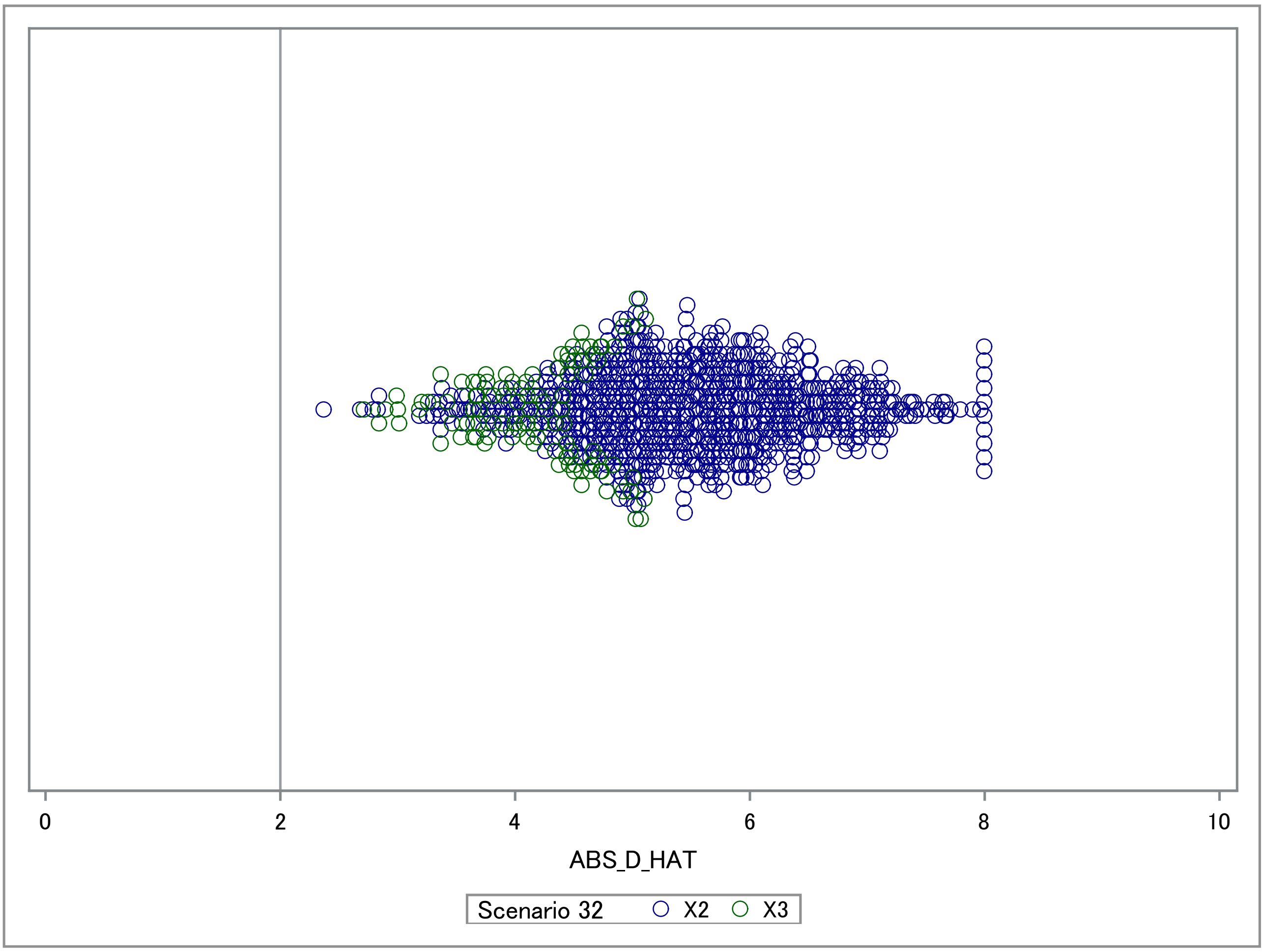
0
2
4
6
8
10
ABS_D_HAT
Scenario 32
X2
X3

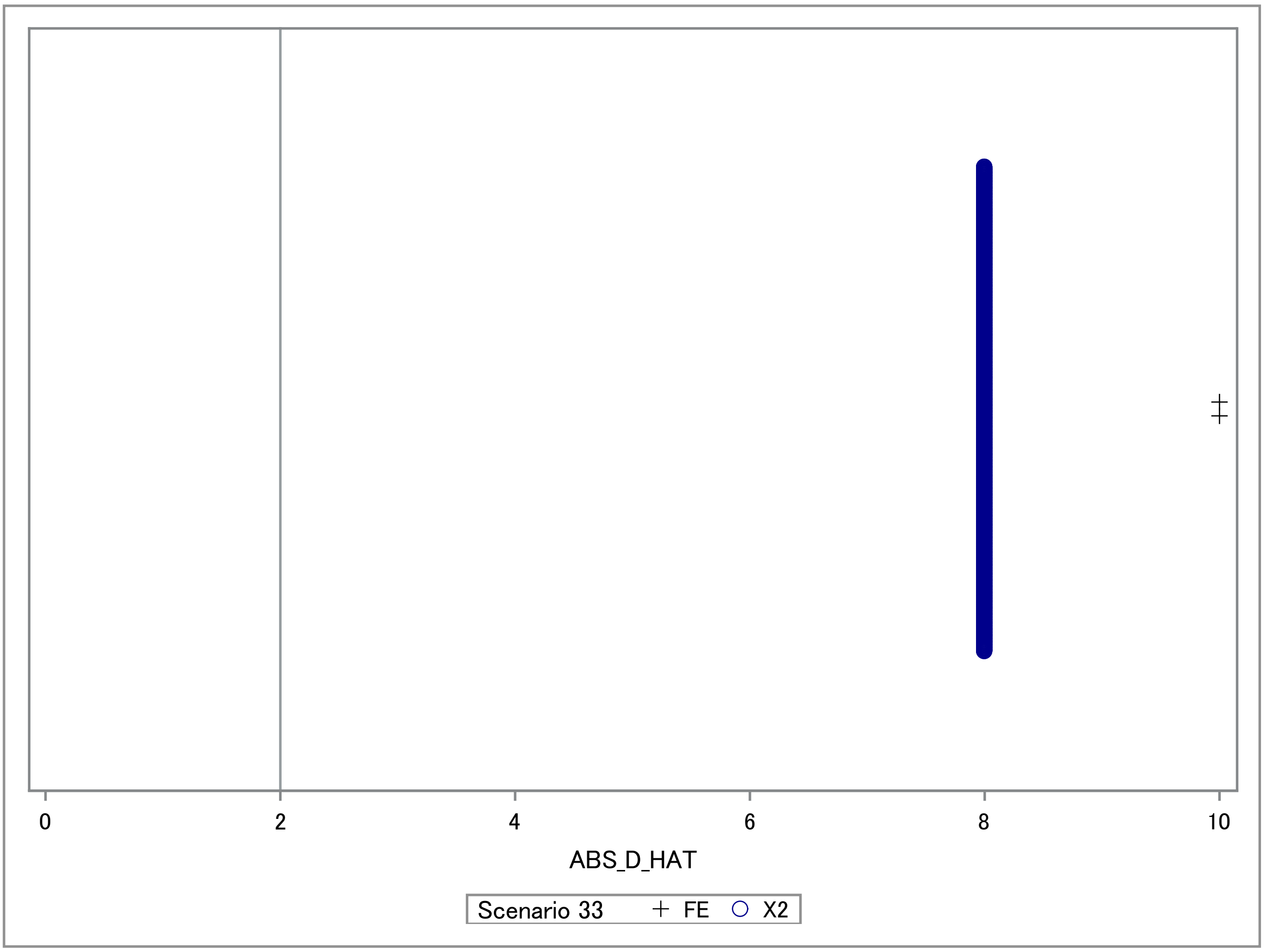
0
2
4
6
8
10
ABS_D_HAT
Scenario 33
FE
X2

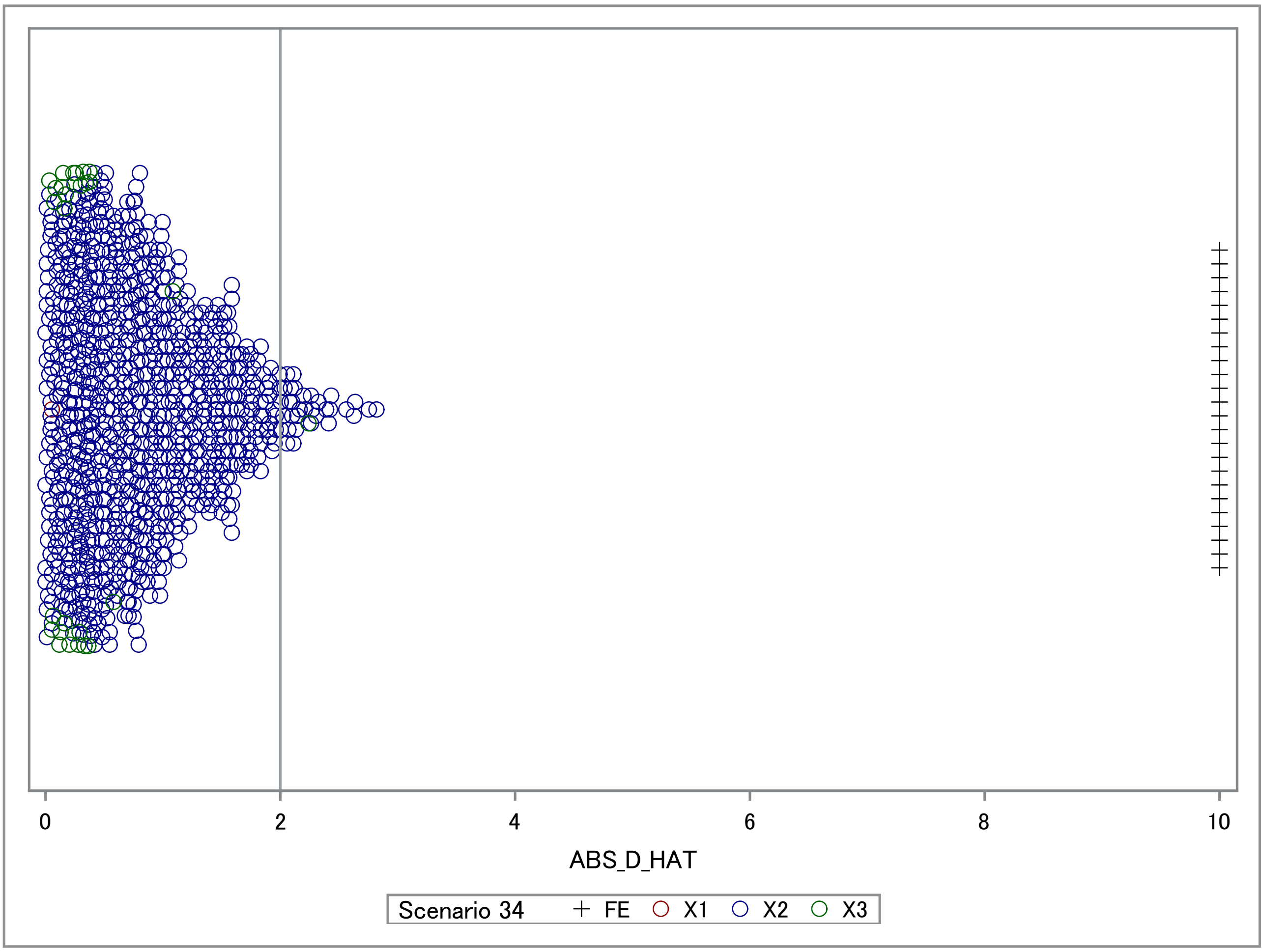
0
2
4
6
8
10
ABS_D_HAT
Scenario 34
FE
X1
X2
X3

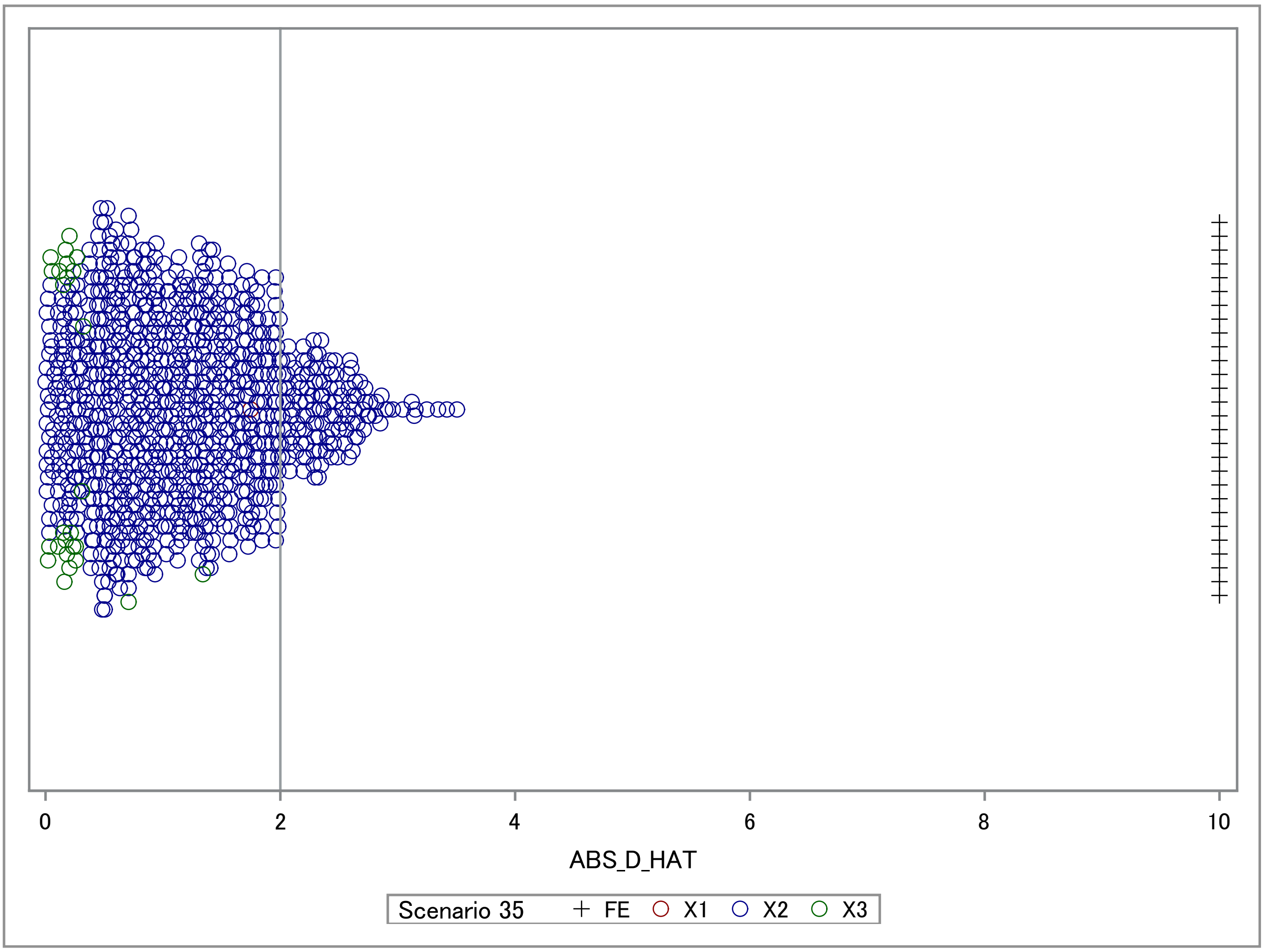
0
2
4
6
8
10
ABS_D_HAT
Scenario 35
FE
X1
X2
X3

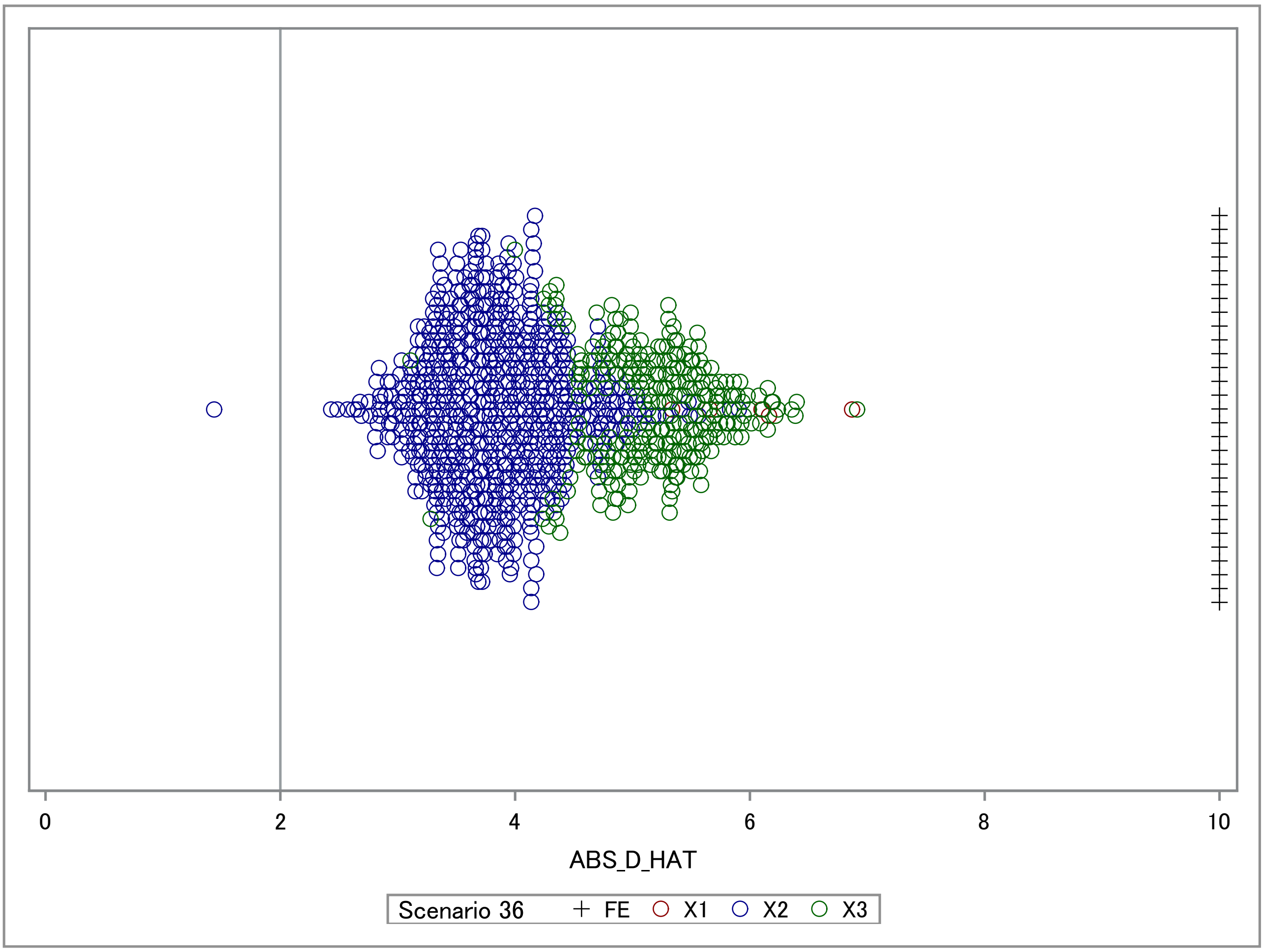
0
2
4
6
8
10
ABS_D_HAT
Scenario 36
FE
X1
X2
X3

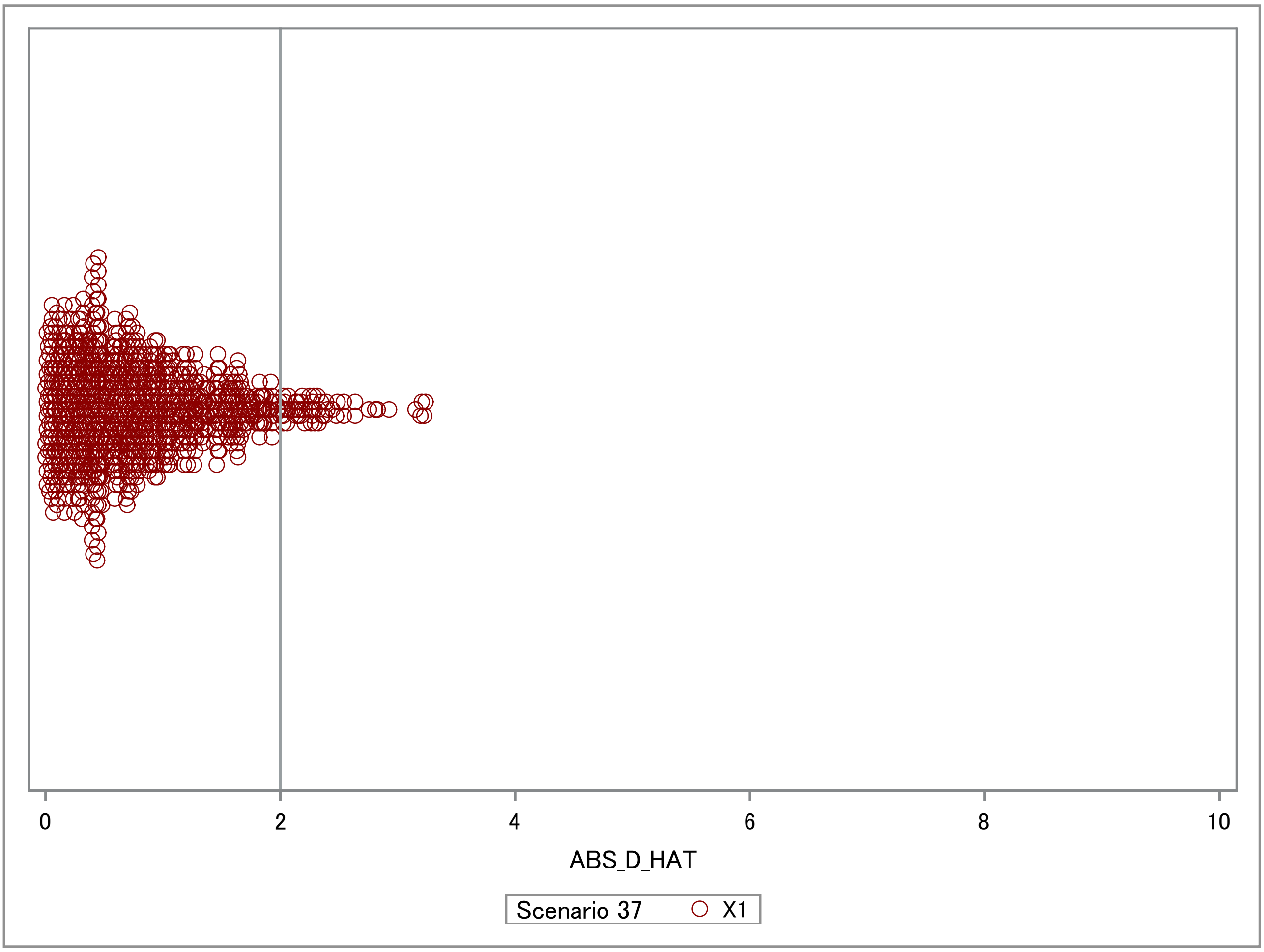
0
2
4
6
8
10
ABS_D_HAT
Scenario 37
X1

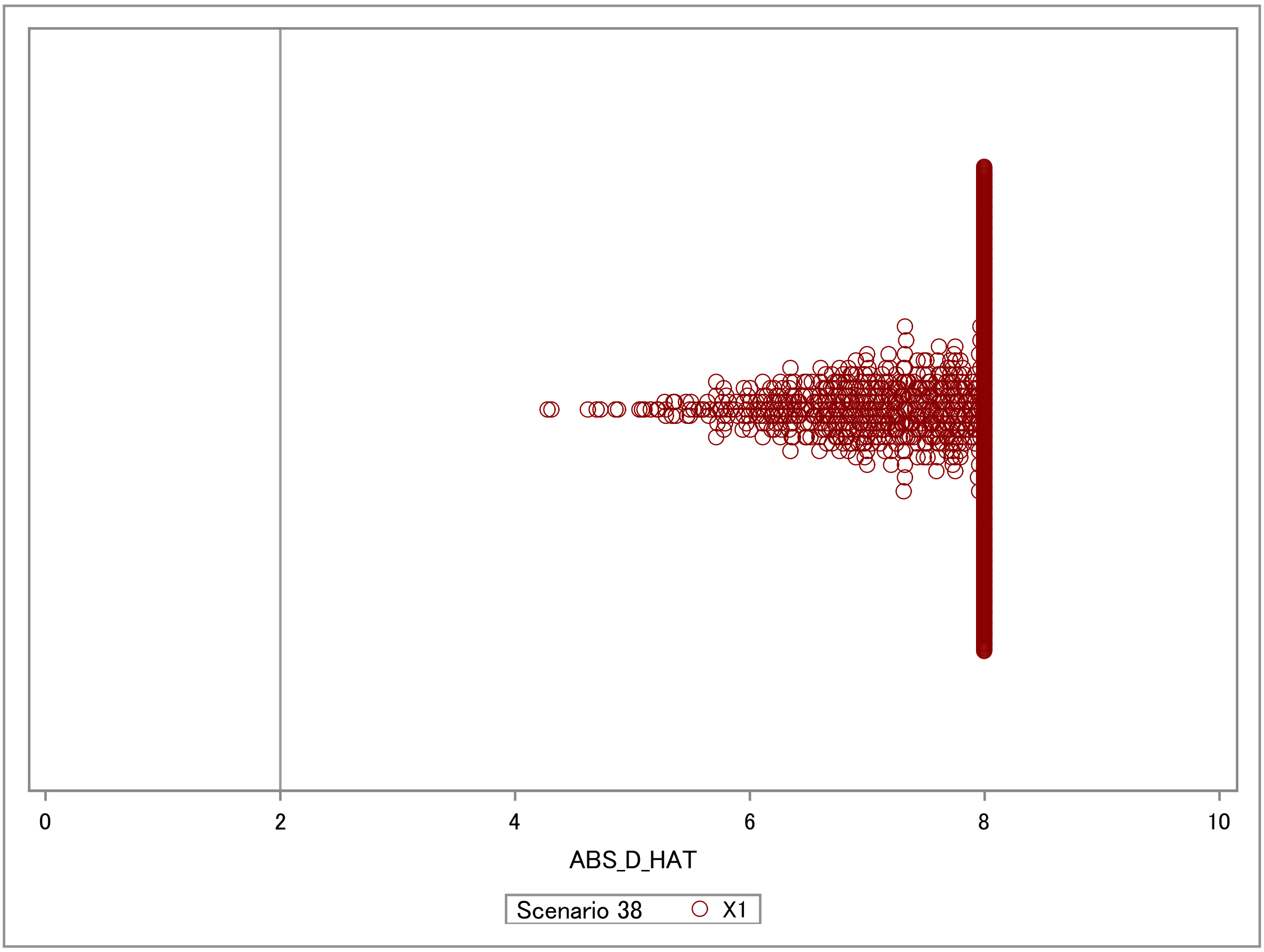
0
2
4
6
8
10
ABS_D_HAT
Scenario 38
X1

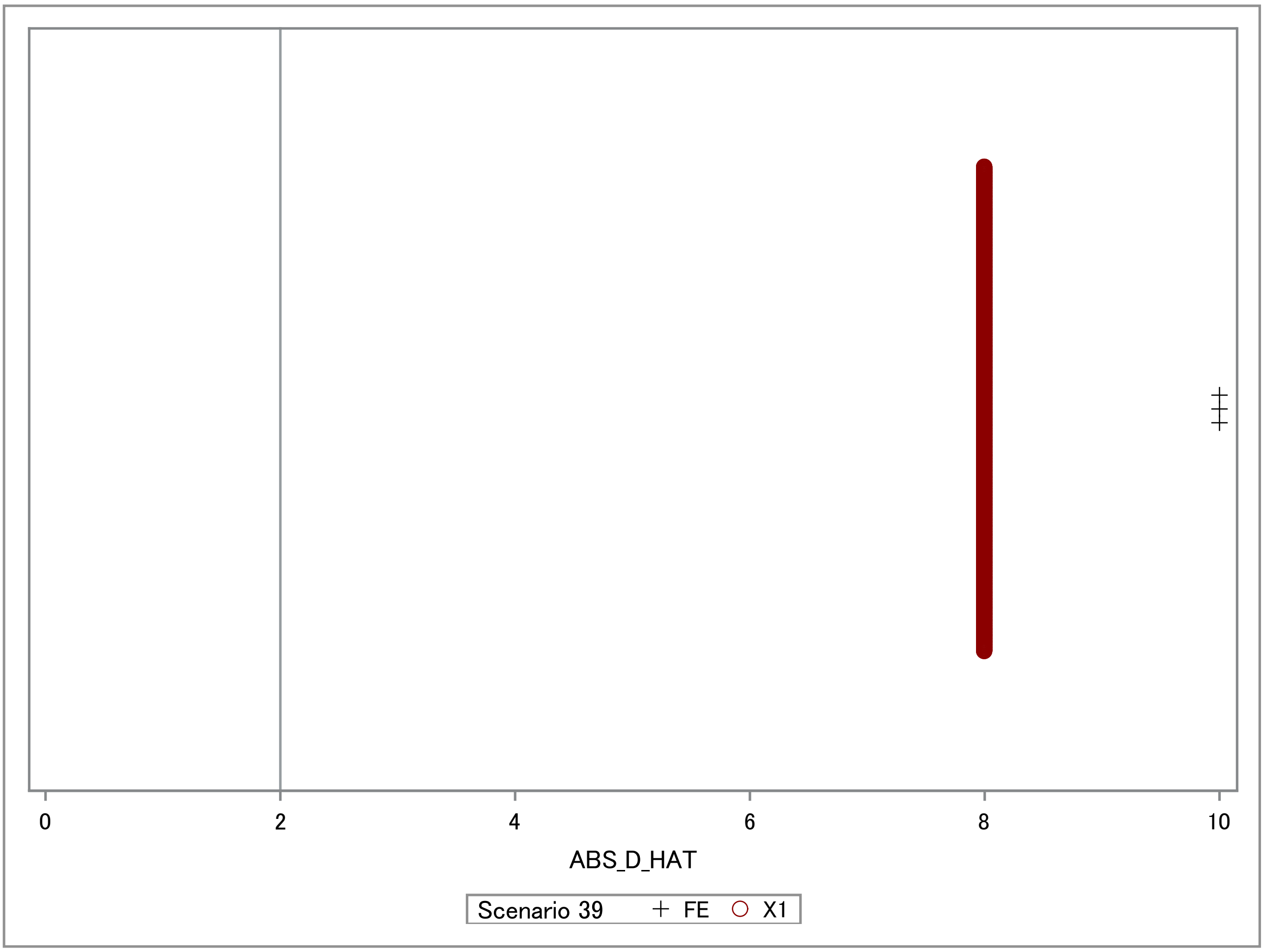
0
2
4
6
8
10
ABS_D_HAT
Scenario 39
FE
X1

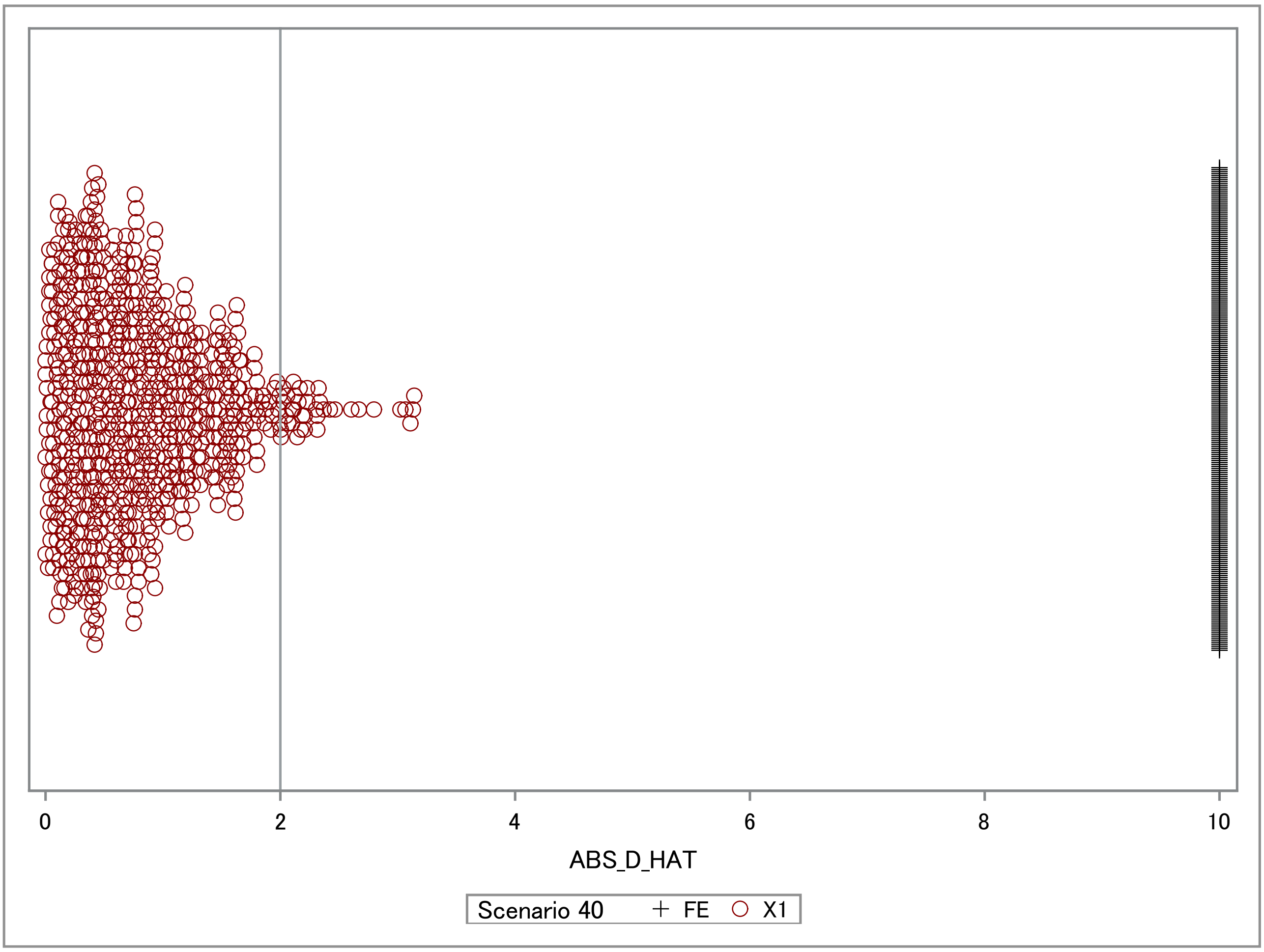
0
2
4
6
8
10
ABS_D_HAT
Scenario 40
FE
X1

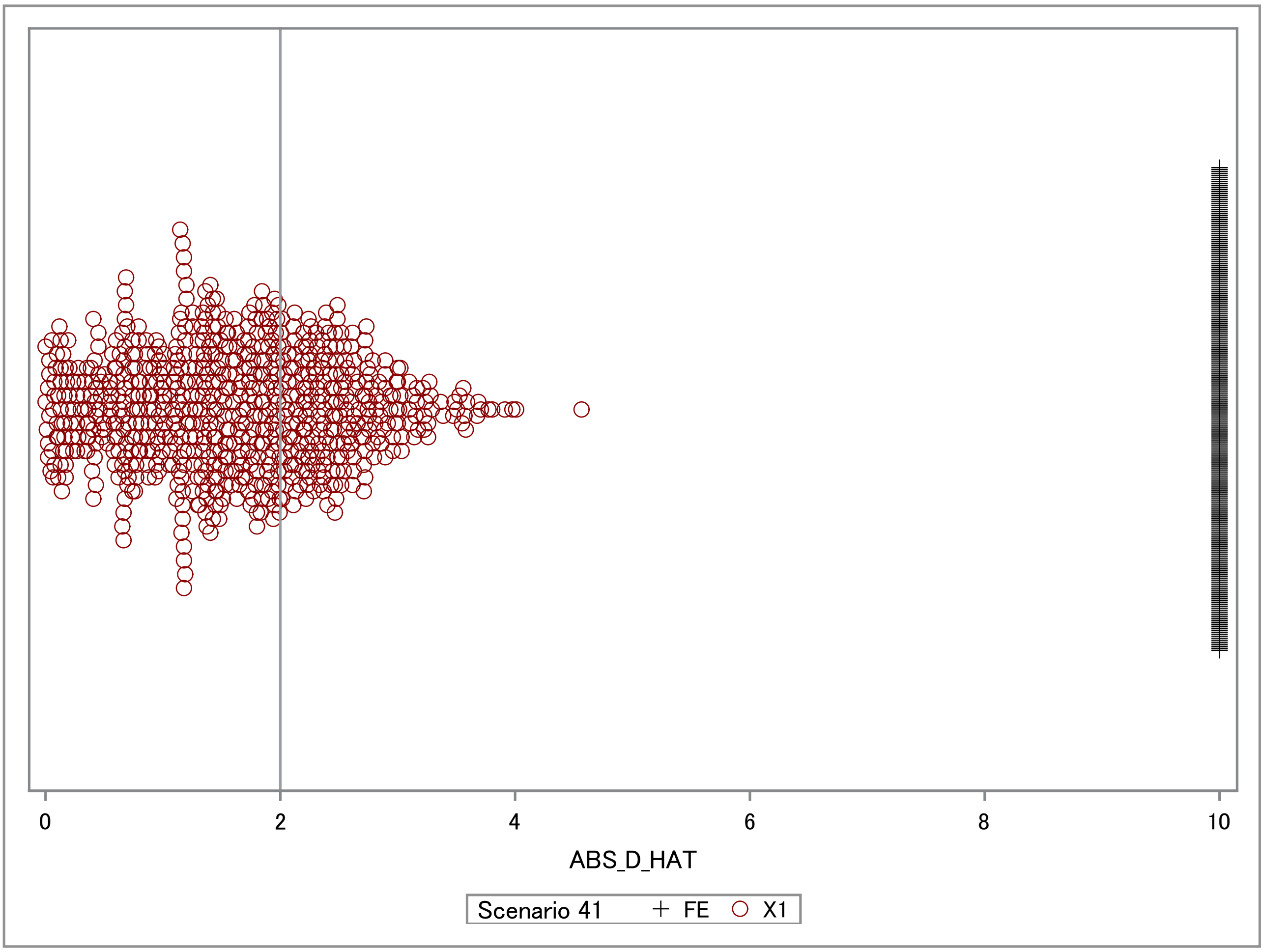
0
2
4
6
8
10
ABS_D_HAT
Scenario 41
FE
X1

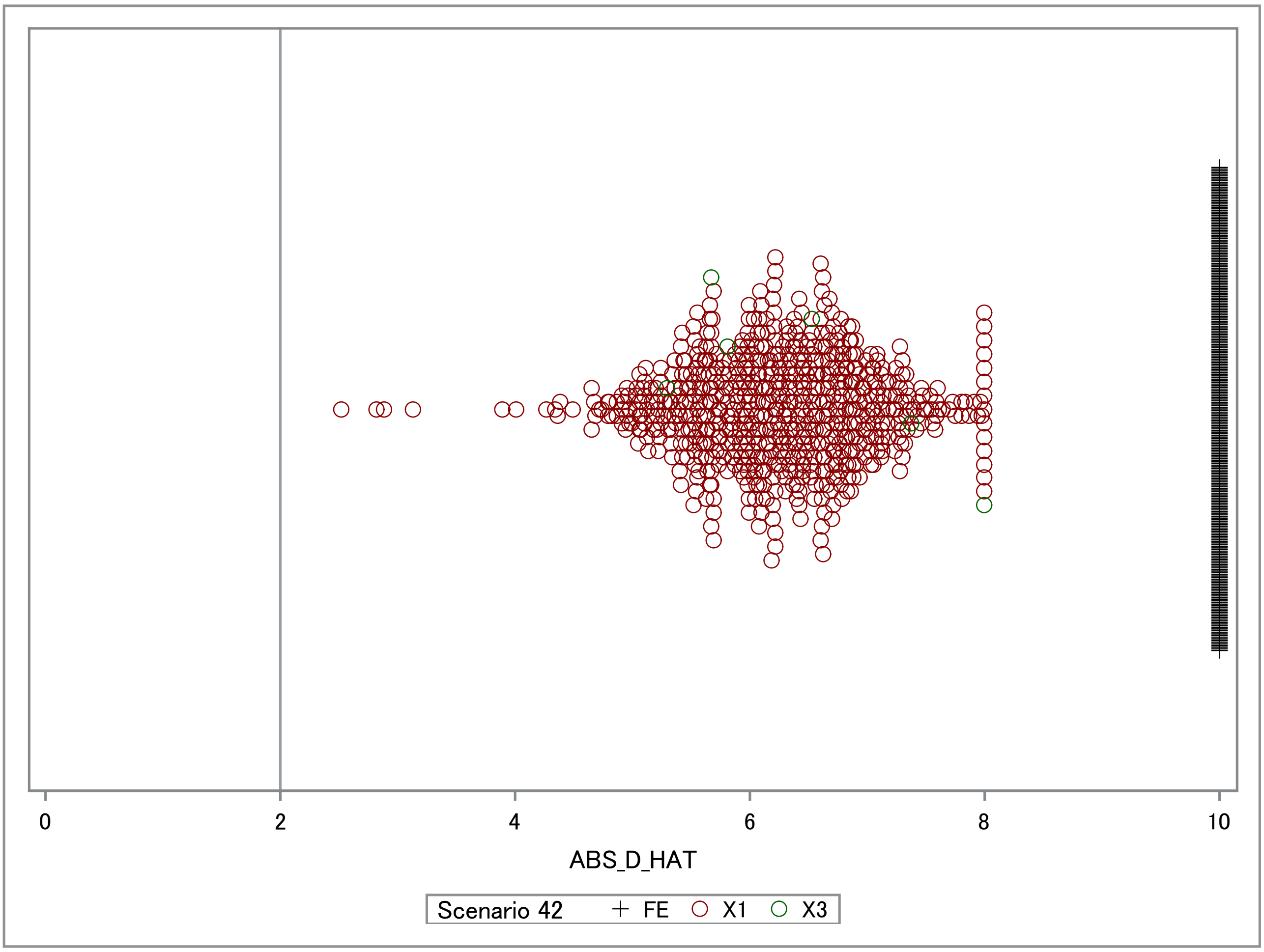
0
2
4
6
8
10
ABS_D_HAT
Scenario 42
FE
X1
X3

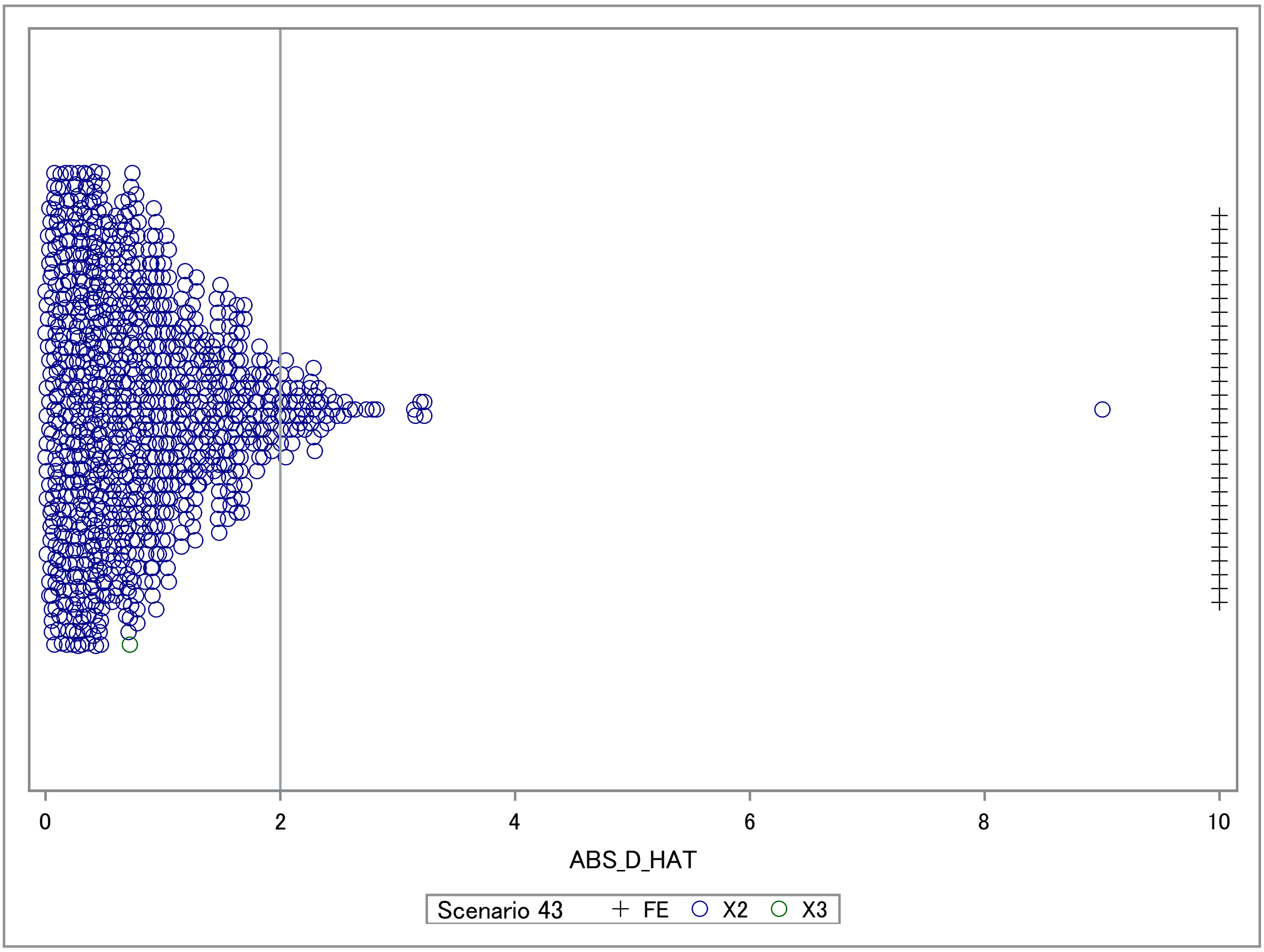
0
2
4
6
8
10
ABS_D_HAT
Scenario 43
FE
X2
X3

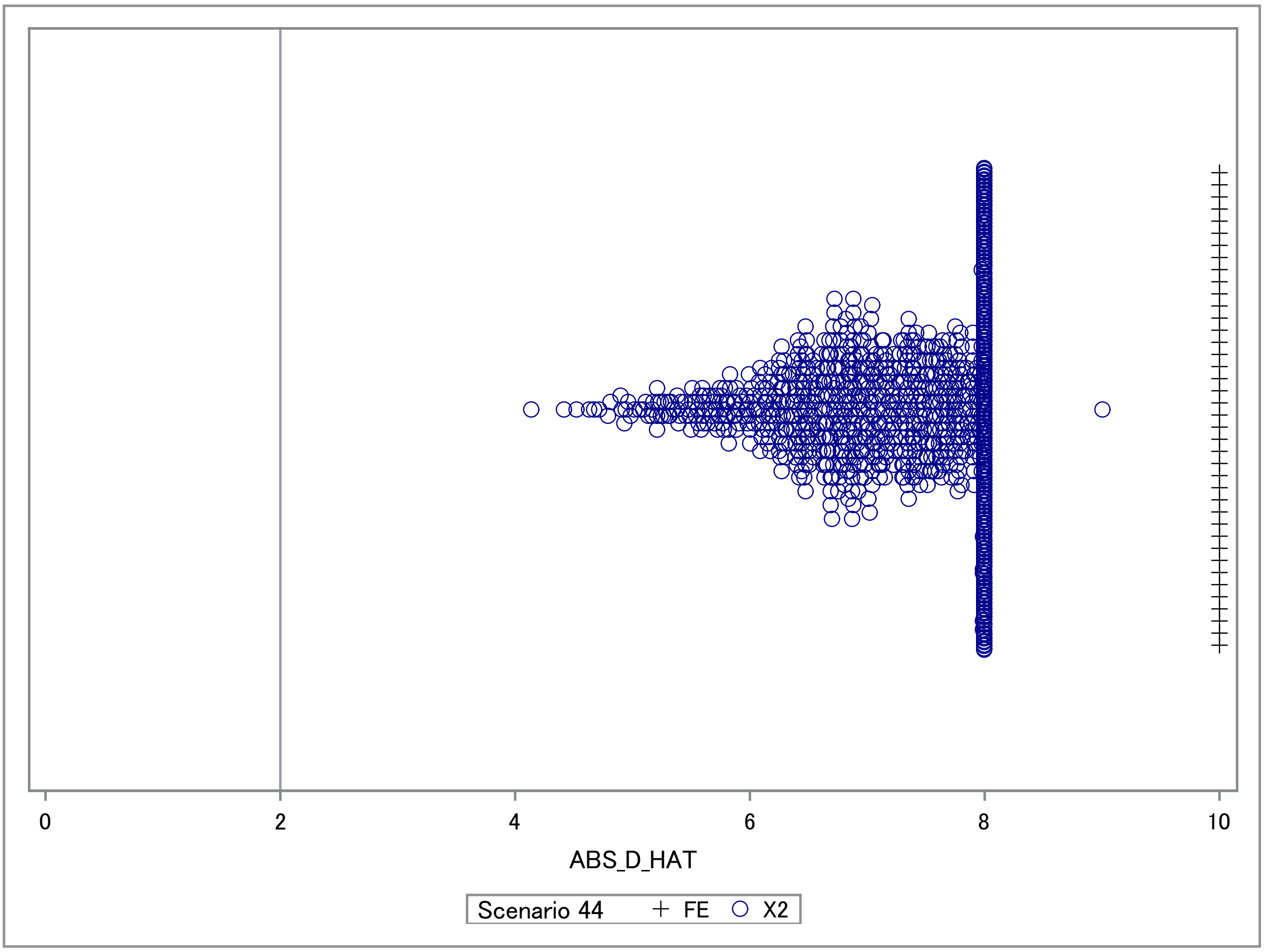
0
2
4
6
8
10
ABS_D_HAT
Scenario 44
FE
X2

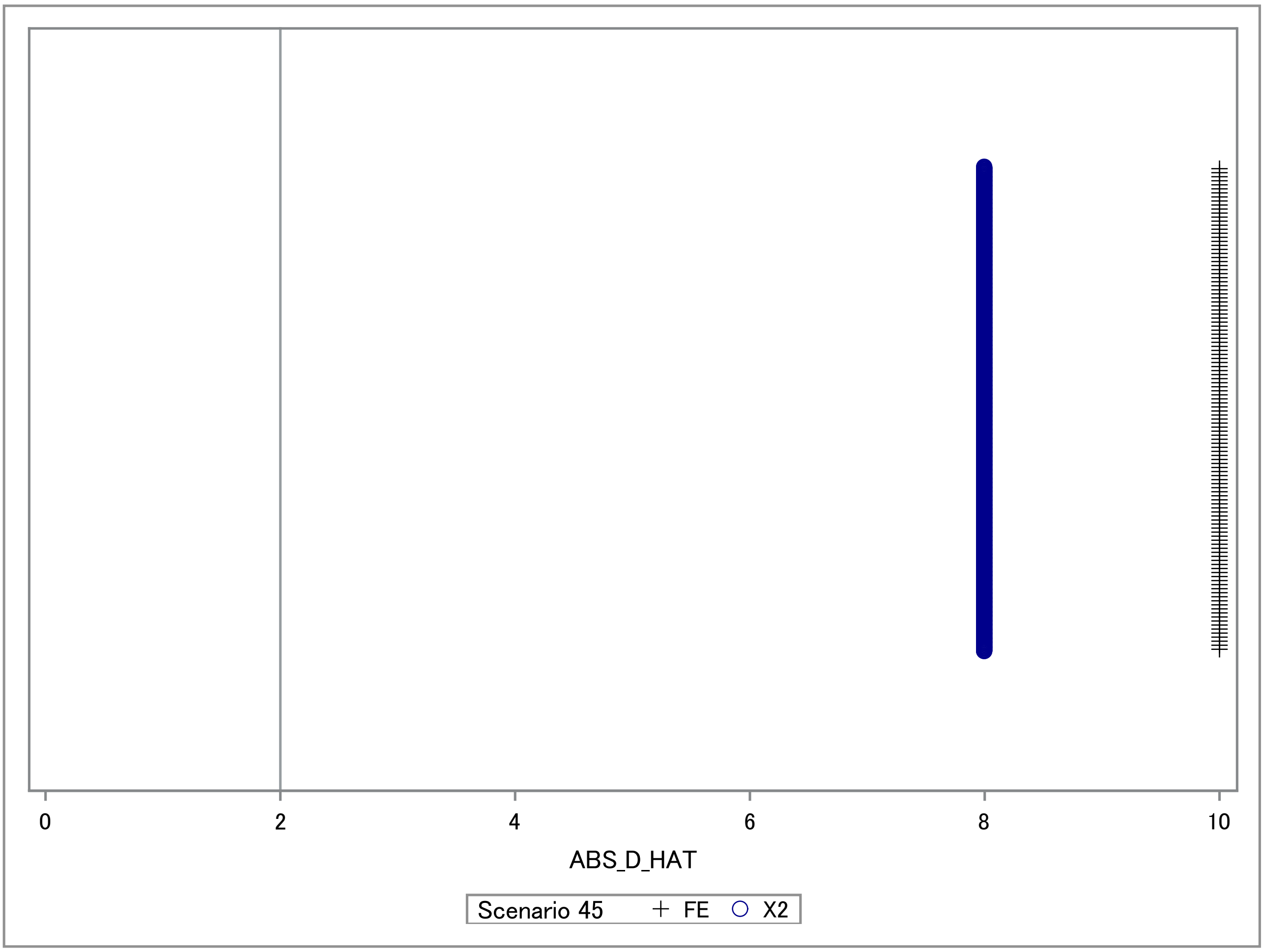
0
2
4
6
8
10
ABS_D_HAT
Scenario 45
FE
X2

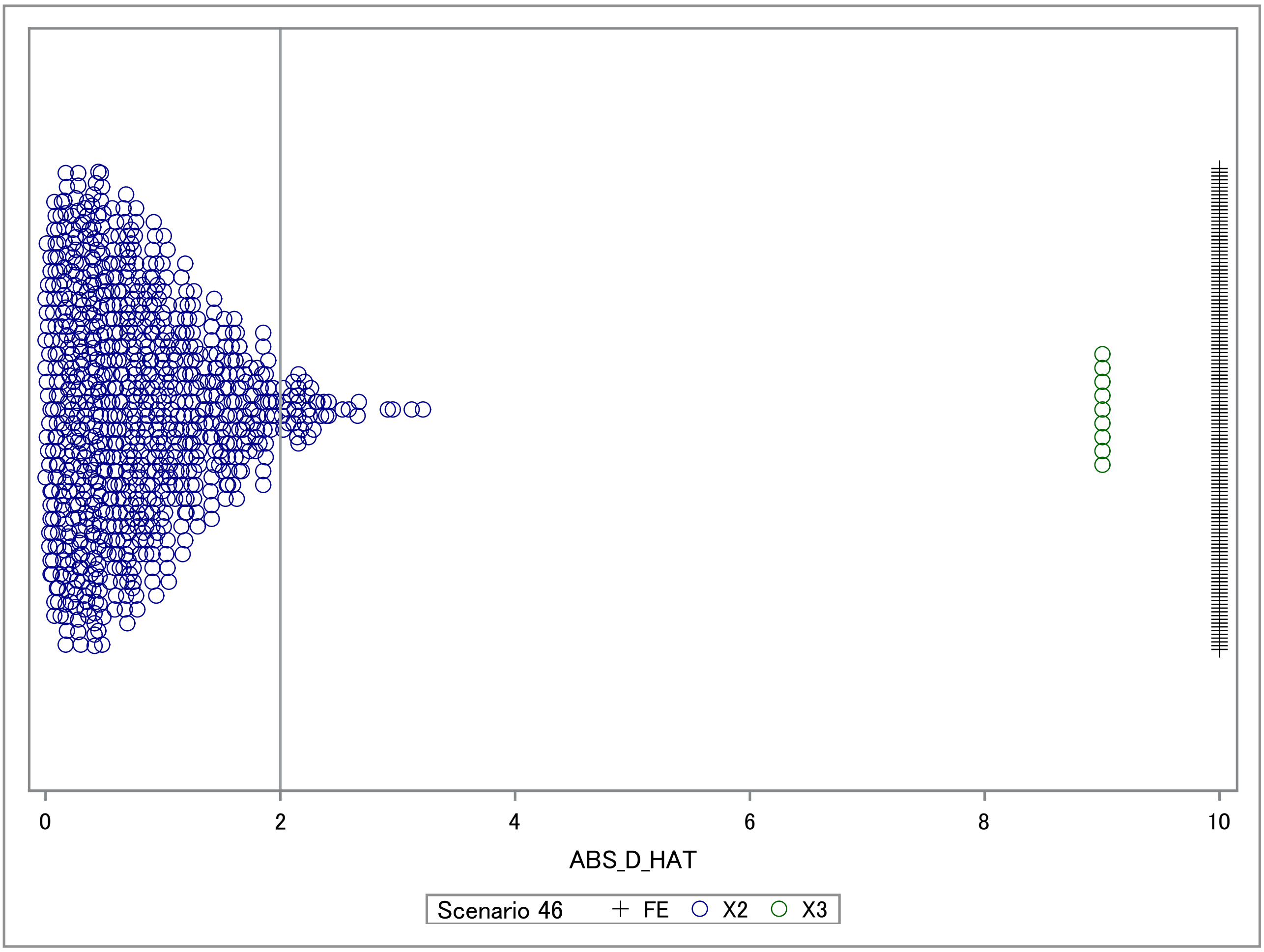
0
2
4
6
8
10
ABS_D_HAT
Scenario 46
FE
X2
X3

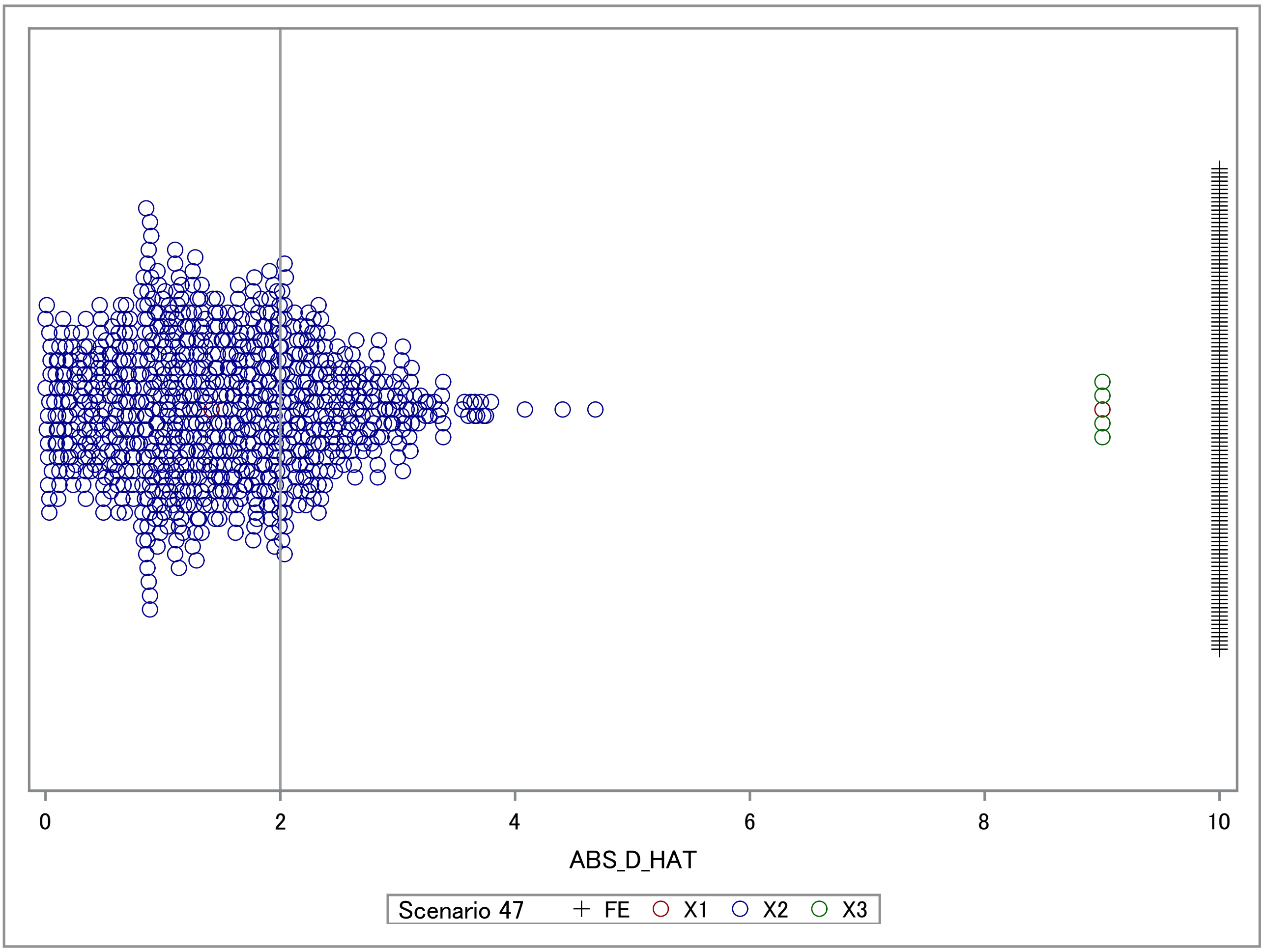
0
2
4
6
8
10
ABS_D_HAT
Scenario 47
FE
X1
X2
X3

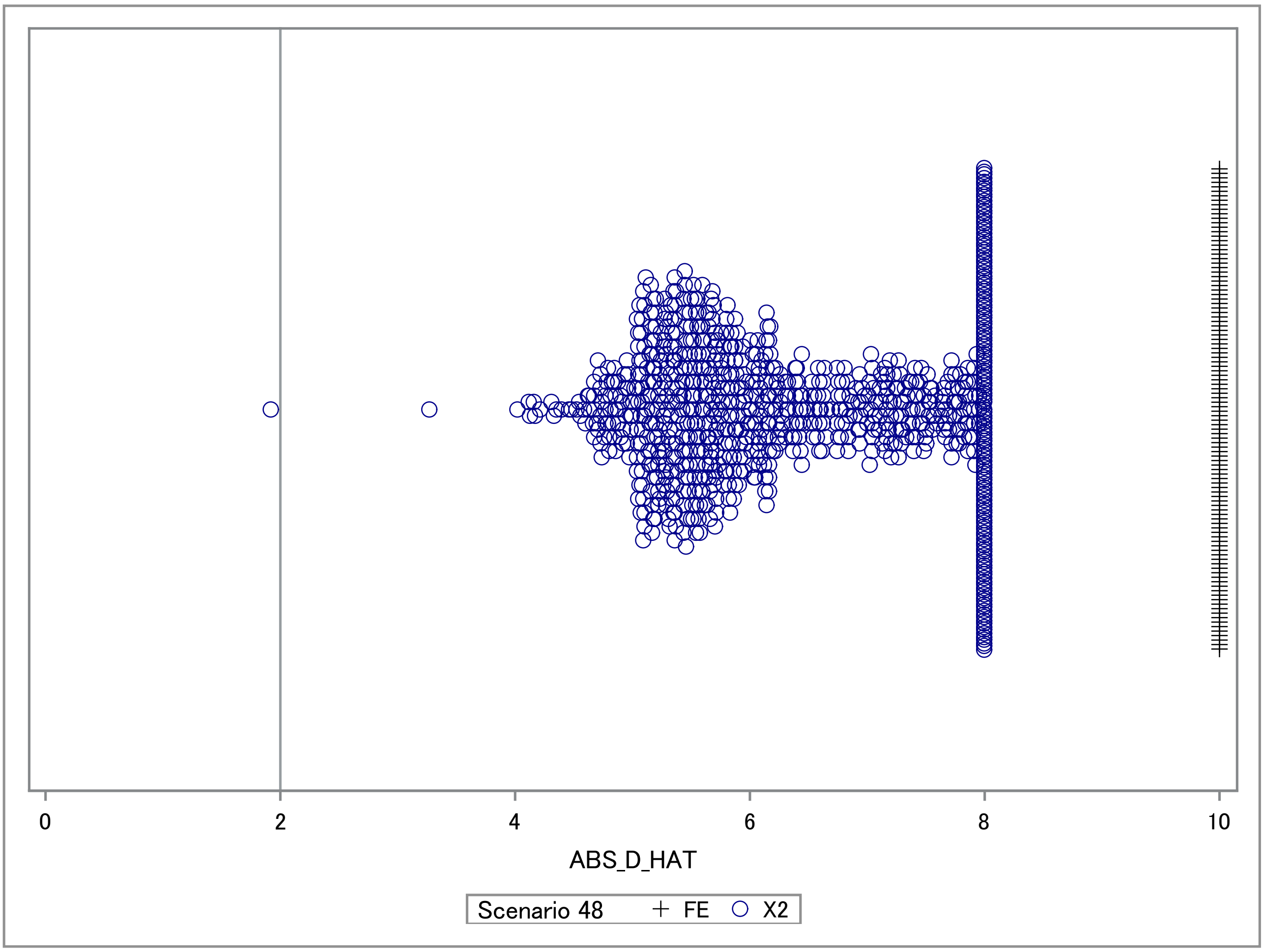
0
2
4
6
8
10
ABS_D_HAT
Scenario 48
FE
X2
