# Supplementary material for: Model-based quantification of protein–protein interaction aberrations for exploring dysregulated signalling pathways through pathway maps and gene expression levels
Source: BMC Bioinformatics. 2026 Jul 8;27:263. doi: 10.1186/s12859-026-06543-8 (PMC13732913; doi:10.1186/s12859-026-06543-8)
Supplement: Supplementary file 3 [file 12859_2026_6543_MOESM3_ESM.pdf]

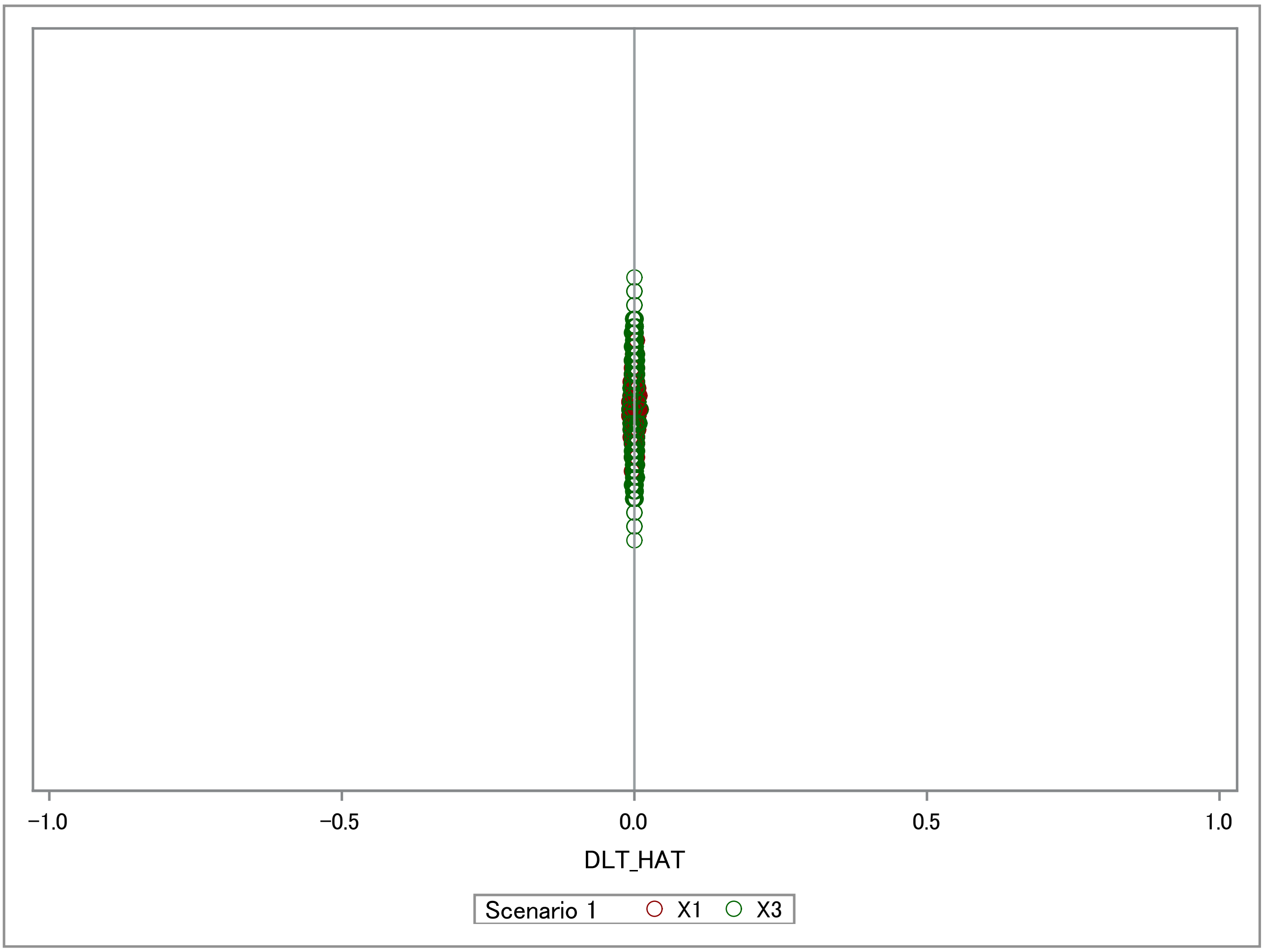

−1.0
−0.5
0.0
0.5
1.0
DLT_HAT
Scenario 1
X1
X3

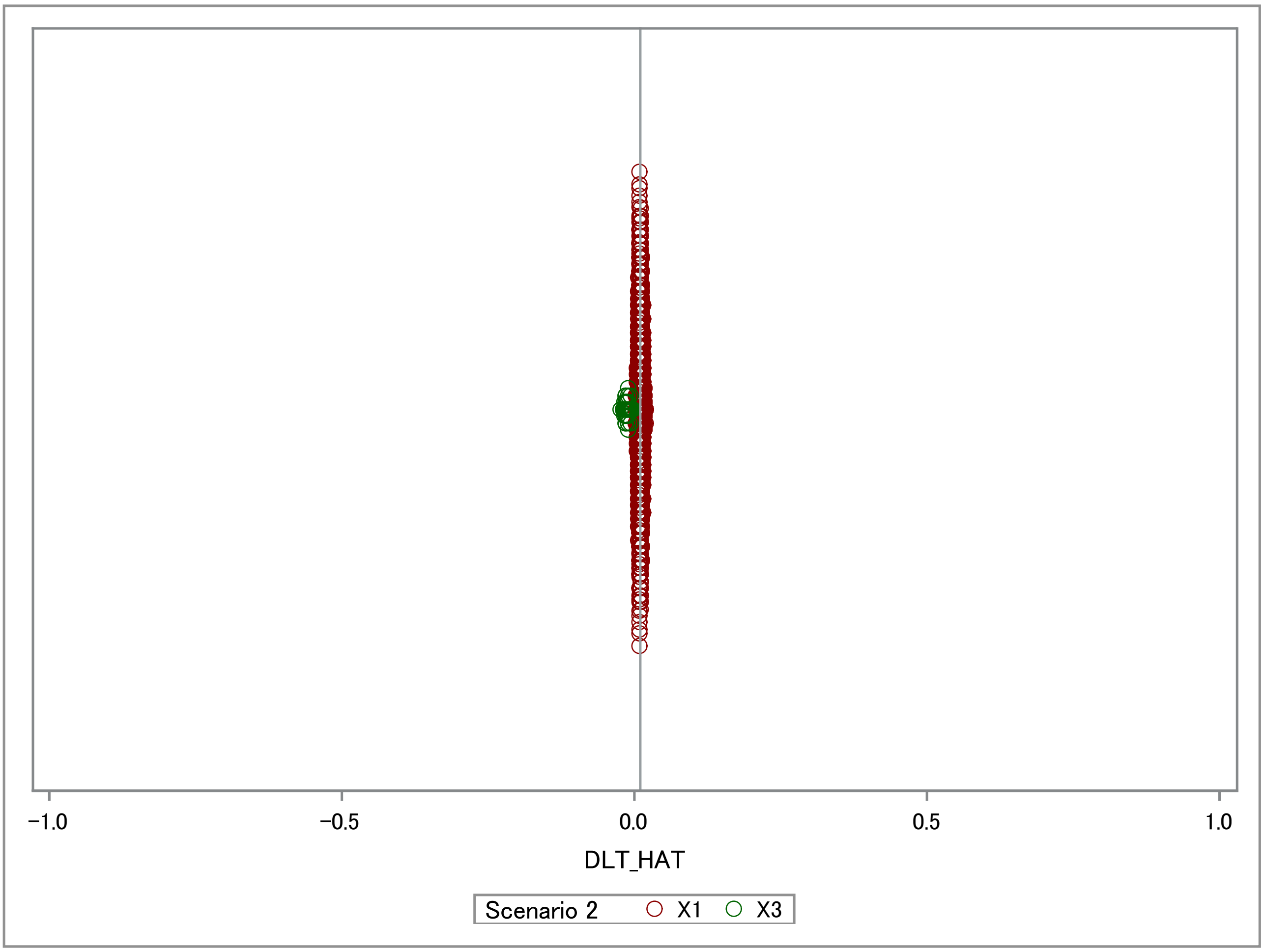

−1.0
−0.5
0.0
0.5
1.0
DLT_HAT
Scenario 2
X1
X3

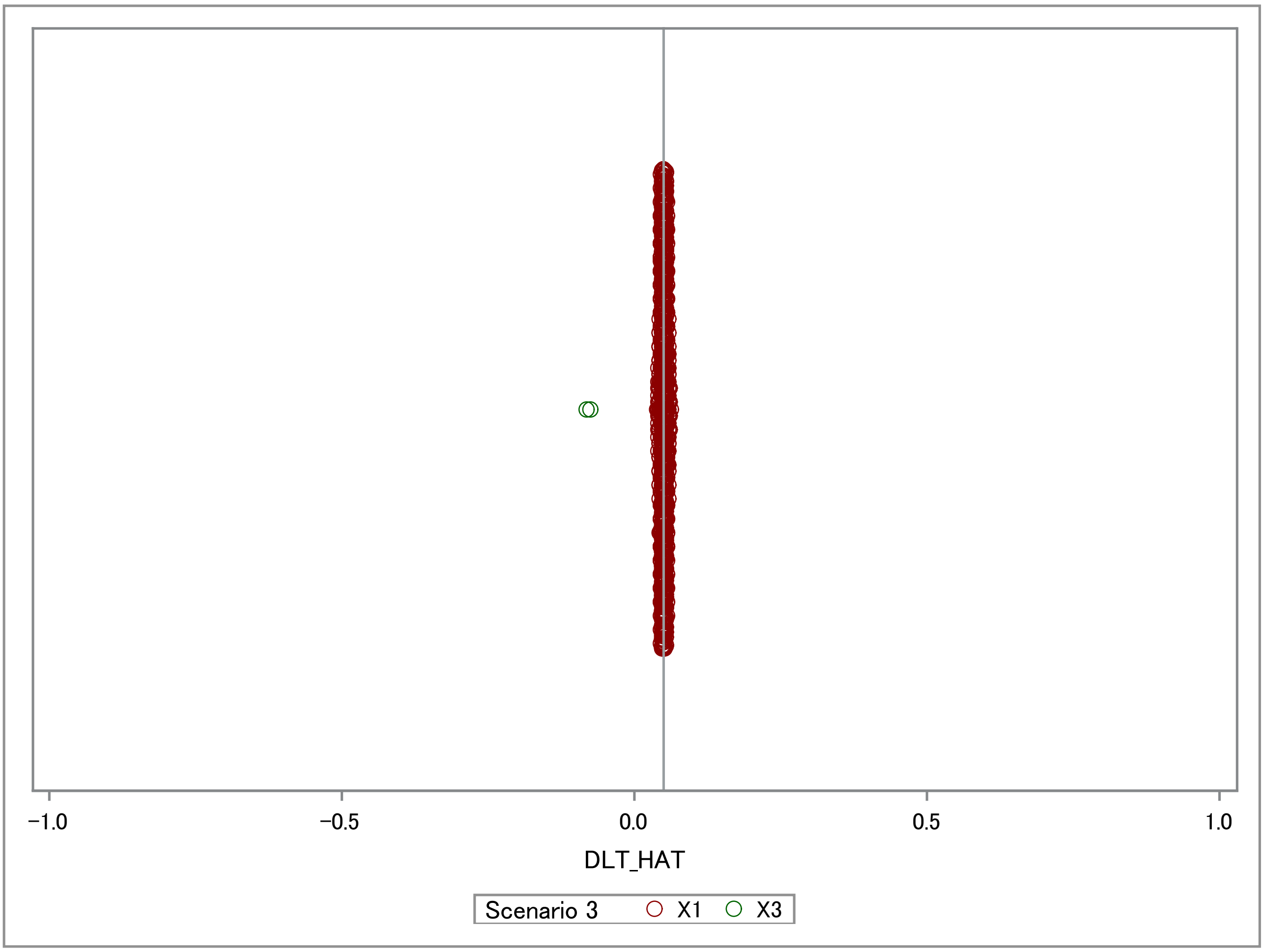
−1.0
−0.5
0.0
0.5
1.0
DLT_HAT
Scenario 3
X1
X3

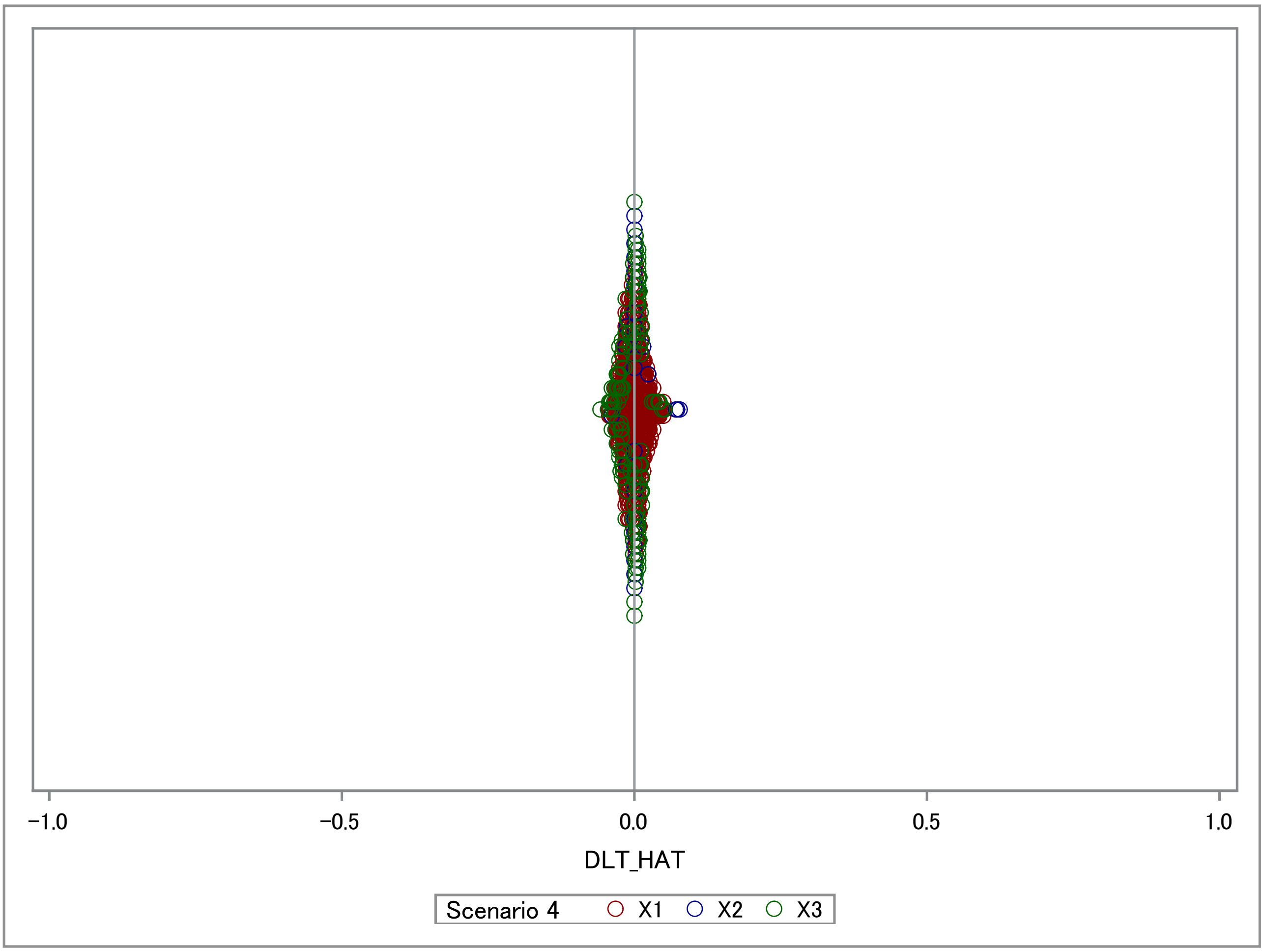

−1.0
−0.5
0.0
0.5
1.0
DLT_HAT
Scenario 4
X1
X2
X3

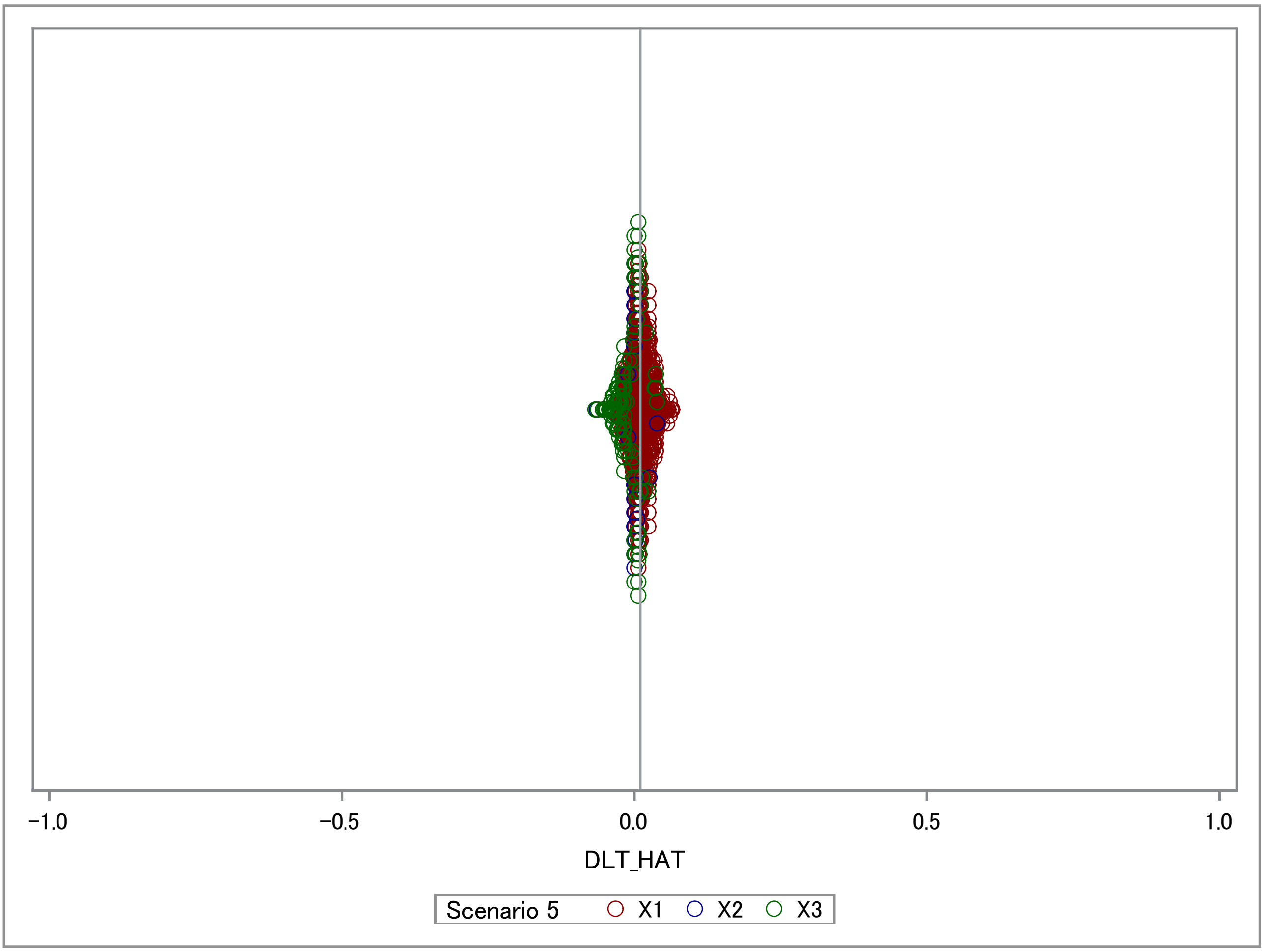

−1.0
−0.5
0.0
0.5
1.0
DLT_HAT
Scenario 5
X1
X2
X3

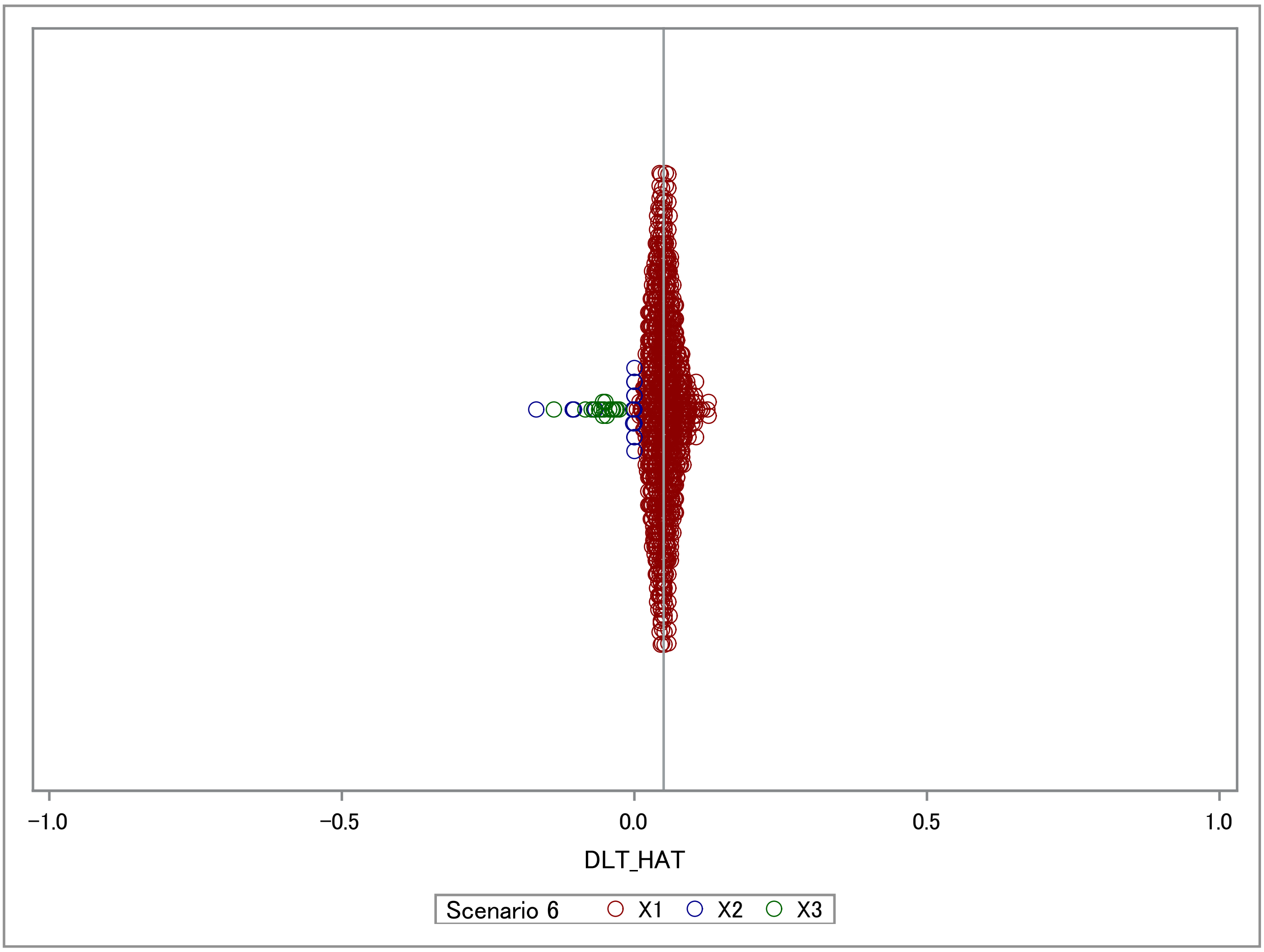
−1.0
−0.5
0.0
0.5
1.0
DLT_HAT
Scenario 6
X1
X2
X3

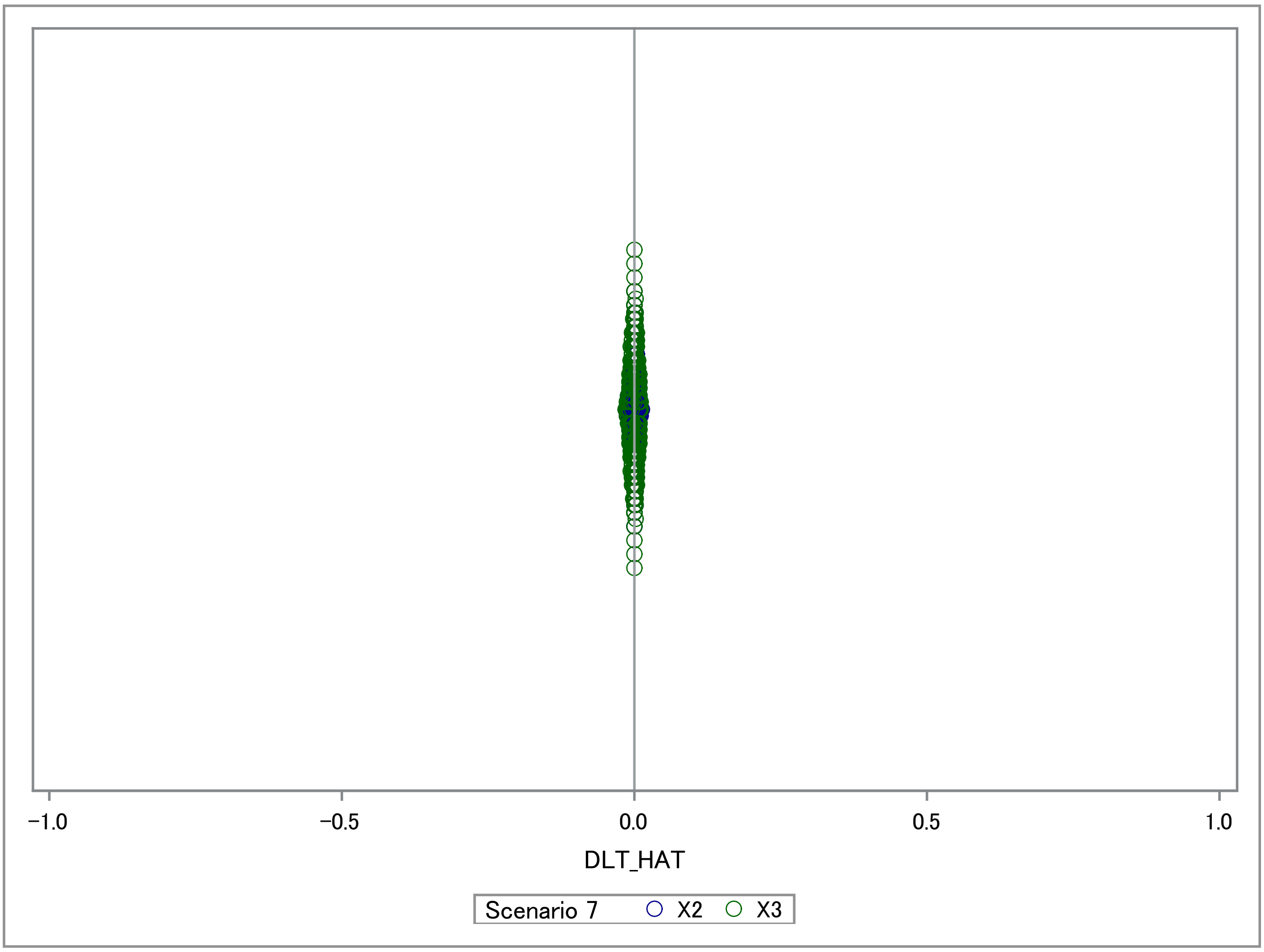
−1.0
−0.5
0.0
0.5
1.0
DLT_HAT
Scenario 7
X2
X3

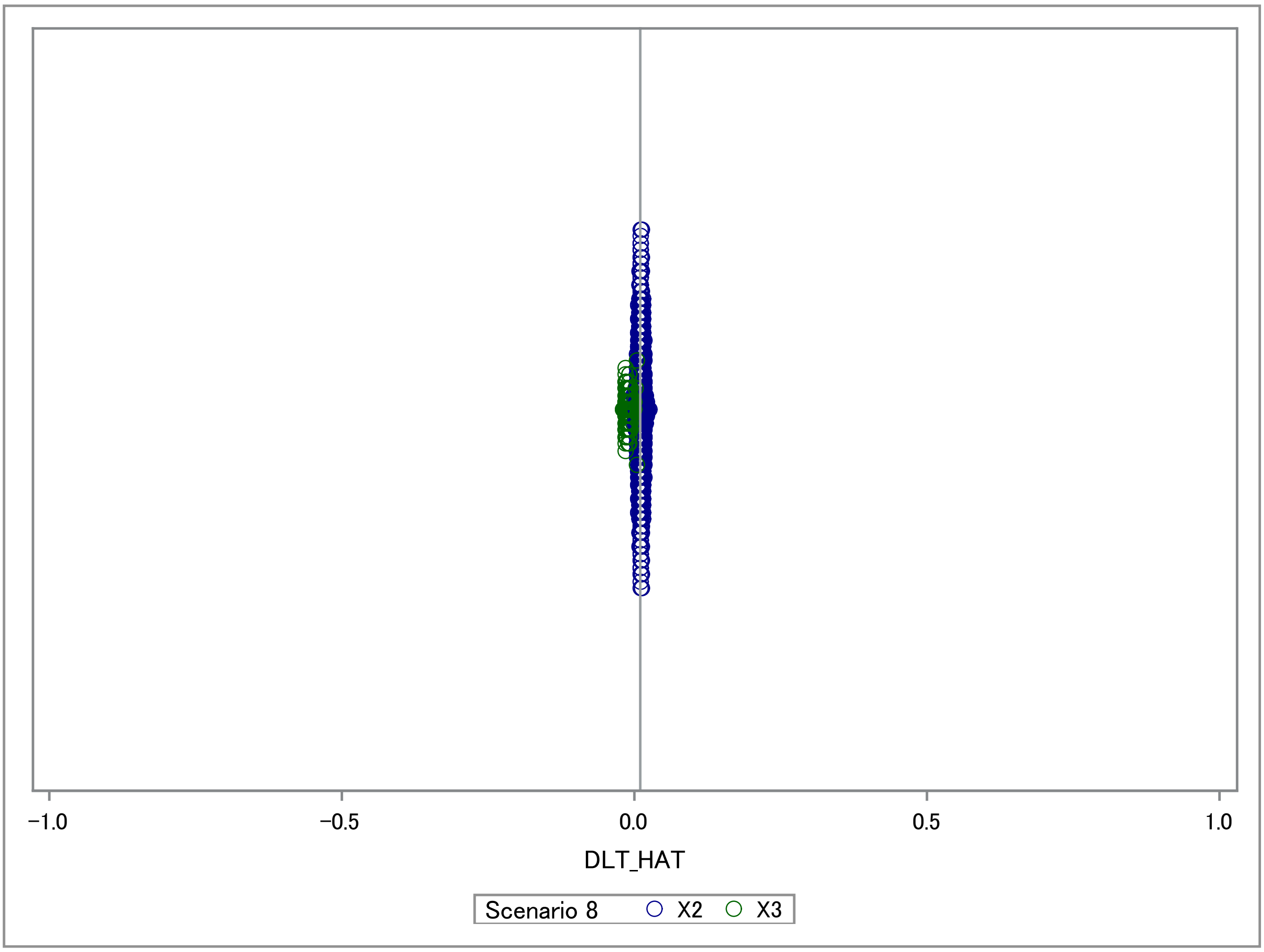
−1.0
−0.5
0.0
0.5
1.0
DLT_HAT
Scenario 8
X2
X3

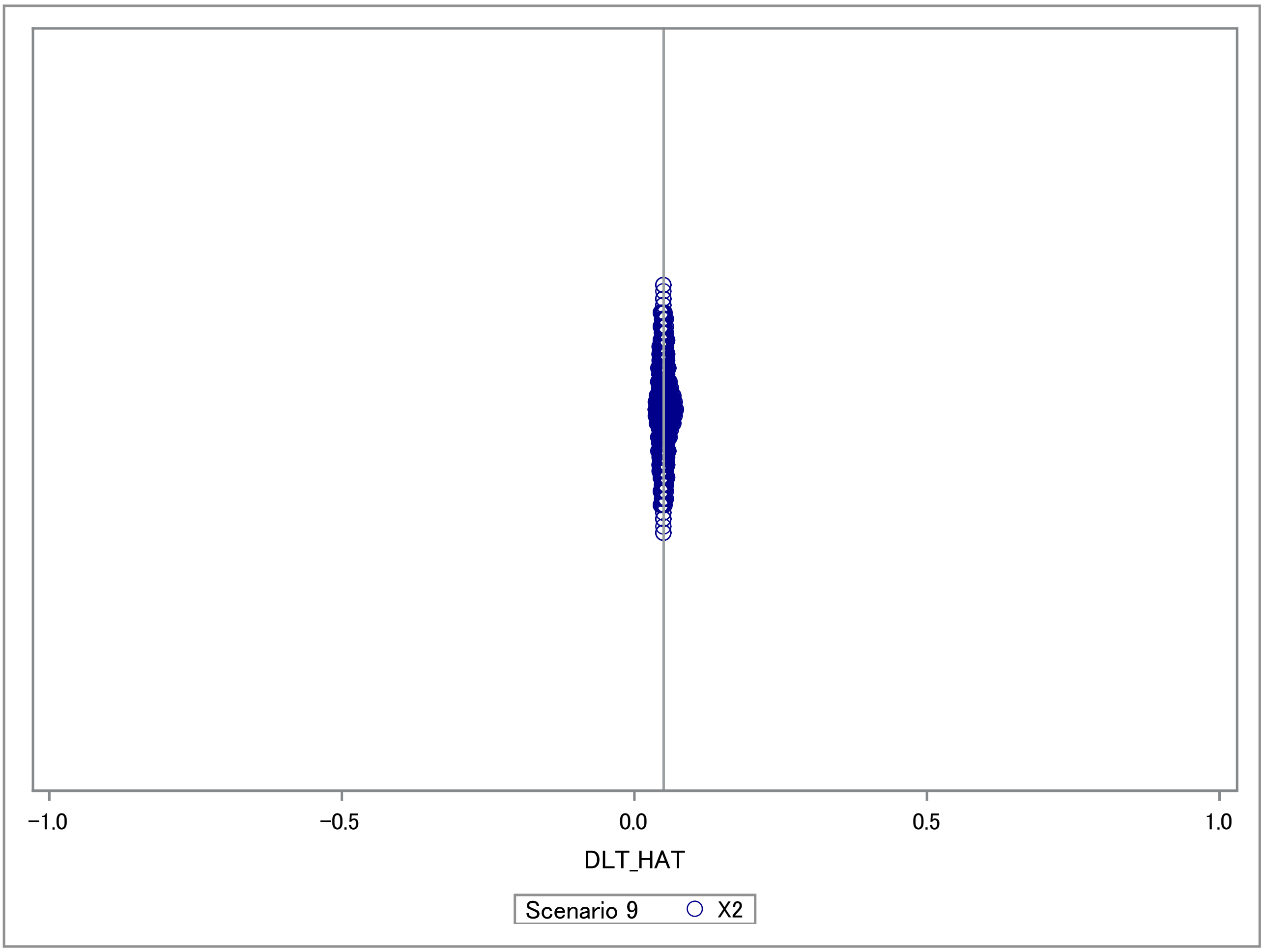
-1.0
-0.5
0.0
0.5
1.0
DLT_HAT
Scenario 9
X2

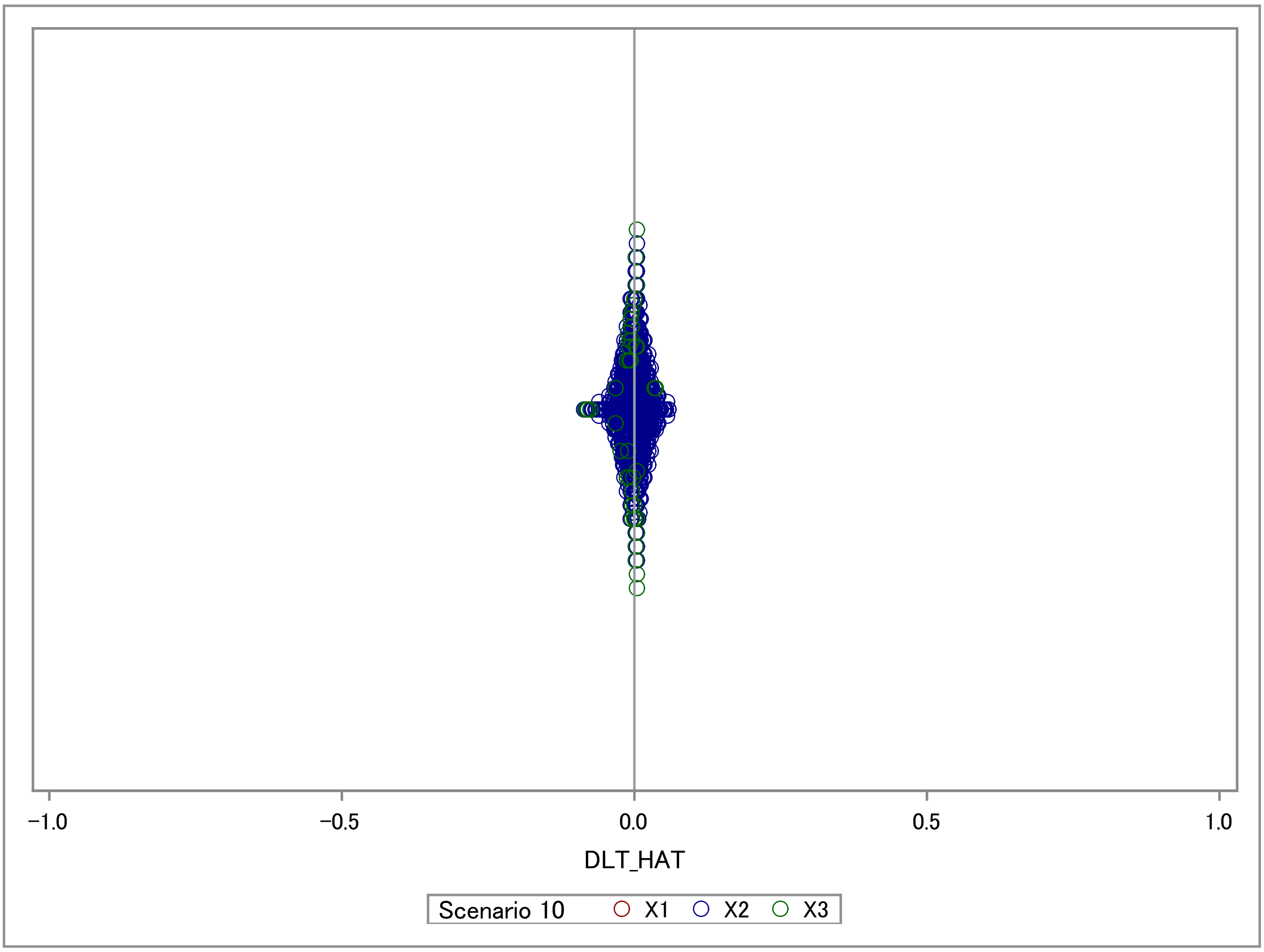
−1.0
−0.5
0.0
0.5
1.0
DLT_HAT
Scenario 10
X1
X2
X3

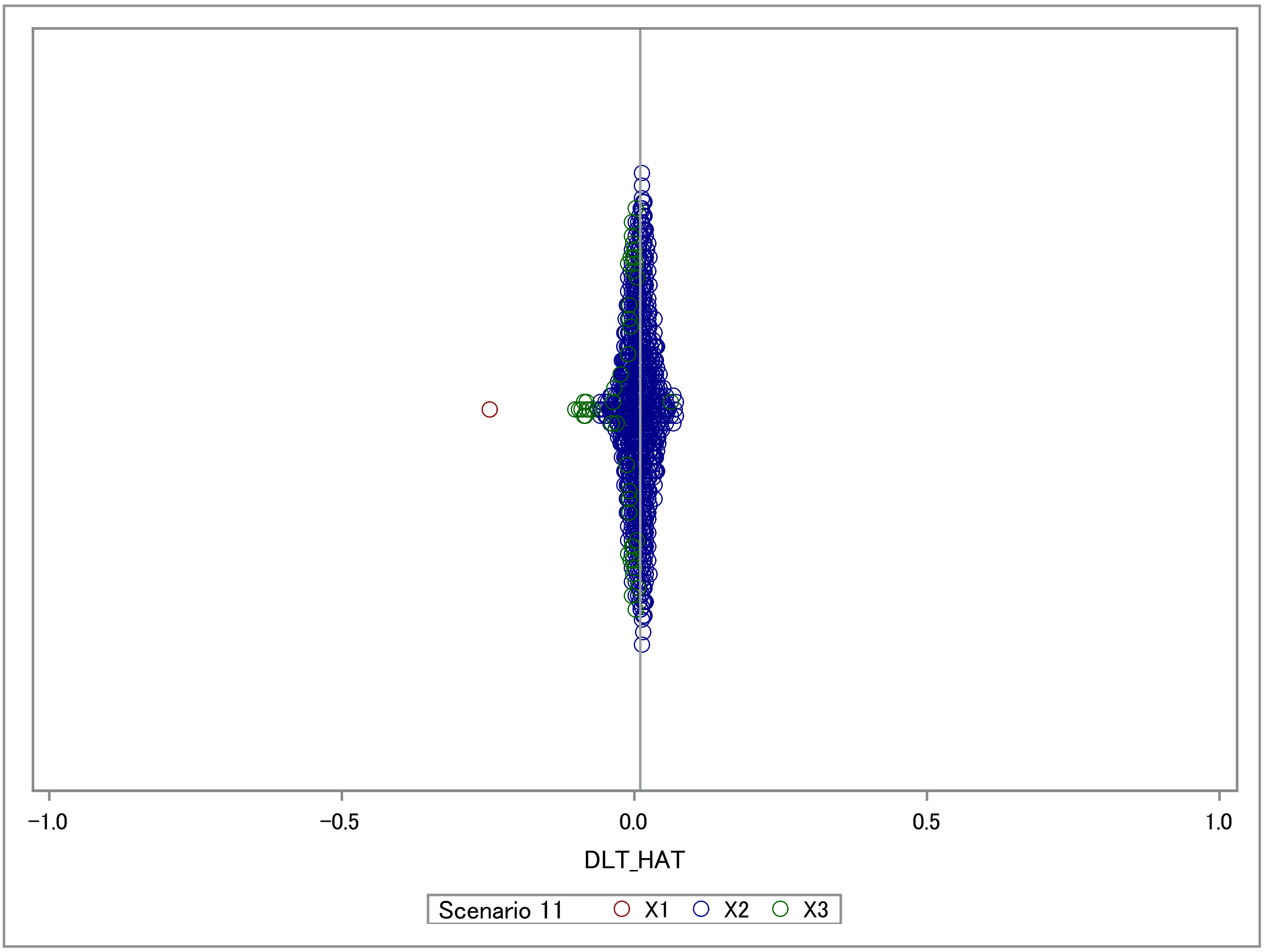
−1.0
−0.5
0.0
0.5
1.0
DLT_HAT
Scenario 11
X1
X2
X3

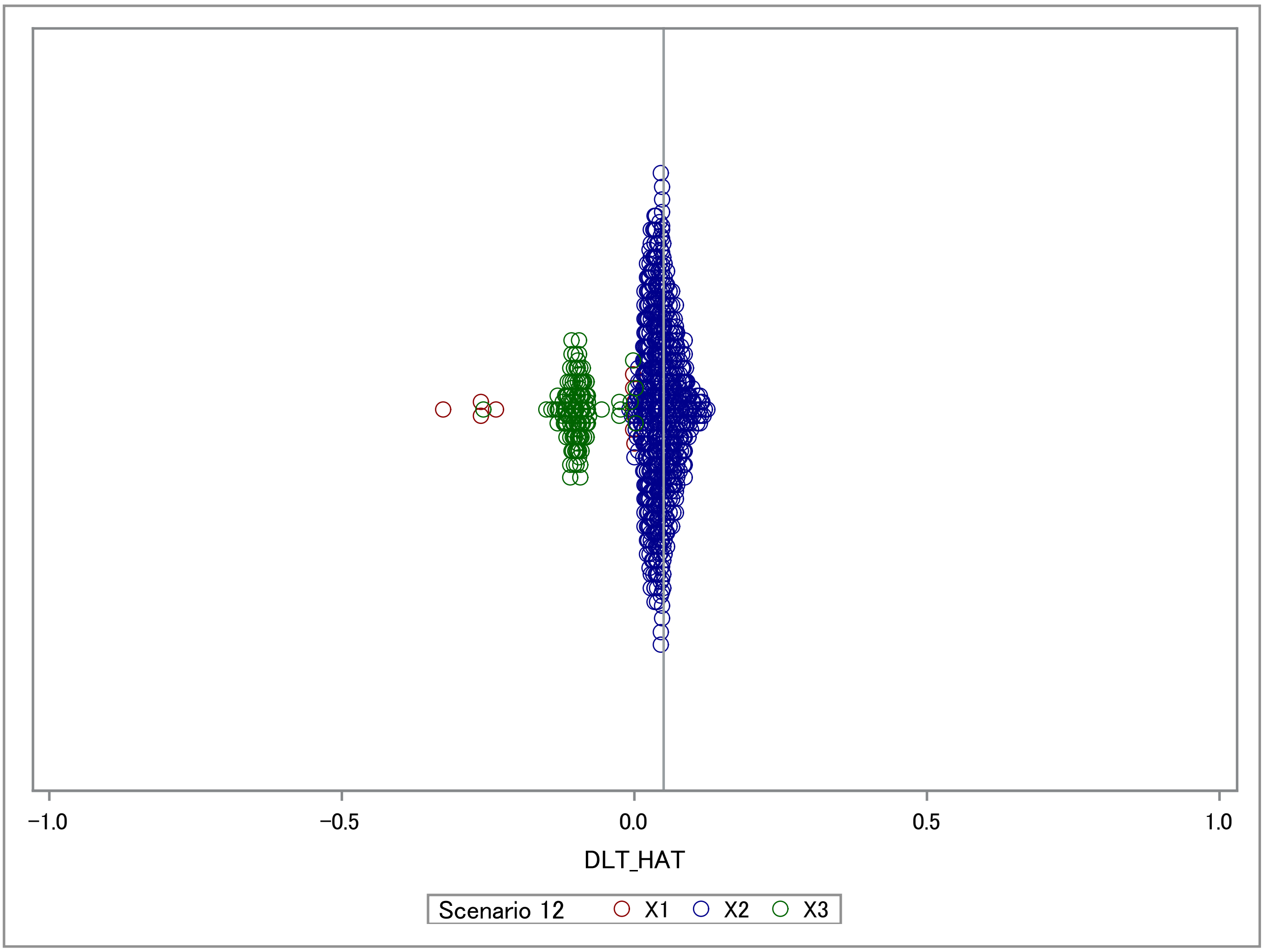

−1.0
−0.5
0.0
0.5
1.0
DLT_HAT
Scenario 12
X1
X2
X3

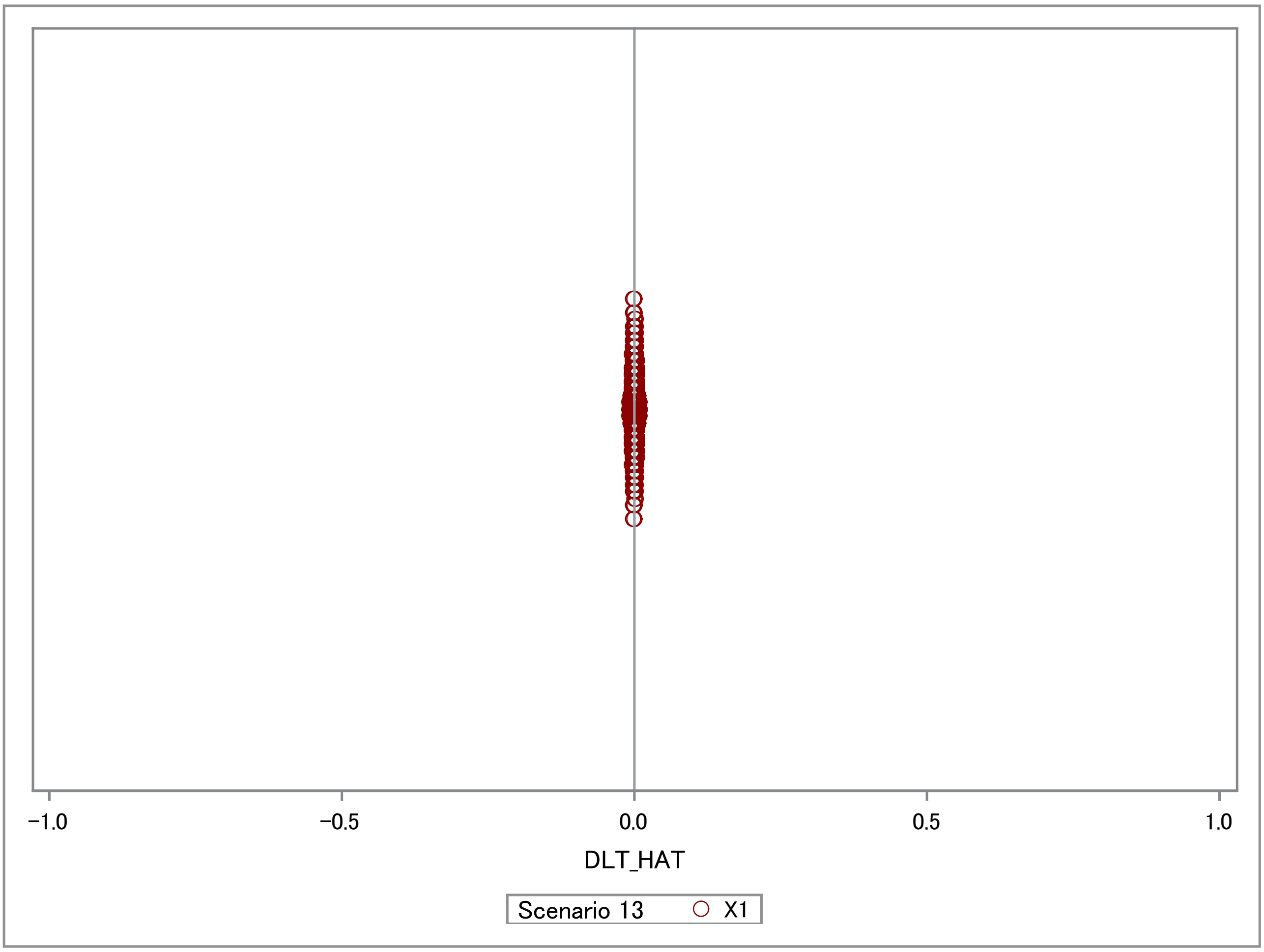
−1.0
−0.5
0.0
0.5
1.0
DLT_HAT
Scenario 13
X1

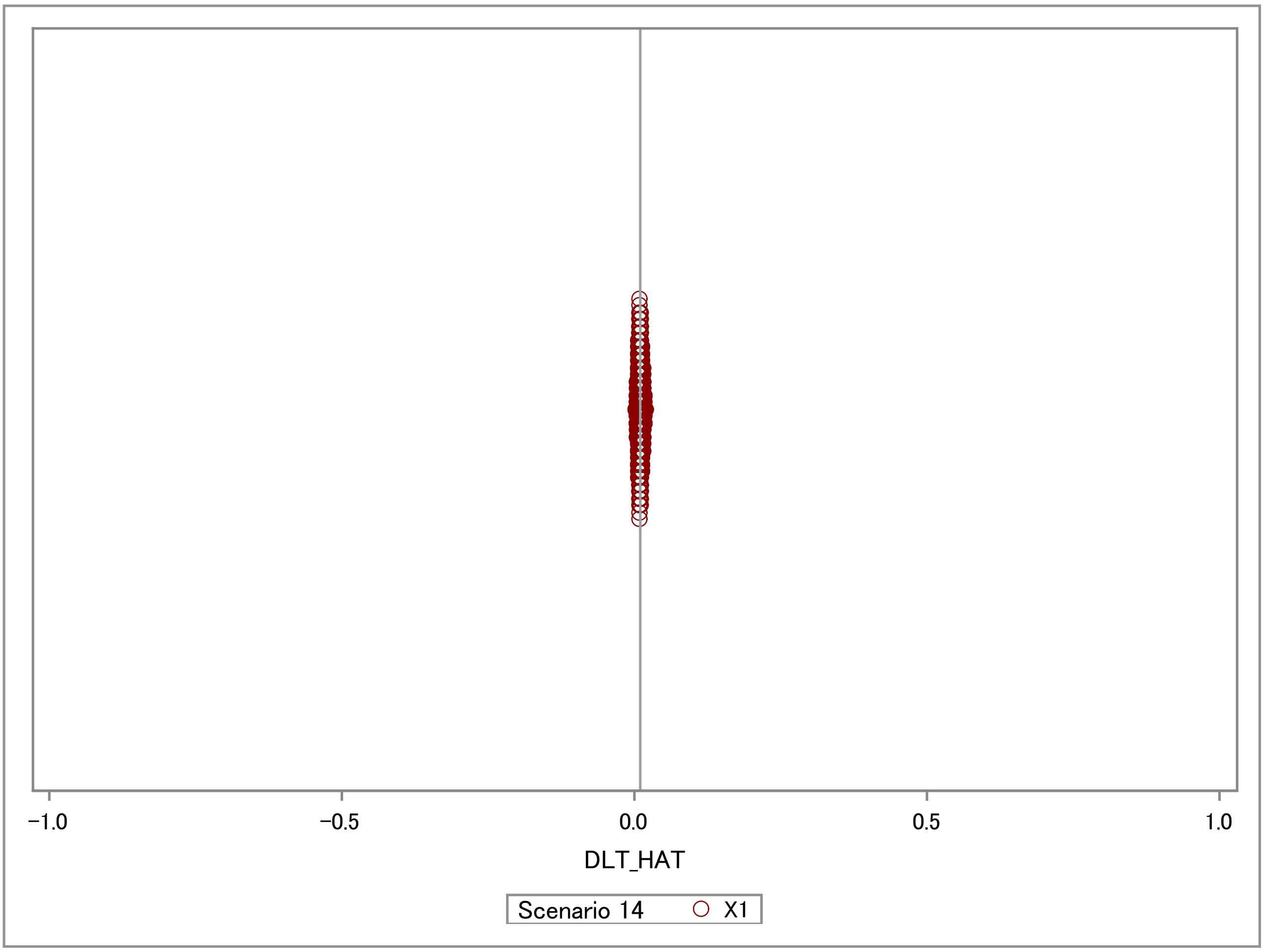

−1.0
−0.5
0.0
0.5
1.0
DLT_HAT
Scenario 14
X1

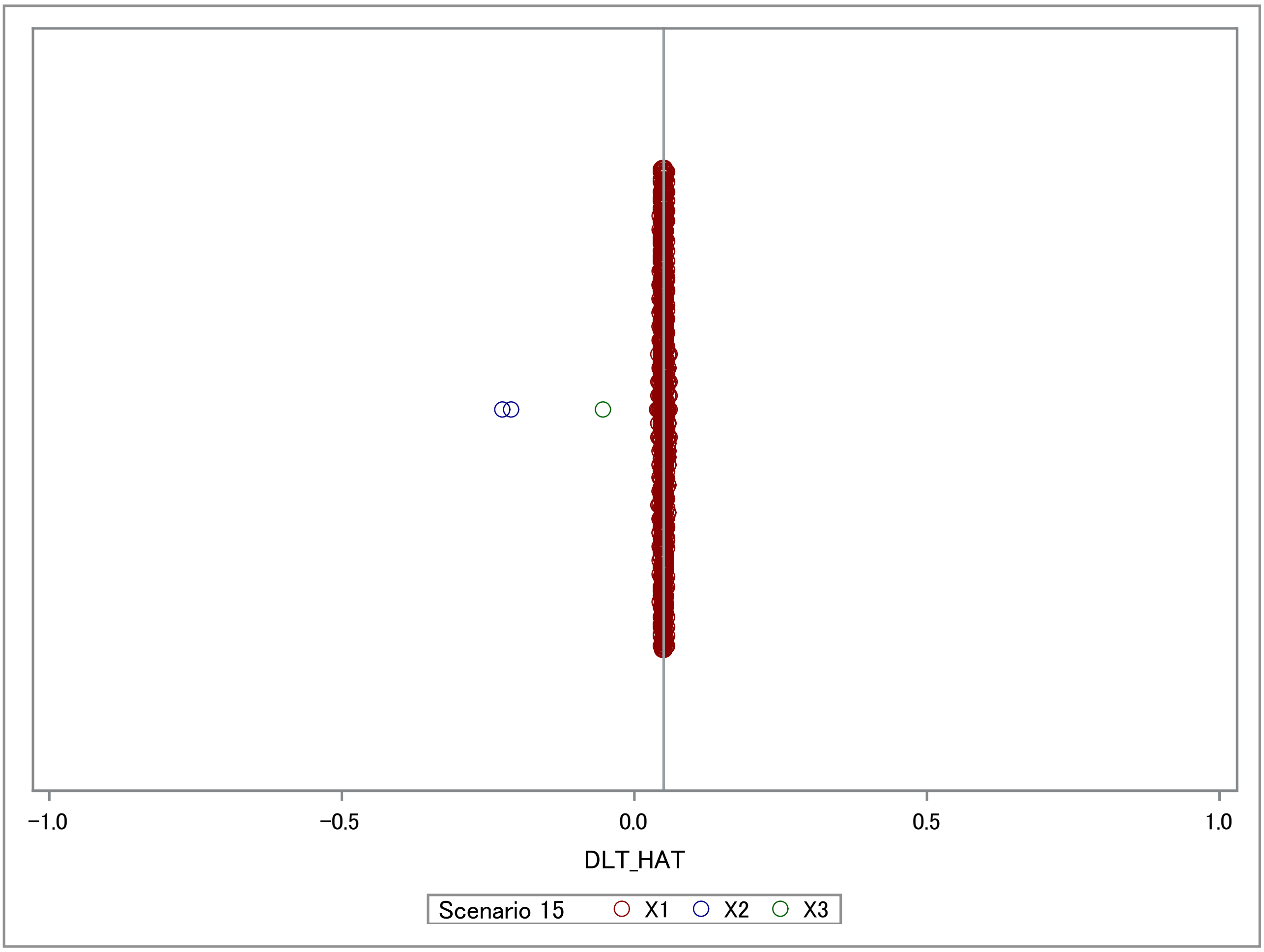
−1.0
−0.5
0.0
0.5
1.0
DLT_HAT
Scenario 15
X1
X2
X3

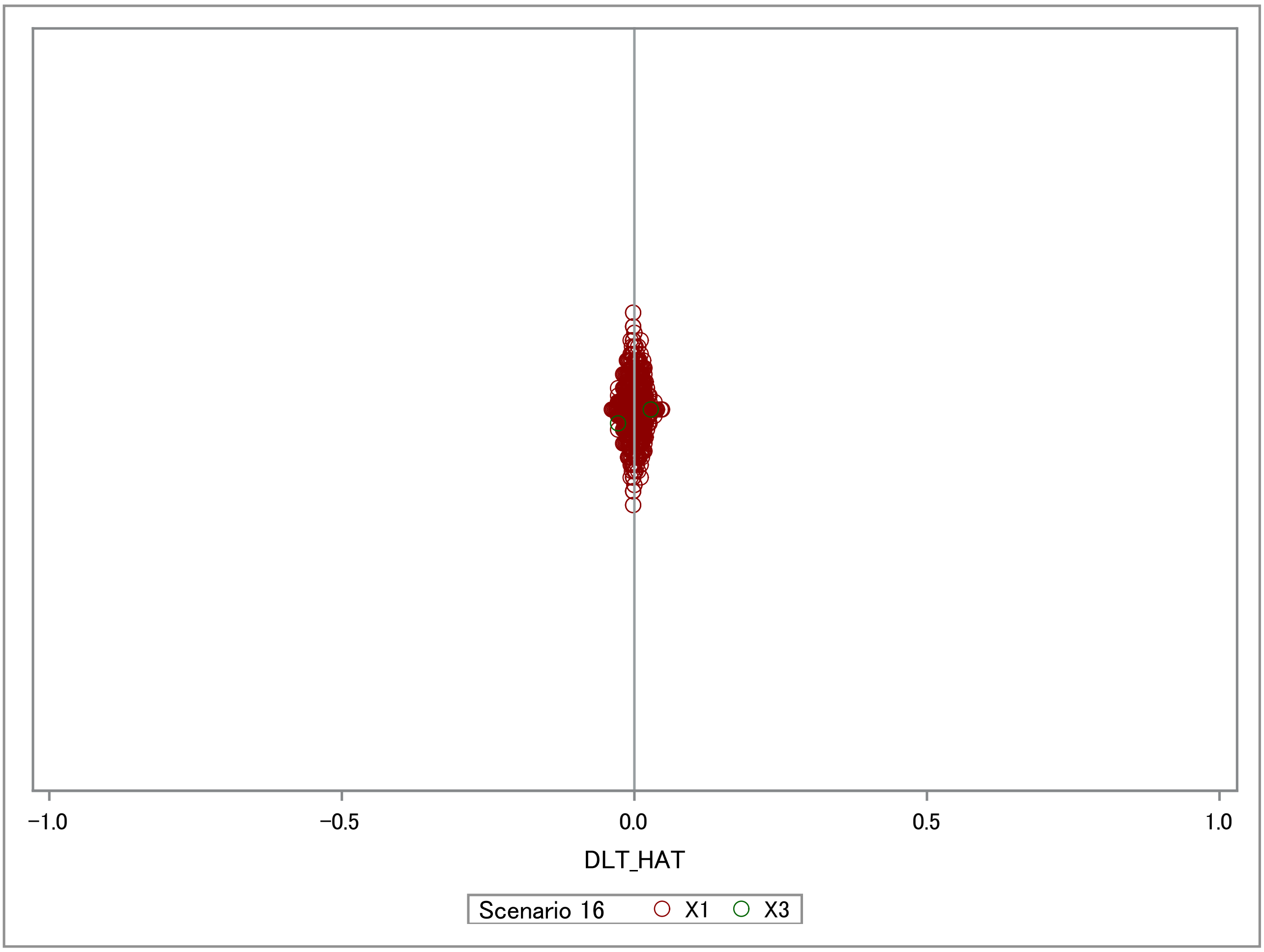

−1.0
−0.5
0.0
0.5
1.0
DLT_HAT
Scenario 16
X1
X3

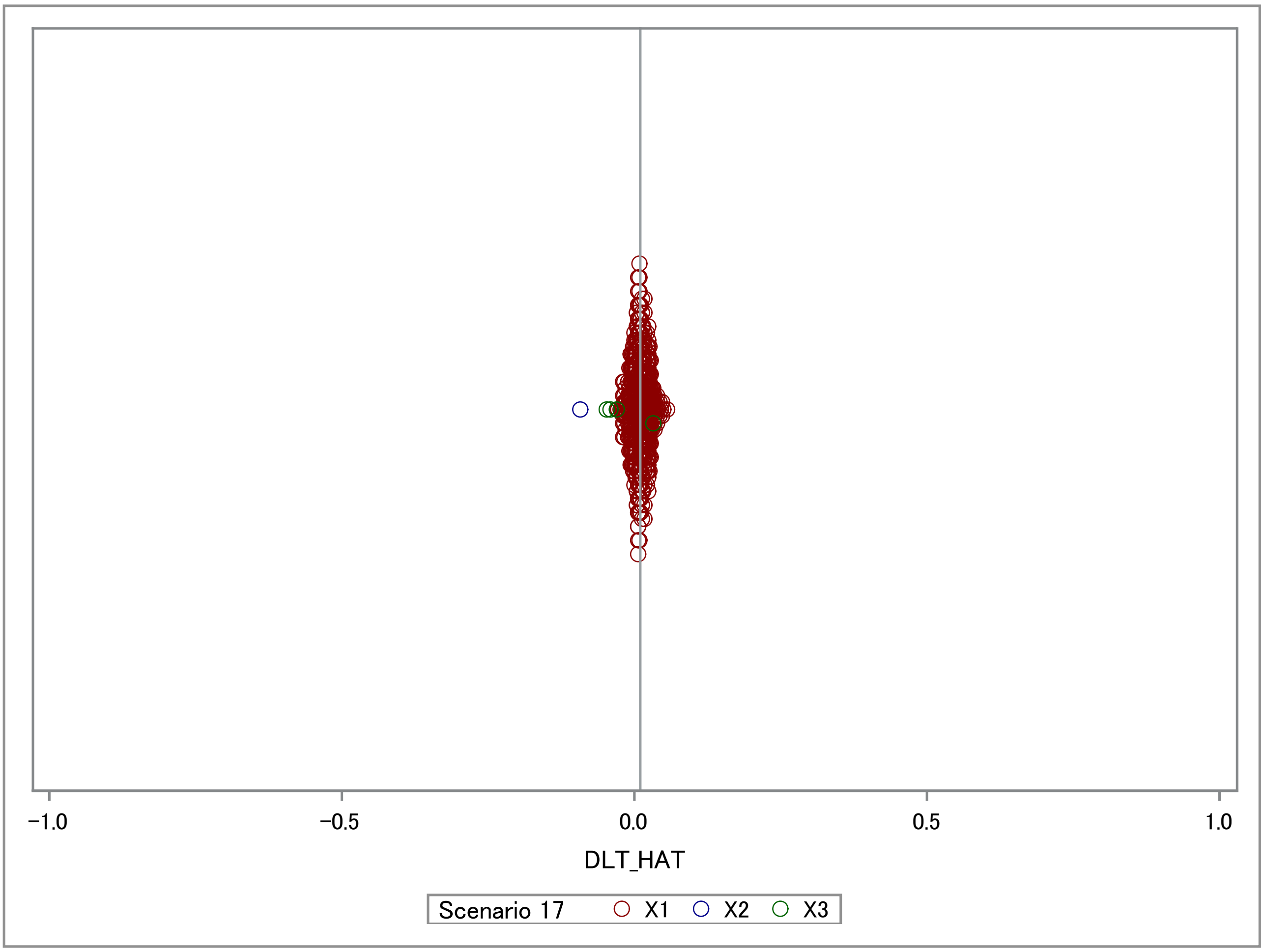

−1.0
−0.5
0.0
0.5
1.0
DLT_HAT
Scenario 17
X1
X2
X3

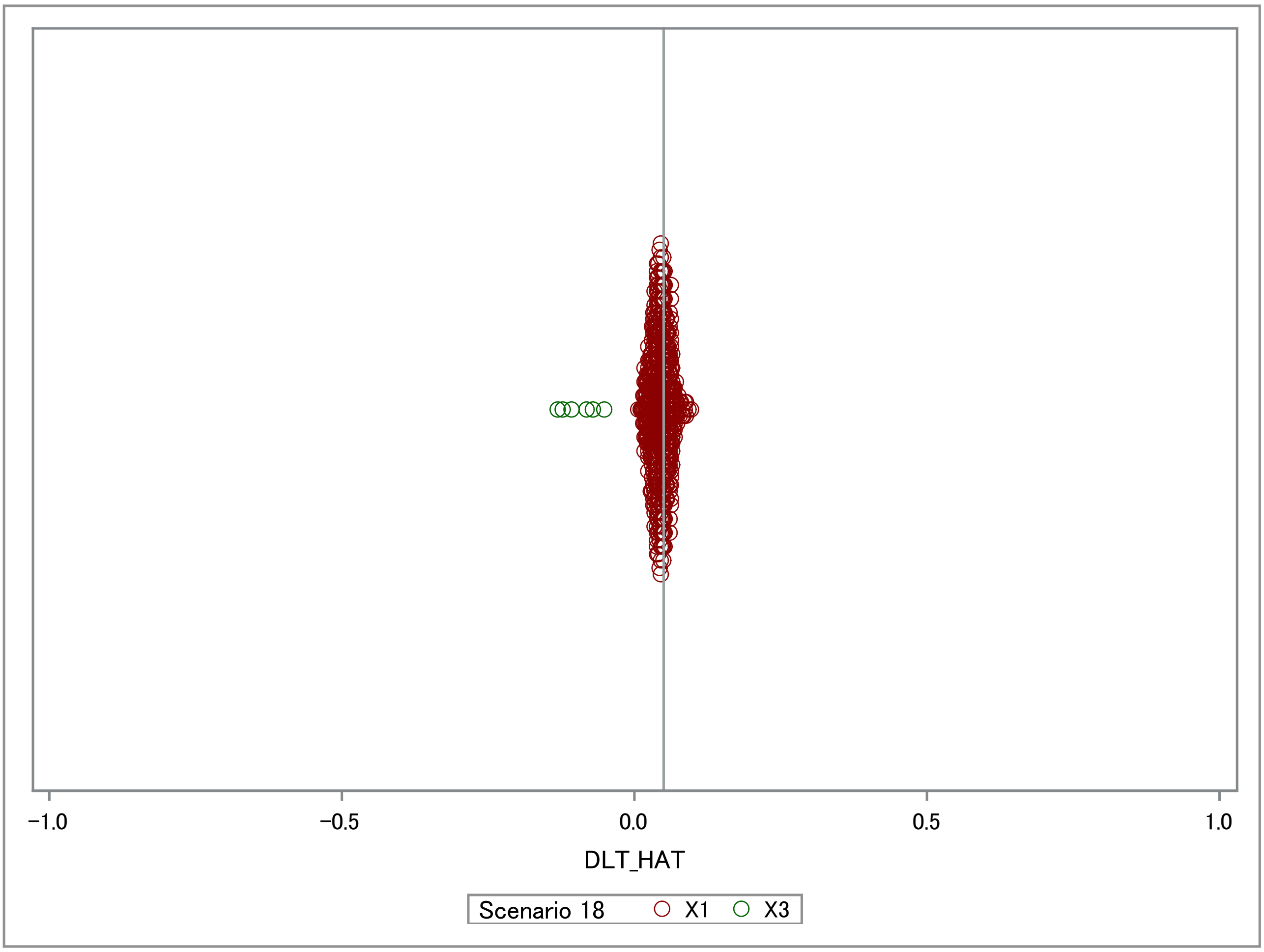
−1.0
−0.5
0.0
0.5
1.0
DLT_HAT
Scenario 18
X1
X3

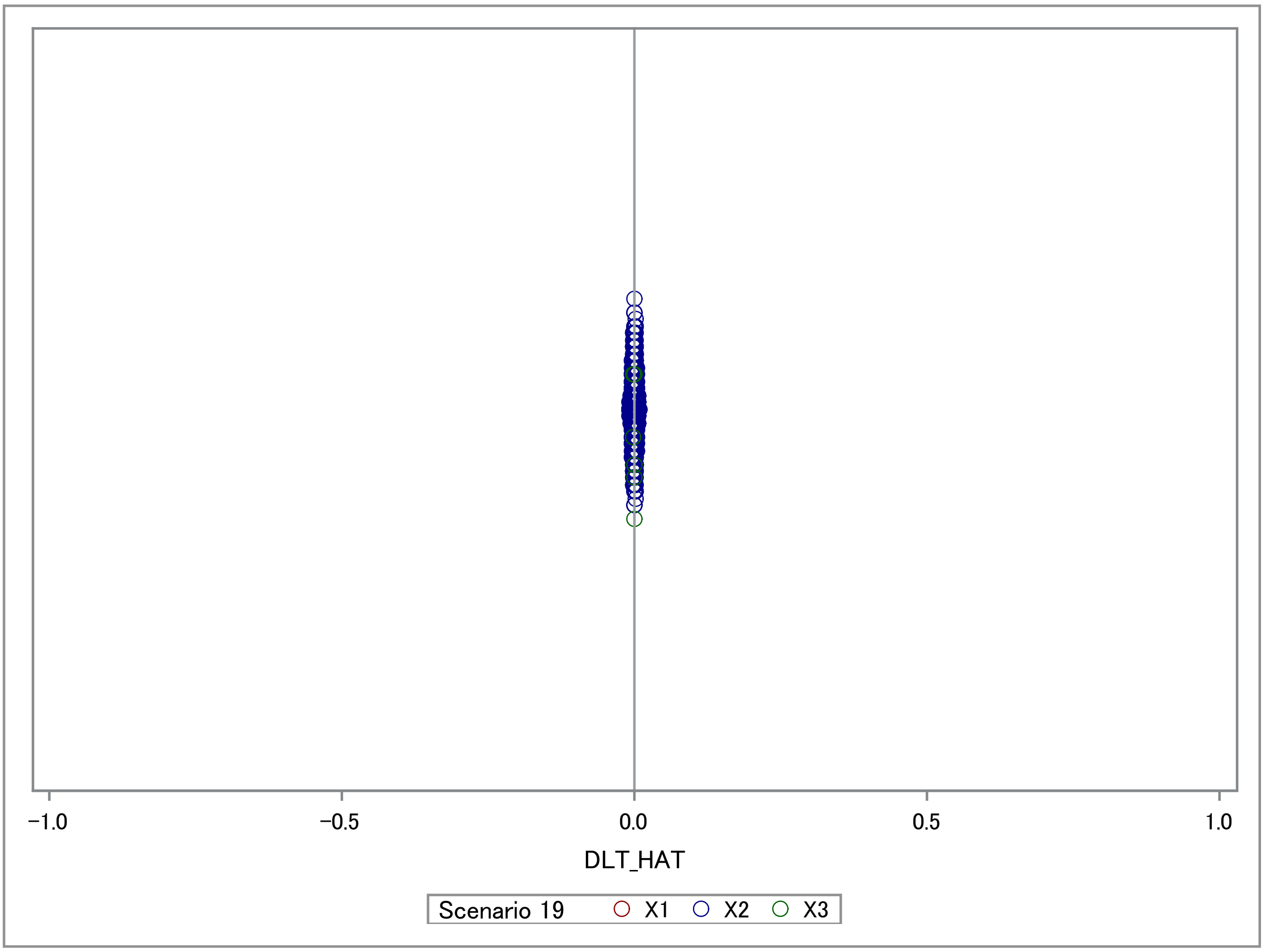
−1.0
−0.5
0.0
0.5
1.0
DLT_HAT
Scenario 19
X1
X2
X3

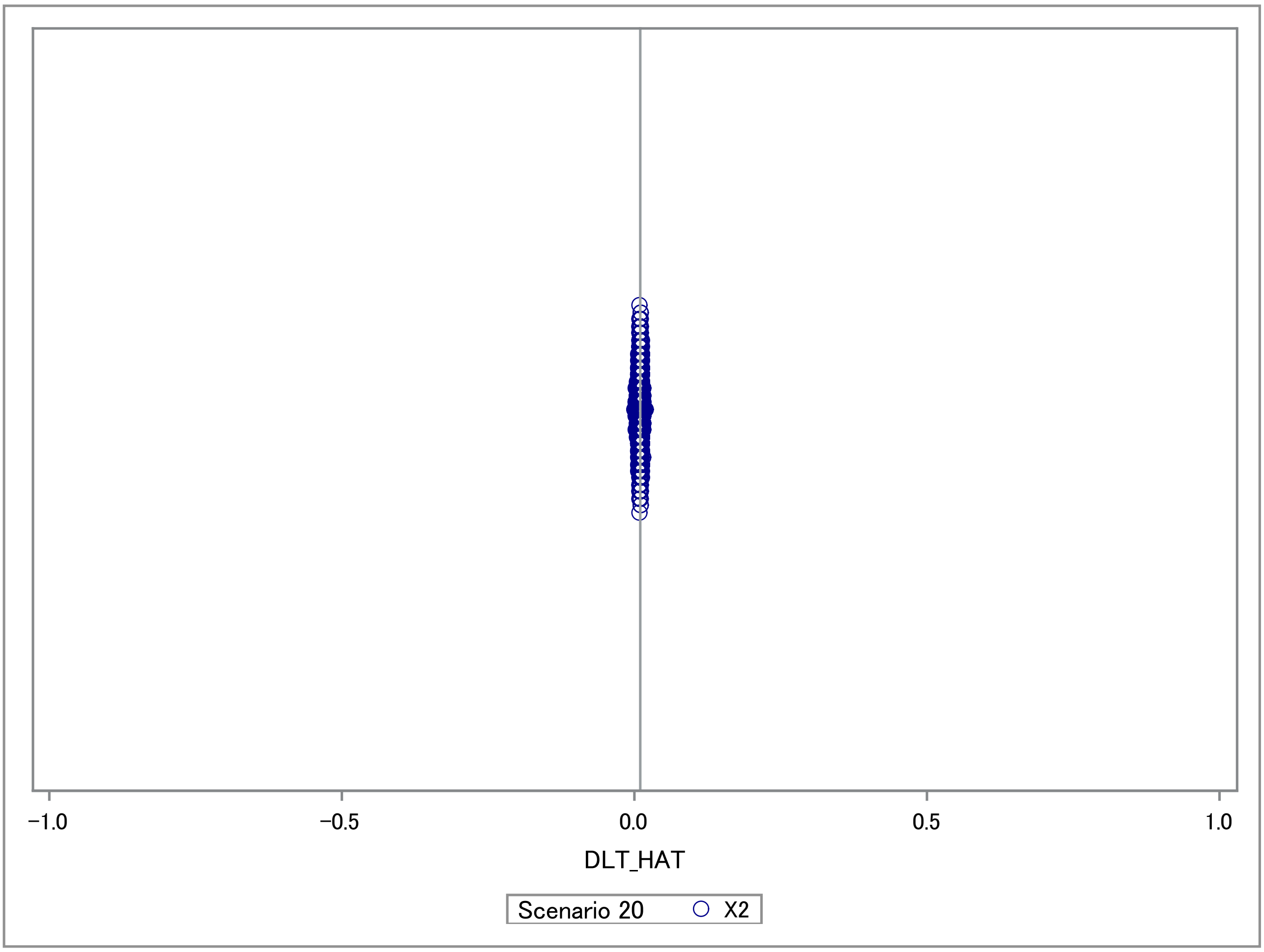
−1.0
−0.5
0.0
0.5
1.0
DLT_HAT
Scenario 20
X2

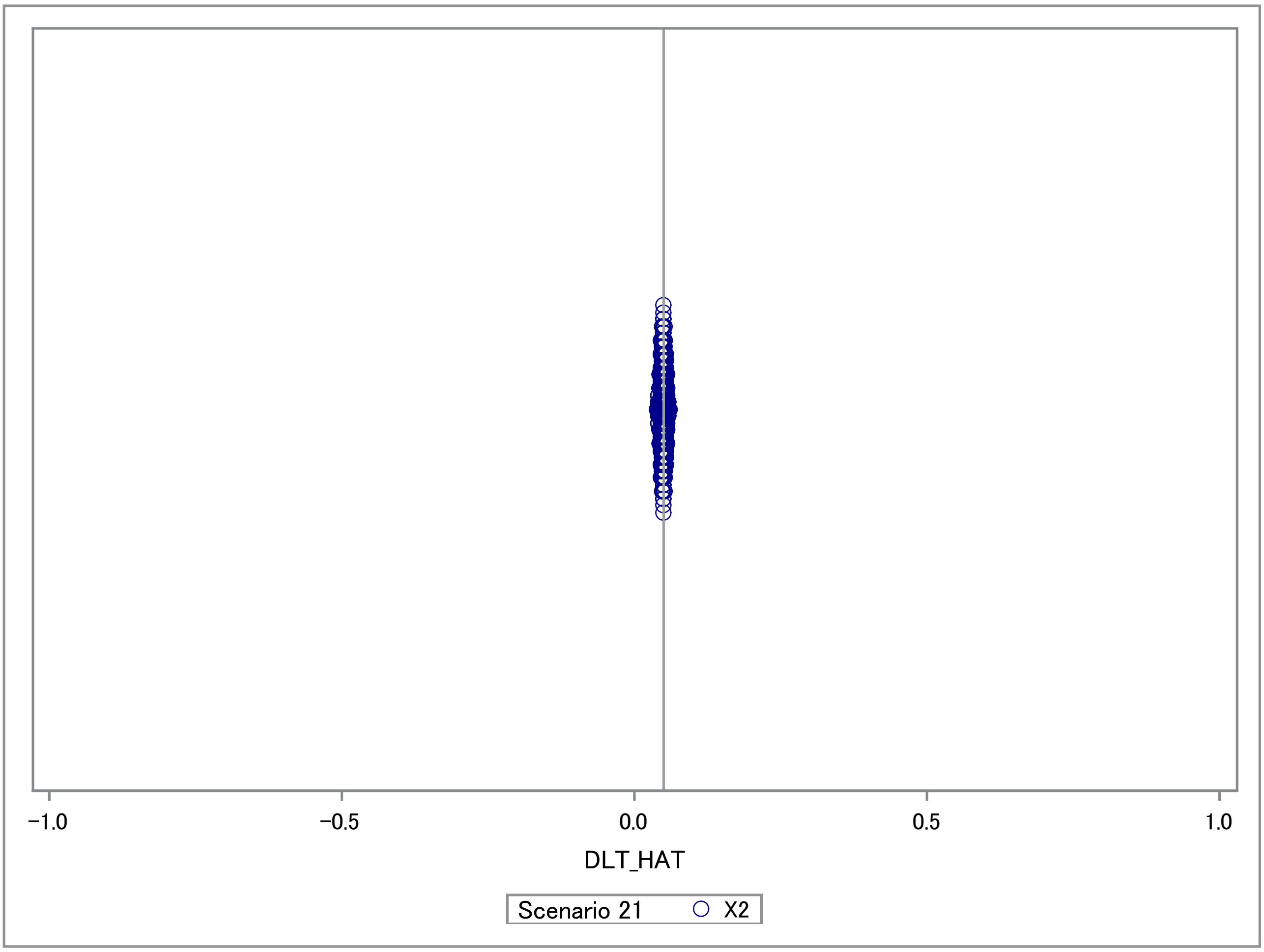

−1.0
−0.5
0.0
0.5
1.0
DLT_HAT
Scenario 21
X2

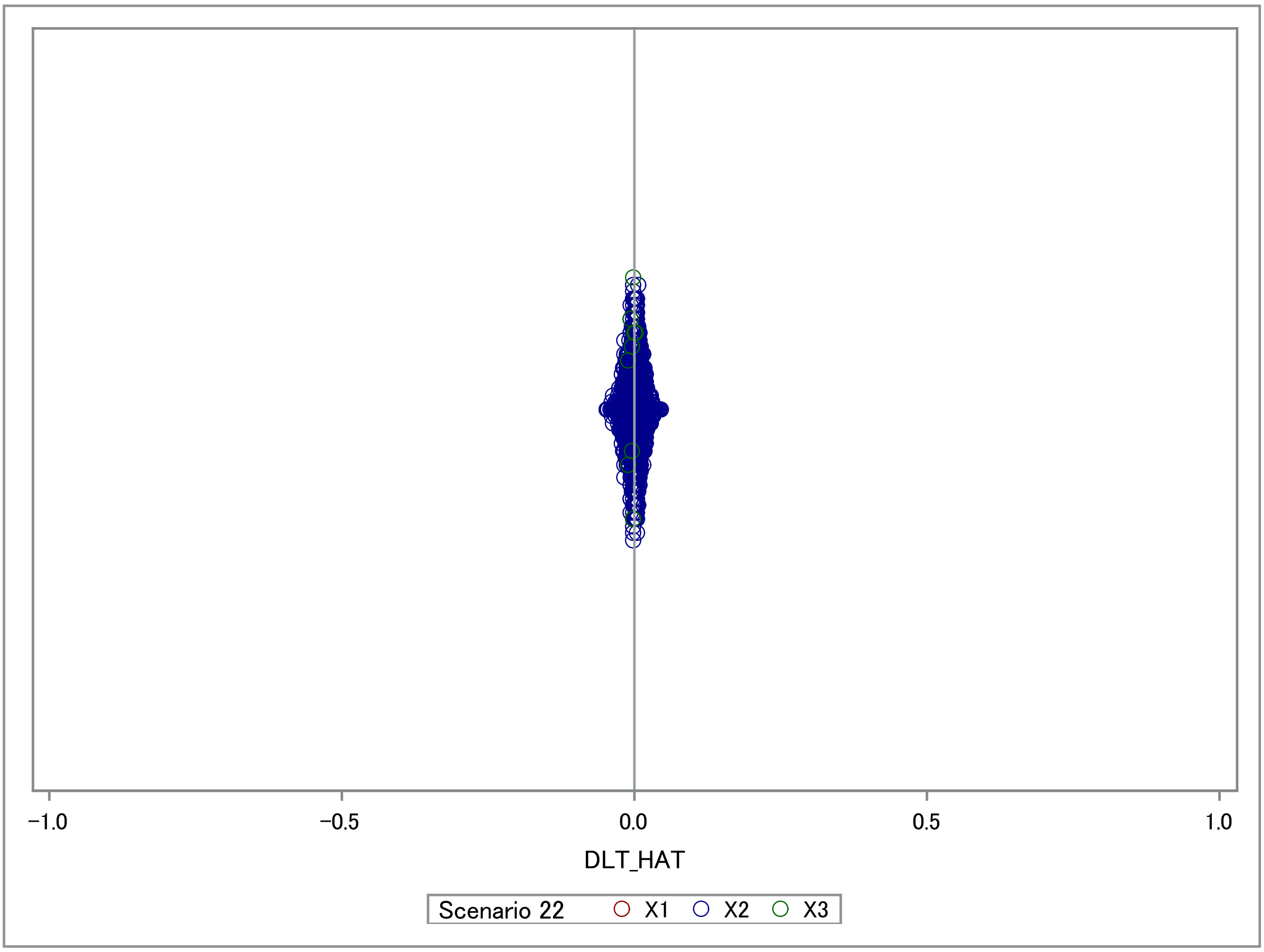
−1.0
−0.5
0.0
0.5
1.0
DLT_HAT
Scenario 22
X1
X2
X3

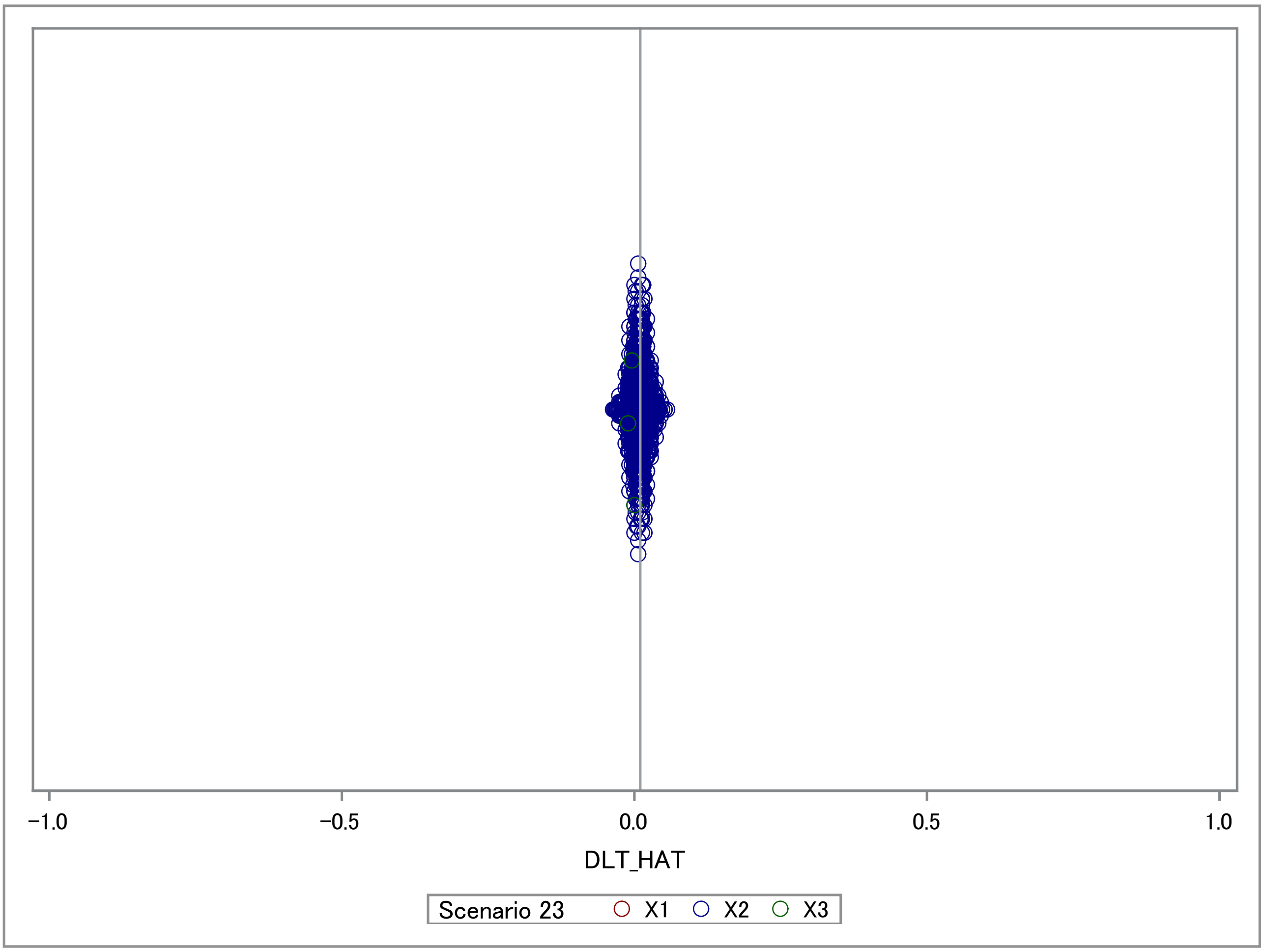

−1.0
−0.5
0.0
0.5
1.0
DLT_HAT
Scenario 23
X1
X2
X3

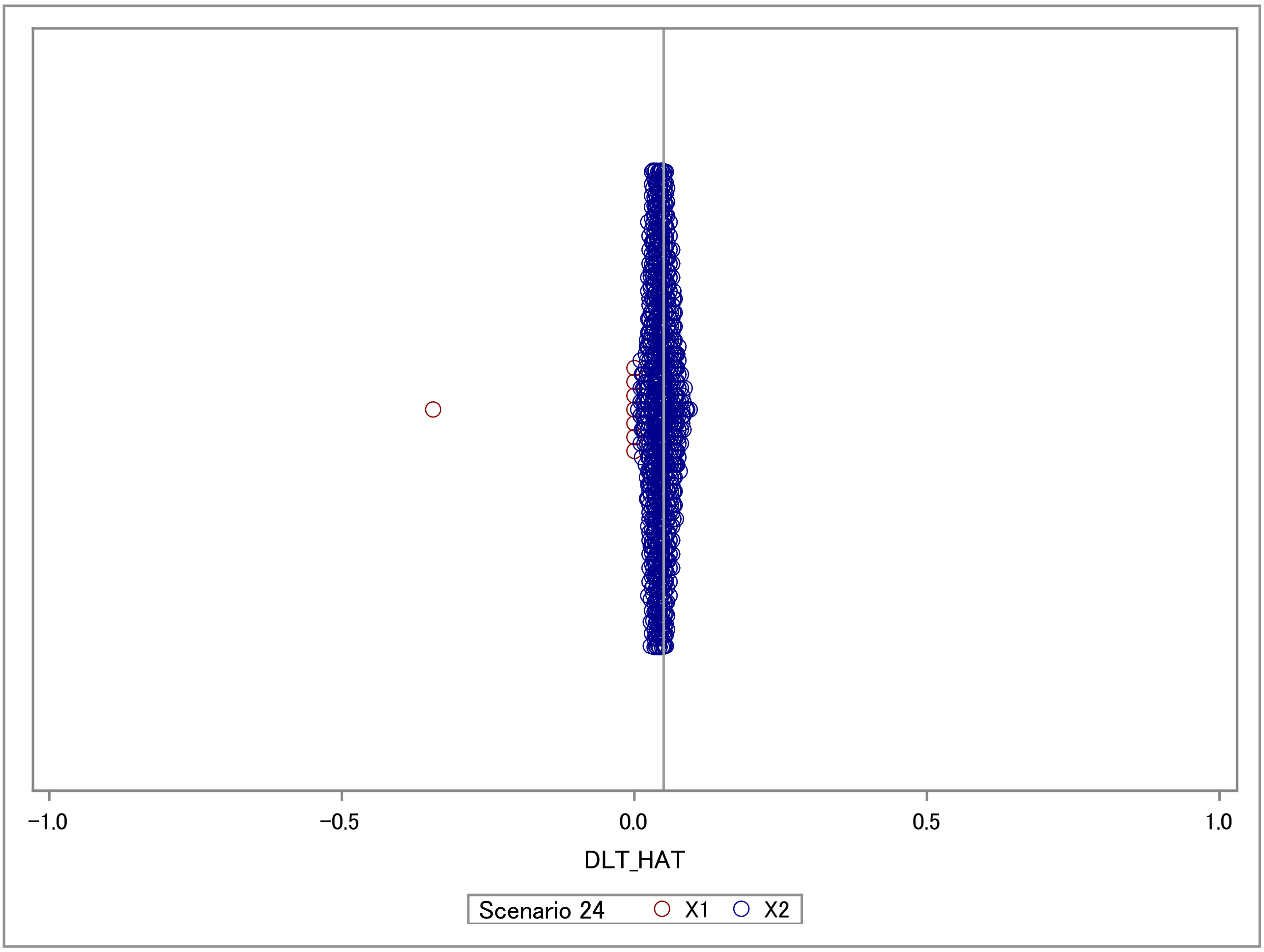
−1.0
−0.5
0.0
0.5
1.0
DLT_HAT
Scenario 24
X1
X2

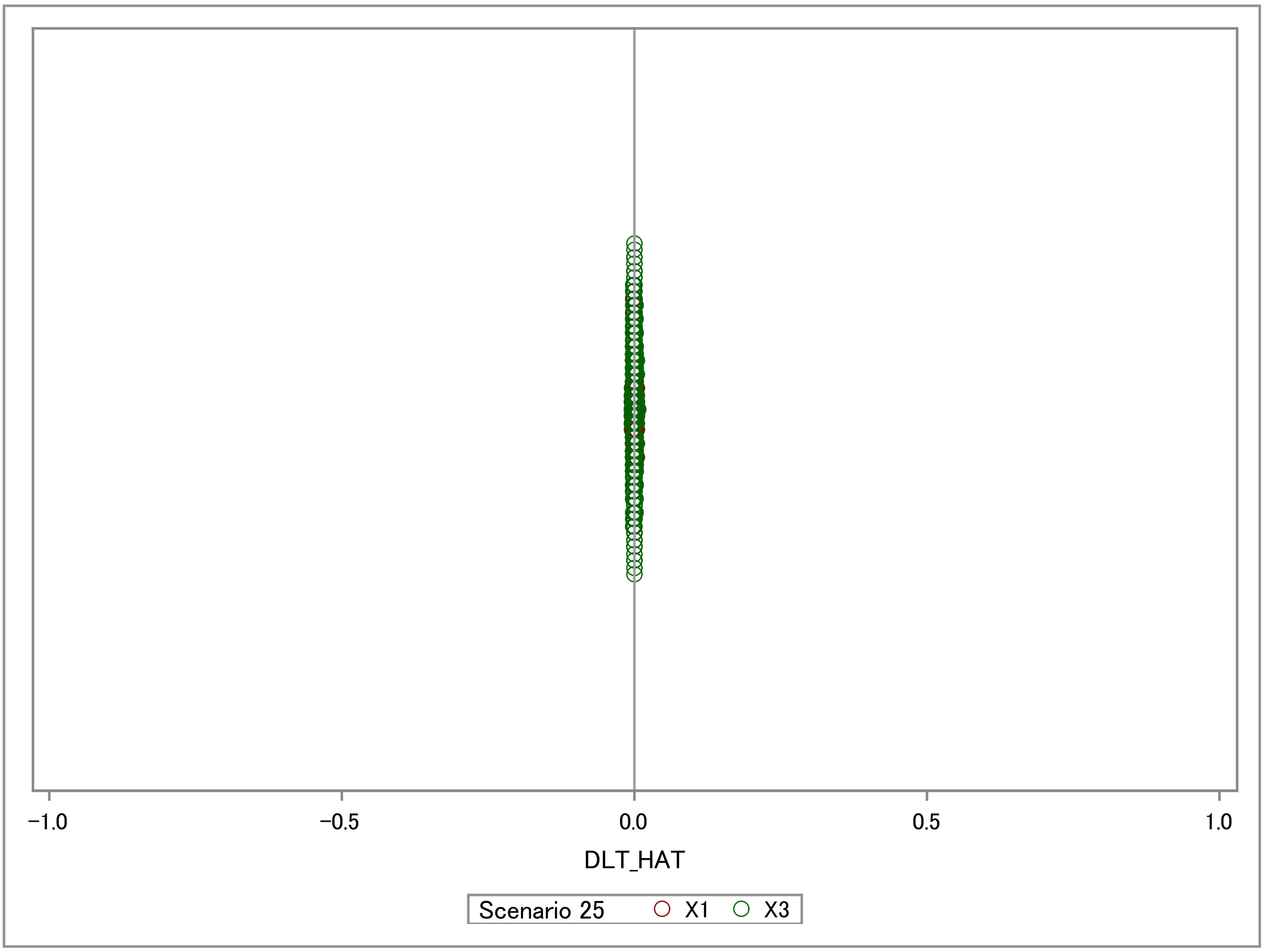

−1.0
−0.5
0.0
0.5
1.0
DLT_HAT
Scenario 25
X1
X3

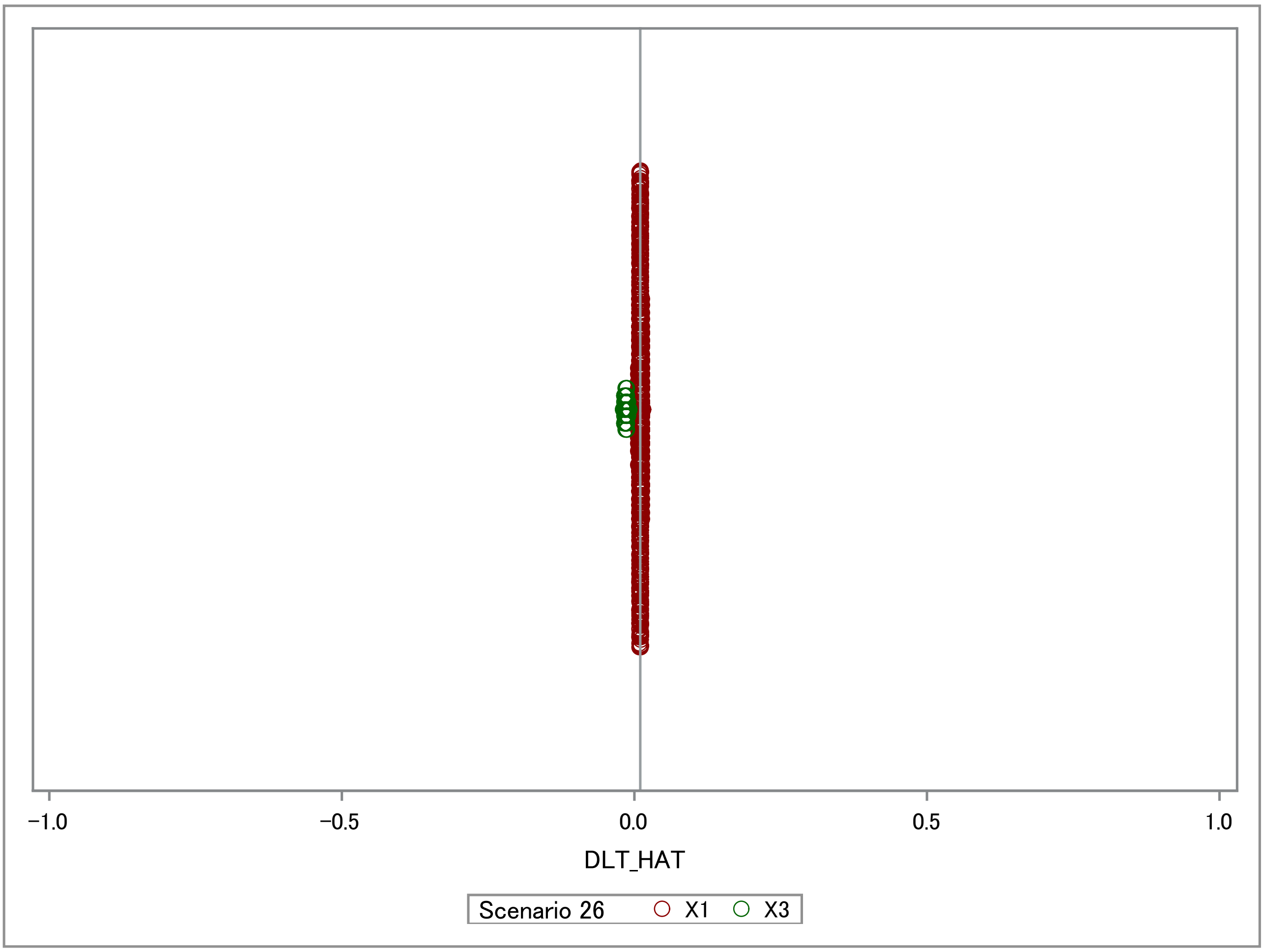
−1.0
−0.5
0.0
0.5
1.0
DLT_HAT
Scenario 26
X1
X3

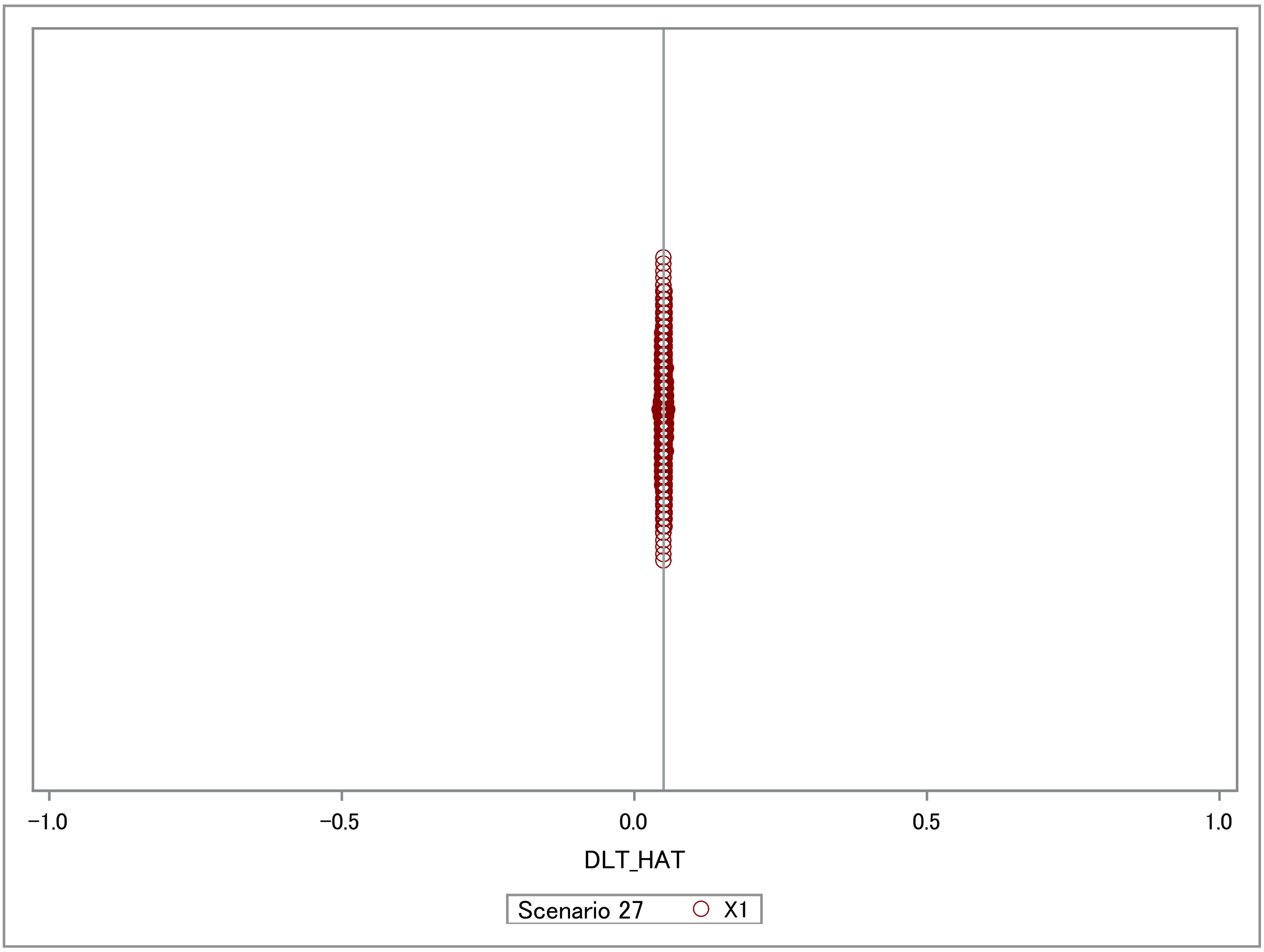

−1.0
−0.5
0.0
0.5
1.0
DLT_HAT
Scenario 27
X1

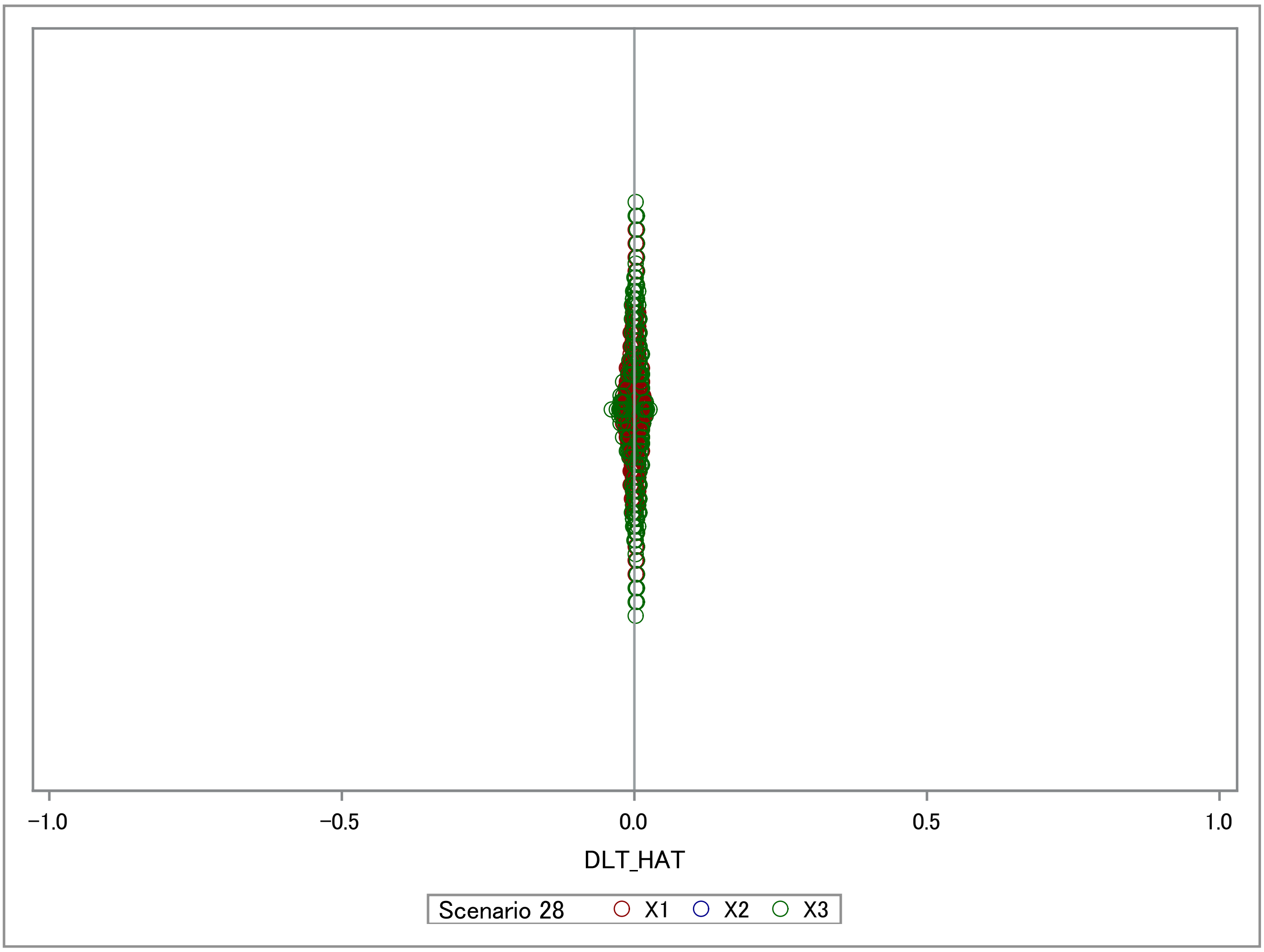
−1.0
−0.5
0.0
0.5
1.0
DLT_HAT
Scenario 28
X1
X2
X3

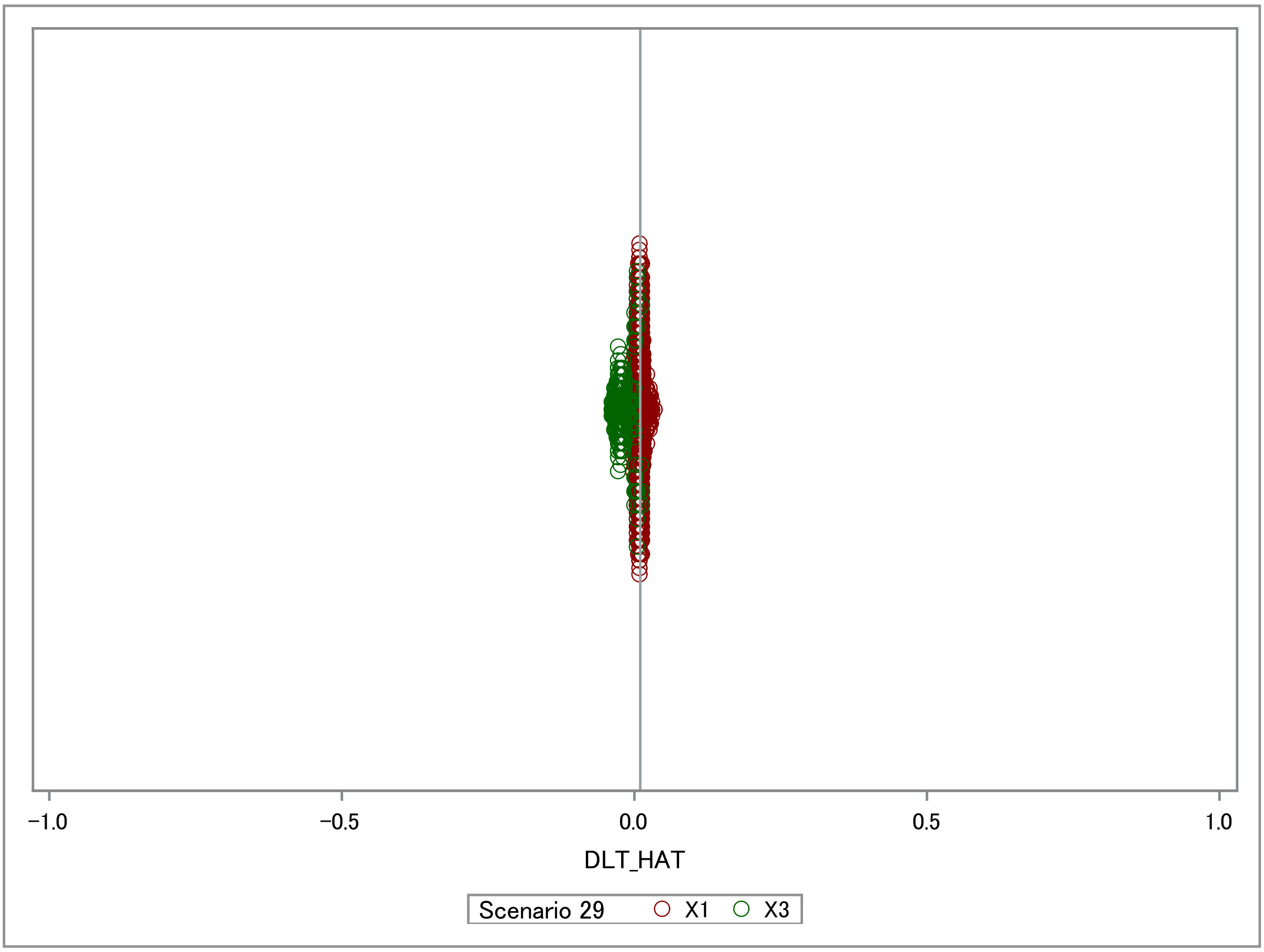
−1.0
−0.5
0.0
0.5
1.0
DLT_HAT
Scenario 29
X1
X3

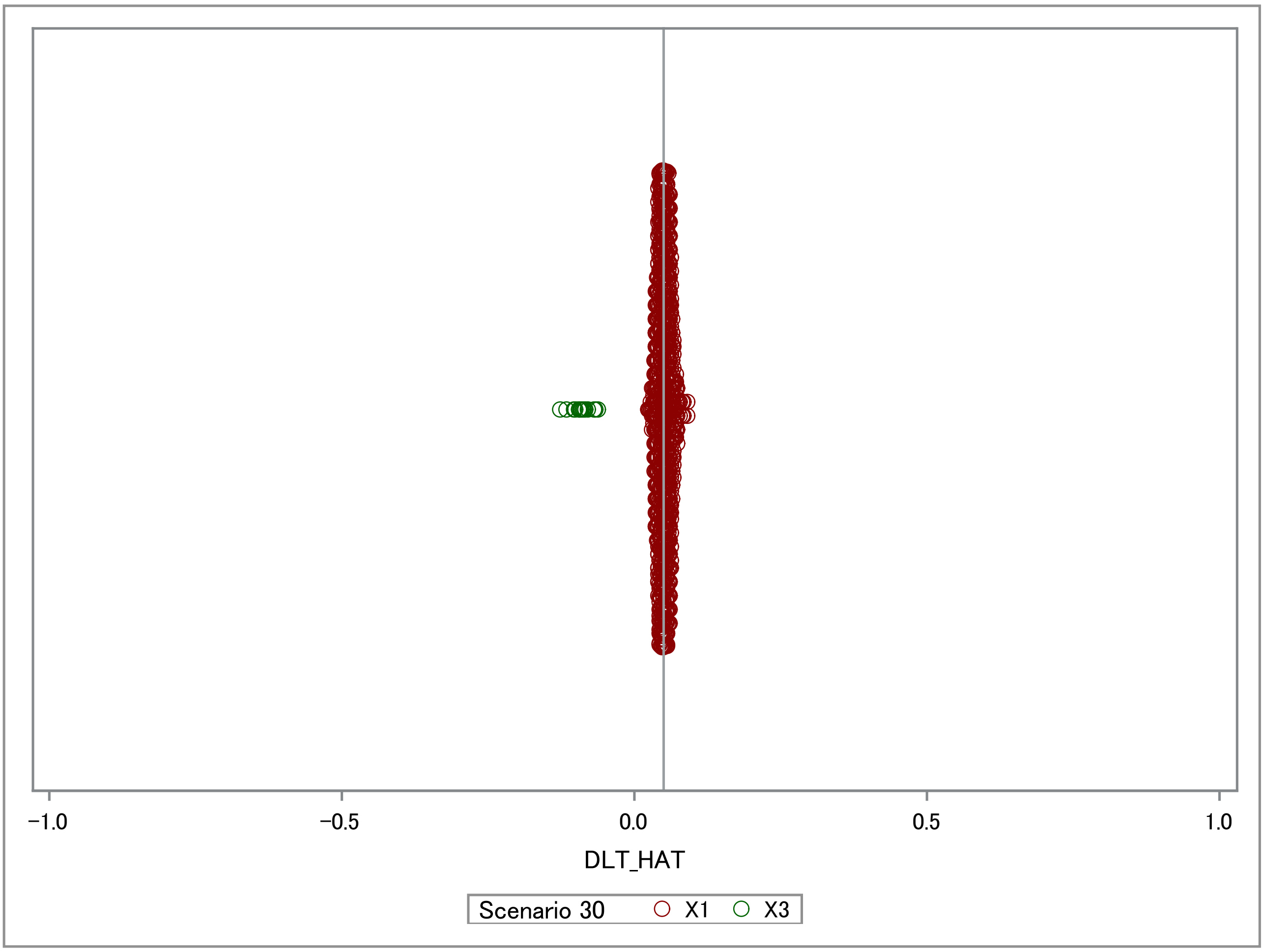

−1.0
−0.5
0.0
0.5
1.0
DLT_HAT
Scenario 30
X1
X3

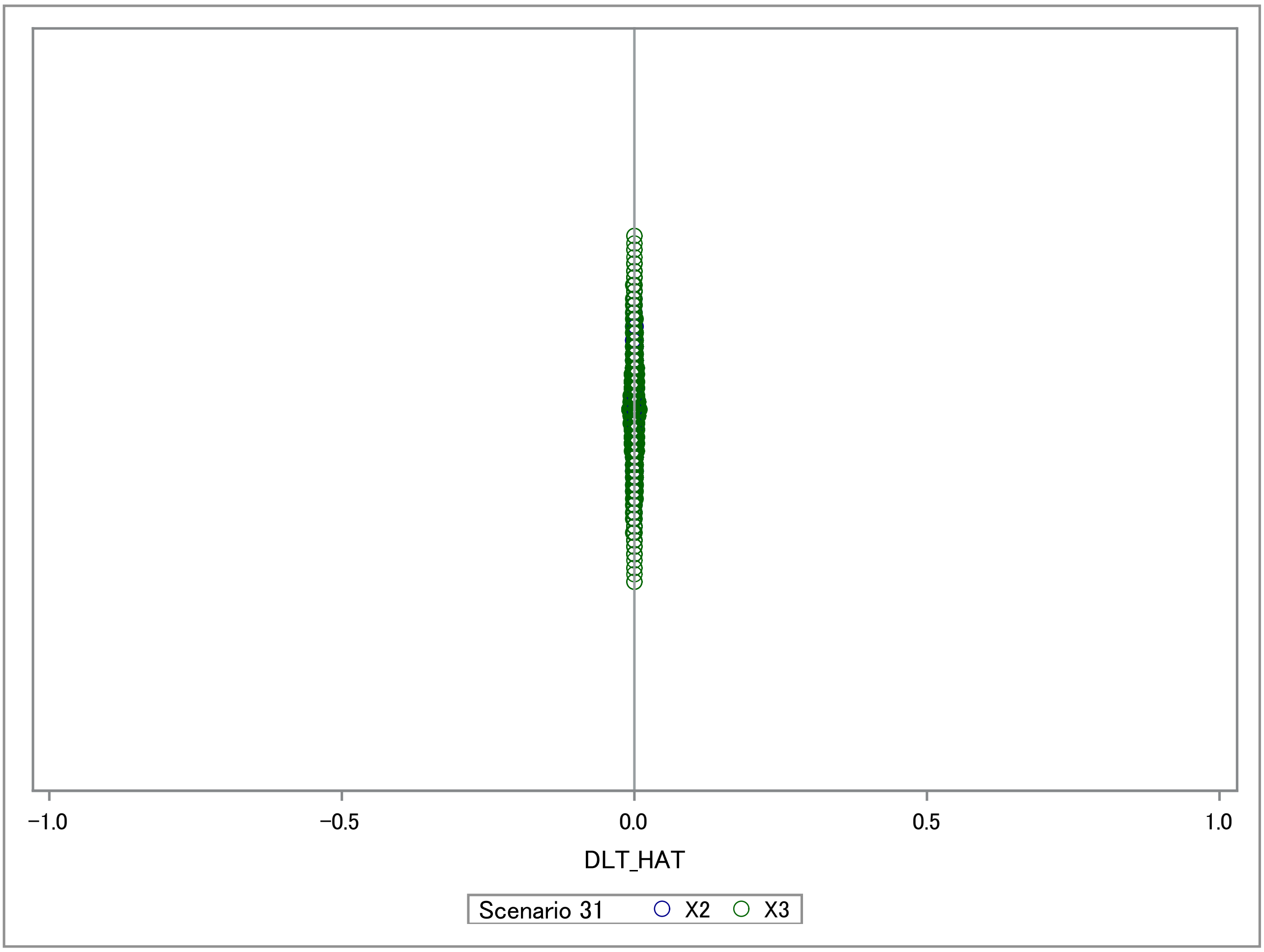
−1.0
−0.5
0.0
0.5
1.0
DLT_HAT
Scenario 31
X2
X3

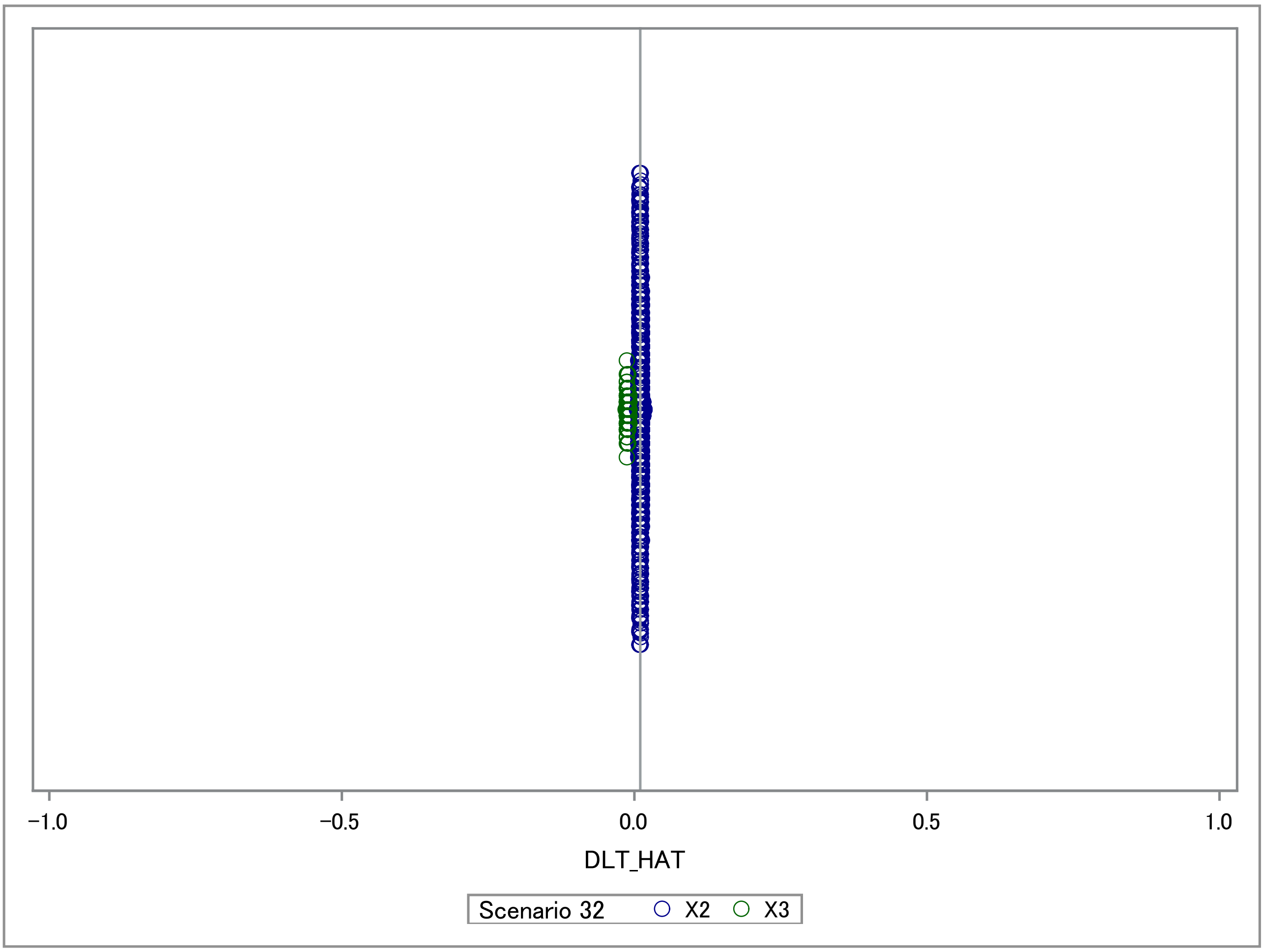
−1.0
−0.5
0.0
0.5
1.0
DLT_HAT
Scenario 32
X2
X3

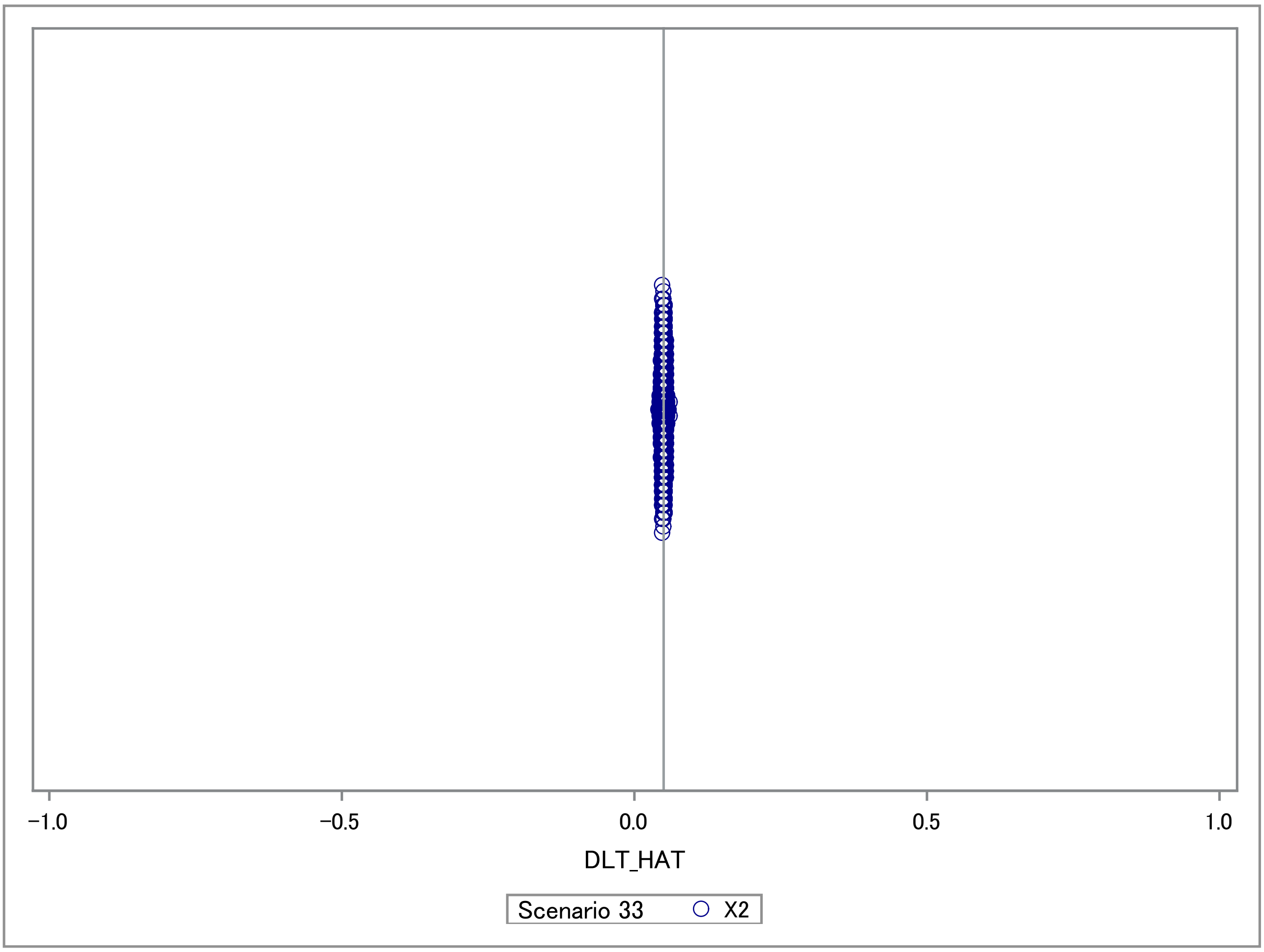
−1.0
−0.5
0.0
0.5
1.0
DLT_HAT
Scenario 33
X2

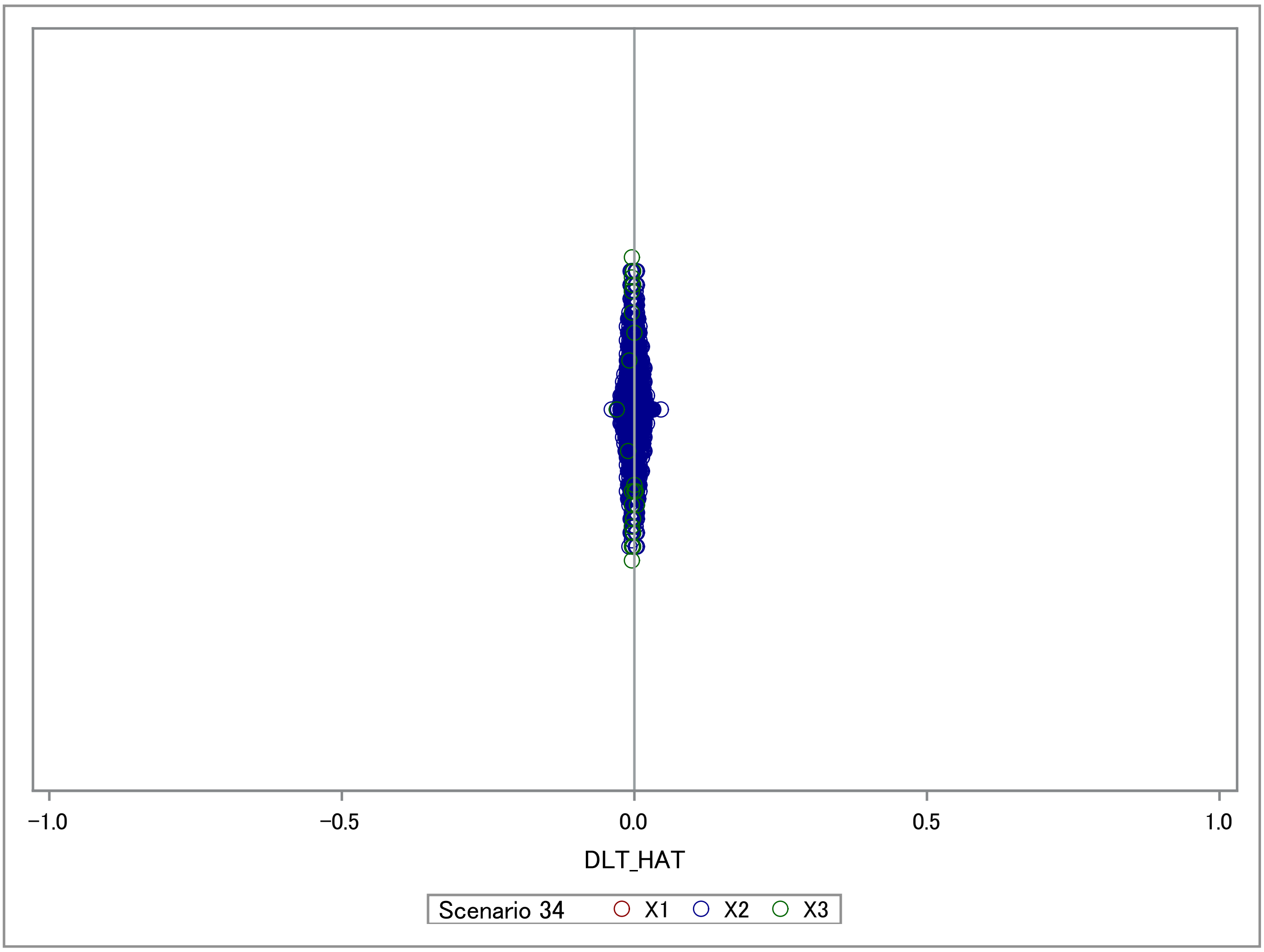
−1.0
−0.5
0.0
0.5
1.0
DLT_HAT
Scenario 34
X1
X2
X3

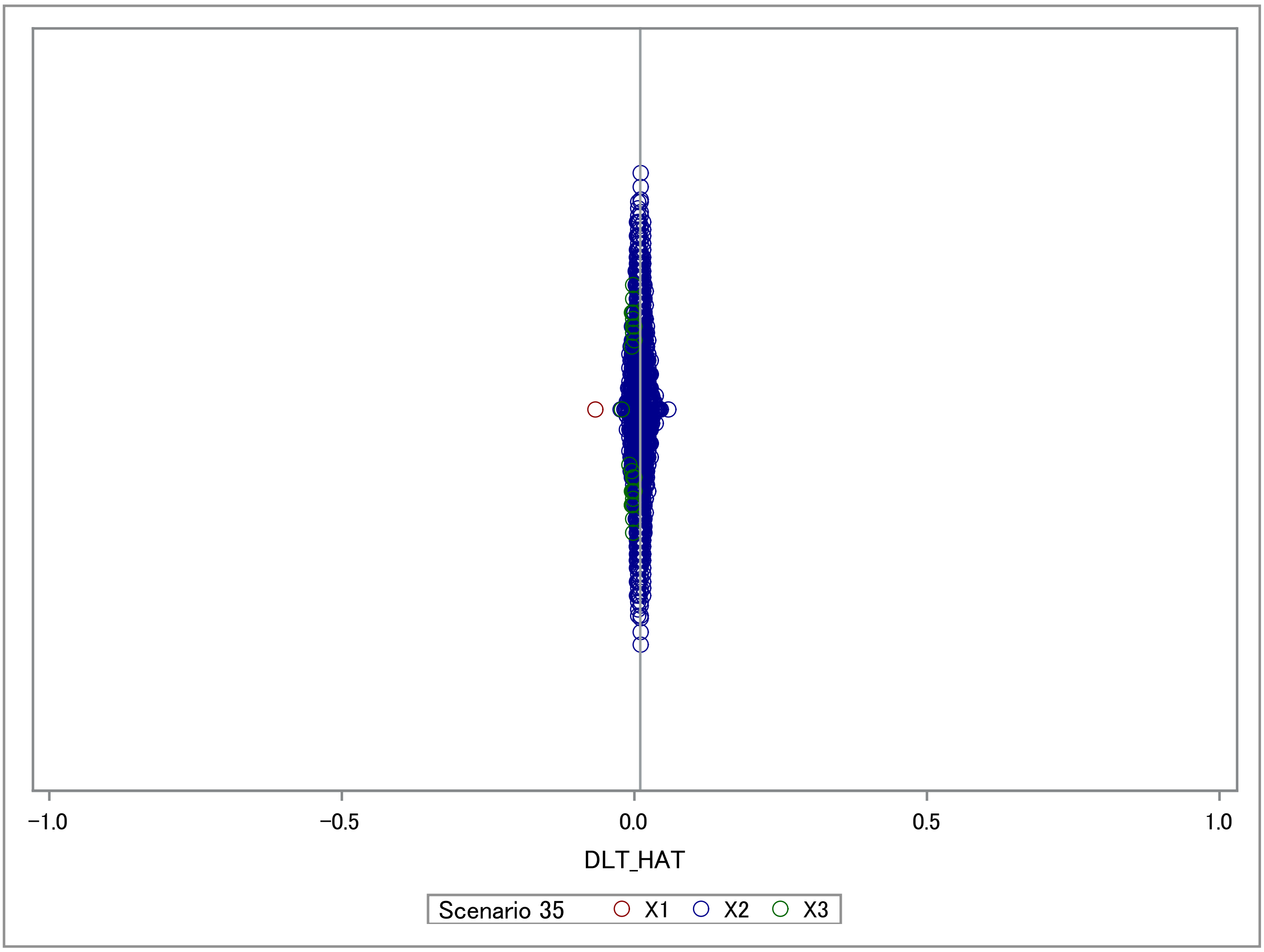
−1.0
−0.5
0.0
0.5
1.0
DLT_HAT
Scenario 35
X1
X2
X3

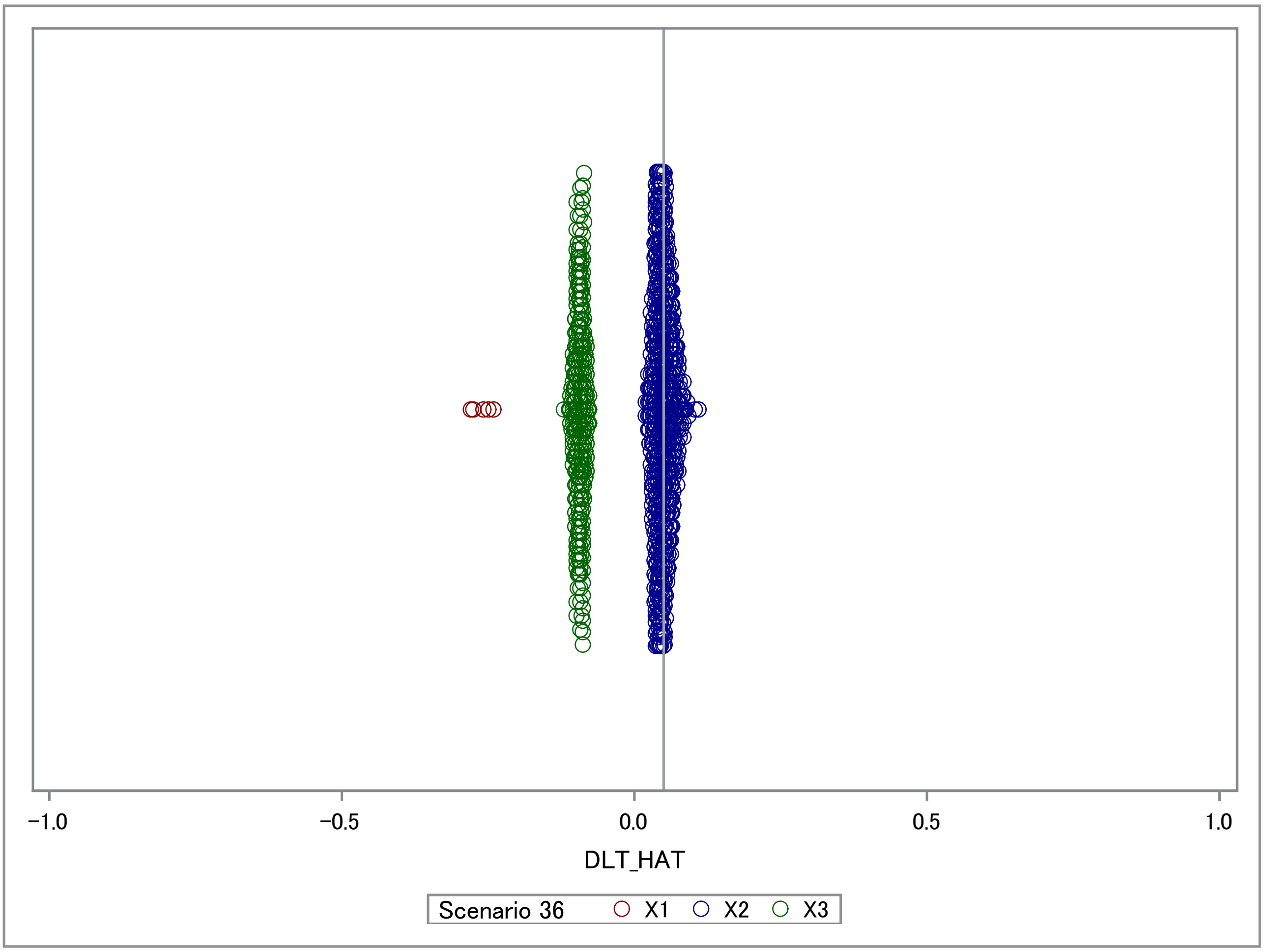
−1.0
−0.5
0.0
0.5
1.0
DLT_HAT
Scenario 36
X1
X2
X3

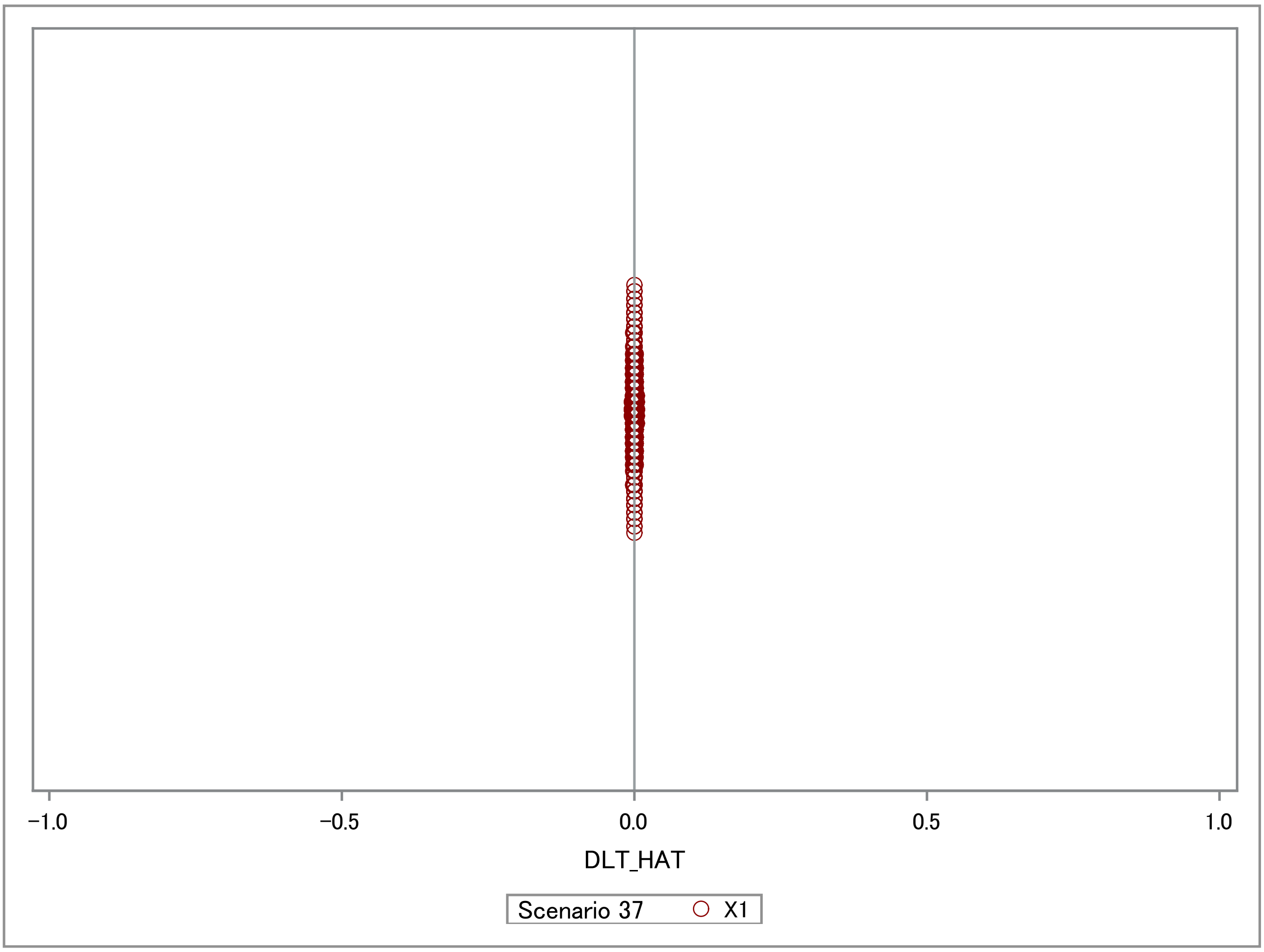
−1.0
−0.5
0.0
0.5
1.0
DLT_HAT
Scenario 37
X1

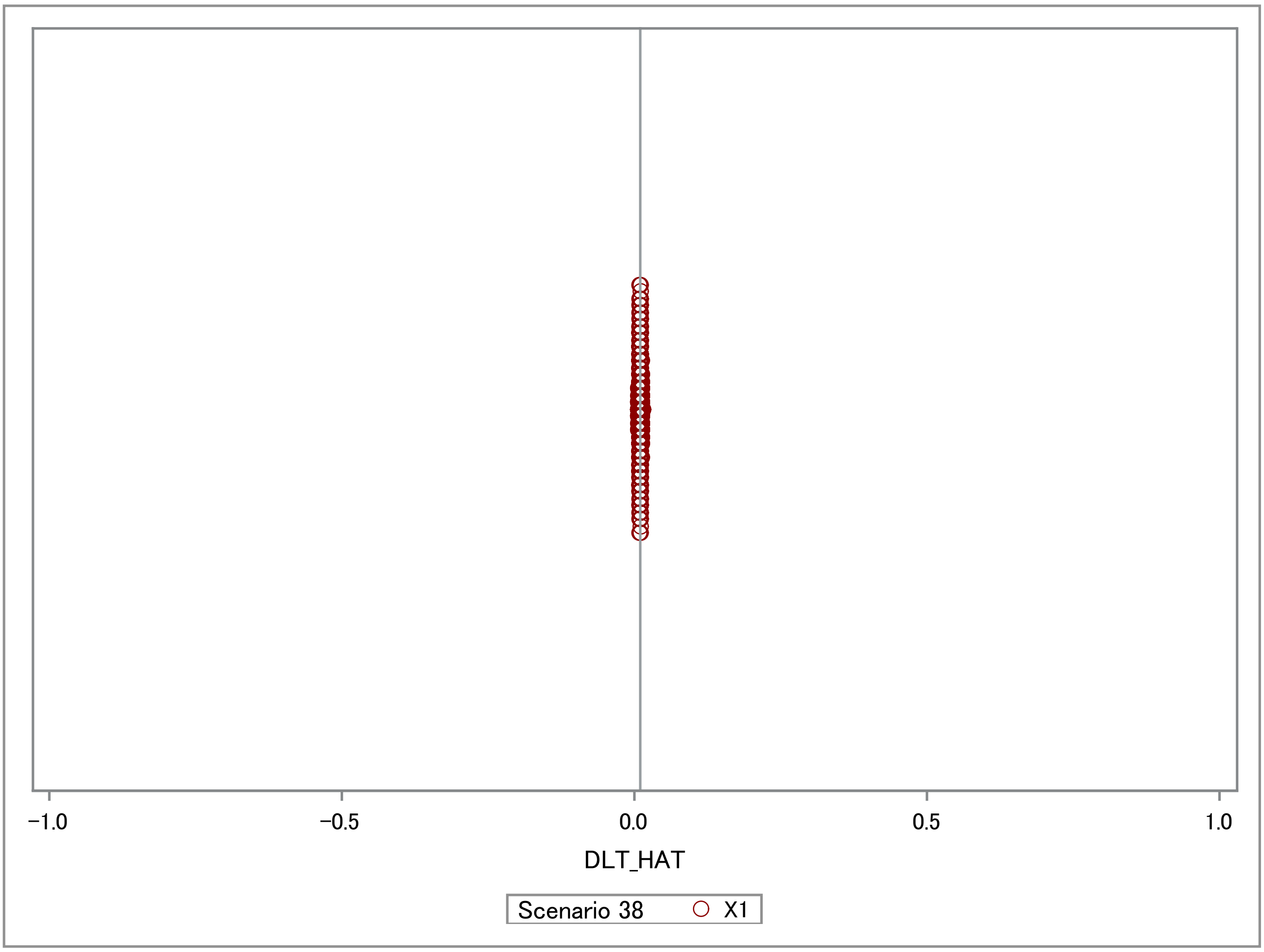
−1.0
−0.5
0.0
0.5
1.0
DLT_HAT
Scenario 38
X1

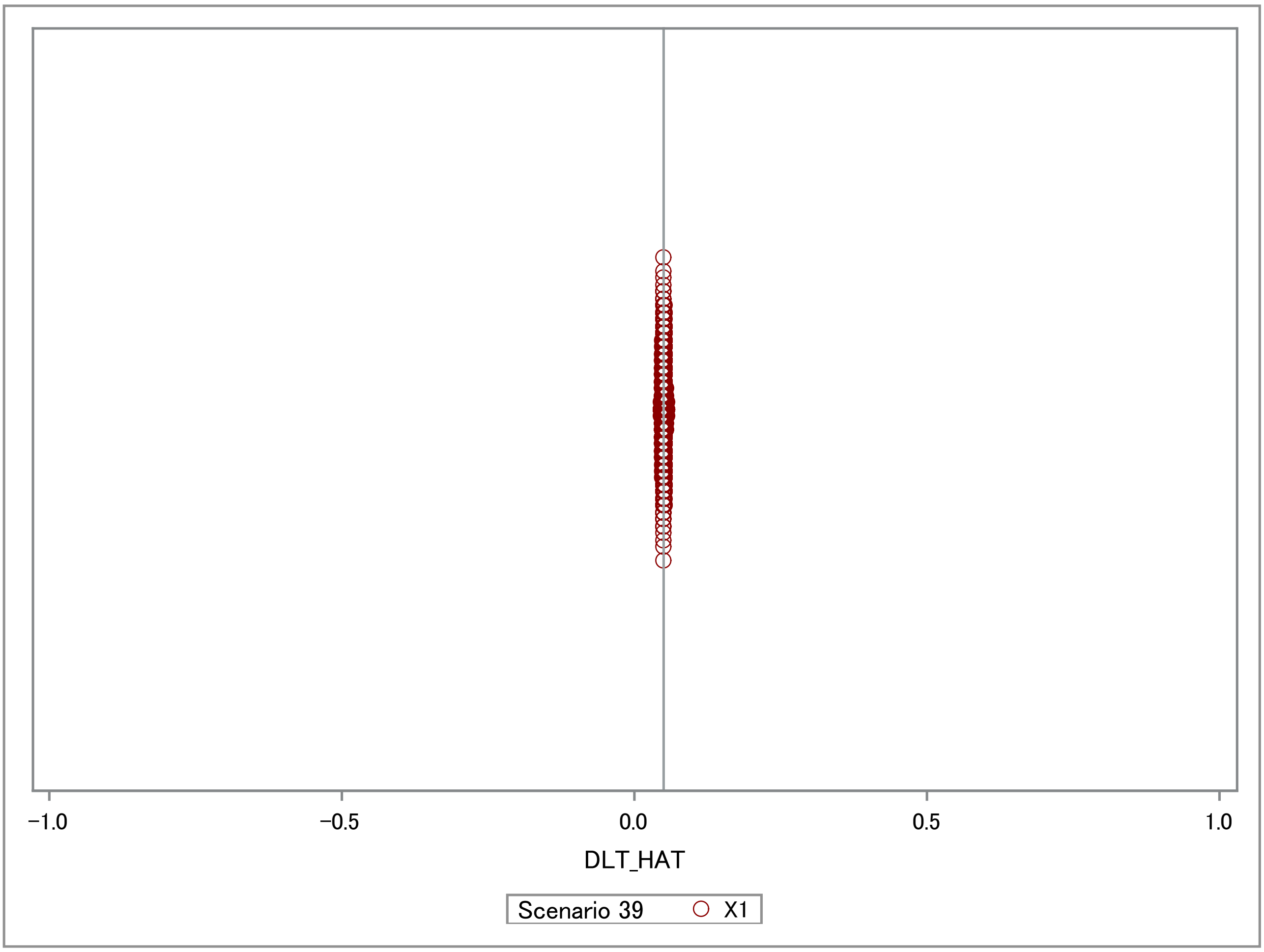
−1.0
−0.5
0.0
0.5
1.0
DLT_HAT
Scenario 39
X1

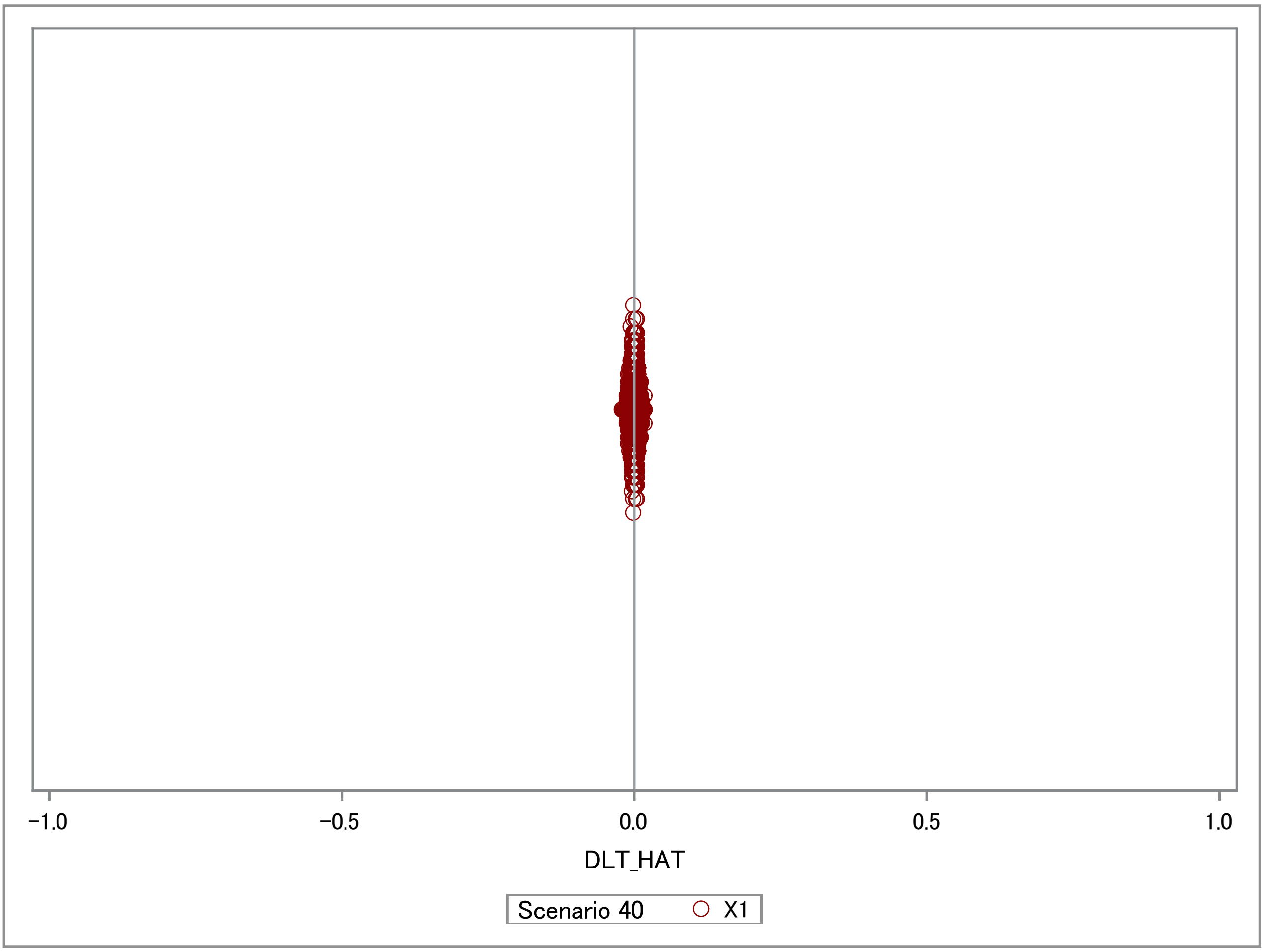

−1.0
−0.5
0.0
0.5
1.0
DLT_HAT
Scenario 40
X1

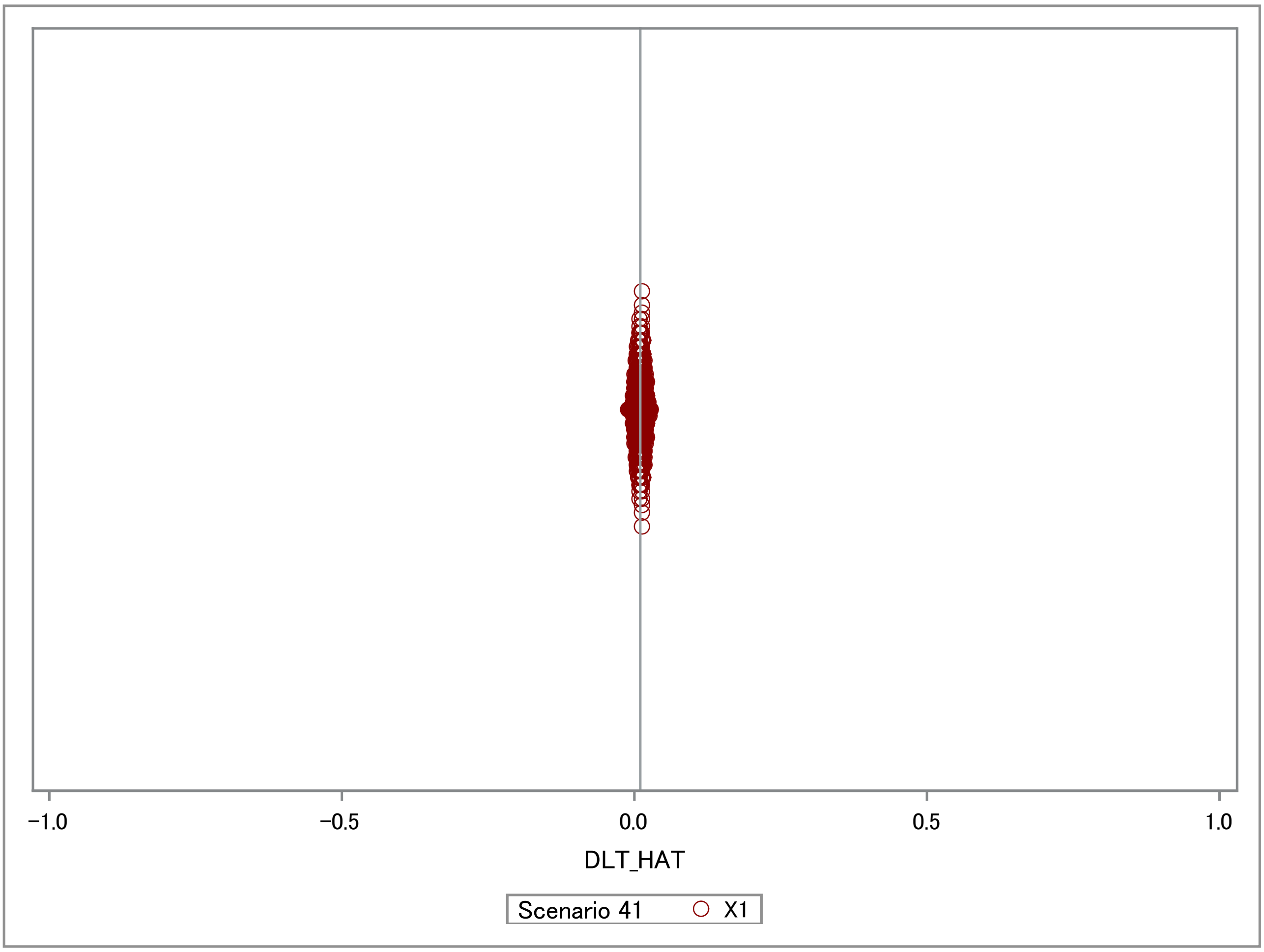

−1.0
−0.5
0.0
0.5
1.0
DLT_HAT
Scenario 41
X1

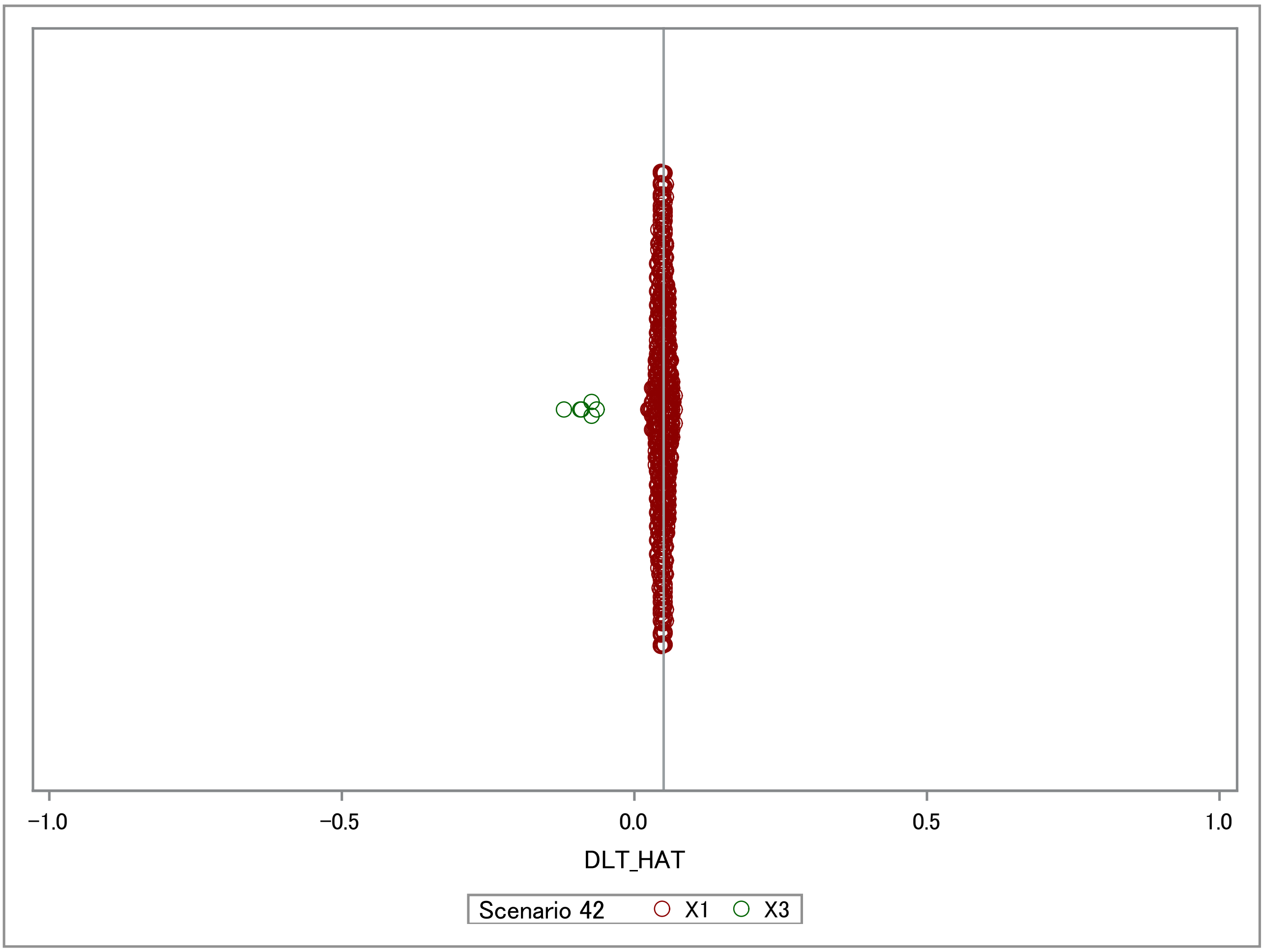
−1.0
−0.5
0.0
0.5
1.0
DLT_HAT
Scenario 42
X1
X3

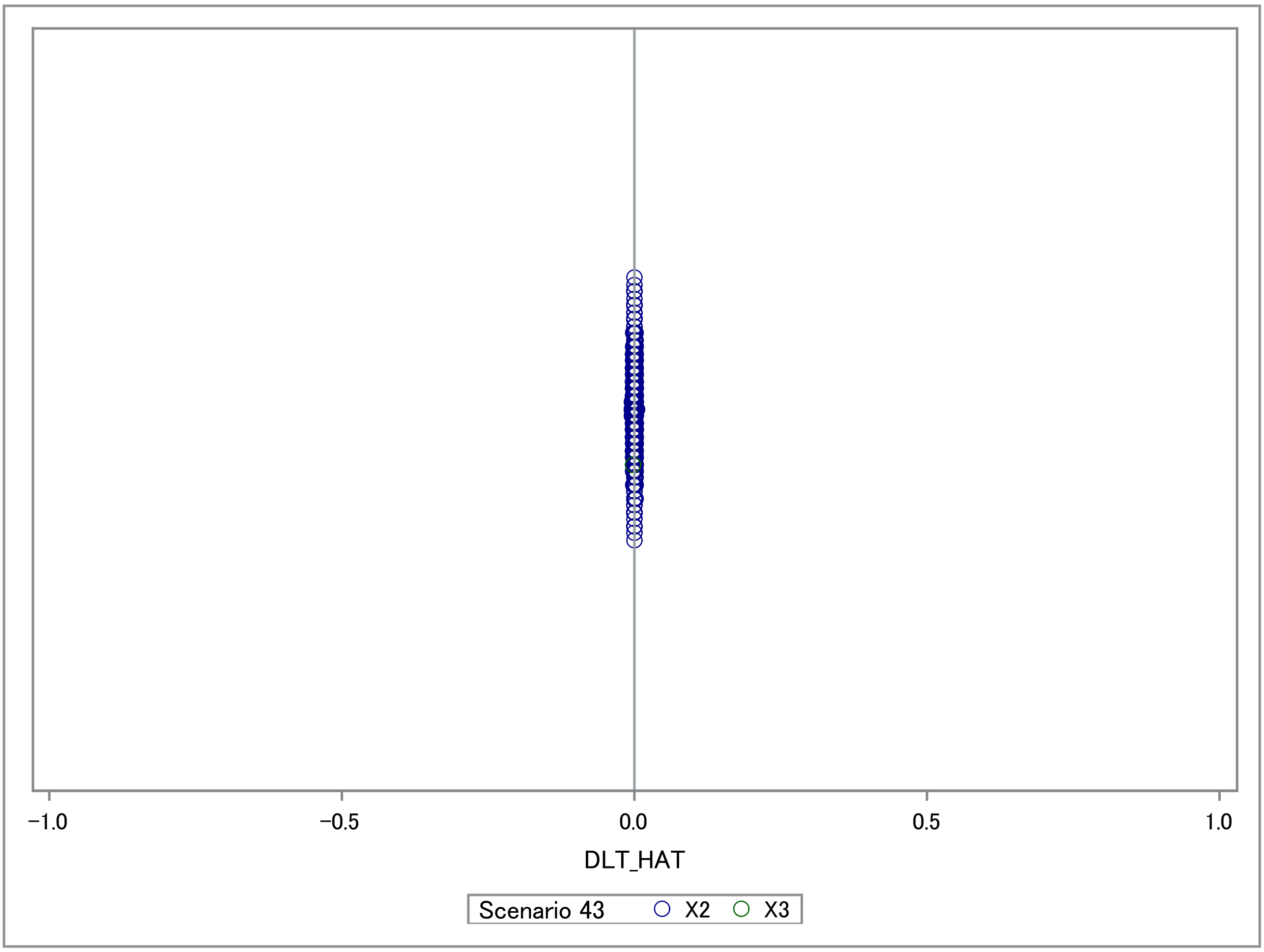
−1.0
−0.5
0.0
0.5
1.0
DLT_HAT
Scenario 43
X2
X3

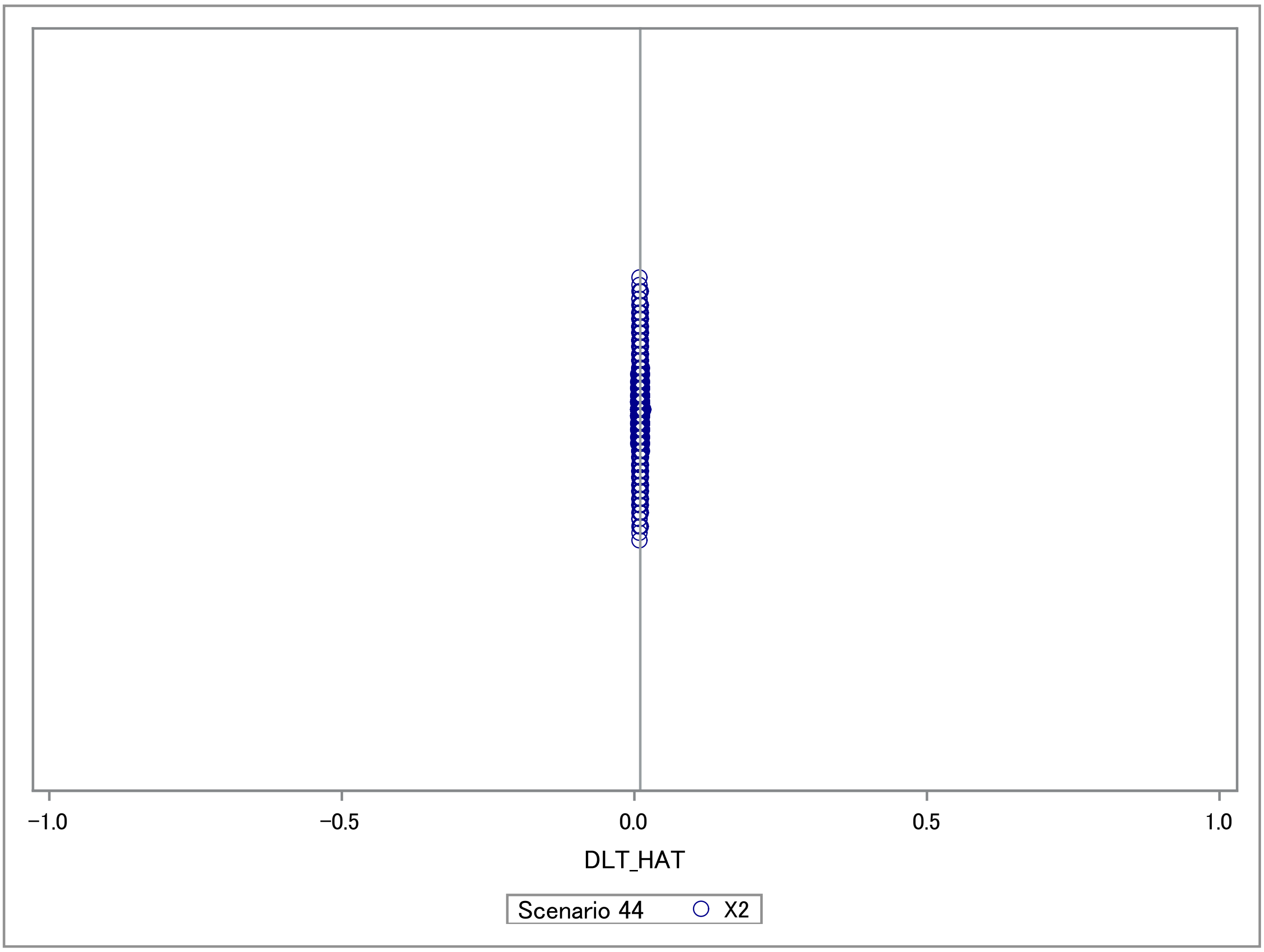
−1.0
−0.5
0.0
0.5
1.0
DLT_HAT
Scenario 44
X2

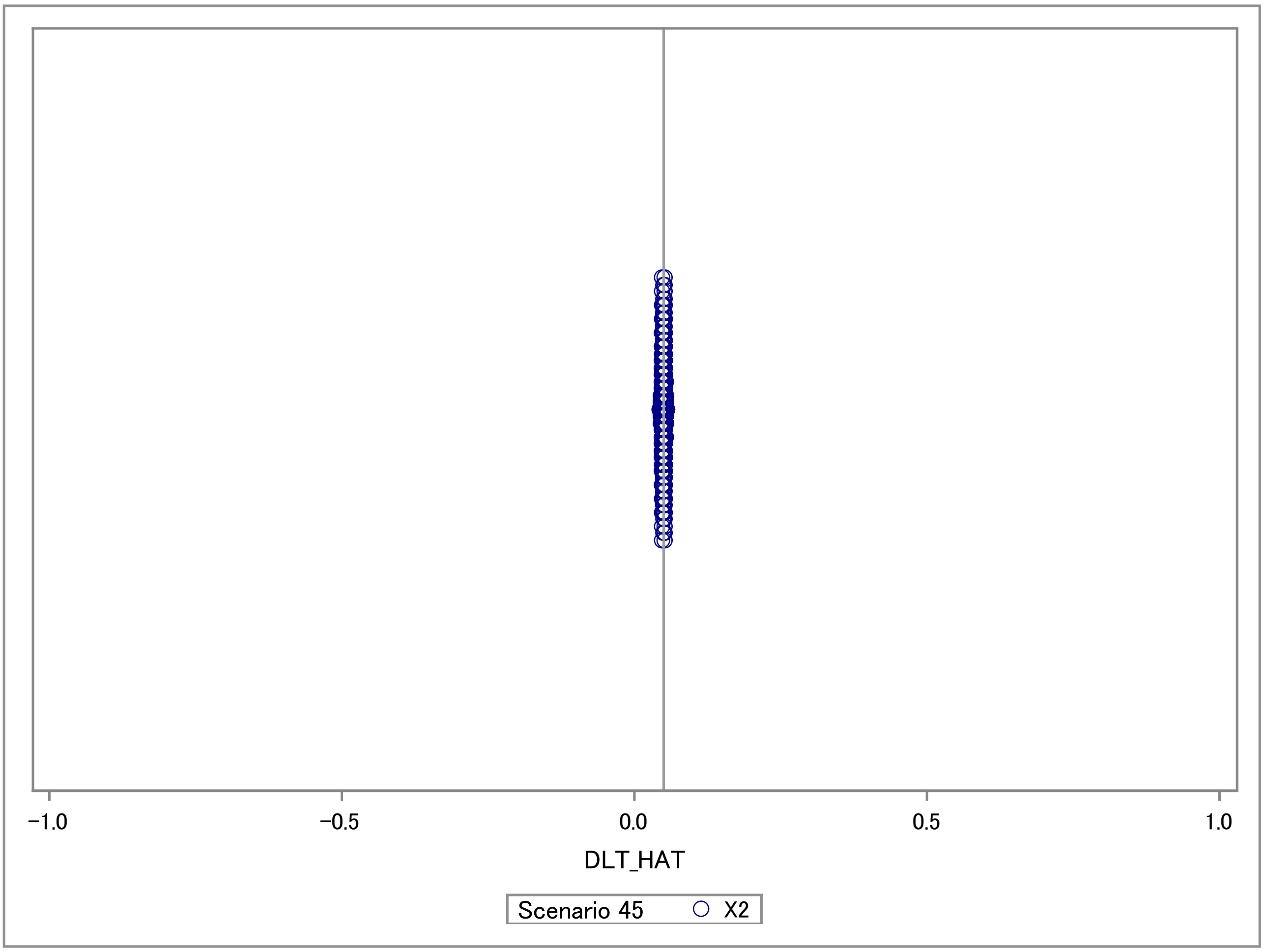
−1.0
−0.5
0.0
0.5
1.0
DLT_HAT
Scenario 45
X2

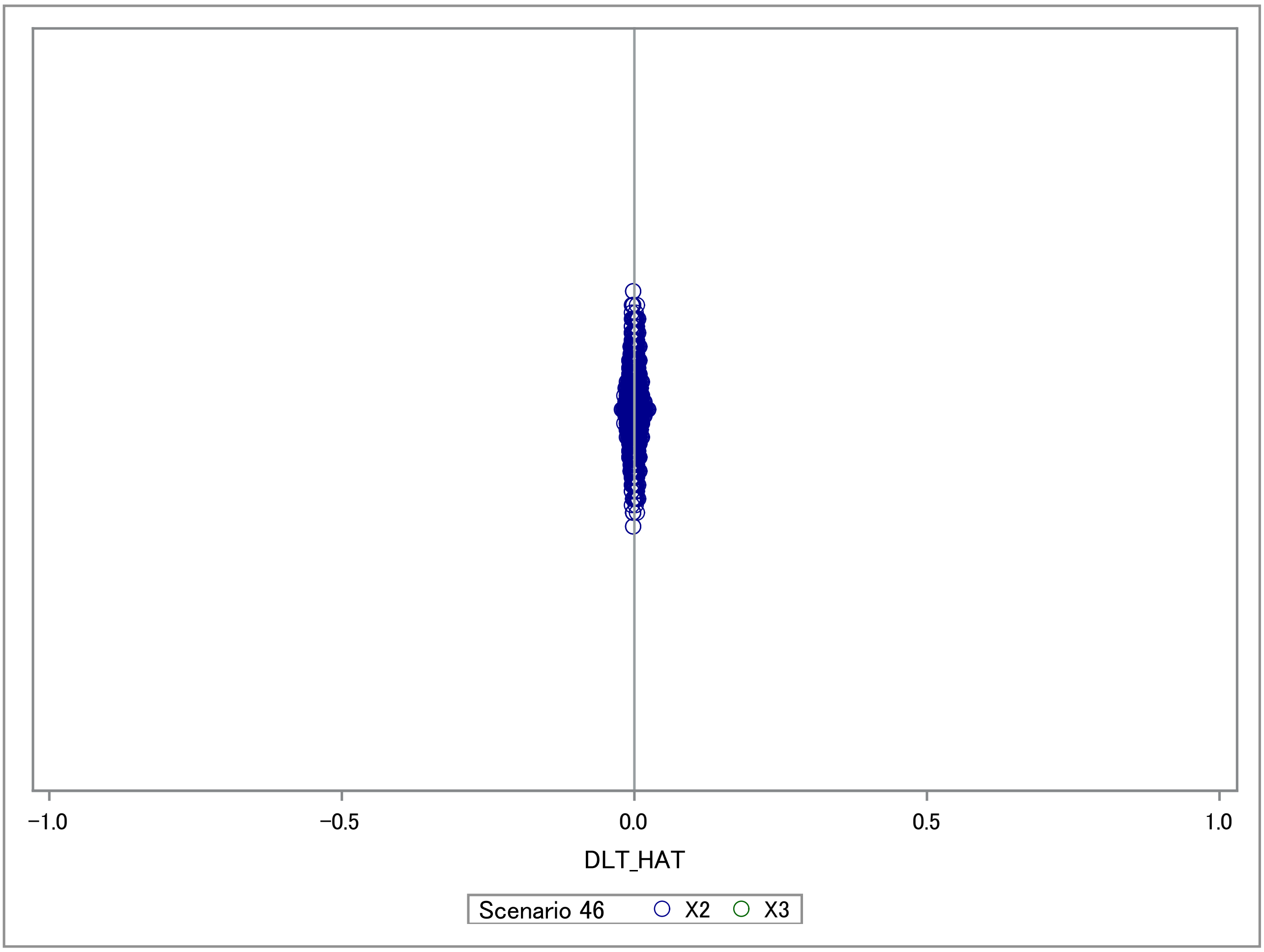
-1.0
-0.5
0.0
0.5
1.0
DLT_HAT
Scenario 46
X2
X3

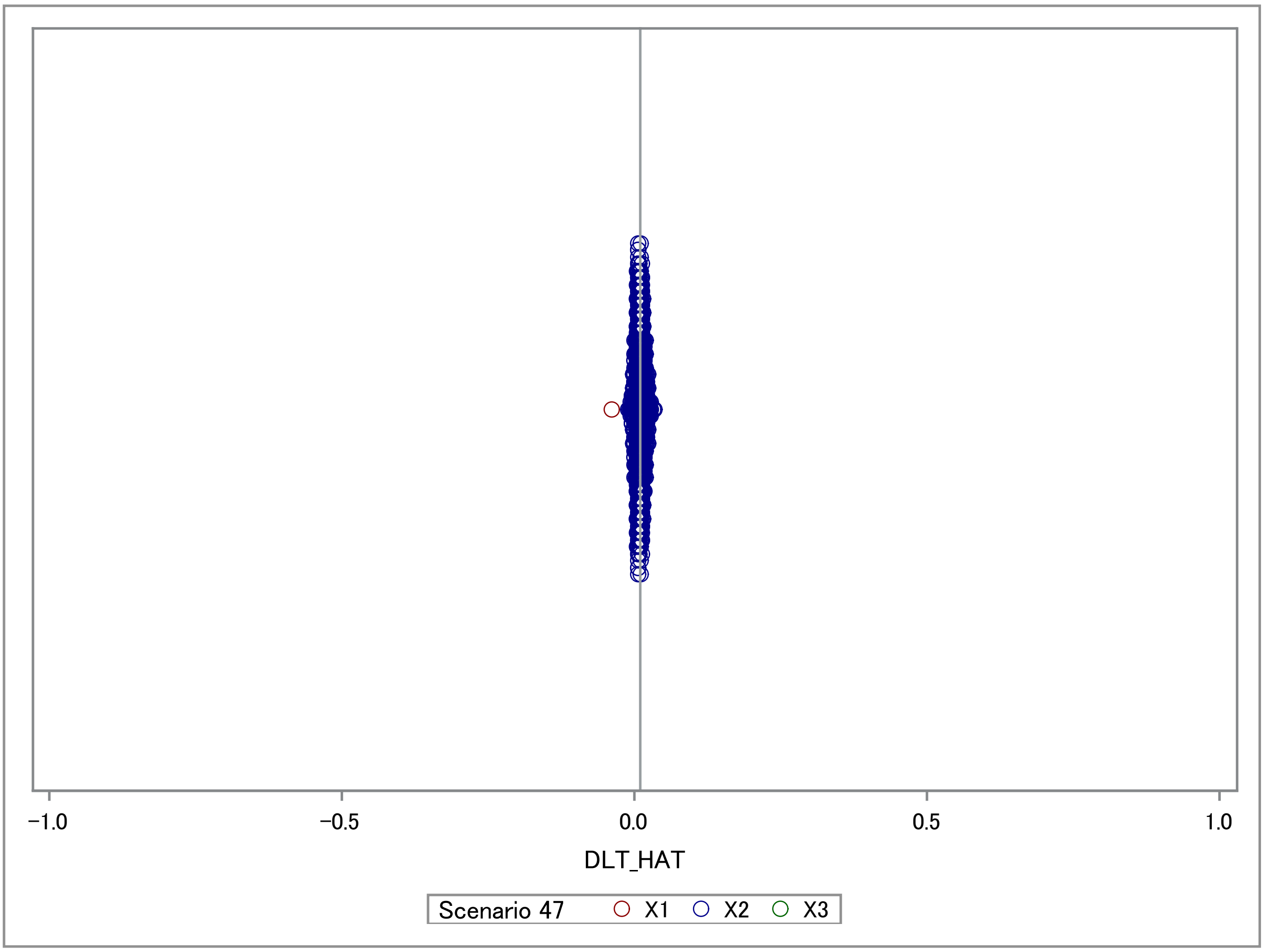
−1.0
−0.5
0.0
0.5
1.0
DLT_HAT
Scenario 47
X1
X2
X3

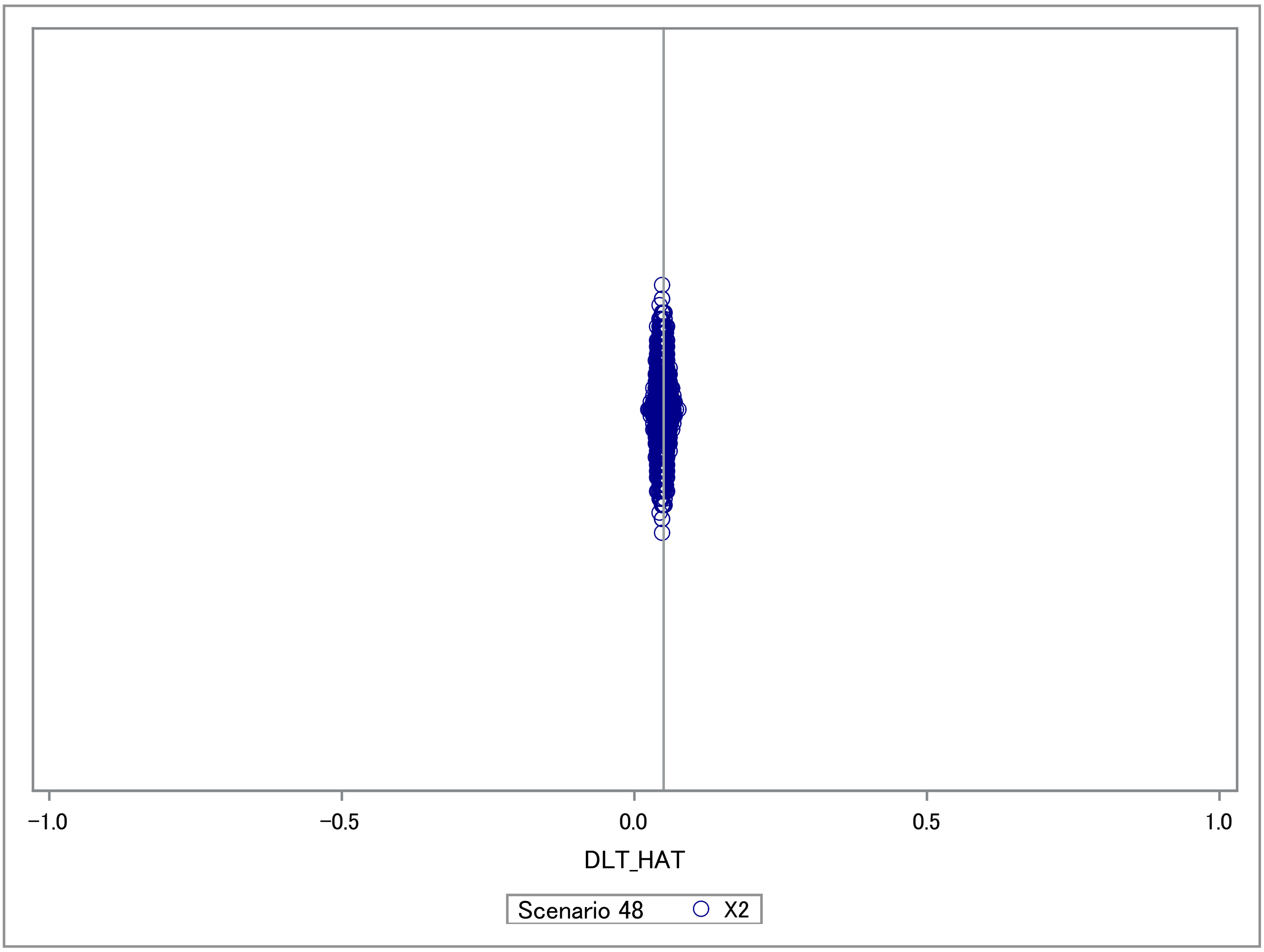

−1.0
−0.5
0.0
0.5
1.0
DLT_HAT
Scenario 48
X2
